# Discovery and Profiling of New Multimodal Phenylglycinamide Derivatives as Potent Antiseizure and Antinociceptive Drug Candidates

**DOI:** 10.1021/acschemneuro.4c00438

**Published:** 2024-08-21

**Authors:** Marcin Jakubiec, Michał Abram, Mirosław Zagaja, Katarzyna Socała, Vanja Panic, Gniewomir Latacz, Szczepan Mogilski, Małgorzata Szafarz, Joanna Szala-Rycaj, Jerry Saunders, Peter J. West, Dorota Nieoczym, Katarzyna Przejczowska-Pomierny, Bartłomiej Szulczyk, Anna Krupa, Elżbieta Wyska, Piotr Wlaź, Cameron S. Metcalf, Karen Wilcox, Marta Andres-Mach, Rafał M. Kamiński, Krzysztof Kamiński

**Affiliations:** † Department of Medicinal Chemistry, Faculty of Pharmacy, Jagiellonian University Medical College, Medyczna 9, Cracow 30-688, Poland; ‡ Department of Experimental Pharmacology, Institute of Rural Health, Jaczewskiego 2, Lublin 20-950, Poland; § Department of Animal Physiology and Pharmacology, Institute of Biological Sciences, Faculty of Biology and Biotechnology, Maria Curie-Skłodowska University, Akademicka 19, Lublin 20-033, Poland; ∥ Department of Pharmacology and Toxicology, University of Utah, Salt Lake City, Utah 84112, United States; ⊥ Department of Technology and Biotechnology of Drugs, Faculty of Pharmacy, Jagiellonian University Medical College, Medyczna 9, Cracow 30-688, Poland; # Department Pharmacodynamics, Faculty of Pharmacy, Jagiellonian University Medical College, Medyczna 9, Cracow 30-688, Poland; ¶ Department of Pharmacokinetics and Physical Pharmacy, Faculty of Pharmacy, Jagiellonian University Medical College, Medyczna 9, Cracow 30-688, Poland; ∇ Chair and Department of Pharmacotherapy and Pharmaceutical Care, Centre for Preclinical Research and Technology, Medical University of Warsaw, Banacha 1B, Warsaw 02-097, Poland; ○ Department of Pharmaceutical Technology and Biopharmaceutics, Jagiellonian University Medical College, Medyczna 9, Cracow 30-688, Poland

**Keywords:** hybrid molecules, multimechanistic compounds, antiseizure activity, antinociceptive activity, *in vitro* functional studies, *in vitro* ADME-Tox studies

## Abstract

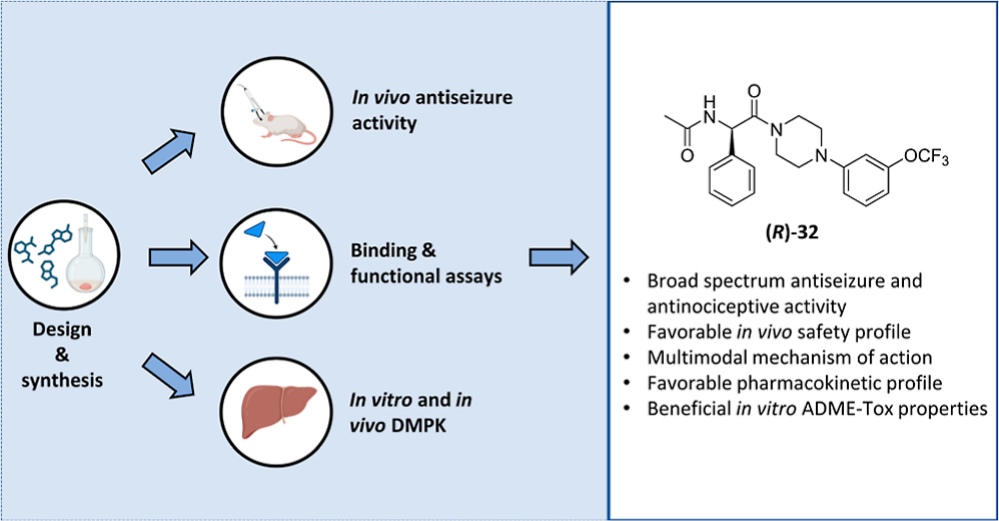

We developed a focused series of original phenyl-glycinamide derivatives which showed potent activity across *in vivo* mouse seizure models, namely, maximal electroshock (MES) and 6 Hz (using both 32 and 44 mA current intensities) seizure models. Following intraperitoneal (*i.p*.) administration, compound **(*R*)-32**, which was identified as a lead molecule, demonstrated potent protection against all seizure models with ED_50_ values of 73.9 mg/kg (MES test), 18.8 mg/kg (6 Hz, 32 mA test), and 26.5 mg/kg (6 Hz, 44 mA test). Furthermore, **(*R*)-32** demonstrated efficacy in both the PTZ-induced kindling paradigm and the *iv*PTZ seizure threshold test. The expression of neurotrophic factors, such as mature brain-derived neurotrophic factor (mBDNF) and nerve growth factor (NGF), in the hippocampus and/or cortex of mice, and the levels of glutamate and GABA were normalized after PTZ-induced kindling by **(*R*)-32**. Importantly, besides antiseizure activity, (***R***)**-32** demonstrated potent antinociceptive efficacy in formalin-induced pain, capsaicin-induced pain, as well as oxaliplatin- and streptozotocin-induced peripheral neuropathy in mice (*i.p*.). No influence on muscular strength and body temperature in mice was observed. Pharmacokinetic studies and *in vitro* ADME-Tox data (*i.e.*, high metabolic stability in human liver microsomes, a weak influence on CYPs, no hepatotoxicity, satisfactory passive transport, *etc.*) proved favorable drug-like properties of (***R***)**-32**. Thermal stability of (***R***)-**32** shown in thermogravimetry and differential scanning calorimetry gives the opportunity to develop innovative oral solid dosage forms loaded with this compound. The *in vitro* binding and functional assays indicated its multimodal mechanism of action. (***R***)**-32**, beyond TRPV1 antagonism, inhibited calcium and sodium currents at a concentration of 10 μM. Therefore, the data obtained in the current studies justify a more detailed preclinical development of (***R***)**-32** for epilepsy and pain indications.

## Introduction

Epilepsy is one of the most common brain diseases, which affects over 70 million people worldwide.^1^ It is characterized by a predisposition to generate spontaneous seizures and has numerous serious consequences such as neurobiological, cognitive, and psychosocial deficits. Epilepsy may have discernible structural, mechanic, infectious, metabolic, and immune etiologies; however, in most people with epilepsy, no obvious cause is identifiable.^2^ The above-mentioned multifactorial pathogenesis of epilepsy often limits substantially the clinical efficacy of currently available antiseizure medications (ASMs). Despite the unquestionable progress in the development of new ASMs and novel therapies, approximately 30% of epilepsy patients still experience uncontrolled and debilitating seizures and suffer from so-called drug-resistant epilepsy (DRE).^3^ The mechanisms underlying drug resistance in epilepsy are also not well understood, which in combination with its multifactorial etiology complicates the optimal selection of ASMs for rational pharmacotherapy.

Neuropathic pain is another serious neurological disorder, which affects 7–10% of the general population, among which 20–30% experience chronic pain.^4^ In this group, only 50% of patients experience pain relief with pharmacotherapy, which reduces neuropathic pain sensation by 30–50%.^4^ Currently, ASMs (*i.e.*, pregabalin and gabapentin) and antidepressants (i.e., amitriptyline, nortriptyline, and duloxetine) are the most commonly used medications for the pharmacotherapy of neuropathic pain.^5,6^ It is postulated that epilepsy and neuropathic pain may have similar neurobiological underpinnings, as reflected by the use of the same pharmacological therapies.^7^ Therefore, it seems advisable that novel ASMs under development should also be evaluated in models of neuropathic pain.

The current strategy for the effective management of epilepsy and neuropathic pain relies mainly on use of combination pharmacotherapy or application of drugs preferably with multimodal (multitarget/multifunctional/multimechanistic) pharmacodynamics, acting on several complementary biological targets.^8,9^ Furthermore, multimodal compounds seem to be especially useful for diseases with a high risk of drug resistance, such as cancer, Parkinson’s disease, Alzheimer’s disease, depression and finally epilepsy, and neuropathic pain.^10−16^ Multitarget compounds, which usually have chimeric or hybrid structures, may include many pharmacophores inside a single chemical scaffold, which enables a broad interaction with therapeutic and, ideally, complementary molecular targets.^17^ Beyond the previously mentioned benefits, the multitarget compounds may also reduce the overall drug burden (especially during combination therapy), and as a result, may reduce the risk of potential drug–drug interactions (DDIs) and multiple side effects, improving therapy compliance.^18−20^

Due to the multifactorial origin of epilepsy, the search for new more effective ASMs with multitarget pharmacodynamics should be directed preferentially toward new molecular targets and/or combining these new targets with already known mechanisms for ASMs. Such a strategy may yield compounds effective in DRE and/or, favorably, substances with disease modifying or antiepileptogenic properties. Consequently, in recent years, a number of potential and promising targets for ASMs have emerged.^21^ Among them is the transient receptor potential vanilloid type 1 (TRPV1) channel, which has been recognized for many years as one of the most extensively researched molecular targets for novel and strong analgesics.^22^ The TRPV1 channel is a member of the TRP channel family, which is found in plasma membranes and whose members are essential for the transcellular transport and/or influx of different ions such Na^+^, K^+^, Ca^2+^, and Mg^2+^. TRPs are implicated in various and important physiological processes, like sensory functions (*i.e.*, thermosensation, nociception, taste transduction) ion homeostasis, as well as many other motile functions, such as muscle contraction and vasomotor control.^23−25^ The TRPV1 channel, also called a capsaicin receptor, is the best characterized among TRPs. As a nonselective cation channel, it is permeable to sodium and calcium, which generate ionic currents responsible for depolarization and action potential firing. Importantly, recent studies have shown that TRPV1 is widely distributed in the central nervous system (CNS—*i.e.*, cortex, hippocampus, *etc.*) beyond the peripheral nervous system,^26^ and it may play an important role in the induction of seizures and propagation of epileptogenesis.^27−29^ Therefore, it is hypothesized that compounds having TRPV1 antagonist activity could be viable candidates for ASMs characterized by antiepileptogenic and disease-modifying properties as well as additionally characterized by potent central and peripheral antinociceptive efficacy. Interestingly, cannabidiol (CBD), one of the newest ASMs effective in DRE (approved among others in Dravet and Lennox-Gastaut syndromes in children), has a multimodal mode of action, with TRPV1 being one of the key molecular targets responsible for its bioactivity.^30^ It is postulated that the multimodal pharmacodynamics of CBD is reflected in a broad spectrum of antiseizure activity across different animal models of seizures in preclinical studies.^31^

**Figure 1:**
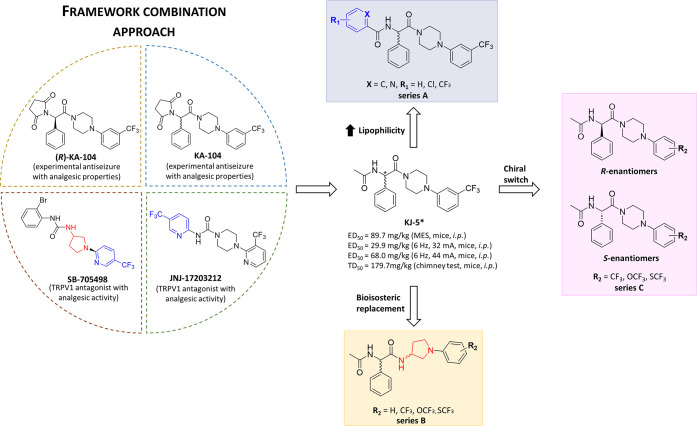
Development process yielding a chemical prototype compound **KJ-5** (*disclosed as compound **53** in Jakubiec *et al.*(38)) and general structures of new hybrid molecules reported herein.

Following the concept of the multitarget strategy, in our previous studies, we have successfully employed a novel strategy for ASM discovery that has been based on chemical hybrid design, taking advantage of the known drug-like structures with a specific mechanism of action and approved ASMs. Consequently, this framework combination approach led to the design and characterization of novel chemical entities with drug-like properties and structurally related mechanisms of action, both multimodal or unprecedent and novel for ASMs (*e.g.*, positive allosteric modulators of GLT1/EAAT-2 transporter of glutamate).^32−35^ The beneficial antiseizure and antinociceptive properties were reported, among others, for compound **KA-104** or its (*R*)-enantiomer, **(*R*)-KA-104** (Figure 1).^34,36,37^ These molecules showed potent and broad-spectrum activity in MES, 6 Hz (32/44 mA), and subcutaneous pentylenetetrazole (*sc*PTZ)-induced seizure models in mice, as well as potent activity in animal models of inflammatory and neuropathic pain (not yet disclosed for **(*R*)-KA-104**). The further chemical modifications of **KA-104** (*i.e.*, opening of the imide ring) resulted in the synthesis of compound **KJ-5**.^38^ Notably, **KJ-5** was designed as a hybrid compound that integrate structural fragments of its chemical prototype—**KA-104** and selective acyclic TRPV1 antagonists such as JNJ-17203212 and SB-705498 with proven analgesic activity in preclinical studies.^22^ Consequently, **KJ-5** demonstrated potent and broad-spectrum antiseizure activity in the MES and 6 Hz (32/44 mA) models in mice and furthermore was characterized by a dual mechanism of action including TRPV1 channel antagonism and blockade of voltage-gated sodium channels.^38^

Taking into consideration beneficial pharmacological properties of **KJ-5** and its congeners, in the present article, we aimed to optimize their CNS drug-like properties based on both structural (physicochemical) optimization and detailed pharmacological characterization. Therefore, in the current studies, we proposed tridirectional modifications A–C of the mentioned hit molecule **KJ-5** (Figure 1): A—introduction of an additional aromatic ring in place of acetyl residue (aiming on increasing lipophilicity and potentially better penetration to the brain) (series A); B—bioisosteric replacement of the piperazine ring into pyrrolidin-3-amine moiety, which is also a well-known pharmacophore for TRPV1 antagonism (*i.e.*, SB-705498) (series B); C—preparation of *R* or *S*-enantiomers for racemates with the most potent antiseizure efficacy identified in our previous studies,^38^ aiming to find spatial configuration preferential for biological activity (series C). It should be stressed here that enantiomers (or diastereoisomers) of drugs or drug candidates often display marked differences in pharmacodynamic, pharmacokinetic, and toxicological properties. The chiral-switch approach of the already marketed racemates and/or development of drug candidates in a predefined enantiomeric/diastereoisomeric form is a useful approach in medicinal chemistry, which helps obtain a better potency or/and lower toxicity for new compounds.^39^ This trend is also strongly visible in the case of ASMs, such as levetiracetam, lacosamide, and eslicarbazepine, which have been implemented in pharmacotherapy as enantiomers.^40^ Therefore, the exact characterization of individual stereoisomers is necessary even in the early stages of drug development, and it is also a crucial step for more detailed preclinical evaluation of compounds described herein.

The current studies utilized an integrated drug discovery approach focused on the development of new ASM candidates, which consists of design, synthesis, and biological characterization. *In vivo* experiments for antiseizure activity using various animal seizure models, including MES, 6 Hz (32 and 44 mA), and intravenous PTZ (*iv*PTZ) seizure tests in mice were conducted for the synthesized compounds. Moreover, the lead compound was evaluated in the PTZ kindling model in mice. Pharmacokinetic and antinociceptive activity studies were carried out for the most promising compounds. We evaluated a number of ADME-Tox properties, including membrane permeability, metabolic stability, hepatotoxicity, and influence on the activity of cytochrome P-450 isoforms, including CYP3A4, CYP2D6, and CYP2C9. These assessments were conducted with the safety profile evaluation in mind, which is essential for the early development of new drug candidates. Finally, we examined the mechanism of action of tested compounds in the *in vitro* studies.

## Results and Discussion

### Chemistry

**Scheme 1:**
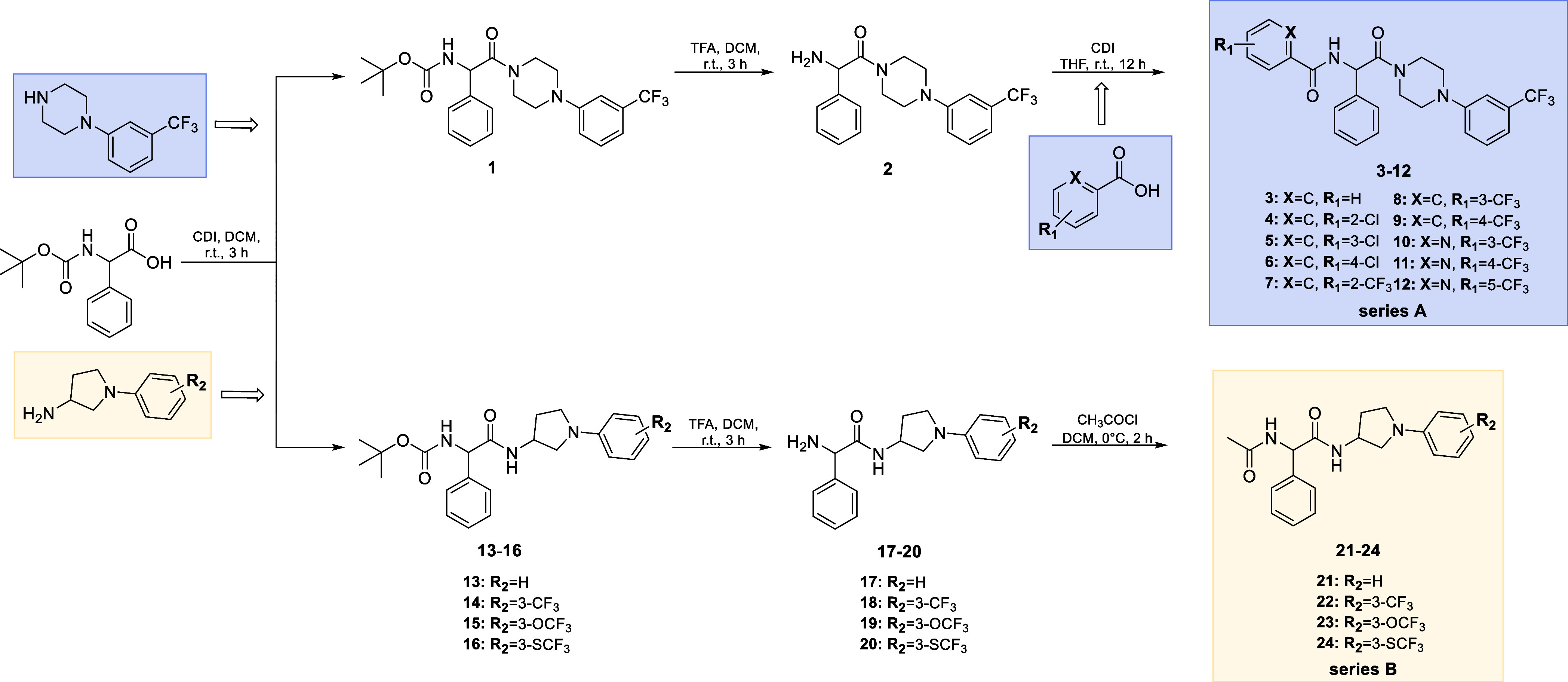
Synthesis of Intermediates and the Final Compounds **3**–**12** (Series A) and **21**–**24** (Series B)

The final compounds **3**–**12** (series A) were obtained applying the multistep synthetic pathway according to Scheme 1. Initially, Boc-protected intermediate **1** was produced by the coupling reaction of 1-(3-(trifluoromethyl)phenyl)piperazine with Boc-d,l-phenylglycine in the presence of carbonyldiimidazole (CDI). Then, removal of the Boc group with trifluoroacetic acid (TFA), followed by neutralization with ammonium hydroxide gave amine derivative **2**. The target compounds **3**–**12** were obtained in a condensation reaction of **2** with appropriate benzoic acid or pyridine-2-carboxylic acid derivatives in the presence of CDI. The crude products were purified by applying column chromatography. The desired compounds were obtained as white solids, followed by crystallization from methanol.

Compounds belonging to series B are 3-amino-pyrrolidine analogues of the most potent antiseizure molecules identified in our previous studies,^36,38^ containing an unsubstituted amine or 3-CF_3_, 3-OCF_3_, and 3-SCF_3_ phenylpiperazines. Noncommercial 1-phenylpyrrolidin-3-amine derivatives (**A5**–**A8**) were created in a two-step process in accordance with Scheme S1 as the initial stage of the synthesis pathway. First, aryl bromides reacted with *N*-Boc-3-amine-pyrrolidine in a Buchwald-Hartwig reaction^41^ in a nitrogen atmosphere to produce the Boc-protected intermediates **A1**–**A4**. Then, removal of the Boc group with TFA was followed by neutralization with 25% ammonium hydroxide and yielded the desired 1-phenylpyrrolidin-3-amine derivatives **A5**–**A8**, which were used for the next reactions without purification (for details, see Supporting Information). The desired compounds **21**–**24** (series B) were synthesized according to a similar procedure described above, using 1-phenylpyrrolidin-3-amine derivatives (**A5**–**A8**) and Boc-d,l-phenylglycine as substrates (Scheme 1). The last stage involved acetyl chloride’s acylation reaction with amine derivatives (**17**–**20**). Compounds **21**–**24** were obtained as solids after purification by column chromatography, followed by wash-up with diethyl ether.

**Scheme 2:**
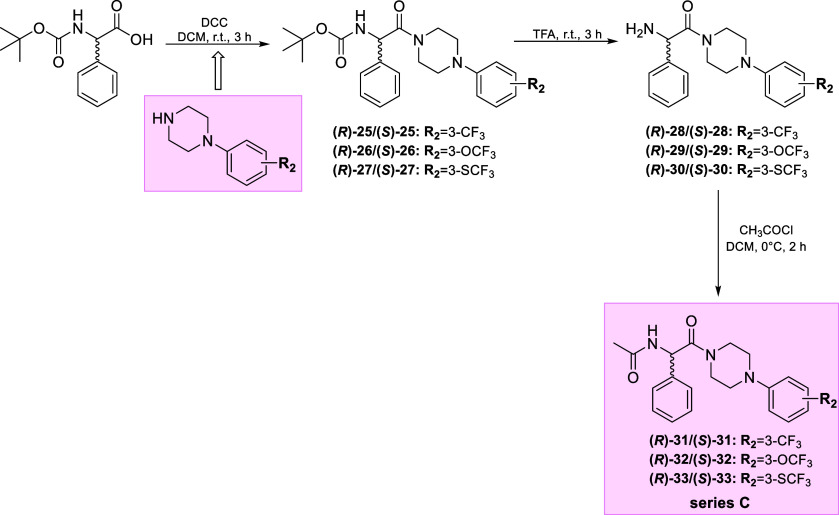
Synthesis of Intermediates and the Final Enantiomers **(*R*)-31–(*R*)-33** and **(*S*)-31–(*S*)-33** (Series C)

The enantiomers (**(*R*)-31–(*R*)-33** and **(*S*)-31–(*S*)-33**) (series C) were obtained according to the procedure depicted in Scheme 2. First, the coupling reaction of the commercially available Boc-d-phenylglycine or Boc-l-phenylglycine with the appropriate phenylpiperazine derivatives (according to the synthetic pathway described in previous studies^38^) in the presence of *N,N′*-Dicyclohexylcarbodiimide (DCC) as the coupling agent yielded Boc-protected intermediates **(*R*)-25–(*R*)-27** and **(*S*)-25–(*S*)-27**. Then, by removal of the Boc protecting group by addition of TFA, the respective amine derivatives **(*R*)-28–(*R*)-30** and **(*S*)-28–(*S*)-30** were obtained. In the last step, these amines were converted to the final compounds **(*R*)-31–(*R*)-33** and **(*S*)-31–(*S*)-33** in acylation reaction with acetyl chloride. Column chromatography was used to purify the crude products. Following the concentration of organic solvents and diethyl ether wash-up, the final compounds were obtained as white powders. The enantiomeric purity of the final compounds was *>* 99%, as determined by chiral HPLC analysis. In the current studies, we decided to restrict the number of enantiomers obtained only to stereoisomers of the most active racemates described in the previous paper.^38^ This allowed for the reduction of the number of animals used in the *in vivo* processes and the discovery of compounds with the best antiseizure properties.

All final compounds were obtained in good yields (>85%). Their structures were confirmed by ^1^H NMR and ^13^C NMR spectra analyses. Moreover, for all intermediates and the final compounds, LC-MS spectra were also obtained. Their purity determined by the UPLC method was >98%. For compounds with the most potent antiseizure properties, namely, *R*-enantiomers (**(*R*)-31–(*R*)-33**), high-resolution mass spectrometry (HRMS) analysis was carried out, as well. According to chiral HPLC, the enantiomeric excess (% ee) was greater than 99%. The chiral HPLC resolution was also carried out for each of the corresponding racemates in order to verify the enantiomeric purity. The Materials and Methods section contains a summary of the physicochemical and spectral data for the final compounds as well as the intermediates.

### Antiseizure Activity

Owing to the intricate pathophysiology of epilepsy, which is actually a diverse group of syndromes or diseases marked by an increased susceptibility for specific kinds of seizures, the most effective ASMs have been identified through phenotypic methods, primarily depending on *in vivo* models, and frequently, their molecular targets have been identified after approval. Nonetheless, we still do not know the precise mechanisms of several ASMs and many candidate compounds coming from target-based drug discovery efforts either failed in clinical development or were not successful commercially due to limited therapeutic utility. Consequently, the development of new ASMs is still based on well-established *in vivo* seizure models.^42,43^ So far, there are numerous different antiseizure tests/models described in the literature, but only a few of them constitute the so-called “the gold standard” utilized in the search for new and effective ASMs. Leading research institutes and programs (*i.e.*, Epilepsy Therapy Screening Program of the National Institute of Neurological Disorders and Stroke, NIH, Bethesda, MD, USA) use widely accepted panel of seizure models, including the MES test for identifying efficacy against generalized tonic-clonic seizures in humans, the 6 Hz test (32 mA, and its more drug resistant 44 mA version) which corresponds to human focal epilepsy, the *sc*PTZ test which relates to human generalized absence or myoclonic seizures.^44−46^ Therefore, during initial antiseizure studies, we tested all final compounds (**3**–**12**, **21–24,** (***S*)-31–(*S*)-33,** and **(*R*)-31–(*R*)-33)** in the MES and 6 Hz (32 mA) tests after intraperitoneal (*i.p*.) administration at a screening dose of 100 mg/kg in mice at time point of 0.5 h (the screening group consisted of four mice and the results obtained are presented in Table S1).

In the MES test, complete seizure protection (100%) was demonstrated only by *R*-enantiomers **(*R*)-31–(*R*)-33** from series C. *S*-enantiomers **(*S*)-31–(*S*)-33** showed weak (25%) protection. Unfortunately, **3**–**12** (series A) and **21**–**24** (series B) were inactive in this animal seizure model. In the 6 Hz (32 mA) test, we observed distinctly more potent protection for compounds tested. Similarly, in this model, *R*-enantiomers **(*R*)-31–(*R*)-33** showed complete protection, whereas *S*-enantiomers were also active and protected 75% of mice-(***S*)-32**, **(*S*)-33** or 50% of animals-**(*S*)-31**. Compounds representing series A and B showed partial activity in this seizure model, namely, 50% protection was demonstrated for **3**, **5**, **10**–**12**, **22,** and **23**, whereas weak 25% protection was observed for **6** and **24**. Other compounds were ineffective.

*R*-enantiomers **(*R*)-31–(*R*)-33** due to broad and potent activity in both MES and 6 Hz (32 mA) were further evaluated in the *sc*PTZ seizures. In this model, only **(*R*)-31** showed weak 25% protection, whereas **(*R*)-32** and **(*R*)-33** were ineffective. These results suggest that degradation of pyrrolidine-2,5-dione, which is the core fragment for compounds described in our previous paper^36^ (i.e., **KA-104**, Figure 1) to acetyl moiety causes almost complete loss of protection against PTZ seizures.

Based on screening results, it may be concluded that replacing the acetyl fragment in chemical prototypes represented by compound **KJ-5** (Figure 1) with aromatic acid residues (series A), as well as the bioisosteric replacement of piperazine to pyrrolidin-3-amine moiety (series B), made compounds inactive. Importantly, these preliminary *in vivo* data proved more beneficial antiseizure protection for *R*-enantiomers (eutomers) compared to *S*-enantiomers (distomers) in both MES and 6 Hz (32 mA) seizure models (series C), which was in line with our previous studies in series of pyrrolidine-2,5-dione derivatives.^34^

In the next step of the pharmacological characterization, the median effective doses (ED_50_) were determined for compounds showing minimum 75% protection at the dose of 100 mg/kg in both MES and 6 Hz (32 mA) tests. Moreover, the ED_50_ values in 6 Hz (44 mA) were also estimated for selected and potent *R*-enantiomers. Furthermore, as part of safety characterization, the median toxic doses (TD_50_) were determined in the chimney test to assess the influence of these compounds on motor coordination (studies performed 0.5 h after *i.p*. administration). Both the aforementioned parameters (ED_50_ and TD_50_) enabled the calculation of protective indexes (PIs), which reflect the comparative the benefit–risk ratio of these experimental therapeutic agents. Table 1 summarizes the obtained results along with previously published data for standard ASMs with established clinical utility, such as valproic acid (VPA), which is widely recognized as a broad-spectrum ASM (effective in the MES, 6 Hz [32/44 mA]); lacosamide (LCS, active in the MES and 6 Hz [32/44 mA] tests); and levetiracetam (LEV, effective in the 6 Hz test [32 mA]). Moreover, Table 1 includes preclinical data for CBD, which showed protection in MES and 6 Hz [32/44 mA] tests, as an example of a multitarget drug, which mechanism of action is partially based on desensitization of TRPV1 and inhibition of sodium currents.^30^

**Table 1:** ED_50_, TD_50_, and PI Values for Selected Phenylglycine Derivatives and Model ASMs (Mice, *i.p*.)^a^

Cmpd	PT (h)^d^	ED_50_ MES (mg/kg)^g^	ED_50_ 6 Hz (32 mA) (mg/kg)	ED_50_ 6 Hz (44 mA) (mg/kg)	TD_50_ (mg/kg)	PI(TD_50_/ED_50_)
**(*R*)-31**	**0.5**	**98.4**	**34.5**	**44.2**	**174.3**	**1.8 (MES)**
		**(82.8–116.8)**	**(28.2–42.2)**	**(36.6–53.4)**	**(153.4–198.1)**^b^	**5.1 (6 Hz, 32 mA)**
						**3.9 (6 Hz, 44 mA)**
**(*R*)-32**	**0.5**	**73.9**	**18.8**	**26.5**	**113.5**^b^	**1.5 (MES)**
		**(62.4–87.6)**	**(12.0–29.5)**	**(16.0–43.9)**	**(90.2–143.0)**	**6.0 (6 Hz, 32 mA)**
						**4.3 (6 Hz, 44 mA)**
**(*R*)-33**	**0.5**	**75.7**	**23.9**	**NT**	**146.3**	**1.9 (MES)**
		**(67.3–85.2)**	**(15.7–36.3)**		**(123.5–173.4)**^b^	**6.1 (6 Hz, 32 mA)**
**KJ-5**^e^	0.5	89.7	29.9	68.0	179.7^b^	2.0 (MES)
		(71.4–112.8)	(20.1–44.4)	(57.2–80.9)	(161.0–200.5)	6.0 (6 Hz, 32 mA)
						2.6 (6 Hz, 44 mA)
**KJ-28**^e^	0.5	73.6	24.6	56.3	166.8^b^	2.3 (MES)
		(63.6–85.2)	(12.2–49.5)	(46.8–67.7)	(109.6–253.8)	6.8 (6 Hz, 32 mA)
						2.8 (6 Hz, 44 mA)
**KJ-37**^e^	0.5	76.1	33.2	NT	156.2^b^	2.1 (MES)
		(61.5–94.3)	(21.2–52.0)		(137.7–177.1)	4.7 (6 Hz, 32 mA)
**LEV**^f^	1.0	>500	15.7	204.0	>500^c^	>31.8 (6 Hz, 32 mA)
			(11.2–18.4)	(154.5–269.5)		>2.5 (6 Hz, 44 mA)
**LCS**^f^	0.5	9.2	5.3	6.9	46.2^c^	5.0 (MES)
		(8.5–10.0)	(3.5–7.8)	(5.4–8.6)	(44.5–48.0)	8.8 (6 Hz, 32 mA)
						6.7 (6 Hz, 44 mA)
**VPA**^f^	0.5	252.7	130.6	183.1	430.7^c^	1.7 (MES)
		(220.1–290.2)	(117.6–145.2)	(143.5–233.7)	(407.9–454.9)	3.3 (6 Hz, 32 mA)
						2.3 (6 Hz, 44 mA)
**CBD**^f^	1.0	80.0	144.0	173.0	272.0^c^	3.4 (MES)
		(65.5–96.0)	(102.0–194.0)	(136.0–213.0)	(241.0–303.0)	1.9 (6 Hz, 32 mA)
						1.6 (6 Hz, 44 mA)

a **Data for the most potent compounds** (***R*)-31–(*R*)-33 have been given in bold for better visualization.** Results are represented as mean ± SD at 95% confidence limit determined by probit analysis. ED_50_, median effective dose.

b TD_50_, median toxic dose determined in the chimney test.

c TD_50_, median toxic dose determined in the rotarod test.

d Pretreatment time.

e Data for racemates: **KJ-5**, **KJ-28**, and **KJ-37** described as compounds **53**, **60**, and **62** in ref (38).

f Reference ASMs: CBD, LEV, LCS, and VPA tested under the same conditions, data taken from literature or own experiments.^31,36^

g No mortality was observed in the MES model for **(*R*)-31**–**(*R*)-33**. NT-not tested.

The outcomes demonstrated that in three acute animal models of seizures–MES, 6 Hz (32 mA), and 6 Hz (44 mA), all eutomers protected mice against seizures in an effective manner. **(*R*)-31** and **(*R*)-32** showed a broad spectrum of antiseizure activity similar to the respective **KJ-5** and **KJ-28**. The most potent protection across all seizure models was observed for **(*R*)-32**, which was identified as a lead molecule in this focused library of compounds. Importantly, **(*R*)-32** and **(*R*)-33** showed more potent activity in all models as well as similar PIs compared to the respective racemates—**KJ-28** and **KJ-37** identified previously.^38^ Finally, it should be strongly underpinned that both **(*R*)-31** and **(*R*)-32** showed distinctly better protection (1.5-fold and 2-fold, respectively) in the 6 Hz (44 mA) model of pharmacoresistant seizures *vs* respective racemates—**KJ-5** and **KJ-28**.

The antiseizure efficacy data obtained with reference ASMs (*i.e.*, VPA, LCS, LEV, and CBD) enabled clear differentiation and comparison of the efficacy/safety profile for compounds tested in the present study. Consequently, all eutomers were more effective in basic animal seizure models, i.e., MES, 6 Hz (32/44 mA), and possessed more beneficial PI than VPA. In comparison to LEV, which acts on SV2A protein located in presynaptic vesicle membranes, **(*R*)-32** showed similar activity in the 6 Hz (32 mA) test and potent activity in the MES test, whereas LEV was inactive in this test. It is noteworthy that (***R*)-32** showed almost 8-fold better potency *vs* LEV in the 6 Hz (44 mA) model of DRE. Compared to CBD with similar multitarget pharmacodynamics as **(*R*)-32**, the latter molecule showed equal activity in the MES test and distinctly more potent protection in the 6 Hz (32 mA) as well as the 6 Hz (44 mA) seizure model. Nevertheless, **(*R*)-32** exhibited lower potency in all three tests and safety margin compared to LCS, which is known to increase the slow inactivation of sodium channels.

Taking into consideration promising antiseizure activity in the MES and 6 Hz models, **(*R*)-32** and other eutomers **(*R*)-31** and **(*R*)-33** were evaluated in a panel of additional *in vivo* and *in vitro* studies focused on their efficacy, safety, and pharmacokinetic properties. Notably, the scope of these assays was the greatest for **(*R*)-32,** which appeared to be most interesting based on preliminary antiseizure data.

Furthermore, the results of thermogravimetric analyses (Figure S1) showed that this compound was thermally stable, while heating up to 190 °C when its weight loss was noted, indicating starting degradation. Bearing in mind that the active pharmaceutical ingredients are often exposed to heat upon a final dosage form manufacturing (*e.g.*, granulation, hot melt-extrusion, and coating), high thermal stability of the compound is a favorable feature predisposing the molecule **(*R*)-32** for a further development. Another property that may considerably slow down the process of bringing a new molecule to the market is limited solubility in water. Thus, a simple approach to verify if it would be possible to overcome this drawback by the physical modification of its crystalline structure was also undertaken. Figure S2 shows differential scanning calorimetry (DSC) heat flow curves recorded for crude compound (1st DSC heating scan) and its quenched liquid (2nd DSC heating scan). The melting endotherm of crystalline **(*R*)-32** had the onset ca. 115 °C and the minimum at 126 °C. After a simple thermal treatment such as cooling of the melt, the amorphous form of **(*R*)-32** was obtained. Its glass transition temperature was relatively low, ca. 30 °C, which means that **(*R*)-32** should be coprocessed with excipients of antiplasticizing properties to develop enabling formulations stable at ambient storage conditions.

### Effect on the Seizure Threshold and Neuromuscular Strength in Mice

**Figure 2:**
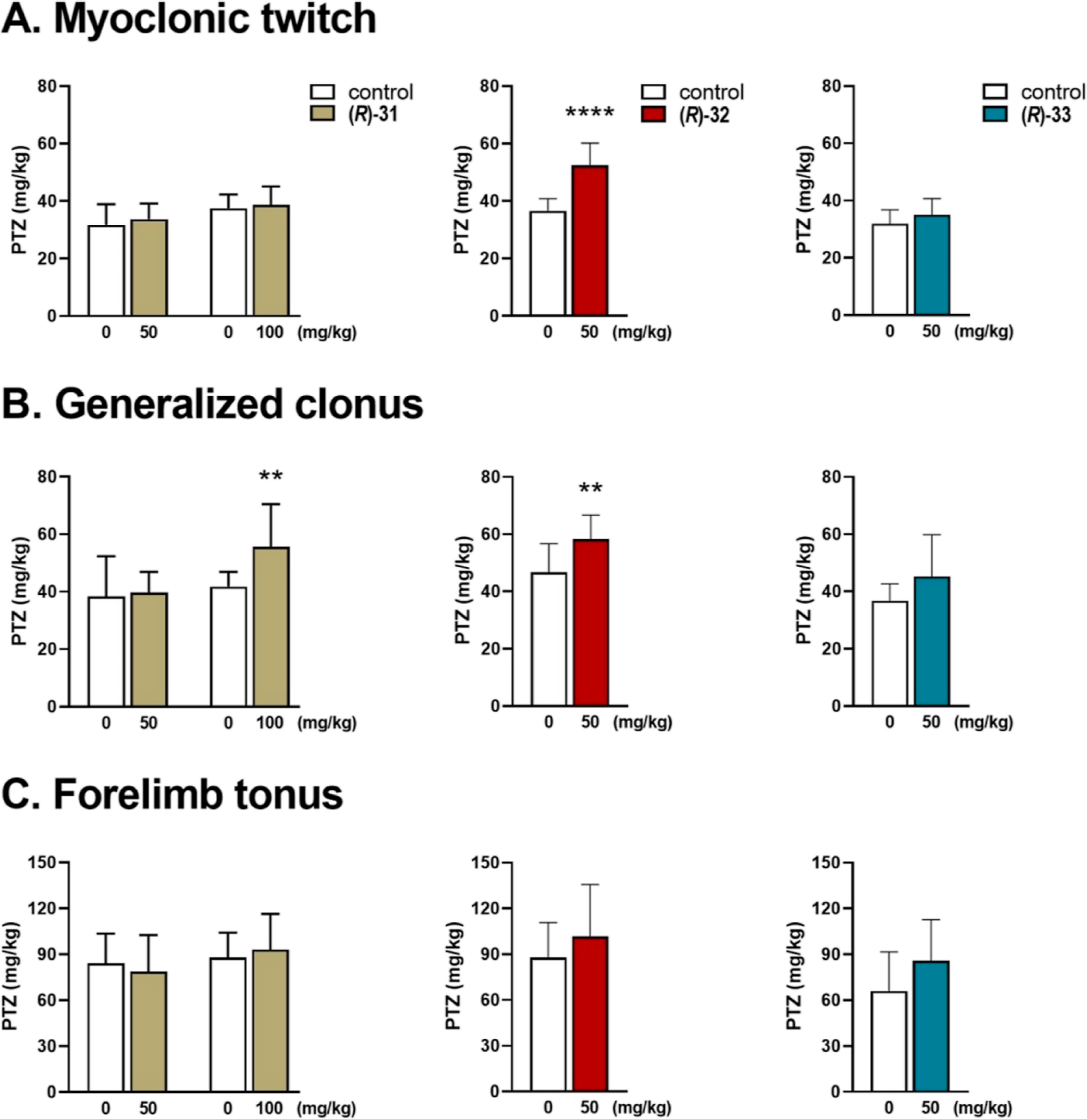
Effect of eutomers **(*R*)-31**, **(*R*)-32**, and **(*R*)-33** on the thresholds for the onset of (A) myoclonic twitch, (B) generalized clonus, and (C) forelimb tonic extension in the timed *iv*PTZ test in mice. **(*R*)-31**, **(*R*)-32**, and **(*R*)-33** were administered, *i.p.*, 30 min before the seizure test. Control animals received vehicle. Data are presented as means + SD in mg/kg of PTZ necessary to induce each of the three end points (*n* = 7–14 animals). Statistical significance was evaluated using Student’s *t*-test: **p < 0.01, ****p < 0.0001 *vs* the vehicle-treated group (GraphPad Prism 8.4.3).

The effects of **(*R*)-31**, **(*R*)-32**, and **(*R*)-33** on the thresholds for the first myoclonic twitch, generalized clonus with loss of righting reflex, and forelimb tonus in the timed *iv*PTZ seizure test in mice were evaluated in the following step of pharmacological characterization. The results are displayed in Figure 2A–C. It should be noted that the *iv*PTZ seizure test is an extremely sensitive method for determining the seizure threshold in rodents.^47^ In this model, **(*R*)-31** at a dose of 50 mg/kg had no effect on the threshold for the onset of any of the studied end points. However, at a higher dose tested (100 mg/kg), it significantly raised the threshold for generalized clonic seizure (*p* < 0.01). Compounds **(*R*)-32** and **(*R*)-33** were tested at a dose of 50 mg/kg only. **(*R*)-32** displayed higher activity in comparison to **(*R*)-31,** as it significantly increased the thresholds for the first myoclonic twitch and generalized clonus (*p* < 0.0001 and *p* < 0.01, respectively). However, **(*R*)-32** did not affect the threshold for the onset of forelimb tonus. **(*R*)-33** was ineffective in the *iv*PTZ seizure threshold test. This effect is consistent with results obtained in acute seizure models, indicating the most potent antiseizure activity **(*R*)-32**.

Prior to the *iv*PTZ test, a grip strength test was conducted to evaluate the tested eutomers’ acute impact on neuromuscular strength. Eutomers **(*R*)-31** (50 and 100 mg/kg), **(*R*)-32** (50 mg/kg), and **(*R*)-33** (50 mg/kg) did not produce any significant effects on neuromuscular strength, as assessed in the grip strength test (Figure S3).

### PTZ-Induced Kindling Model in Mice

**Figure 3:**
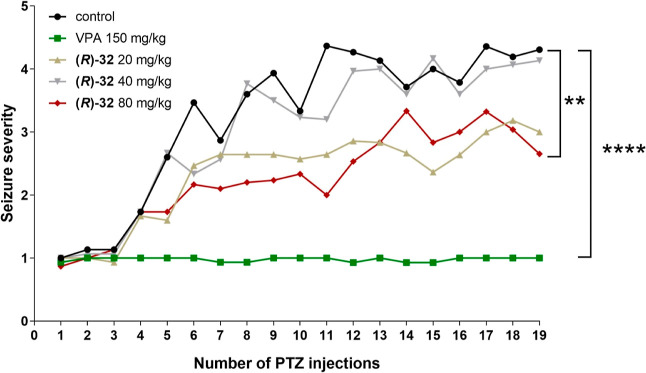
Effect of repeated treatment with **(*R*)-32** in PTZ-induced kindling model in mice. **(*R*)-32**, VPA, or vehicle were administered *i.p*. every 24 h. PTZ at a subconvulsive dose of 40 mg/kg was given *i.p*. three times a week, 30 min after administration of compound **(*R*)-32**, VPA, or vehicle. Data are presented as means of seizure severity (*n* = 10–15 animals). Statistical significance was evaluated by a mixed effects model for repeated measures followed by Tukey’s post hoc test: **p < 0.01, ****p < 0.001 *vs* control group (GraphPad Prism 8.4.3).

We investigated the impact of lead compound **(*R*)-32** on PTZ-induced kindling in mice to further assess its antiseizure potential (Figure 3). This compound showed the most potent antiseizure activity (in MES, 6 Hz [32/44 mA] and *iv*PTZ tests). The PTZ-induced kindling model is widely recognized as a useful method to induce chronic increase in susceptibility to epileptic seizures. In the PTZ control group, the mean seizure severity score (± SD) increased from 1.00 ± 0.00 to 4.31 ± 1.38 after the first and the last PTZ injection, respectively. VPA (150 mg/kg), a positive control, suppressed kindling progression, whereas **(*R*)-32** administered repeatedly at doses of 20 and 40 mg/kg did not significantly affect kindling development. However, **(*R*)-32** at the highest dose tested (80 mg/kg) slightly suppressed kindling progression, which was demonstrated by a significant reduction in the mean seizure severity score as compared to the PTZ control group (*p* < 0.01 after 19th PTZ injection). The average seizure severity score in the group treated with **(*R*)-32** at 80 mg/kg was 0.87 ± 0.35 after the first PTZ injection and 2.65 ± 1.28 after the last PTZ injection.

24 h after kindling completion, animals were subjected to the spontaneous locomotor activity test, the elevated plus maze test, and the forced swim test (FST). There were no discernible alterations in locomotor activity, and there were no reports of anxiety or depression-like symptoms (Figure S4).

### Spontaneous Electrographic Bursting in an *In Vitro* Model of Pharmacoresistant Seizure-like Activity

**Figure 4:**
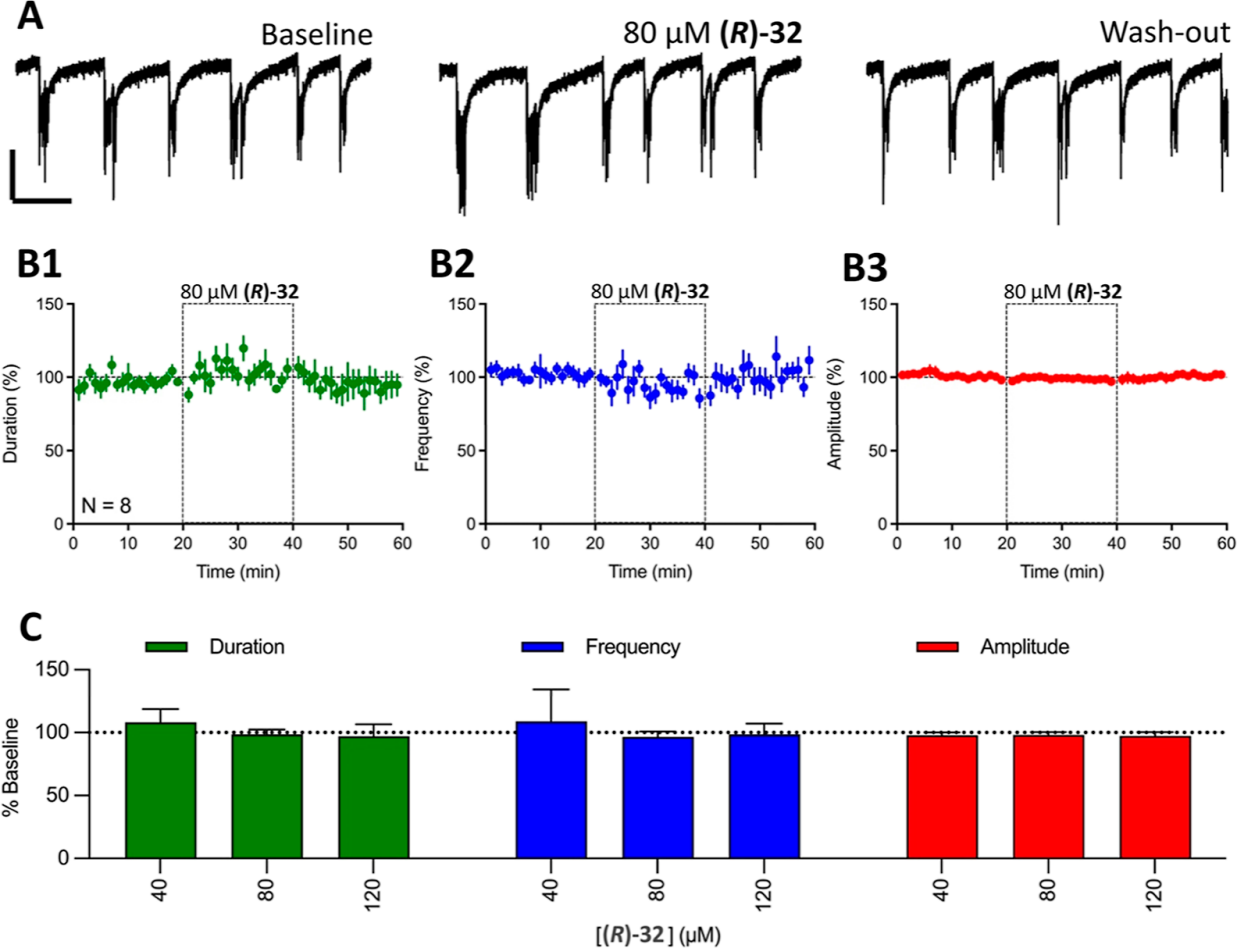
**(*R*)-32** fails to affect REDs (recurrent epileptiform discharges) at any concentrations tested. (A) Representative traces of REDs recorded before (baseline), in the presence of 80 μM **(*R*)-32**, and 20 min after washout. (B) Time courses for 80 μM **(*R*)-32**’s effects of REDs duration (B1, green), frequency (B2, blue), and amplitude (B3, red). Dashed-line boxes from 20 to 40 min represent the duration of 80 μM **(*R*)-32** exposure. Data points represent mean ± SD for REDs parameters in 1 min bins. (C) Concentration-dependent effects of **(*R*)-32** on REDs duration (green), frequency (blue), and amplitude (red). Bars represent mean + SD of 13 slices (40 μM), 8 slices (80 μM), and 10 slices (120 μM).

The effects of **(*R*)-32** on spontaneous recurrent epileptiform discharges (REDs) recorded in the medial entorhinal cortex of a brain slices obtained from rats that had previously experienced kainate-induced status epilepticus (KA-rats) were evaluated at 40, 80, and 120 μM. A 20 min bath exposure to **(*R*)-32** failed to significantly affect any quantified RED parameters (duration, frequency, and amplitude) at any of the tested concentrations. Representative traces of REDs before, during, and after exposure to 80 μM **(*R*)-32** are illustrated in Figure 4; the characteristics of the REDs in the presence of **(*R*)-32** were notably similar to those observed during baseline. Time course data confirmed these observations by demonstrating no significant deviations from baseline RED duration, frequency, or amplitude (Figure 4B1–B3, respectively). Finally, the average duration, frequency, and amplitude of REDs after a 20 min exposure, and normalized to baseline measurements, is illustrated in Figure 4C for 40, 80, and 120 μM **(*R*)-32**. No significant effects were observed for any of these parameters at any concentrations tested. Accordingly, no EC_50_ quantifications were performed.

### Effect on Glutamate and GABA Concentrations in the Hippocampus and Cortex of PTZ-Kindled Mice

**Figure 5:**
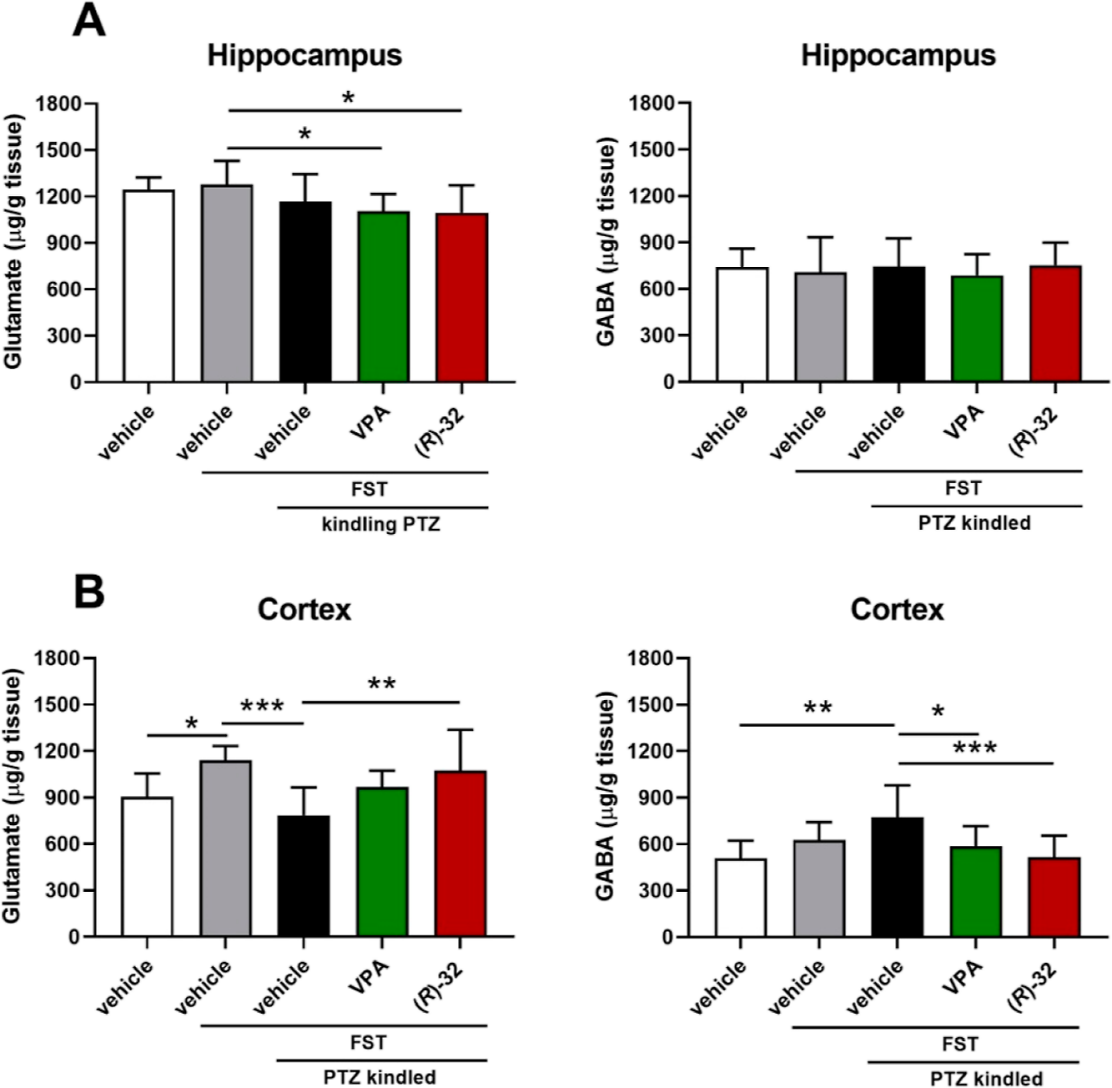
Changes in glutamate and GABA concentrations in the hippocampus (A) and cortex (B). Data are shown as means + SD (*n* = 9–14 animals). Statistical significance was evaluated by one-way ANOVA followed by Tukey’s post hoc test: *p < 0.05, **p < 0.01, ***p < 0.001 (GraphPad Prism 8.4.3).

After behavioral tests (FST, PTZ-induced kindling), we determined the changes in glutamate and GABA concentrations in the hippocampus and cortex. Since exposure to the FST can evoke various neurochemical alterations, two additional groups were employed in this experiment, *i.e.*, control nonkindled mice that were exposed to the FST and naïve mice. No marked differences in both glutamate and GABA concentrations in the hippocampus were observed (Figure 5A). There was only a slight decrease of glutamate concentrations in the VPA- and **(*R*)-32**-treated groups as compared to the nonkindled FST control group. We found a significant increase in glutamate concentration in the cortex of mice subjected to the FST (*p* < 0.05 *vs* naive mice). PTZ-induced kindling decreased glutamate concentration (by ∼30%) in comparison to the nonkindled control group exposed to the FST (*p* < 0.001), whereas **(*R*)-32** (80 mg/kg) reversed this effect (*p* < 0.01 *vs* the PTZ-kindled control group; Figure 5B). Moreover, **(*R*)-32** (80 mg/kg) and VPA (150 mg/kg) significantly decreased GABA concentration in the cortex (*p* < 0.001 and *p* < 0.05 *vs* the PTZ-kindled control group; Figure 5B). Given that seizures are typically accompanied by elevated extracellular glutamate levels (an excitatory neurotransmitter), a decreased glutamate concentration in the cortex of kindled animals may appear to be an unexpected outcome. It should be highlighted, however, that the concentrations of glutamate and GABA in tissue homogenates rather than dialysate were measured in brain samples acquired 24 h following the previous PTZ dose, which may have an impact on the outcomes. On the other hand, decreased glutamate levels could be caused by the up-regulation of glutamate transporters, as the expression of glutamate transporters can increase following seizures.^48^ For example, an increased expression of hippocampal GLT-1 was observed 24 h after kainic acid-induced status epilepticus in mice and PTZ-induced kindling in rats.^49^ Moreover, similar to our findings, Szyndler *et al.*(50) found decreased glutamate concentrations, with no changes in GABA concentrations, in homogenates of the hippocampus, striatum, and prefrontal cortex of PTZ-kindled rats. Changes in glutamate and GABA concentrations in kindled animals treated with **(*R*)-32** suggest its potential effect on glutamate and GABA synthesis/metabolism, but this needs further careful evaluation.

### BDNF and NGF Expression in the Hippocampus and Cortex of PTZ-Kindled Mice

**Figure 6:**
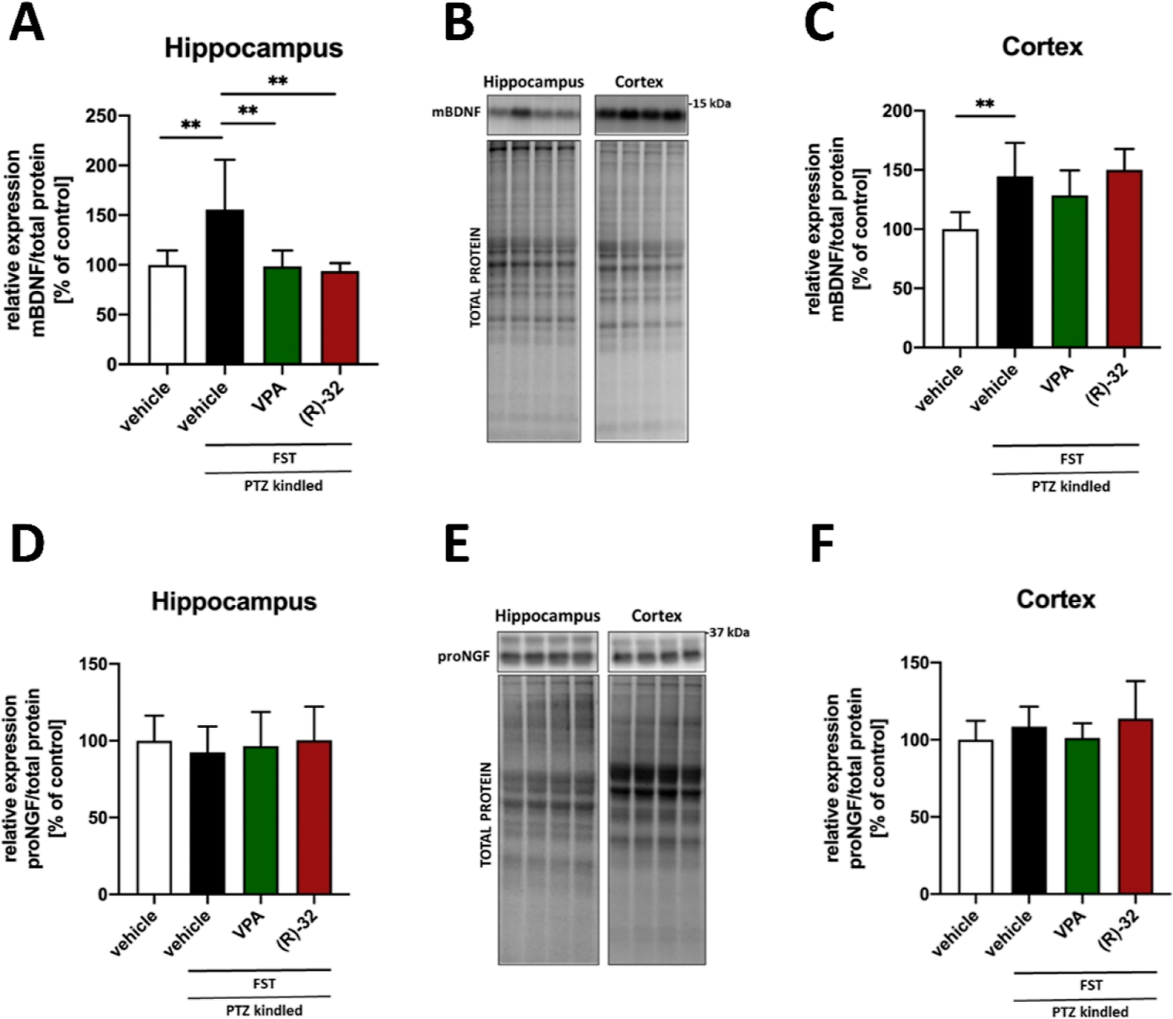
Changes in mature-BDNF (mBDNF) expression in hippocampus (A) and cortex (C) and in proNGF expression in hippocampus (D) and cortex (F) with representative immunoblots, together with total protein amount visualized by the stain-free technique (B,E). Data are shown as means of relative expressions + SD (*n* = 8). Statistical significance was evaluated by one-way ANOVA followed by Tukey’s post hoc test: **p < 0.01 (GraphPad Prism 8.4.3).

Studies show that brain injury and seizures per se can induce the overexpression of the brain-derived neurotrophic factor (BDNF), particularly in the hippocampus. This suggests that the overexpression of neurotrophins caused by the initial insult may contribute to the development and maintenance of neuronal hyperexcitability in hippocampal networks, thereby promoting epileptogenesis. However, there are also few reports showing opposite findings.^51,52^ In our study, PTZ-induced kindling caused a significant increase of mature-BDNF (mBDNF) protein expression both in the hippocampus and in the cortex (*p* < 0.01), while repeated administration of VPA and **(*R*)-32** at 80 mg/kg reversed the overexpression of mBDNF in the hippocampus (*p* < 0.01 *vs* the PTZ kindling control group), but not in the cortex of kindled mice (Figure 6A–C). No changes in the expression of the nerve growth factor (NGF) were observed (Figure 6D–F). Thus, the suppression of kindling development by VPA and **(*R*)-32** at 80 mg/kg could be related, at least in part, to the downregulation of mBDNF in the hippocampus. It is also worth noticing that **(*R*)-32** decreased the expression of mBDNF at a level comparable to VPA, though it was less effective against the PTZ kindling-induced seizures than VPA.

### Effect on the Capsaicin-Induced Hypothermia in Mice

Both in preclinical animal investigations and in human clinical trials, a number of TRPV1 antagonists, particularly the first-generation TRPV1 antagonists with polymodal mechanisms of action, have been shown to cause hyperthermia.^53−55^ In this study, we used the capsaicin-induced hypothermia model in mice to evaluate the effect of eutomers **(*R*)-31**, **(*R*)-32**, and **(*R*)-33** on body temperature and to provide more data related to their on-target activity (*i.e.*, antagonism of TRPV1) (Figure S5A–D). Capsaicin given *i.p*. at a dose of 5 mg/kg produced a marked decrease in rectal temperature at 15 min post injection (*p* < 0.0001). The temperature returned to normal values within 30–60 min after capsaicin injection. BCTC—TRPV1 antagonist (positive control) administered at a dose of 20 mg/kg caused a significant increase in body temperature at 15 min after injection (*p* < 0.05), while coadministration of BCTC with capsaicin alleviated the capsaicin-induced hypothermia (*p* < 0.0001 *vs* the capsaicin-treated group at 15 and 30 min post capsaicin injection). Compounds **(*R*)-31** and **(*R*)-32** administered alone (50 mg/kg) did not significantly affect body temperature. However, **(*R*)-33** given alone (50 mg/kg) caused a significant drop in rectal temperature at 15 min post administration (*p* < 0.001). None of the tested compounds reversed the capsaicin-induced hypothermia.

The TRPV1 channel is activated by capsaicin, protons, or heat, and it was suggested that only one activation mode, *i.e.*, by protons, plays a role in the influence of TRPV1 antagonists on body temperature. The first-generation TRPV1 antagonists work by blocking all three TRPV1 activation modes and they often cause hyperthermia. Second-generation TRPV1 antagonists are mode-specific, and compounds that block, potentiate, or have no effect on TRPV1 activation by protons can induce hyperthermia and hypothermia or have no influence on body temperature, respectively.^53−55^ Since **(*R*)-33** significantly decreased body temperature, it can be speculated that this compound may potentiate the proton-induced activation of TRPV1. However, in *in vitro* functional studies **(*R*)-33** showed similar TRPV1 antagonist properties in comparison to other compounds tested [**(*R*)-31** and **(*R*)-32**]. However, caution must be taken, as **(*R*)-33** was tested after a single administration at one dose and hypothermia was observed only 15 min after administration. Moreover, we measured body temperature using a rectal probe, not a telemetry system, which would provide a more accurate assessment.

### Antinociceptive Activity

To evaluate the analgesic properties of **(*R*)-31** (the most potent TRPV1 antagonist; see the *in vitro* studies section) and **(*R*)-32** (lead compound characterized by the most effective antiseizure properties), we used well-established *in vivo* pain models. One of the most widely used experimental models for studying pain and analgesia is the formalin test. The formalin test is a highly valuable tool in preclinical research for the development of new pain relief medications. Its primary strength lies in the precise quantification of pain-related behaviors in response to noxious stimuli, such as formalin. Additionally, the formalin test enables the examination of two separate types of pain sensations, which are observed as two distinct phases of the test. The first phase of the test reflects acute peripheral pain and is triggered by the direct activation of nociceptors through TRPA1 channels. The late phase of the test results from the release of inflammatory mediators (prostaglandins, bradykinin, and cytokines released from the damaged tissues and activated immune cells) and central nociceptive sensitization.^56,57^

**Figure 7:**
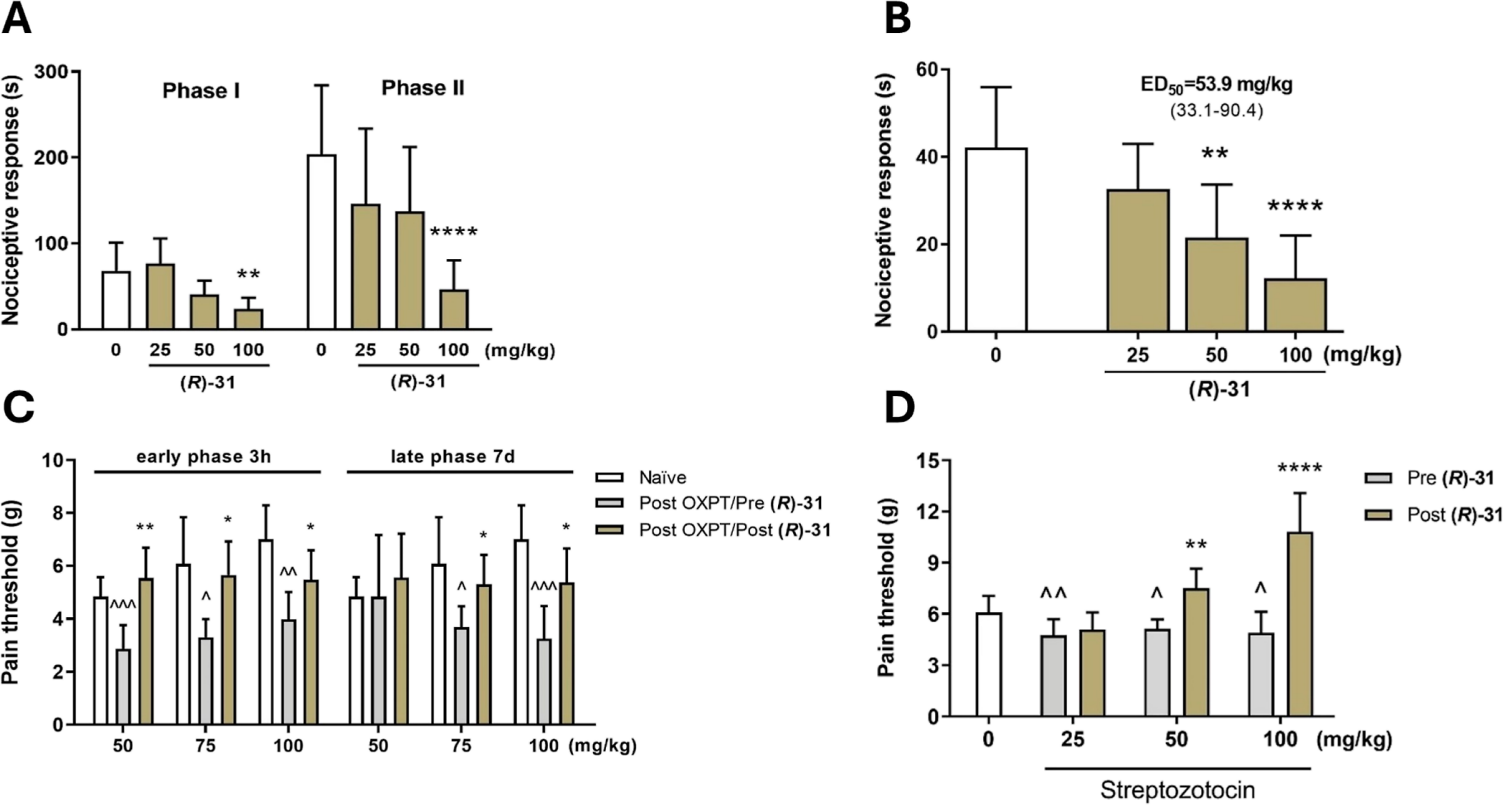
(A) Effect of compound **(*R*)-31** on the duration of licking/biting behavior in the acute phase (0–5 min after formalin injection and in the late phase and 15–30 min after formalin injection). The test compound or vehicle (1% Tween 80) was administered 30 min *i.p*. before the test. (B) Effect of compound **(*R*)-31** on the duration of the nociceptive response in capsaicin-induced pain. The test compound or vehicle (1% Tween 80) was administered 30 min (*i.p*.) before the capsaicin injection. The results are presented as bar plots showing the mean ± SEM. (C) Antiallodynic effects of compound **(*R*)-31** in the tactile allodynia in oxaliplatin (OXPT)-induced peripheral neuropathy. The compound was administered at the doses of 50, 75, and 100 mg/kg 30 min before the evaluation in the von Frey test carried out 3 h and 7 days after OXPT injection. (D) Antiallodynic effects of compound **(*R*)-31** in the tactile allodynia in streptozotocin (STZ)-induced peripheral neuropathy. The compound was administered at the doses of 25, 50, and 100 mg/kg 30 min before the evaluation in the von Frey test carried out 21 days after STZ injection. The statistical significance (A, B) was evaluated by one-way ANOVA, followed by Dunnett’s post hoc test: **p < 0.01, ****p < 0.0001, *n* = 8–10 mice per group. The statistical significance (C, D) was evaluated by repeated measures analysis of variance (ANOVA), followed by Dunnett’s post hoc comparison: *p < 0.05, **p < 0.01, and ****p < 0.0001 when results compared to the OXPT-treated group (Post Oxali/Pre **(*R*)-31**) or STZ-treated group (Post **(*R*)-31**) and ^∧^*p* < 0.05, ^∧∧^*p* < 0.01, and ^∧∧∧^*p* < 0.001, when results compared to naive mice, *n* = 10 mice per group (GraphPad Prism 8).

**Figure 8:**
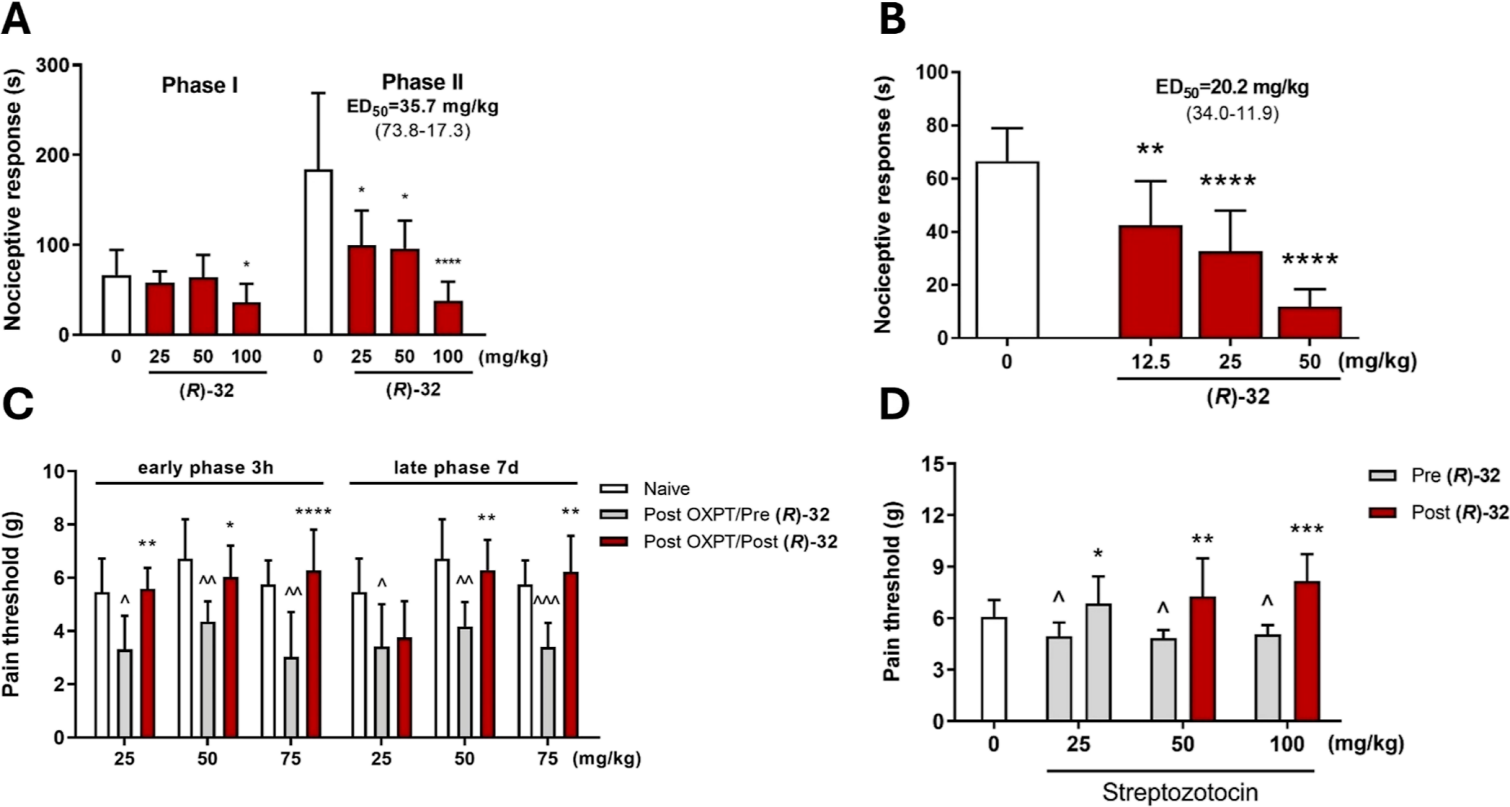
(A) Effect of compound **(*R*)-32** on the duration of licking/biting behavior in the acute phase (0–5 min after formalin injection and in the late phase and 15–30 min after formalin injection). The test compound or vehicle (1% Tween 80) was administered 30 min *i.p*. before the test. (B) Effect of compound **(*R*)-32** on the duration of the nociceptive response in capsaicin-induced pain. The test compound or vehicle (1% Tween 80) was administered 30 min (*i.p*.) before the capsaicin injection. The results are presented as bar plots showing the mean ± SEM. (C) Antiallodynic effects of compound **(*R*)-32** in the tactile allodynia in oxaliplatin (OXPT)-induced peripheral neuropathy. The compound was administered at the doses of 25, 50, and 75 mg/kg 30 min before the evaluation in the von Frey test carried out 3 h and 7 days after OXPT injection. (D) Antiallodynic effects of compound **(*R*)-32** in the tactile allodynia in streptozotocin (STZ)-induced peripheral neuropathy. The compound was administered at the doses of 25, 50, and 100 mg/kg 30 min before the evaluation in the von Frey test carried out 21 days after STZ injection. The statistical significance (A, B) was evaluated by one-way ANOVA followed by Dunnett’s post hoc test: *p < 0.05, **p < 0.01, ****p < 0.0001, *n* = 8–10 mice per group. The statistical significance (C, D) was evaluated by repeated measures analysis of variance (ANOVA), followed by Dunnett’s post hoc comparison: *p < 0.05, **p < 0.01, and ***p < 0.01, ****p < 0.0001 when results compared to the OXPT-treated group (Post Oxali/Pre **(*R*)-32**) or STZ-treated group (Post **(*R*)-32**) and ^∧^*p* < 0.05, ^∧∧^*p* < 0.01, and ^∧∧∧^*p* < 0.001 when results compared to naive mice, *n* = 10 mice per group (GraphPad Prism 8).

Administration of **(*R*)-31** before the formalin injection significantly attenuated the nociceptive response in mice in both phases of the test but only at the highest dose of 100 mg/kg (Figure 7A). **(*R*)-32** at the dose of 100 mg/kg decreased the nociceptive response in the acute phase, while in the second phase, the compound inhibited nociceptive reaction at all tested doses (25, 50, and 100 mg/kg), which shows the high potency of the compound in attenuating chronic inflammatory pain. Its ED_50_ value in the late phase was found to be 35.7 mg/kg (Figure 8A).

We used other models and methods to obtain a more comprehensive understanding of the analgesic profile of the test compounds. We decided to evaluate the influence of the compounds on TRPV1-dependent pain using the capsaicin test. Capsaicin induces acute pain by activation of TRPV1 receptors, which is different from formalin, which acts more broadly by activation of TRPA1 receptors as well as other targets.^58^ Both tested compounds significantly decreased the paw licking or biting behavior in the capsaicin test, revealing higher potency than in the formalin test (Figures 7B and 8B). The ED_50_ values in the capsaicin test were 53.9 and 20.2 mg/kg for **(*R*)-31** and **(*R*)-32**, respectively. Consequently, these results seem to indicate higher *in vivo* potency of both compounds in inhibiting TRPV1-dependent pain, which is in line the higher *in vitro* potency for TRPV1 antagonism *vs* TRPA1. Finally, **(*R*)-32** appeared to have generally the highest potency in pain models.

Given the therapeutic challenges in treatment of neuropathic pain, we evaluated the activity of the compounds in two different experimental models of this condition.^59^ Oxaliplatin (OXPT), used as the first model, has been shown to cause damage to sensory neurons and their axons, resulting in axonal degeneration and demyelination. This damage can lead to alterations in the expression and function of ion channels and receptors involved in nociceptive signaling, such as voltage-gated sodium channels, potassium channels, and TRPs.^60^ Moreover, neuroinflammation is now emerging to be relevant in the pathophysiology of OXPT-induced neuropathy.^61^ In experimental conditions, these mechanisms trigger mechanical allodynia, which can be observed as a decrease in the pain threshold, as assessed by the von Frey test.^37^

The administration of OXPT resulted in a statistically significant decrease of the pain threshold measured 3 h and 7 days after injection, which correspond to the early and late phase of neuropathic pain. The decreases of the pain threshold ranged between 46.4 and 60.6% from the baseline values (*i.e.*, before OXPT administration). We tested **(*R*)-31** at the doses of 50, 75, and 100 mg/kg (Figure 7C). The early phase effect of OXPT was considerably negated by a single dosage of the tested compound. Interestingly, the most potent effect was observed at the dose of 50 mg/kg, which completely reversed the effect of OXPT, reaching the pain threshold value of 114.5% of the initial value. The values for higher doses were 93 and 78% for 75 and 100 mg/kg, respectively. We observed similar effects in the late phase where the values for 75 and 100 mg/kg were 87 and 77%, respectively. Exceptionally, in the group treated with 50 mg/kg of **(*R*)-31** and in the respective control group, we did not observe the allodynic effect of the OXPT. These results may be interpreted as an inverted U-shaped dose response. This shows that the compound at a dose of 50 mg/kg reaches maximal efficacy, which decreases at higher doses. This kind of activity is well-known in pharmacology and highlights a potentially narrow therapeutic window, necessitating optimal dose selection.^62^ Since **(*R*)-32** showed higher potency than **(*R*)-31** in the formalin and capsaicin test, we tested that compound in OXPT-induced allodynia at the doses of 25, 50, and 75 mg/kg (Figure 8C). The single administration of the test compound significantly reversed the effect of OXPT in the early phase at all tested doses, reaching the pain threshold values of 102, 90, and 109% of the baseline for 25, 50, and 75 mg/kg, respectively. We did not observe the analgesic effects for the dose of 25 mg/kg in the late phase (68% of the baseline), but the activity of higher doses was statistically significant and resulted in the pain threshold values of 94 and 108% for 50 and 75 mg/kg, respectively.

To test whether the compounds are active in the neuropathic pain resulting from another mechanism of neuronal injury, we tested them in streptozotocin (STZ)-induced neuropathy. STZ is the commonly used experimental model to induce diabetic neuropathy. Streptozotocin is toxic to insulin-producing beta cells in the pancreas and selectively damages these cells, leading to insulin deficiency, which subsequently results in chronic hyperglycemia. Hyperglycemia is a major factor in the development of diabetic neuropathy. Elevated blood glucose levels increase its uptake by nerve cells, which results in metabolic and oxidative stress. Moreover, hyperglycemia leads to the formation of advanced glycation end products (AGEs), which are abnormal protein structures. AGEs can accumulate in neuronal tissue and contribute to nerve dysfunction.^63^ STZ has been shown to alter the expression and function of ion channels and receptors such as voltage-gated sodium channels, potassium channels, TRPs, and purinergic receptors. Additionally, STZ-induced neuropathic pain may also involve alterations in the levels of neurotransmitters such as glutamate and GABA.^64,65^ As nerve damage progresses, animals may exhibit sensory abnormalities and neuropathic pain-like behaviors, such as mechanical allodynia.^66^

A single *i.p*. administration of STZ at the dose of 200 mg/kg resulted in allodynia, observed as a significantly decreased pain threshold to the values ranging from 78 to 84% of the baseline (the value before STZ administration). The activity of **(*R*)-31** at the doses of 50 and 100 mg/kg significantly increased the pain threshold values to 123 and 178% of the baseline, respectively. We did not observe any significant effects at the dose of 25 mg/kg (Figure 7D). **(*R*)-32** showed significant activity in all tested doses. The observed increases in pain threshold values were 113, 120 and 134% for the doses of 25, 50, and 100 mg/kg, respectively (Figure 8D).

Head-to-head comparison of the activity of the two tested compounds in STZ-induced diabetic neuropathy showed that **(*R*)-31** had higher efficacy (maximal possible effect), but **(*R*)-32** had greater potency, which may allow for administration of lower doses with efficient antinociceptive activity. The obtained results revealed that both **(*R*)-31** and **(*R*)-32** are effective in attenuating neuropathic pain resulting from distinct mechanisms of nervous system damage.

Compared with VPA, which was previously tested in our laboratory,^33^ the test compounds showed higher analgesic activity. In the formalin test, VPA was active in the late phase of the test, revealing a potency that was significantly lower than that obtained for **(*R*)-31**, especially for **(*R*)-32**. The ED_50_ value for VPA was 132.9 mg/kg, whereas that for **(*R*)-32** was 35.7 mg/kg. This difference was even more pronounced for capsaicin-induced pain. VPA was active only at the highest administered dose of 200 mg/kg, while the ED_50_ for **(*R*)-31** and **(*R*)-32** was 53.9 and 20.2 mg/kg, respectively. The highest potency and efficacy of the investigated compounds in the capsaicin test may have resulted from the greater involvement of TRPV1 receptors in their overall analgesic activity.

### Pharmacokinetic Studies

Several pharmacokinetic parameters were determined by the use of the noncompartmental analysis (Table 2).

**Table 2:** Pharmacokinetic Parameters of **(*R*)-32** Estimated in Serum and Brain Following *i.p*. and *p.o*. Administration to Mice Using Non-compartmental Analysis

pharmacokineticparameter (unit)^a^	serum			brain		
dose (mg/kg)	25 ***i.p***.	50 ***i.p***.	25 ***p.o***.	25 ***i.p***.	50 ***i.p***.	25 ***p.o***.
*t*_max_ (min)	15	15	5	15	15	5
*C*_max_ (ng/mL(g))	7613.33	15,900.00	1266.00	8266.67	21,475.00	1870.33
λ_*z*_ (1/min)	0.009	0.011	0.005	0.009	0.011	0.005
*t*_1/2 λ___*z*__ (min)	77.51	64.27	127.16	80.52	62.32	132.52
AUC_0–∞_ (ng·min/mL(g))	228,063.48	464,877.54	118,998.11	274,802.60	689,733.29	156,252.29
V_*z*_/F (L/kg)	12.26	9.97	38.54			
CL/F (L/min/kg)	0.110	0.108	0.210			
MRT (min)	42.32	33.59	156.01	45.54	47.34	150.93

a Pharmacokinetic parameters: *t*_max_- time to reach *C*_max_; *C*_max_- maximum serum/brain concentration; λ_*z*_- terminal slope; *t*_1/2 λ_*z*__- terminal half-life; AUC_0–∞_- area under the curve; V_*z*_/F- volume of distribution; CL/F- clearance; MRT- mean residence time.

Concentration versus time profiles of **(*R*)-32** in the mouse serum and brain after *i.p*. administration of 25 and 50 mg/kg are presented in Figure S6, whereas Figure S7 shows the same parameters for **(*R*)-32** after oral (*p.o*.) administration.

Pharmacokinetic profile analysis revealed that **(*R*)-32** was very quickly absorbed after both *i.p*. and *p.o*. dosing as *t*_max_ was no longer than 15 min in both cases. Interestingly, the absorption was even faster after *p.o*. administration. The same pattern was observed in the brain tissue. The compound penetrated very fast to the brain tissue, reaching concentrations higher than in serum at almost all data points (Figures S6 and S7). Brain-to-serum AUC ratios exceeded 1, *i.e.,* 1.2 and 1.3 after *i.p*. and *p.o*. dose of 25 mg/kg, respectively, and 1.5 following 50 mg/kg. *C*_max_ and AUC (both parameters established after *i.p*. injection) increased dose-proportionally, indicating linear pharmacokinetics in the tested concentration range. Consequently, similar *t*_0.5_, V/F, and CL/F values were observed after both *i.p*. doses. These parameters were 2–3 times higher after the *p.o*. dosing, probably as the result of a lower value of the fraction of dose absorbed (F) from the gastrointestinal tract. The ratios of *C*_max_ and AUC in serum after *p.o*. dosing compared to the respective values of these parameters obtained after *i.p*. administration of the same dose were lower than 0.5, which may indicate a higher bioavailability of **(*R*)-32** after the *i.p*. dosing. The values of V/F were high and significantly exceeded mouse body water, indicating a robust distribution of **(*R*)-32** to murine organs and tissues. The mean residence time (MRT) was almost 4-fold higher after *p.o*. dosing in comparison to *i.p*. administration; thus, a longer pharmacological effect may be expected when the studied compound is administered orally.

### *In Vitro* Binding and Functional Assays

Due to the fact that the most active compounds reported herein are (***R***)-enantiomers of the previously obtained racemates, *i.e.,***KJ-5**,^38^ it is hypothesized that they have similar and multimodal mechanisms of action, including antagonism of TRPV1, sodium, and calcium channels. The current body of literature consistently reports that both sodium (Nav_*x*_) and Cav_1.2_ calcium ion channels can be involved in the pathogenesis of epilepsy^67−69^ and neuropathic pain.^70−73^ Consequently, these ion channels are recognized as well-known molecular targets for a majority of clinically used ASMs such as LCS, lamotrigine, carbamazepine, and oxcarbazepine.^74^ Therefore, we performed binding and functional studies toward sodium (site 2) and calcium Cav_1.2_ channels at concentrations of 10 μM for **(*R*)-31**−**(*R*)-33**. Notably this concentration can be reached in CNS as determined in the *in vivo* PK studies (see above). As it is shown in Table 3, despite strong structural similarities, these compounds revealed different affinities toward sodium channels, namely, a significant effect was observed for 3-SCF_3_ derivative **(*R*)-33**, whereas the 3-OCF_3_ analogue (**(*R*)-32**) and 3-CF_3_ congener (**(*R*)-31**) displayed a weaker interaction.

**Table 3:** *In Vitro* Binding and Functional Assays for Eutomers **(*R*)-31–(*R*)-33**

binding studies	source	% inhibition of control specific binding (concentration [μM])^a^		
		**(*R*)-31**	**(*R*)-32**	**(*R*)-33**
Na^+^ channel (site 2)	rat cerebralcortex	33.9 (10)	40.7 (10)	63.2 (10)

functional studies	source	% inhibition of control agonist response (concentration [μM])^a^		
		**(*R*)-31**	**(*R*)-32**	**(*R*)-33**
Cav_1.2_ (*h*) calcium ion channel cell-based antagonist calcium flux assay	human recombinant HEK-293 cell	75.0 (10)	31.0 (10)	49.0 (10)
TRPM8 (*h*) (antagonist effect)	human recombinant HEK-293 cell	4.6 (10)	12.6 (10)	NT
TRPA1 (*h*) transient potentialion channel cell based antagonist calcium flux assay	human recombinant HEK-293 cell	13.3 (10)	9.6 (10)	NT
TRPV1 (VR1) (*h*) (antagonist effect)	human recombinant CHO cells	IC_50_ = 3.5 μM, *K*_B_ = 0.46 μM	IC_50_ = 11 μM, *K*_B_ = 1.4 μM	IC_50_ = 13 μM, *K*_B_ = 1.6 μM

a Results showing activity higher than 50% are considered to represent significant effects of the test compounds; results showing an inhibition between 25 and 50% are indicative of moderate effect; results showing an inhibition lower than 25% are not considered significant and mostly attributable to the variability of the signal around the control level. IC_50_- concentration causing a half-maximal inhibition of the control agonist response, *K*_B_- dissociation constants, NT- not tested.

In functional assays, only the compound **(*R*)-31** showed a significant inhibition of calcium currents mediated by the Cav_1.2_ channel, whereas **(*R*)-32** and **(*R*)-33** revealed only a moderate effect. Collectively, these *in vitro* studies indicate that simultaneous, albeit moderate modulation of both sodium and calcium currents may produce a synergistic effect that could potentially contribute to their broad antiseizure and antinociceptive activity *in vivo*.

Further *in vitro* functional studies confirmed TRPV1 antagonist activity for **(*R*)-31–(*R*)-33**. In general, all compounds showed a similar and moderate blockade of TRPV1 (IC_50_ range 3.5–11 μM). Other compounds obtained in current studies (Table S2) did not show expected TRPV1 antagonism properties. Despite structural similarities to several TRPA1 and TRPM8 ligands,^22,75−79^ these compounds did not interact with these TRPs, and thus their analgesic properties are not likely related to TRPA1 or TRPM8. Binding assays performed herein for CBD at the concentration of 100 μM (Table S3) revealed significant interaction with sodium channels and Cav_1.2_ channel (dihydropyridine site), as well as CBD appeared to be potent agonist of CB1 receptor and moderate antagonist of TRPV1 channel.

**Figure 9:**
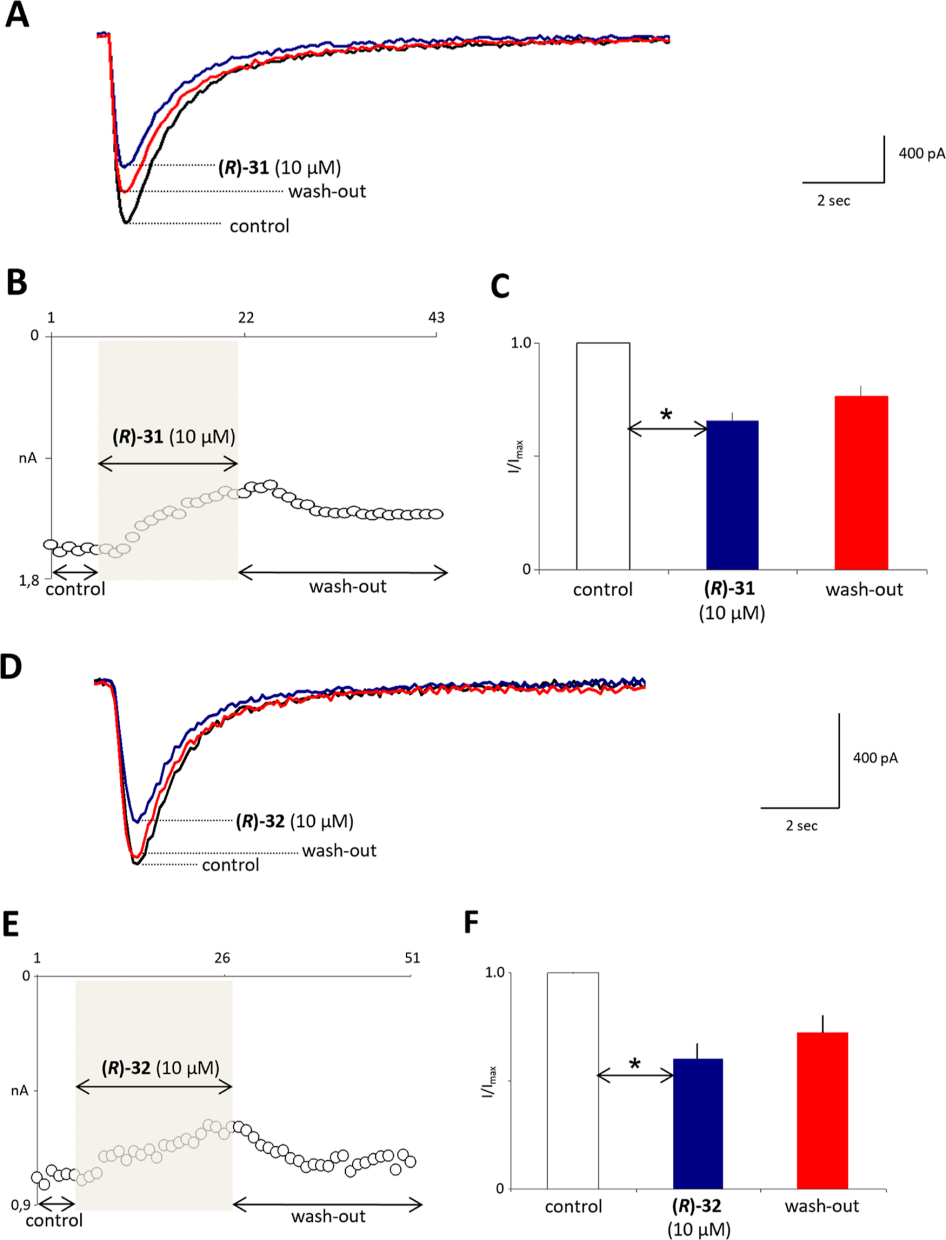
Compounds **(*R*)-31** and **(*R*)-32** inhibit fast voltage-gated sodium currents in prefrontal cortex pyramidal neurons. (A) Example sodium current recordings in control (black trace), after application of **(*R*)-31** (blue trace) and after wash-out (red trace). Current traces were evoked by a rectangular voltage-step. (B) Influence of **(*R*)-31** on sodium current is shown on an example neuron. Current traces were evoked once every 10 s. The vertical axis shows maximal current amplitudes (white circles) in control, in the presence of **(*R*)-31** and after wash-out. The horizontal axis shows trace number. (C) Averaged, normalized maximal sodium current amplitudes in control, in the presence of **(*R*)-31** and after wash-out. An asterisk shows statistical significance. (D) Example sodium current recordings in control (black trace), after application of **(*R*)-32** (blue trace) and after wash-out (red trace). Current traces were evoked by a rectangular voltage-step. (E) Influence of **(*R*)-32** on sodium current is shown on an example neuron. Current traces were evoked once every 10 s. The vertical axis shows maximal current amplitudes (white circles) in control, in the presence of **(*R*)-32** and after wash-out. The horizontal axis shows trace number. (F) Averaged, normalized maximal sodium current amplitudes in control, in the presence of **(*R*)-32** and after wash-out. An asterisk shows statistical significance.

During further mechanistic studies, the most effective antiseizure compounds, **(*R*)-31** and **(*R*)-32**, were also examined for their impact on fast voltage-gated sodium channels in rat prefrontal cortex pyramidal neurons using the patch-clamp technique (maximal currents were evoked by rectangular voltage steps to −10 mV, Figure 9A–F).^37^

As a result, we found that both **(*R*)-31** and **(*R*)-32** inhibited voltage-gated sodium currents at a concentration of 10 μM. Example sodium current traces are shown in Figure 9A, D in control (black trace), in the presence of **(*R*)-31** or **(*R*)-32** (blue trace), and after wash-out (red trace). An example neuron is also shown in Figure 9B, E (white circles show maximal current amplitudes). The averaged, maximal-normalized amplitudes of voltage-gated sodium currents were 1.0 in control, 0.66 ± 0.04 after application of **(*R*)-31** and 0.76 ± 0.05 after wash-out [control *vs***(*R*)-31***p* < 0.05 (Tukey’s post hoc test) *n* = 5, Figure 9C], whereas 0.61 ± 0.07 after application of **(*R*)-32** and 0.72 ± 0.08 after wash-out [control *vs***(*R*)-32***p* < 0.05 (Tukey’s post hoc test) *n* = 5, Figure 9F]. In consequence, **(*R*)-32** inhibited stronger maximal amplitudes of voltage-gated sodium currents than **(*R*)-31**. Consistently, in binding studies, **(*R*)-32** showed a higher affinity to sodium channels in comparison to **(*R*)-31**. Importantly, these *in vitro* results seem to correlate with *in vivo* data, *i.e.,***(*R*)-32** showed higher antiseizure potency *vs***(*R*)-31**.

Binding and electrophysiological studies described above proved interaction of **(*R*)-32** with rat sodium channels. Therefore, as a part of the more detailed functional characterization using automated patch clamp method, we tested the influence of **(*R*)-32** on sodium currents mediated by several Navx channels comprising 5 different human subunits, namely, Nav_1.1_, Nav_1.2_, Nav_1.3_, Nav_1.6_, and Nav_1.7_, which are involved in both seizure and/or pain modulation.^74,80,81^ As summarized in Tables S4–S8, **(*R*)-32** starting from 10 μM effectively and in concentration-dependent manner decreased sodium currents mediated by all aforementioned sodium channel subunits. Furthermore, **(*R*)-32** appeared to bind exclusively to sodium channels that are in an inactivated state (see pulse 2 in Tables S4–S8), but not to the channels that are in an open state (pulse 1) (for additional details, see Supporting Information).

In summary, based on *in vitro* binding and functional studies, it is suggested that the moderate interaction of **(*R*)-32** with the molecular targets such as TRPV1, sodium, and calcium channels may result in both broad-spectrum antiseizure and antinociceptive activities.

### *In Vitro* ADME-Tox Assays

We characterized drug-like and safety profiles of **(*R*)-31**, **(*R*)-32**, and **(*R*)-33** using *in vitro* tests, which involved determination of passive permeability, metabolic stability, DDIs predictions, and hepatotoxicity. This panel of ADME-Tox studies is typically used to further assess “developability” of new molecular entities, as an integral part of drug discovery process.

Precoated PAMPA Plate System Gentest (Corning, Tewksbury, MA, USA), a model of passive blood–brain barrier permeability, was used to test the **(*R*)-32** lead compound. The results were compared to caffeine’s permeability, which is commonly used as a permeable reference compound. In this study, **(*R*)-32** showed satisfactory passive penetration with a calculated permeability coefficient (*Pe*) of approximately 70% of that of caffeine (2.42 × 10^–6^*vs* 3.46 × 10^–6^ cm/s, respectively).

The incubation with human liver microsomes (HLMs) allows for the prediction of metabolic stability in the human body after *in vivo* administration. The obtained data indicated that % remaining of **(*R*)-31** (85.24), **(*R*)-32** (78.53), and **(*R*)-33** (70.32), in the respective reaction mixture, was found to be much higher in comparison to the reference drug verapamil, which was metabolically unstable in HLMs (30.84). Moreover, the potential metabolic biotransformation pathways in human were also predicted and are outlined in Table 4. UPLC materials and *in silico* results of the metabolic pathways and the most probable structures of metabolites are presented in Figures S8–S18.

**Table 4:** Metabolic Stability and the Most Probable Biotransformation Pathways in the Presence of HLMs

substrate	molecular mass (***m*/*z***)	% remaining	molecular mass of the metabolite (***m*/*z***)	metabolic pathway^a^
**(*R*)-31**	406.28	85.24	**454.01 (M1)**	***triple-hydroxylation***
			404.19 (M2)	*dehydrogenation*
			422.22 (M3)	*hydroxylation*
**(*R*)-32**	422.22	78.53	**438.25 (M1)**	***hydroxylation***
			420.22 (M2)	*dehydrogenation*
**(*R*)-33**	438.18	70.32	**454.26 (M1)**	***hydroxylation***
			436.21 (M2)	*dehydrogenation*
			454.08 (M3)	*hydroxylation*
**verapamil**^b^	455.31	30.84	**441.35 (M1)**	***demethylation***
			291.32 (M2)	*defragmentation*
			165.09 (M3)	*defragmentation*
			441.29 (M4)	*demethylation*
			427.33 (M5)	*double-demethylation*
			277.26 (M6)	*defragmentation*

a Main metabolic pathways have been bolded.

b Data for verapamil were previously reported in ref (82).

**Figure 10:**
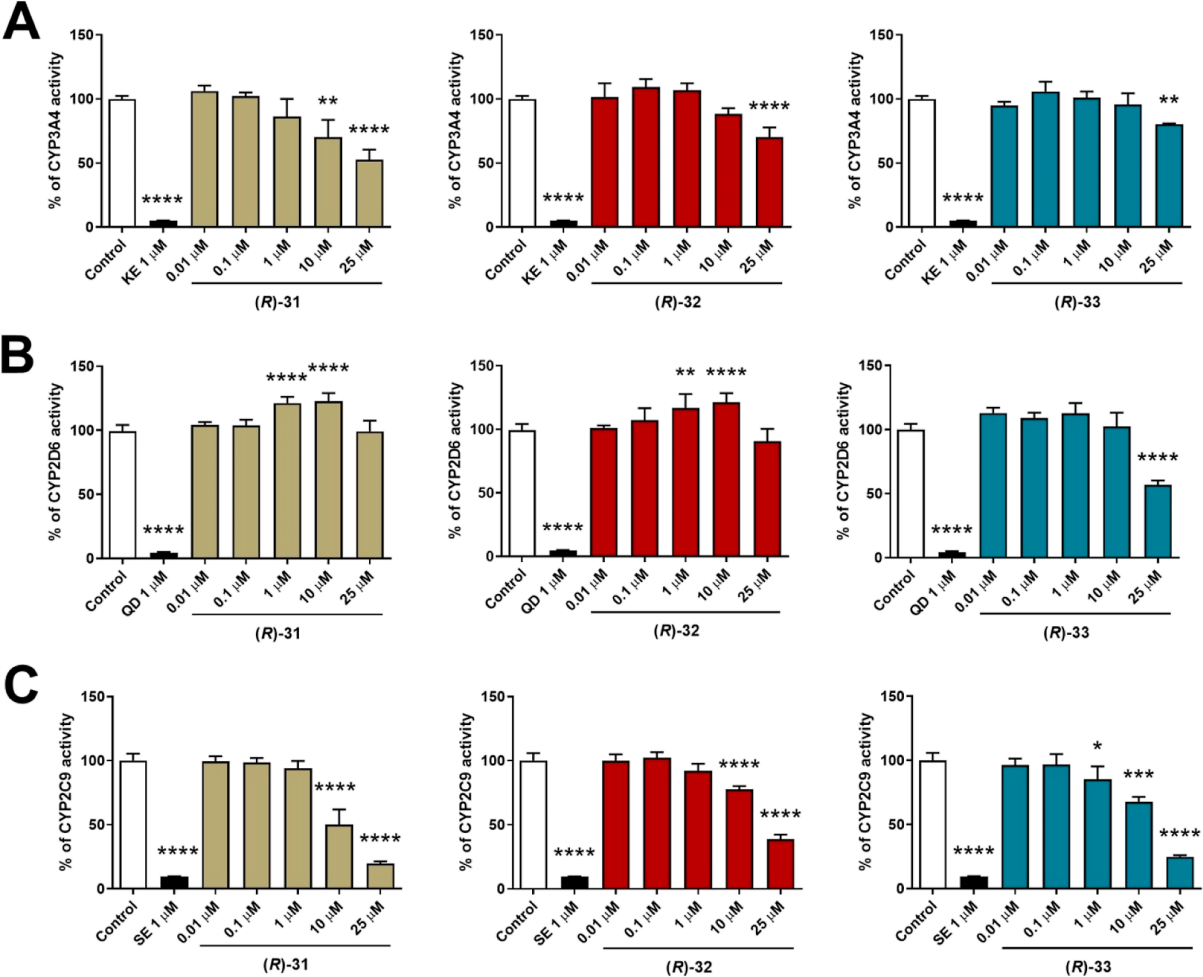
Influence of **(*R*)-31**, **(*R*)-32,** and **(*R*)-33** on: CYP3A4 activity (A) and the reference inhibitor ketoconazole (KE), CYP2D6 activity (B) and the reference inhibitor quinidine (QD), CYP2C9 activity (C) and the reference inhibitor sulfaphenazole (SE). The results are presented as means + SD. Statistical significance was evaluated by one-way ANOVA, followed by Bonferroni’s multiple comparison post hoc test (Graph Pad Prism 8.0.1 software) *p < 0.05, **p < 0.01, ***p < 0.001, ****p < 0.0001.

The effect of **(*R*)-31**, **(*R*)-32**, and **(*R*)-33** on cytochrome P450 isoforms 3A4, 2D6, and 2C9 is shown in Figure 10. In general, all the tested compounds inhibited significantly CYP3A4 isoform but only at the high concentration used, which is 25 μM. The strongest effect was observed for **(*R*)-31,** which significantly inhibited CYP3A4 also at 10 μM (Figure 10A). Regarding CYP2D6, weak activation by **(*R*)-31** and **(*R*)-32** was observed at 1 and 10 μM, whereas **(*R*)-33** slightly inhibited CYP2D6 at 25 μM (Figure 10B). The inhibition effect of enantiomers on CYP2C9 was also determined, but only at the highest concentrations (Figure 10C). In summary, the potential for DDIs of the tested enantiomers in comparison to the reference CYP inhibitors was estimated as very low.

**Figure 11:**
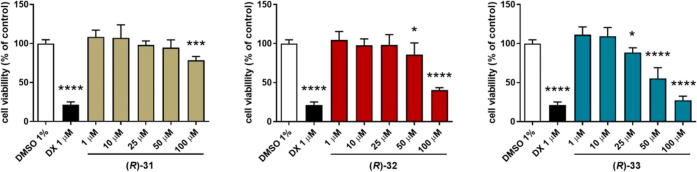
Effect of **(*R*)-31**, **(*R*)-32**, and **(*R*)-33** and cytostatic drug doxorubicin (DX) on the hepatoma HepG2 cell line viability after 72 h of incubation at 37 °C, 5% CO_2_. The results are presented as means + SD. Statistical significance was evaluated by one-way ANOVA, followed by Bonferroni’s multiple comparison post hoc test (Graph Pad Prism 8.0.1 software, San Diego, CA, USA): *p < 0.05, ***p < 0.001, ****p < 0.0001 *vs* negative control (DMSO 1% in growth media).

The hepatotoxicity potential was assessed with the HepG2 cell line and the reference cytostatic drug doxorubicin. Overall, the tested compounds showed moderate and generally acceptable hepatotoxicity in comparison to the reference compound. The highest toxicity was observed for the sulfur-containing compound, which reduced slightly, but statistically significant cell viability at concentration of 25 μM **(*R*)-33.** However, this result was still much less pronounced compared to doxorubicin, which decreased cell viability to approximately 20% at 1 μM (Figure 11).

## Conclusions

The present chemical and pharmacological studies led to the identification of new multimodal compounds belonging to functionalized phenylglycinamide derivatives. Several of these revealed broad-spectrum and potent protection across several acute seizure models in mice (*i.p*.), namely, MES, 6 Hz (32 mA), and 6 Hz (44 mA). Consequently, the most potent protection was observed for the ***R***-enantiomers of the racemates described previously.^38^ Compound **(*R*)-32**, which was identified as the lead compound, in addition to its activity in the aforementioned electrically induced seizures, was also effective in the *iv*PTZ seizure threshold test and chronic PTZ-kindling model in mice, indicating a broad spectrum and likely multimodal mechanism of action. **(*R*)-32**, similarly to the VPA, normalized the concentration of Glu, GABA, and mBDNF expression in mice after chronic PTZ-kindling model in mice. This molecule also revealed a potent antinociceptive effect in the formalin-induced tonic pain, capsaicin-induced pain, and especially in the OXPT-, as well as STZ-induced peripheral neuropathy. Furthermore, *in vivo* evaluations showed that at the effective doses, **(*R*)-32** had no influence on the neuromuscular strength and body temperature in mice. The mechanism of action of **(*R*)-32** is likely multimodal and involves TRPV1 antagonism (potentially novel molecular target for development of ASMs),^27^ as well as inhibition of sodium currents. Furthermore, both *in vivo* pharmacokinetic as well as *in vitro* studies indicated favorable ADME-Tox properties, making **(*R*)-32** an interesting candidate for further preclinical development with potential therapeutic utility in epilepsy and neuropathic pain indications.

In the next steps of the preclinical investigations, we plan to test **(*R*)-32** in the zebrafish Dravet syndrome model, as well as to focus on further understanding of its mechanism of action. We hypothesize that optimally selected and potentially synergistic multimodal pharmacodynamics (i.e., TRPV1/Na_v_x channels) may be a promising strategy for discovery of new ASMs with broad-spectrum of antiseizure and antinociceptive activities. Furthermore, TRPV1 inhibition may modulate neuroinflammation processes in the CNS, which have been recently established as one of the key players in seizure induction and epileptogenesis. Consequently, such multimodal molecules may offer a valuable advantage over currently available ASMs and may also possess disease-modifying or/and antiepileptogenic properties.

## Experimental Section

### Chemistry

#### General Information

All chemicals and solvents were purchased from commercial suppliers and were used without further purification. Melting points (mp) were determined in open capillaries on a Büchi 353 melting point apparatus (Büchi Labortechnik, Flawil, Switzerland). TLC and the gradient UPLC chromatography were used to assess the purity and homogeneity of the compounds. TLC was carried out on silica gel 60 F_254_ precoated aluminum sheets (Macherey-Nagel, Düren, Germany), using the following developing systems: S_1_–DCM/MeOH (9:0.3; *v/v*), S_2_–DCM/MeOH (9:0.5; *v/v*). Spots detection: UV light (λ = 254 nm). The UPLC and mass spectra (LC-MS) were obtained on Waters ACQUITY TQD system (Waters, Milford, CT, USA) with the MS-TQ detector and UV–vis-DAD eλ detector. The ACQUITY UPLC BEH C18, 1.7 μm (2.1 × 100 mm) column was used with the VanGuard Acquity UPLC BEH C18, 1.7 μm (2.1 × 5 mm) (Waters, Milford, CT, USA). Standard solutions (1 mg/mL) of each compound were prepared in analytical grade MeCN/water mixture (1:1; *v/v*). Conditions applied were as follows: eluent A (water/0.1% HCOOH), eluent B (MeCN/0.1% HCOOH), a flow rate of 0.3 mL/min, a gradient of 5–100% B over 10 min, and an injection volume of 10 μL. The UPLC retention times (*t*_R_) are given in minutes. The purity of target compounds determined by the use of the chromatographic UPLC method was ≥95%.

The UPLC analyses and high-resolution mass spectra (LC-HRMS) were obtained on Waters ACQUITY I-Class PLUS SYNAPT XS High Resolution Mass Spectrometer (Waters, Milford, CT, USA) with the MS-Q-TOF detector and UV–vis-DAD eλ detector. The ACQUITY UPLC CSH C18, 1.7 μm (2.1 × 100 mm) column was used with the VanGuard Acquity UPLC CSH C18, 1.7 μm (2.1 × 5 mm) (Waters, Milford, CT, USA). Standard solution (1 mg/mL) of compound was prepared in analytical grade MeCN/water mixture (1:1; *v/v*). Conditions applied were as follows: eluent A (water/0.1% HCOOH), eluent B (MeCN/0.1% HCOOH), a flow rate of 0.3 mL/min, a gradient of 5–100% B over 13 min, and an injection volume of 1 μL. Preparative column chromatography was performed using silica gel 60 (particle size 0.063–0.200; 70–230 Mesh ATM) purchased from Merck (Darmstadt, Germany). Elemental analyses (C, H, and N) for final compounds were carried out by a micro method using the elemental Vario EI III Elemental analyzer (Hanau, Germany). The results of elemental analyses were within 0.4% of the theoretical values. ^1^H NMR and ^13^C NMR spectra were obtained in a JEOL-500 spectrometer (JEOL USA, Inc. MA, USA) in CDCl_3_ operating at 500 MHz (^1^H NMR) and 126 MHz (^13^C NMR). Chemical shifts are reported in δ values (ppm) relative to TMS δ = 0 (^1^H), as an internal standard. The *J* values are expressed in Hertz (Hz). Signal multiplicities are represented by the following abbreviations: s (singlet), br s (broad singlet), d (doublet), br d (broad doublet), dd (double doublet), t (triplet), td (triple doublet), q (quartet), m (multiplet). Enantiomeric purity was determined using a chiral HPLC technique on a Shimadzu Prominence LC-2030C SD Plus system (Shimadzu Corporation, Kyoto, Japan) equipped with an Amylose-C (250× 4.6 mm) chiral column. The analysis was performed under the following conditions: column temperature: 20 °C, mixture of eluents: hexane/i- PrOH = 80/20 (*v/v*), flow rate: 1 mL/min, injection volume: 10 μL, analysis time: 40 min. (isocratic), detection at the wavelength λ = 210 nm. Enantiomeric purity is expressed in %.

### General Method for the Preparation of Compound **1**

In 20 mL of DCM, CDI (3.9 g, 24 mmol, 1.2 equiv) was dissolved. Then, while stirring, this solution was added to Boc-d,l-phenylglicine (5 g, 20 mmol, 1 equiv) that had been dissolved in 30 mL of DCM. Drops of the (20 mmol, 1 equiv) 3-trifluoromethylpiperazine solution in 30 mL of DCM were added after 0.5 h. The mixture was stirred for approximately 3 h at room temperature and evaporated to dryness. Using the following development system S_1_, column chromatography was used to purify the crude product. Following the concentration of organic solvents at low pressure, the compound was obtained as light oil.

### *Tert*-butyl (2-Oxo-1-phenyl-2-(4-(3-(trifluoromethyl)phenyl)piperazin-1-yl)ethyl) Carbamate (**1**)

Light yellow oil. Yield: 83% (7.7 g); TLC: *R*_*f*_ = 0.77 (S_1_); UPLC (purity >99%): *t*_R_ = 8.47 min. LC-MS (ESI): *m*/*z* calcd for C_24_H_28_N_3_O_3_F_3_ (M + H)^+^ 464.21; found, 464.3.

### General Method for the Preparation of Compound **2**

TFA (45 mmol, 3 equiv) was added to the solution of **1** (15 mmol, 1 equiv) in DCM (5 mL), and it was stirred at room temperature for 3 h. Afterward, the organic solvents were evaporated to dryness. After the resultant oil residue had been dissolved in 20 mL of water, then 25% ammonium hydroxide was cautiously added to pH = 8. The aqueous layer was extracted with DCM (3 × 20 mL), dried over Na_2_SO_4_, and concentrated to give the **2** as yellow oil. Without being purified, intermediate **2** was used as the substrate for the final reaction.

### 2-Amino-2-phenyl-1-(4-(3-(trifluoromethyl)phenyl)piperazin-1-yl)ethan-1-one (**2**)

Yellow oil. Yield: 97% (5.3 g); TLC: *R*_*f*_ = 0.25 (S_2_); UPLC (purity >99%): *t*_R_ = 4.89 min. LC-MS (ESI): *m*/*z* calcd for C_19_H_20_N_3_OF_3_ (M + H)^+^ 364.16; found, 364.2.

### General Method for the Preparation of the Final Compounds **3–12**

CDI (0.19 g, 1.2 mmol, 1.2 equiv) was dissolved in 10 mL of THF. Afterward, this solution was added to the appropriate aromatic acid (0.36 g, 1 mmol, 1 equiv) dissolved in 10 mL of THF (while stirring). After 0.5 h, the intermediate **2** (1 mmol, 1 equiv) dissolved in 5 mL of THF was added in drops. The mixture was stirred for approximately 12 h at room temperature and evaporated to dryness. Using the developing system S_1_, column chromatography was utilized to purify crude products. After concentrating organic solvents under low pressure and recrystallizing from methanol, the compounds were obtained as a white powder.

### *N*-(2-Oxo-1-phenyl-2-(4-(3-(trifluoromethyl)phenyl)piperazin-1-yl)ethyl)benzamide (**3**)

White solid. Yield: 86% (0.55 g); mp 140.9–141.2 °C; TLC: *R*_*f*_ = 0.77 (S_1_); UPLC (purity >99%): *t*_R_ = 7.98 min. LC-MS (ESI): *m*/*z* calcd for C_26_H_24_N_3_O_2_F_3_ (M + H)^+^ 468.19; found, 468.3. ^1^H NMR (500 MHz, CDCl_3_) δ 2.60 (ddd, *J* = 11.5, 8.0, 2.9 Hz, 1H, piperazine), 3.07 (ddd, *J* = 11.7, 8.3, 2.9 Hz, 1H, piperazine), 3.11–3.17 (m, 1H, piperazine), 3.22–3.32 (m, 1H, piperazine), 3.49–3.56 (m, 1H, piperazine), 3.58–3.66 (m, 1H, piperazine), 3.68–3.76 (m, 1H, piperazine), 3.98 (ddd, *J* = 13.0, 5.6, 3.2 Hz, 1H, piperazine), 6.10 (d, *J* = 7.5 Hz, 1H, CH), 6.96 (br d, *J* = 8.6 Hz, 1H, ArH), 7.01 (s, 1H, ArH), 7.09 (d, *J* = 8.0 Hz, 1H, ArH), 7.27–7.44 (m, 6H, ArH), 7.44–7.53 (m, 3H, ArH), 7.76–7.87 (m, 3H, ArH, NH). ^13^C NMR (126 MHz, CDCl_3_) δ 42.2, 45.2, 48.6, 54.4, 112.9 (br d, *J* = 3.6 Hz), 116.9 (br d, *J* = 3.6 Hz), 119.4, 124.2 (q, *J* = 272.0 Hz) 127.2, 128.0, 128.6, 128.7, 129.4, 129.8, 131.7 (q, *J* = 32.2 Hz), 131.8, 134.0, 137.6, 150.8, 166.3. Anal. Calcd for C_26_H_24_N_3_O_2_F_3_: C, 66.80; H, 5.17; N, 8.99. Found: C, 66.74; H, 5.28; N, 8.82.

### 2-Chloro-*N*-(2-oxo-1-phenyl-2-(4-(3-(trifluoromethyl)phenyl)piperazin-1-yl)ethyl) Benzamide (**4**)

White solid. Yield: 88% (0.44 g); mp 134.8–135.9 °C; TLC: *R*_*f*_ = 0.73 (S_1_); UPLC (purity >99%): *t*_R_ = 8.09 min. LC-MS (ESI): *m*/*z* calcd for C_26_H_23_N_3_O_2_ClF_3_ (M + H)^+^ 502.15; found, 502.3. ^1^H NMR (500 MHz, CDCl_3_) δ 2.58 (br d, *J* = 8.59 Hz, 1H, piperazine), 3.04 (ddd, *J* = 11.9, 8.2, 3.4 Hz, 1H, piperazine), 3.14 (ddd, *J* = 11.9, 6.2, 3.2 Hz, 1H, piperazine), 3.23–3.32 (m, 1H, piperazine), 3.49–3.57 (m, 1H, piperazine), 3.58–3.66 (m, 1H, piperazine), 3.69 (ddd, *J* = 13.2, 8.0, 3.4 Hz, 1H, piperazine), 3.98 (ddd, *J* = 13.2, 5.7, 3.4 Hz, 1H, piperazine), 6.09 (d, *J* = 7.5 Hz, 1H, CH), 6.95 (dd, *J* = 8.0, 2.3 Hz, 1H, ArH), 7.01 (s, 1H, ArH), 7.09 (d, *J* = 8.0 Hz, 1H, ArH), 7.27 (dd, *J* = 7.5, 1.7 Hz, 1H, ArH), 7.29–7.40 (m, 6H, ArH), 7.48–7.53 (m, 2H, ArH), 7.61 (dd, *J* = 8.0, 1.7 Hz, 1H, ArH), 7.92 (d, *J* = 6.9 Hz, 1H, NH). ^13^C NMR (126 MHz, CDCl_3_) δ 42.2, 45.2, 48.6, 54.8, 112.9 (br d, *J* = 3.6 Hz), 116.9, (br d, *J* = 3.6 Hz), 119.3, 124.6 (q, *J* = 272.0 Hz), 127.0, 128.1, 128.7, 129.3, 129.8 130.3, 130.4, 131.5 (q, *J* = 32.2 Hz), 131.6, 134.4, 137.3, 150.8, 165.3, 168.0. Anal. Calcd for C_26_H_23_N_3_O_2_ClF_3_: C, 62.22; H, 4.62; N, 8.37. Found: C, 62.44; H, 4.82; N, 8.26.

### 3-Chloro-*N*-(2-oxo-1-phenyl-2-(4-(3-(trifluoromethyl)phenyl)piperazin-1-yl)ethyl) Benzamide (**5**)

White solid. Yield: 86% (0.43 g); mp 151.7–152.4 °C; TLC: *R*_*f*_ = 0.74 (S_1_); UPLC (purity >99%): *t*_R_ = 8.45 min. LC-MS (ESI): *m*/*z* calcd for C_26_H_23_N_3_O_2_ClF_3_ (M + H)^+^ 502.15; found, 502.3. ^1^H NMR (500 MHz, CDCl_3_) δ 2.54–2.65 (m, 1H, piperazine), 3.07 (ddd, *J* = 11.9, 8.2, 3.4 Hz, 1H, piperazine), 3.13 (ddd, *J* = 11.9, 6.2, 3.2 Hz, 1H, piperazine), 3.24–3.32 (m, 1H, piperazine), 3.47–3.55 (m, 1H, piperazine), 3.56–3.66 (m, 1H, piperazine), 3.71 (ddd, *J* = 13.0, 7.9, 3.2 Hz, 1H, piperazine), 3.98 (ddd, *J* = 13.2, 6.0, 3.2 Hz, 1H, piperazine), 6.06 (d, *J* = 7.5 Hz, 1H, CH), 6.96 (dd, *J* = 8.0, 2.3 Hz, 1H, ArH), 7.01 (s, 1H, ArH), 7.09 (d, *J* = 7.5 Hz, 1H, ArH), 7.28–7.41 (m, 5H, ArH), 7.44 (ddd, *J* = 8.0, 2.3, 1.2 Hz, 1H, ArH), 7.47–7.51 (m, 2H, ArH), 7.62–7.69 (m, 1H, ArH), 7.79 (t, *J* = 2.0 Hz, 1H, ArH), 7.81 (d, *J* = 6.9 Hz, 1H, NH). ^13^C NMR (126 MHz, CDCl_3_) δ 42.2, 45.2, 48.6, 54.6, 112.9 (br d, *J* = 3.6 Hz), 117.0 (br d, *J* = 3.6 Hz), 119.4, 125.2 (q, *J* = 272.8 Hz), 125.3, 127.6, 128.0, 128.8, 129.4, 129.8, 129.9, 131.7 (q, *J* = 32.0 Hz), 131.8, 134.8, 135.8, 137.3, 150.8, 164.9, 168.2. Anal. Calcd for C_26_H_23_N_3_O_2_ClF_3_: C, 62.22; H, 4.62; N, 8.37. Found: C, 62.14; H, 4.73; N, 8.48.

### 4-Chloro-*N*-(2-oxo-1-phenyl-2-(4-(3-(trifluoromethyl)phenyl)piperazin-1-yl)ethyl) Benzamide (**6**)

White solid. Yield: 84% (0.42 g); mp 165.2–166.7 °C; TLC: *R*_*f*_ = 0.73 (S_1_); UPLC (purity = 98.5%): *t*_R_ = 8.41 min. LC-MS (ESI): *m*/*z* calcd for C_26_H_23_N_3_O_2_ClF_3_ (M + H)^+^ 502.15; found, 502.3. ^1^H NMR (500 MHz, CDCl_3_) δ 2.53–2.64 (m, 1H, piperazine), 3.02–3.10 (m, 1H, piperazine), 3.10–3.17 (m, 1H, piperazine), 3.23–3.33 (m, 1H, piperazine), 3.48–3.55 (m, 1H, piperazine), 3.57–3.65 (m, 1H, piperazine), 3.67–3.76 (m, 1H, piperazine), 3.98 (ddd, *J* = 13.0, 6.2, 3.2 Hz, 1H, piperazine), 6.05 (d, *J* = 6.9 Hz, 1H, CH), 6.96 (dd, *J* = 8.3, 2.0 Hz, 1H, ArH), 7.01 (s, 1H, ArH), 7.09 (d, *J* = 7.5 Hz, 1H, ArH), 7.27–7.35 (m, 2H, ArH), 7.36–7.40 (m, 4H, ArH), 7.46–7.52 (m, 2H, ArH), 7.70–7.77 (m, 2H, ArH), 7.80 (d, *J* = 6.9 Hz, 1H, NH). ^13^C NMR (126 MHz, CDCl_3_) δ 42.2, 45.2, 48.6 (d, *J* = 1.8 Hz), 54.5, 112.9 (br d, *J* = 3.6 Hz) 117.0 (br d, *J* = 3.6 Hz), 119.4, 124.2 (q, *J* = 272.8 Hz), 128.0, 128.7, 128.8, 128.9, 129.4, 129.8, 131.7 (q, *J* = 32.0H) 132.3, 137.4, 138.0, 150.8, 165.2, 168.3. Anal. Calcd for C_26_H_23_N_3_O_2_ClF_3_: C, 62.22; H, 4.62; N, 8.37. Found: C, 62.14; H, 4.49; N, 8.47.

### *N*-(2-Oxo-1-phenyl-2-(4-(3-(trifluoromethyl)phenyl)piperazin-1-yl)ethyl)-2 (trifluoromethyl)benzamide (**7**)

White solid. Yield: 87% (0.46 g); mp 165.2–166.7 °C; TLC: *R*_*f*_ = 0.72 (S_1_); UPLC (purity >99%): *t*_R_ = 8.18 min. LC-MS (ESI): *m*/*z* calcd for C_27_H_23_N_3_O_2_F_6_ (M + H)^+^ 536.17; found, 536.2. ^1^H NMR (500 MHz, CDCl_3_) δ 2.57 (ddd, *J* = 11.9, 8.2, 3.2 Hz, 1H, piperazine), 3.04 (ddd, *J* = 11.9, 8.2, 3.4 Hz, 1H, piperazine), 3.14 (ddd, *J* = 11.9, 6.0, 3.0 Hz, 1H, piperazine), 3.24–3.32 (m, 1H, piperazine) 3.47–3.56 (m, 1H, piperazine) 3.57–3.64 (m, 1H, piperazine), 3.68 (ddd, *J* = 13.1, 8.1, 3.2 Hz, 1H, piperazine), 3.94–4.04 (m, 1H, piperazine), 6.08 (d, *J* = 7.2 Hz, 1H, CH), 6.96 (dd, *J* = 8.3, 2.3 Hz, 1H, ArH), 7.01 (s, 1H, ArH), 7.09 (d, *J* = 7.7 Hz, 1H, ArH), 7.29–7.41 (m, 4H, ArH), 7.44 (br d, *J* = 7.2 Hz, 1H, NH), 7.46–7.58 (m, 5H, ArH), 7.64–7.68 (m, 1H, ArH).^13^C NMR (126 MHz, CDCl_3_) δ 42.2, 45.2, 48.6, 54.7, 112.9, (br d, *J* = 4.2 Hz), 116.9 (br d, *J* = 3.6 Hz), 119.4, 123.53 (q, *J* = 273.6 Hz), 124.2 (q, *J* = 272.8 Hz), 126.5 (q, *J* = 4.8 Hz), 127.6 (q, *J* = 32.0 Hz), 128.0, 128.7, 128.8, 129.3, 129.8, 130.1, 131.6 (q, *J* = 32.0 Hz), 132.0, 135.4, 136.9, 150.8, 166.6, 167.8. Anal. Calcd for C_27_H_23_N_3_O_2_F_6_: C, 60.56; H, 4.33; N, 7.85. Found: C, 60.38; H, 4.38; N, 7.95.

### *N*-(2-Oxo-1-phenyl-2-(4-(3-(trifluoromethyl)phenyl)piperazin-1-yl)ethyl)-3-(trifluoromethyl)benzamide (**8**)

White solid. Yield: 89% (0.48 g); mp 143.2–144.1 °C; TLC: *R*_*f*_ = 0.69 (S_1_); UPLC (purity >99%): *t*_R_ = 8.59 min. LC-MS (ESI): *m*/*z* calcd for C_27_H_23_N_3_O_2_F_6_ (M + H)^+^ 536.17; found, 536.2. ^1^H NMR (500 MHz, CDCl_3_) δ 2.54–2.64 (m, 1H, piperazine), 3.02–3.11 (m, 1H, piperazine), 3.11–3.18 (m, 1H, piperazine), 3.24–3.33 (m, 1H, piperazine), 3.47–3.57 (m, 1H, piperazine), 3.58–3.67 (m, 1H, piperazine), 3.69–3.78 (m, 1H, piperazine) 3.99 (ddd, *J* = 13.2, 6.3, 3.4 Hz, 1H, piperazine) 6.08 (d, *J* = 7.5 Hz, 1H, CH), 6.96 (dd, *J* = 8.0, 2.3 Hz, 1H, ArH), 7.01 (s, 1H, ArH), 7.10 (d, *J* = 7.5 Hz, 1H, ArH), 7.29–7.42 (m, 4H, ArH), 7.47–7.58 (m, 3H, ArH), 7.73 (d, *J* = 7.5 Hz, 1H, ArH), 7.90 (br d, *J* = 7.5 Hz, 1H, NH), 7.97 (d, *J* = 8.0 Hz, 1H, ArH), 8.07 (s, 1H, ArH).^13^C NMR (126 MHz, CDCl_3_) δ 42.3, 45.2, 48.6 (d, *J* = 3.0 Hz), 54.7, 112.9 (br d, *J* = 4.2 Hz), 117.0 (br d, *J* = 3.6 Hz), 119.4, 123.7 (q, *J* = 273.6 Hz), 124.2 (q, *J* = 272.8 Hz), 124.5 (br d, *J* = 3.6 Hz), 128.1, 128.4 (br d, *J* = 3.6 Hz), 128.9, 129.2, 129.4, 129.8, 130.4, 131.2 (q, *J* = 32.6 Hz), 131.7 (q, *J* = 32.0 Hz, 1C), 134.8, 137.2, 150.8, 164.8, 168.1. Anal. Calcd for C_27_H_23_N_3_O_2_F_6_: C, 60.56; H, 4.33; N, 7.85. Found: C, 60.65; H, 4.25; N, 7.72.

### *N*-(2-Oxo-1-phenyl-2-(4-(3-(trifluoromethyl)phenyl)piperazin-1-yl)ethyl)-4-(trifluoromethyl) Benzamide (**9**)

White solid. Yield: 86% (0.45 g); mp 103.4–104.2 °C; TLC: *R*_*f*_ = 0.71 (S_1_); UPLC (purity = 98.6%): *t*_R_ = 8.58 min. LC-MS (ESI): *m*/*z* calcd for C_27_H_23_N_3_O_2_F_6_ (M + H)^+^ 536.17; found, 536.2. ^1^H NMR (500 MHz, CDCl_3_) δ 2.54–2.63 (m, 1H, piperazine), 3.07 (td, *J* = 8.0, 4.0 Hz, 1H, piperazine), 3.14 (ddd, *J* = 11.9, 6.2, 3.2 Hz, 1H, piperazine), 3.23–3.33 (m, 1H, piperazine), 3.48–3.57 (m, 1H, piperazine), 3.57–3.65 (m, 1H, piperazine), 3.66–3.76 (m, 1H, piperazine), 3.99 (ddd, *J* = 13.3, 5.9, 3.2 Hz, 1H, piperazine), 6.06 (d, *J* = 6.9 Hz, 1H, CH), 6.96 (dd, *J* = 8.0, 2.3 Hz, 1H, ArH), 7.00–7.03 (m, 1H, ArH), 7.10 (d, *J* = 7.5 Hz, 1H, ArH), 7.29–7.41 (m, 4H, ArH), 7.50 (d, *J* = 6.9 Hz, 2H, ArH), 7.66 (d, *J* = 8.6 Hz, 2H, ArH), 7.91 (br d, *J* = 8.0 Hz, 3H, ArH, NH). ^13^C NMR (126 MHz, CDCl_3_) δ 42.3, 45.2, 48.6 (d, *J* = 2.4 Hz), 54.6, 112.9 (br d, *J* = 3.6 Hz), 117.0 (br d, *J* = 4.2 Hz), 119.4, 123.7 (q, *J* = 272.8 Hz), 124.2 (q, *J* = 272.2 Hz), 125.6, 127.7, 128.0, 128.9, 129.4, 129.8, 131.7 (q, *J* = 31.8 Hz), 133.5 (q, *J* = 32.8 Hz), 137.2, 150.8, 165.0, 168.1. Anal. Calcd for C_27_H_23_N_3_O_2_F_6_: C, 60.56; H, 4.33; N, 7.85. Found: C, 60.42; H, 4.22; N, 7.92.

### *N*-(2-Oxo-1-phenyl-2-(4-(3-(trifluoromethyl)phenyl)piperazin-1-yl)ethyl)-3-(trifluoromethyl)picolinamide (**10**)

White solid. Yield: 85% (0.45 g); mp 105.3–106.7 °C; TLC: *R*_*f*_ = 0.72 (S_1_); UPLC (purity >99%): *t*_R_ = 8.13 min. LC-MS (ESI): *m*/*z* calcd for C_26_H_22_N_4_O_2_F_6_ (M + H)^+^ 537.17; found, 537.1. ^1^H NMR (500 MHz, CDCl_3_) δ 2.63 (ddd, *J* = 11.7, 8.0, 3.2 Hz, 1H, piperazine), 3.03–3.19 (m, 2H, piperazine), 3.22–3.33 (m, 1H, piperazine), 3.47–3.58 (m, 1H, piperazine), 3.58–3.67 (m, 1H, piperazine), 3.67–3.80 (m, 1H, piperazine), 3.98 (ddd, *J* = 13.1, 6.2, 3.2 Hz, 1H, piperazine), 6.11 (d, *J* = 7.73 Hz, 1H, CH), 6.96 (dd, *J* = 8.3, 2.3 Hz, 1H, ArH), 7.01 (s, 1H, ArH), 7.09 (d, *J* = 7.7 Hz, 1H, ArH), 7.27–7.42 (m, 4H, ArH), 7.47–7.60 (m, 3H, ArH), 8.11 (dd, *J* = 8.0, 1.2 Hz, 1H, ArH), 8.74 (dd, *J* = 4.7, 1.3 Hz, 1H, ArH), 9.06 (d, *J* = 8.0 Hz, 1H, NH). ^13^C NMR (126 MHz, CDCl_3_) δ 42.1, 45.2, 48.7 (br d, *J* = 3.6 Hz), 54.2, 112.9 (br d, *J* = 4.2 Hz), 116.9 (br d, *J* = 3.6 Hz), 119.3, 122.9, (q, *J* = 273.4 Hz), 124.2 (q, *J* = 272.8 Hz), 125.5, 126.0 (q, *J* = 34.4 Hz), 128.0, 128.7, 129.4, 129.8, 131.6 (q, *J* = 31.8 Hz), 136.1 (br d, *J* = 6.0 Hz), 137.2, 149.0, 150.9 (d, *J* = 3.6 Hz), 162.3, 168.0. Anal. Calcd for C_26_H_22_N_4_O_2_F_6_: C, 58.21; H, 4.13; N, 10.44. Found: C, 58.08; H, 4.18; N, 10.57.

### *N*-(2-Oxo-1-phenyl-2-(4-(3-(trifluoromethyl)phenyl)piperazin-1-yl)ethyl)-4-(trifluoromethyl)picolinamide (**11**)

White solid. Yield: 88% (0.47 g); mp 101.2–102.4 °C; TLC: *R*_*f*_ = 0.71 (S_1_); UPLC (purity >99%): *t*_R_ = 8.76 min. LC-MS (ESI): *m*/*z* calcd for C_26_H_22_N_4_O_2_F_6_ (M + H)^+^ 537.17; found, 537.1. ^1^H NMR (500 MHz, CDCl_3_) δ 2.60–2.68 (m, 1H, piperazine), 3.05–3.12 (m, 1H, piperazine), 3.12–3.18 (m, 1H, piperazine), 3.24–3.32 (m, 1H, piperazine), 3.49–3.59 (m, 1H, piperazine), 3.61–3.69 (m, 1H, piperazine), 3.71–3.79 (m, 1H, piperazine), 4.00 (ddd, *J* = 13.2, 6.3, 3.4 Hz, 1H, piperazine), 6.08 (d, *J* = 7.5 Hz, 1H, CH), 6.96 (dd, *J* = 8.6, 2.3 Hz, 1H, ArH), 7.02 (s, 1H, ArH), 7.09 (d, *J* = 7.5 Hz, 1H, ArH), 7.28–7.40 (m, 4H, ArH), 7.49–7.55 (m, 2H, ArH), 7.60–7.64 (m, 1H, ArH), 8.36 (s, 1H, ArH), 8.77 (d, *J* = 4.6 Hz, 1H, ArH), 9.36 (d, *J* = 7.5 Hz, 1H, NH). ^13^C NMR (126 MHz, CDCl_3_) δ 42.2, 45.2, 48.7, 54.4, 112.9 (br d, *J* = 4.2 Hz), 116.9 (br d, *J* = 3.6 Hz), 118.4 (br d, *J* = 3.6 Hz), 119.4, 122.6 (q, *J* = 273.4 Hz), 124.2 (q, *J* = 272.2 Hz), 121.9 (br d, *J* = 3.0 Hz), 128.0, 128.8, 129.4, 129.8, 131.6 (q, *J* = 31.8 Hz), 137.1, 139.8 (q, *J* = 34.8 Hz), 149.5, 150.9, 151.2, 162.2, 167.9. Anal. Calcd for C_26_H_22_N_4_O_2_F_6_: C, 58.21; H, 4.13; N, 10.44. Found: C, 58.35; H, 4.08; N, 10.38.

### *N*-(2-Oxo-1-phenyl-2-(4-(3-(trifluoromethyl)phenyl)piperazin-1-yl)ethyl)-5-(trifluoromethyl)picolinamide (**12**)

White solid. Yield: 86% (0.46 g); mp 146.5–147.2 °C; TLC: *R*_*f*_ = 0.71 (S_1_); UPLC (purity = 98.2%): *t*_R_ = 8.76 min. LC-MS (ESI): *m*/*z* calcd for C_26_H_22_N_4_O_2_F_6_ (M + H)^+^ 537.17; found, 537.1. ^1^H NMR (500 MHz, CDCl_3_) δ 2.59–2.67 (m, 1H, piperazine), 3.04–3.11 (m, 1H, piperazine), 3.12–3.18 (m, 1H, piperazine), 3.23–3.31 (m, 1H, piperazine), 3.50–3.58 (m, 1H, piperazine), 3.61–3.68 (m, 1H, piperazine), 3.70–3.78 (m, 1H, piperazine), 4.00 (ddd, *J* = 13.2, 6.3, 3.4 Hz, 1H, piperazine), 6.06 (d, *J* = 7.5 Hz, 1H, CH), 6.96 (dd, *J* = 8.0, 2.3 Hz, 1H, ArH), 7.01 (s, 1H, ArH), 7.09 (d, *J* = 7.5 Hz, 1H, ArH), 7.27–7.41 (m, 4H, ArH), 7.49–7.55 (m, 2H, ArH), 8.05 (dd, *J* = 8.0, 1.7 Hz, 1H, ArH), 8.25 (d, *J* = 8.0 Hz, 1H, ArH), 8.82–8.86 (m, 1H, ArH), 9.38 (d, *J* = 7.5 Hz, 1H, NH). ^13^C NMR (126 MHz, CDCl_3_) δ 42.2, 45.2, 48.7, 54.4, 112.9 (br d, *J* = 3.6 Hz), 116.9 (br d, *J* = 3.6 Hz) 119.4, 123.2 (q, *J* = 272.2 Hz), 124.2 (q, *J* = 272.6 Hz), 128.0, 128.9 (q, *J* = 33.2 Hz), 128.8, 129.4, 129.8, 131. (q, *J* = 32.0 Hz), 134.7, (br d, *J* = 3.6 Hz), 137.1, 145.6 (br d, *J* = 3.6 Hz) 150.9, 152.6, 162.2, 167.9. Anal. Calcd for C_26_H_22_N_4_O_2_F_6_: C, 58.21; H, 4.13; N, 10.44. Found: C, 58.17; H, 4.19; N, 10.52.

### General Method for the Preparation of Intermediates **13–16**

In 10 mL of DCM, CDI (0.39 g, 2.4 mmol, 1.2 equiv) was dissolved. Then, while stirring, this solution was added to Boc-d,l-glycine (0.5 g, 2 mmol, 1 equiv) that had been dissolved in 10 mL of DCM. After 0.5 h, the respective piperazine derivatives (**A5–A8**, 2 mmol, 1 equiv) dissolved in 5 mL of DCM were added in drops. The mixture was stirred for approximately 3 h at room temperature and evaporated to dryness. Using the developing system S_1_, column chromatography was utilized to purify crude products. After concentrating organic solvents under low pressure, the compounds were obtained as light oils.

### *Tert*-butyl (2-Oxo-1-phenyl-2-((1-phenylpyrrolidin-3-yl)amino)ethyl)carbamate (**13**)

Light yellow oil. Yield: 92% (0.72 g); TLC: *R*_*f*_ = 0.65 (S_1_); UPLC (purity = 98.1%): *t*_R_ = 7.58 min. LC-MS (ESI): *m*/*z* calcd for C_23_H_29_N_3_O_3_ (M + H)^+^ 396.22; found, 396.3.

### *Tert*-butyl (2-Oxo-1-phenyl-2-((1-(3-(trifluoromethyl)phenyl)pyrrolidin-3-yl)amino)ethyl) Carbamate (**14**)

Light yellow oil. Yield: 89% (0.82 g); TLC: *R*_*f*_ = 0.72 (S_1_); UPLC (purity = 95.7%): *t*_R_ = 8.59 min. LC-MS (ESI): *m*/*z* calcd for C_24_H_28_N_3_O_3_F_3_ (M + H)^+^ 464.21; found, 464.3.

### *Tert*-butyl (2-Oxo-1-phenyl-2-((1-(3-(trifluoromethoxy)phenyl)pyrrolidin-3-yl)amino)ethyl) Carbamate (**15**)

Light yellow oil. Yield: 92% (0.88 g); TLC: *R*_*f*_ = 0.74 (S_1_); UPLC (purity >99%): *t*_R_ = 9.09 min. LC-MS (ESI): *m*/*z* calcd for C_24_H_28_N_3_O_4_F_3_ (M + H)^+^ 480.21; found, 480.2.

### *Tert*-butyl (2-Oxo-1-phenyl-2-((1-(3-((trifluoromethyl)thio)phenyl)pyrrolidin-3-yl)amino)ethyl) Carbamate (**16**)

Yellow oil. Yield: 90% (0.88 g); TLC: *R*_*f*_ = 0.75 (S_1_); UPLC (purity = 97.8%): *t*_R_ = 9.50 min. LC-MS (ESI): *m*/*z* calcd for C_24_H_28_N_3_O_3_SF_3_ (M + H)^+^ 496.18; found, 496.2.

### General Method for the Preparation of Compounds **17–20**

TFA (3 equiv) was added to the **13**–**16** (1.7 mmol, 1 equiv) solution in DCM (10 mL) and was stirred at room temperature for 3 h. Afterward, the organic solvents were evaporated to dryness. After, the resultant oil residue had been dissolved in 20 mL of water, and then 25% ammonium hydroxide was cautiously added to pH = 8. The aqueous layer was extracted with DCM (3 × 20 mL), dried over Na_2_SO_4_, and concentrated to give the **17**–**20** as yellow oils. Without being purified, intermediates **17**–**20** were used as substrates for the final reaction.

### 2-Amino-2-phenyl-*N*-(1-phenylpyrrolidin-3-yl)acetamidemide (**17**)

Yellow oil. Yield: 98% (0.49 g); TLC: *R*_*f*_ = 0.24 (S_2_); UPLC (purity = 97.2%): *t*_R_ = 4.85 min. LC-MS (ESI): *m*/*z* calcd for C_18_H_21_N_3_O (M + H)^+^ 296.17; found, 296.3.

### 2-Amino-2-phenyl-*N*-(1-(3-(trifluoromethyl)phenyl)pyrrolidin-3-yl)acetamide (**18**)

Yellow oil. Yield: 97% (0.63 g); TLC: *R*_*f*_ = 0.25 (S_2_); UPLC (purity = 98.4%): *t*_R_ = 5.09 min. LC-MS (ESI): *m*/*z* calcd for C_19_H_20_N_3_OF_3_ (M + H)^+^ 364.12; found, 364.3.

### 2-Amino-2-phenyl-*N*-(1-(3-(trifluoromethoxy)phenyl)pyrrolidin-3-yl)acetamide (**19**)

Yellow oil. Yield: 98% (0.64 g); TLC: *R*_*f*_ = 0.27 (S_2_); UPLC (purity >99%): *t*_R_ = 5.51 min. LC-MS (ESI): *m*/*z* calcd for C_19_H_20_N_3_O_2_F_3_ (M + H)^+^ 380.15; found, 380.2.

### 2-Amino-2-phenyl-*N*-(1-(3-((trifluoromethyl)thio)phenyl)pyrrolidin-3-yl)acetamide (**20**)

Yellow oil. Yield: 97% (0.64 g); TLC: *R*_*f*_ = 0.27 (S_2_); UPLC (purity = 98.7%): *t*_R_ = 5.72 min. LC-MS (ESI): *m*/*z* calcd for C_19_H_20_N_3_OSF_3_ (M + H)^+^ 396.13; found, 396.2.

### General Method for the Preparation of the Final Compounds **21–24**

10 mL of DCM was used to dissolve intermediates **17**–**20** (1.5 mmol, 1 equiv). Then, triethylamine (TEA) (4.5 mmol, 3 equiv) was added to the solution, stirring at 0 °C. After, acetyl chloride (2.3 mmol, 1.5 equiv) was added dropwise to the final compounds 21–24 at 0 °C in an ice bath. After bringing the reaction mixture to room temperature and stirring it for a further 2 h, it was evaporated to dryness. Next, the crude product was purified applying column chromatography, using developing system S_2_. After concentrating organic solvents at low pressure and using diethyl ether for wash-up, the compounds were obtained as white powders.

### 2-Acetamido-2-phenyl-*N*-(1-phenylpyrrolidin-3-yl)acetamide (**21**)

White solid. Yield: 90% (0.45 g); mp 189.7–190.5 °C; TLC: *R*_*f*_ = 0.48 (S_2_); UPLC (purity >99%): *t*_R_ = 5.65 min. LC-MS (ESI): *m*/*z* calcd for C_20_H_23_N_3_O_2_ (M + H)^+^ 338.18 found 338.3. ^1^H NMR (500 MHz, CDCl_3_) δ 1.79 (s, 1H, pyrrolidine), 1.86–1.93 (m, 3H, CH_3_), 2.13 (dt, *J* = 13.7, 6.8 Hz, 1H, pyrrolidine), 3.06 (ddd, *J* = 9.7, 5.7, 3.7 Hz, 1H, pyrrolidine), 3.17–3.31 (m, 2H, pyrrolidine), 3.39–3.50 (m, 1H, pyrrolidine), 4.43–4.50 (m, 1H, pyrrolidine), 5.75 (d, *J* = 7.7 Hz, 1H, CH), 6.41–6.50 (m, 2H, ArH), 6.63–6.70 (m, 1H, ArH), 7.13 (br d, *J* = 7.7 Hz, 1H, NH), 7.16–7.22 (m, 2H, ArH), 7.24–7.31 (m, 3H, ArH), 7.31–7.39 (m, 2H, ArH), 7.40–7.46 (m, 1H, NH). ^13^C NMR (126 MHz, CDCl_3_) δ 23.2, 31.5, 45.8, 49.8, 53.1, 56.6 (br d, *J* = 4.2 Hz), 111.9, 116.3, 127.0, 128.3, 128.9, 129.3 (br d, *J* = 9.0 Hz), 138.1, 138.2, 147.5, 169.9, 170.2. Anal. Calcd for C_20_H_23_N_3_O_2_: C, 71.19; H, 6.87; N, 12.45. Found: C, 71.36; H, 6.69; N, 12.28.

### 2-Acetamido-2-phenyl-*N*-(1-(3-(trifluoromethyl)phenyl)pyrrolidin-3-yl)acetamide (**22**)

White solid. Yield: 91% (0.55 g); mp 203.7–204.4 °C; TLC: *R*_*f*_ = 0.53 (S_2_); UPLC (purity >99%): *t*_R_ = 6.59 min. LC-MS (ESI): *m*/*z* calcd for C_21_H_22_N_3_O_2_F_3_ (M + H)^+^ 406.17 found 406.3. ^1^H NMR (500 MHz, CDCl_3_) δ 1.77–1.94 (m, 4H, CH_3_, pyrrolidine), 2.05–2.23 (m, 1H, pyrrolidine), 2.93–3.04 (m, 1H, pyrrolidine), 3.15–3.30 (m, 2H, pyrrolidine), 3.38–3.51 (m, 1H, pyrrolidine), 4.36–4.52 (m, 1H, pyrrolidine) 5.77 (dd, *J* = 8.0, 2.9 Hz, 1H, CH), 6.52–6.63 (m, 2H, ArH), 6.81–6.92 (m, 1H, ArH), 7.12 (dd, *J* = 7.9, 2.7 Hz, 1H, NH), 7.23–7.39 (m, 6H, ArH), 7.56–7.70 (m, 1H, NH). ^13^C NMR (126 MHz, CDCl_3_) δ 23.1, 31.4, (br d, *J* = 19.3 Hz), 45.7 (br d, *J* = 6.0 Hz), 49.7, 53.0 (br d, *J* = 31.4 Hz), 56.5, 108.0, 112.5 114.8, 124.5 (q, *J* = 272.8 Hz), 126.9, 128.3, 129.0, 129.6 (br d, *J* = 4.2 Hz), 131.4 (q, *J* = 32.0 Hz), 138.1 (d, *J* = 15.7 Hz), 147.4, 169.9, 170.3 (d, *J* = 3.0 Hz). Anal. Calcd for C_21_H_22_N_3_O_2_F_3_: C, 62.21; H, 5.47; N, 10.36. Found: C, 62.38; H, 5.32; N, 10.18.

### 2-Acetamido-2-phenyl-*N*-(1-(3-(trifluoromethoxy)phenyl)pyrrolidin-3-yl)acetamide (**23**)

White solid. Yield: 88% (0.55 g); mp 173.1–174.2 °C; TLC: *R*_*f*_ = 0.55 (S_2_); UPLC (purity >99%): *t*_R_ = 6.82 min. LC-MS (ESI): *m*/*z* calcd for C_21_H_22_N_3_O_3_F_3_ (M + H)^+^ 422.16 found 422.2. ^1^H NMR (500 MHz, CDCl_3_) δ 1.76–1.93 (m, 4H, CH_3_, pyrrolidine), 2.05–2.21 (m, 1H, pyrrolidine), 2.98 (dd, *J* = 9.9, 3.6 Hz, 1H, pyrrolidine), 3.13–3.27 (m, 2H, pyrrolidine), 3.36–3.53 (m, 1H, pyrrolidine), 4.41–4.51 (m, 1H, pyrrolidine) 5.77 (dd, *J* = 8.0, 3.2 Hz, 1H, CH), 6.22 (br d, *J* = 7.7 Hz, 1H, ArH), 6.32 (td, *J* = 8.5, 1.9 Hz, 1H, ArH), 6.47–6.54 (m, 1H, ArH), 7.07–7.19 (m, 2H, ArH), 7.22–7.39 (m, 5H, ArH, NH), 7.61 (br dd, *J* = 18.9, 7.2 Hz, 1H, NH). ^13^C NMR (126 MHz, CDCl_3_) δ 23.1, 31.4 (br d, *J* = 19.9 Hz), 45.8 (br d, *J* = 4.2 Hz), 49.7 (br d, *J* = 5.4 Hz), 53.0 (br d, *J* = 21.1 Hz), 56.5, 104.3, 108.0, 110.2, 119.6, 121.6 (d, *J* = 1.2 Hz), 126.9, 128.3, 128.9, 130.1, 138.1 (q, *J* = 33.8 Hz), 148.6, 150.5, 169.9, 170.26 (d, *J* = 2.4 Hz). Anal. Calcd for C_21_H_22_N_3_O_3_F_3_: C, 59.85; H, 5.26; N, 9.97. Found: C, 60.02; H, 5.14; N, 10.08.

### 2-Acetamido-2-phenyl-*N*-(1-(3-((trifluoromethyl)thio)phenyl)pyrrolidin-3-yl)acetamide (**24**)

White solid. Yield: 92% (0.6 g); mp 168.8–169.7 °C; TLC: *R*_*f*_ = 0.57 (S_2_); UPLC (purity = 98.2%): *t*_R_ = 7.17 min. LC-MS (ESI): *m*/*z* calcd for C_21_H_22_N_3_O_2_SF_3_ (M + H)^+^ 438.14 found 438.2. ^1^H NMR (500 MHz, CDCl_3_) δ 1.75–1.94 (m, 4H, CH_3_, pyrrolidine), 2.06–2.22 (m, 1H, pyrrolidine), 2.94–3.03 (m, 1H, pyrrolidine), 3.13–3.29 (m, 2H, pyrrolidine), 3.44 (ddd, *J* = 12.4, 10.1, 6.2 Hz, 1H, pyrrolidine), 4.46 (dt, *J* = 10.7, 5.1 Hz, 1H, pyrrolidine), 5.77 (dd, *J* = 7.9, 3.0 Hz, 1H, CH), 6.46–6.55 (m, 1H, ArH), 6.66 (br d, *J* = 8.6 Hz, 1H, ArH), 6.90–6.95 (m, 1H, ArH), 7.11 (dd, *J* = 7.9, 3.9 Hz, 1H, ArH), 7.16–7.39 (m, 6H, ArH, NH), 7.57–7.67 (m, 1H, NH). ^13^C NMR (126 MHz, CDCl_3_) δ 23.1, 31.4 (d, *J* = 19.3 Hz), 45.7 (d, *J* = 5.4 Hz), 49.7 (d, *J* = 6.6 Hz), 52.9 (d, *J* = 22.3 Hz), 56.6 (d, *J* = 5.4 Hz), 114.0, 118.9, 123.5, 124.9, 126.9 (d, *J* = 2.4 Hz), 128.3, 128.9, 129.9 (q, *J* = 306.6 Hz), 130.0 (d, *J* = 2.4 Hz), 138.1 (q, *J* = 32.6 Hz), 147.9, 169.9, 170.3 (d, *J* = 2.4 Hz). Anal. Calcd for C_21_H_22_N_3_O_2_SF_3_: C, 57.66; H, 5.07; N, 9.61. Found: C, 57.54; H, 5.02; N, 9.73.

### General Method for the Preparation of Compounds **(*R*)-25**–**(*R*)-27** and **(*S*)-25**–**(*S*)-27**

20 mL of DCM was used to dissolve of *N*,*N*-dicyclohexylcarbodiimide (DCC) (1.97 g, 9.6 mmol, 1.2 equiv). This solution was then added, while stirring, to the appropriate d- or l-Boc-phenylglycine (2.0 g, 8 mmol, 1 equiv), which previously had been dissolved in 20 mL of DCM. After 0.5 h, the respective piperazine derivatives (8 mmol, 1 equiv) dissolved in 10 mL of DCM was added in drops. The mixture was stirred for approximately 3 h at a room temperature and evaporated to dryness. Column chromatography was used to purify the crude products, utilizing mixture S_1_ as the developing system. Compounds **(*R*)-25**–**(*R*)-27** and **(*S*)-25**–**(*S*)-27** were obtained as light oils, followed by concentration of organic solvents under reduced pressure.

### (***R***)*-tert*-Butyl (2-Oxo-1-phenyl-2-(4-(3-(trifluoromethyl)phenyl)piperazin-1-yl)ethyl) Carbamate (**(*R*)-25**)

Light yellow oil. Yield: 85% (3.14 g); TLC: *R*_*f*_ = 0.78 (S_1_); UPLC (purity >99%): *t*_R_ = 8.35 min. LC-MS (ESI): *m*/*z* calcd for C_24_H_28_N_3_O_3_F_3_ (M + H)^+^ 464.21; found, 464.4.

### (***S***)*-tert*-Butyl (2-Oxo-1-phenyl-2-(4-(3-(trifluoromethyl)phenyl)piperazin-1-yl)ethyl) Carbamate (**(*S*)-25**)

Light yellow oil. Yield: 82% (3.03 g); TLC: *R*_*f*_ = 0.77 (S_1_); UPLC (purity >99%): *t*_R_ = 8.44 min. LC-MS (ESI): *m*/*z* calcd for C_24_H_28_N_3_O_3_F_3_ (M + H)^+^ 464.21; found, 464.3.

### (***R***)*-tert*-Butyl (2-Oxo-1-phenyl-2-(4-(3-(trifluoromethoxy)phenyl)piperazin-1-yl)ethyl) Carbamate (**(*R*)-26**)

Light yellow oil. Yield: 84% (3.21 g); TLC: *R*_*f*_ = 0.80 (S_1_); UPLC (purity >99%): *t*_R_ = 8.84 min. LC-MS (ESI): *m*/*z* calcd for C_24_H_28_N_3_O_4_ F_3_ (M + H)^+^ 481.21 found 481.4.

### (***S***)*-tert*-Butyl (2-Oxo-1-phenyl-2-(4-(3-(trifluoromethoxy)phenyl)piperazin-1-yl)ethyl) Carbamate (**(*S*)-26**)

Light yellow oil. Yield: 82% (3.13 g); TLC: *R*_*f*_ = 0.81 (S_1_); UPLC (purity >99%): *t*_R_ = 8.92 min. LC-MS (ESI): *m*/*z* calcd for C_24_H_28_N_3_O_4_ F_3_ (M + H)^+^ 481.21 found 481.3.

### (***R***)*-tert*-Butyl (2-Oxo-1-phenyl-2-(4-(3-((trifluoromethyl)thio)phenyl)piperazin-1-yl)ethyl) Carbamate (**(*R*)-27**)

Light yellow oil. Yield: 84% (3.31 g); TLC: *R*_*f*_ = 0.82 (S_1_); UPLC (purity >99%): *t*_R_ = 8.95 min. LC-MS (ESI): *m*/*z* calcd for C_24_H_28_N_3_O_3_SF_3_ (M + H)^+^ 496.18 found 496.2.

### (***S***)*-tert*-Butyl (2-Oxo-1-phenyl-2-(4-(3-((trifluoromethyl)thio)phenyl)piperazin-1-yl)ethyl) Carbamate (**(*S*)-27**)

Light yellow oil. Yield: 86% (3.39 g); TLC: *R*_*f*_ = 0.82 (S_1_); UPLC (purity >99%): *t*_R_ = 8.99 min. LC-MS (ESI): *m*/*z* calcd for C_24_H_28_N_3_O_3_SF_3_ (M + H)^+^ 496.18 found 496.2.

### General Method for the Preparation of Compounds **(*R*)-28**–**(*R*)-30** and **(*S*)-28**–**(*S*)-30**

TFA (18 mmol, 3 equiv) was added to the solution containing **(*R*)-25–(*R*)-27** and **(*S*)-25–(*S*)-27** (6 mmol, 1 equiv) in DCM (20 mL) and was stirred at room temperature for 3 h. Afterward, the organic solvents were evaporated to dryness. After, the resultant oil residue had been dissolved in 20 mL of water, and then 25% ammonium hydroxide was cautiously added to pH = 8. The aqueous layer was extracted with DCM (3 × 20 mL), dried over Na_2_SO_4_, and concentrated to give **(*R*)-28**–**(*R*)-30** and **(*S*)-28**–**(*S*)-30** as yellow or bronze oils. Intermediates **(*R*)-28**–**(*R*)-30** and **(*S*)-28**–**(*S*)-30** were advanced to the last step reaction without further purification.

### (***R***)-2-Amino-2-phenyl-1-(4-(3-(trifluoromethyl)phenyl)piperazin-1-yl)ethan-1-one (**(*R*)-28**)

Yellow oil. Yield: 98% (2.14 g); TLC: *R*_*f*_ = 0.24 (S_2_); UPLC (purity >99%): *t*_R_ = 4.84 min. LC-MS (ESI): *m*/*z* calcd for C_19_H_20_N_3_OF_3_ (M + H)^+^ 364.16; found, 364.2.

### (***S***)-2-Amino-2-phenyl-1-(4-(3-(trifluoromethyl)phenyl)piperazin-1-yl)ethan-1-one (**(*S*)-28**)

Yellow oil. Yield: 99% (2.16 g); TLC: *R*_*f*_ = 0.25 (S_2_); UPLC (purity >99%): *t*_R_ = 4.89 min. LC-MS (ESI): *m*/*z* calcd for C_19_H_20_N_3_OF_3_ (M + H)^+^ 364.16; found, 364.2.

### (***R***)-2-Amino-2-phenyl-1-(4-(3-(trifluoromethoxy)phenyl)piperazin-1-yl)ethan-1-one (**(*R*)-29**)

Yellow oil. Yield: 99% (2.25 g); TLC: *R*_*f*_ = 0.27 (S_2_); UPLC (purity >99%): *t*_R_ = 5.48 min. LC-MS (ESI): *m*/*z* calcd for C_19_H_20_N_3_O_2_F_3_ (M + H)^+^ 380.15 found 380.3.

### (***S***)-2-Amino-2-phenyl-1-(4-(3-(trifluoromethoxy)phenyl)piperazin-1-yl)ethan-1-one (**(*S*)-29**)

Yellow oil. Yield: 97% (2.20 g); TLC: *R*_*f*_ = 0.27 (S_2_); UPLC (purity >99%): *t*_R_ = 5.52 min. LC-MS (ESI): *m*/*z* calcd for C_19_H_20_N_3_O_2_F_3_ (M + H)^+^ 380.15 found 380.2.

### (***R***)-2-Amino-2-phenyl-1-(4-(3-((trifluoromethyl)thio)phenyl)piperazin-1-yl)ethan-1-one (**(*R*)-30**)

Yellow oil. Yield: 98% (2.32 g); TLC: *R*_*f*_ = 0.27 (S_2_); UPLC (purity >99%): *t*_R_ = 5.59 min. LC-MS (ESI): *m*/*z* calcd for C_19_H_20_N_3_OSF_3_ (M + H)^+^ 396.13 found 396.2.

### (***S***)-2-Amino-2-phenyl-1-(4-(3-((trifluoromethyl)thio)phenyl)piperazin-1-yl)ethan-1-one (**(*S*)-30**)

Yellow oil. Yield: 97% (2.30 g); TLC: *R*_*f*_ = 0.26 (S_2_); UPLC (purity >99%): *t*_R_ = 5.62 min. LC-MS (ESI): *m*/*z* calcd for C_19_H_20_N_3_OSF_3_ (M + H)^+^ 396.13 found 396.2.

### General Method for the Preparation of the Final Compounds **(*R*)-31**–**(*R*)-33** and **(*S*)-31**–**(*S*)-33**

20 mL of DCM was used to dissolve the intermediates **(*R*)-28**–**(*R*)-30** and **(*S*)-28–(*S*)-30** (5.5 mmol, 1 equiv). Then, at 0 °C, triethylamine (TEA) (16.5 mmol, 3 equiv) was added and mixed. To obtained the final compounds **(*R*)-31**–**(*R*)-33** and **(*S*)-31**–**(*S*)-33**, acetyl chloride (8.25 mmol, 1.5 equiv) was added dropwise at 0 °C (ice bath). Then, the reaction mixture was allowed to warm up to room temperature and was stirred for an additional 2 h and then evaporated to dryness. Column chromatography was used to purify the crude products, utilizing mixture S_2_ as the developing system. **(*R*)-31–(*R***)-**33** and (***S*)-31–(*S*)-33** were obtained as white solids, followed by the concentration of organic solvents under reduced pressure and wash-up with diethyl ether.

### (***R***)-*N*-(2-Oxo-1-phenyl-2-(4-(3-(trifluoromethyl)phenyl)piperazin-1-yl)ethyl)acetamide (**(*R*)-31**)

White solid. Yield: 92% (2.05 g); mp 112.9–113.5 °C; TLC: *R*_*f*_ = 0.53 (S_2_); UPLC (purity >99%): *t*_R_ = 6.66 min. LC-MS (ESI): *m*/*z* calcd for C_21_H_22_N_3_O_2_F_3_ (M + H)^+^ 406.17; found, 406.2. UPLC/HRMS (purity >99%): *t*_R_ = 6.97 min. HRMS (ESI-QTOF): *m*/*z* calcd for C_21_H_22_N_3_O_2_F_3_ (M + H)^+^ 406.1664; found, 406.1698. ^1^H NMR (500 MHz, CDCl_3_) δ 1.99 (s, 3H, CH_3_), 2.58 (t, *J* = 8.31 Hz, 1H, piperazine), 3.08 (ddd, *J* = 11.7, 8.0, 3.2 Hz, 1H, piperazine), 3.12–3.19 (m, 1H, piperazine), 3.25–3.33 (m, 1H, piperazine), 3.54 (ddd, *J* = 13.3, 5.9, 2.9 Hz, 1H, piperazine) 3.61–3.70 (m, 1H, piperazine), 3.75–3.84 (m, 1H, piperazine), 4.01 (ddd, *J* = 13.3, 6.3, 3.3 Hz, 1H, piperazine), 5.90 (d, *J* = 7.5 Hz, 1H, CH), 7.01 (br d, *J* = 7.2 Hz, 1H, NH), 7.07–7.15 (m, 2H, ArH), 7.18 (d, *J* = 7.7 Hz, 1H, ArH), 7.28–7.43 (m, 6H). ^13^C NMR (126 MHz, CDCl_3_) δ 23.4, 41.8, 44.8, 49.4, 49.6, 53.9, 113.6, 118.4, 120.3, 124.0 (q, *J* = 272.8 Hz), 127.9, 128.7, 129.4, 130.1, 131.9 (q, *J* = 32.2 Hz) 137.5, 149.6, 168.4, 169.3. Chiral HPLC > 99% ee (*t*_R_ = 12.820 min). Anal. Calcd for C_21_H_22_N_3_O_2_F_3_: C, 62.21; H, 5.47; N, 10.36. Found: C, 62.04; H, 5.29; N, 10.53.

### (***S***)-*N*-(2-Oxo-1-phenyl-2-(4-(3-(trifluoromethyl)phenyl)piperazin-1-yl)ethyl)acetamide (**(*S*)-31**)

White solid. Yield: 90% (2.00 g); mp 109.8–110.6 °C; TLC: *R*_*f*_ = 0.52 (S_2_); UPLC (purity >99%): *t*_R_ = 6.70 min. LC-MS (ESI): *m*/*z* calcd for C_21_H_22_N_3_O_2_F_3_ (M + H)^+^ 406.17; found, 406.2. ^1^H NMR (500 MHz, CDCl_3_) δ 1.99 (s, 3H, CH_3_), 2.53–2.61 (m, 1H, piperazine), 3.01–3.15 (m, 2H, piperazine), 3.22–3.30 (m, 1H, piperazine), 3.41–3.50 (m, 1H, piperazine), 3.56 (ddd, *J* = 13.3, 7.9, 3.2 Hz, 1H, piperazine), 3.69 (ddd, *J* = 13.2, 7.9, 3.3 Hz, 1H, piperazine), 3.95 (ddd, *J* = 13.1, 6.2, 3.3 Hz, 1H, piperazine), 5.91 (d, *J* = 7.5 Hz, 1H, CH) 6.95 (dd, *J* = 8.3, 2.3 Hz, 1H, ArH), 7.00 (s, 1H, ArH), 7.03 (br d, *J* = 7.5 Hz, 1H, NH), 7.09 (d, *J* = 7.5 Hz, 1H, ArH), 7.26–7.44 (m, 6H, ArH).^13^C NMR (126 MHz, CDCl_3_) δ 23.4, 42.1, 45.2, 48.6 (d, *J* = 3.0 Hz), 53.9, 112.9 (br d, *J* = 3.6 Hz, 1C), 116.90, (br d, *J* = 3.6 Hz), 119.4, 124.2 (q, *J* = 272.4 Hz), 127.9, 128.6, 129.3, 129.8, 131.6 (q, *J* = 32.0 Hz), 137.7, 150.8, 168.3, 169.2. Chiral HPLC > 99% ee (*t*_R_ = 16.818 min). Anal. Calcd for C_21_H_22_N_3_O_2_F_3_: C, 62.21; H, 5.47; N, 10.36. Found: C, 62.35; H, 5.22; N, 10.24.

### (***R***)-*N*-(2-Oxo-1-phenyl-2-(4-(3-(trifluoromethoxy)phenyl)piperazin-1-yl)ethyl)acetamide (**(*R*)-32**)

White solid. Yield: 91% (2.11 g); mp 122.4–123.3 °C; TLC: *R*_*f*_ = 0.54 (S_2_); UPLC (purity >99%): *t*_R_ = 7.12 min. LC-MS (ESI): *m*/*z* calcd for C_21_H_22_N_3_O_3_F_3_ (M + H)^+^ 422.16; found, 422.3. UPLC/HRMS (purity >99%): *t*_R_ = 7.15 min. HRMS (ESI-QTOF): *m*/*z* calcd for C_21_H_22_N_3_O_3_F_3_ (M + H)^+^ 422.1691; found, 422.1747. ^1^H NMR (500 MHz, CDCl_3_) δ 1.98 (s, 3H, CH_3_), 2.54 (td, *J* = 7.95, 3.87 Hz, 1H, piperazine), 2.98–3.11 (m, 2H, piperazine), 3.19–3.26 (m, 1H, piperazine), 3.39–3.48 (m, 1H, piperazine), 3.51–3.59 (m, 1H, piperazine), 3.63–3.71 (m, 1H, piperazine), 3.93 (ddd, *J* = 13.0, 6.2, 3.2 Hz, 1H, piperazine), 5.91 (d, *J* = 7.5 Hz, 1H, CH), 6.59 (s, 1H, ArH), 6.67–6.73 (m, 2H, ArH), 7.05 (br d, *J* = 7.5 Hz, 1H, NH), 7.20 (t, *J* = 8.2 Hz, 1H, ArH), 7.27–7.42 (m, 5H, ArH).^13^C NMR (126 MHz, CDCl_3_) δ 23.4, 42.1, 45.1, 48.5 (d, *J* = 9.0 Hz), 53.9, 108.9, 112.2, 114.3, 120.5 (q, *J* = 257.1 Hz), 127.9, 128.6, 129.3, 130.3, 137.7, 150.3, 151.2 168.3, 169.2. Chiral HPLC > 99% ee (*t*_R_ = 11.847 min). Anal. Calcd for C_21_H_22_N_3_O_3_F_3_: C, 59.85; H, 5.26; N, 9.97. Found: C, 59.71; H, 5.20; N, 10.14.

### (***S***)-*N*-(2-Oxo-1-phenyl-2-(4-(3-(trifluoromethoxy)phenyl)piperazin-1-yl)ethyl)acetamide (**(*S*)-32**)

White solid. Yield: 90% (2.09 g); mp 124.2–125.1 °C; TLC: *R*_*f*_ = 0.55 (S_2_); UPLC (purity >99%): *t*_R_ = 7.19 min. LC-MS (ESI): *m*/*z* calcd for C_21_H_22_N_3_O_3_F_3_ (M + H)^+^ 422.16; found, 422.2. ^1^H NMR (500 MHz, CDCl_3_) δ 1.99 (s, 3H, CH_3_), 2.47–2.59 (m, 1H, piperazine), 2.97–3.12 (m, 2H, piperazine), 3.18–3.28 (m, 1H, piperazine), 3.40–3.48 (m, 1H, piperazine), 3.55 (ddd, *J* = 13.3, 8.0, 3.2 Hz, 1H, piperazine), 3.68 (ddd, *J* = 13.1, 8.0, 3.3 Hz, 1H, piperazine), 3.93 (ddd, *J* = 13.1, 6.2, 3.3 Hz, 1H, piperazine), 5.91 (d, *J* = 7.5 Hz, 1H, CH), 6.56–6.63 (m, 1H, ArH), 6.67–6.72 (m, 2H, ArH), 7.01 (br d, *J* = 7.5 Hz, 1H, NH), 7.21 (t, *J* = 8.2 Hz, 1H, ArH), 7.27–7.42 (m, 5H, ArH). ^13^C NMR (126 MHz, CDCl_3_) δ 23.4, 42.1, 45.1, 48.5 (d, *J* = 9.7 Hz), 53.9, 109.0, 112.2, 114.3, 120.5 (q, *J* = 257.1 Hz), 127.9, 128.6, 129.3, 130.3, 137.7, 150.3 (d, *J* = 1.2 Hz), 152.0, 168.3, 169.2. Chiral HPLC > 99% ee (*t*_R_ = 15.796 min). Anal. Calcd for C_21_H_22_N_3_O_3_F_3_: C, 59.85; H, 5.26; N, 9.97. Found: C, 59.64; H, 5.14; N, 9.82.

### (***R***)-*N*-(2-Oxo-1-phenyl-2-(4-(3-((trifluoromethyl)thio)phenyl)piperazin-1-yl)ethyl)acetamide (**(*R*)-33**)

White solid. Yield: 90% (2.16 g); mp 127.8–128.6 °C; TLC: *R*_*f*_ = 0.56 (S_2_); UPLC (purity = 98.2%): *t*_R_ = 7.23 min. LC-MS (ESI): *m*/*z* calcd for C_21_H_22_N_3_O_2_SF_3_ (M + H)^+^ 438.14; found, 438.3. UPLC/HRMS (purity = 98.3%): *t*_R_ = 7.51 min. HRMS (ESI-QTOF): *m*/*z* calcd for C_21_H_22_N_3_O_2_SF_3_ (M + H)^+^ 438.1463; found, 438.1506. ^1^H NMR (500 MHz, CDCl_3_) δ 1.99 (s, 3H, CH_3_), 2.56 (ddd, *J* = 11.7, 8.0, 3.2 Hz, 1H, piperazine), 2.99–3.13 (m, 2H, piperazine), 3.18–3.29 (m, 1H, piperazine), 3.40–3.49 (m, 1H, piperazine), 3.56 (ddd, *J* = 13.1, 7.8, 3.2 Hz, 1H, piperazine), 3.68 (ddd, *J* = 13.2, 7.9, 3.3 Hz, 1H, piperazine), 3.94 (ddd, *J* = 13.1, 6.2, 3.3 Hz, 1H, piperazine), 5.91 (d, *J* = 7.5 Hz, 1H, CH), 6.86–6.93 (m, 1H, ArH), 6.99–7.06 (m, 2H, ArH, NH), 7.12 (d, *J* = 7.7 Hz, 1H, ArH), 7.24–7.42 (m, 6H, ArH).^13^C NMR (126 MHz, CDCl_3_) δ 23.4, 42.1, 45.1, 48.6 (br d, *J* = 10.3 Hz), 53.9 118.5, 123.7, 125.3, 127.8, 127.9, 128.6, 129.3, 129.7 (q, *J* = 308.4 Hz), 130.1, 137.7, 151.3, 168.3, 169.2. Chiral HPLC > 99% ee (*t*_R_ = 12.343 min). Anal. Calcd for C_21_H_22_N_3_O_2_SF_3_: C, 57.66; H, 5.07; N, 9.61. Found: C, 57.78; H, 4.91; N, 9.49.

### (***S***)-*N*-(2-Oxo-1-phenyl-2-(4-(3-((trifluoromethyl)thio)phenyl)piperazin-1-yl)ethyl)acetamide (**(*S*)-33**)

White solid. Yield: 91% (2.18 g); mp 124.3–125.1 °C; TLC: *R*_*f*_ = 0.57 (S_2_); UPLC (purity >99%): *t*_R_ = 7.26 min. LC-MS (ESI): *m*/*z* calcd for C_21_H_22_N_3_O_2_SF_3_ (M + H)^+^ 438.14; found, 438.2. ^1^H NMR (500 MHz, CDCl_3_) δ 1.99 (s, 3H, CH_3_), 2.50–2.61 (m, 1H, piperazine), 2.98–3.11 (m, 2H, piperazine), 3.19–3.31 (m, 1H, piperazine), 3.41–3.50 (m, 1H, piperazine), 3.56 (ddd, *J* = 13.1, 7.8, 3.2 Hz, 1H, piperazine), 3.69 (ddd, *J* = 13.0, 8.0, 3.3 Hz, 1H, piperazine), 3.94 (ddd, *J* = 13.1, 6.2, 3.3 Hz, 1H, piperazine), 5.91 (d, *J* = 7.5 Hz, 1H, CH), 6.90 (dd, *J* = 8.5, 1.9 Hz, 1H, ArH), 7.01 (br d, *J* = 7.5 Hz, 1H, NH), 7.04, (s, 1H, ArH), 7.12 (d, *J* = 7.5 Hz, 1H, ArH), 7.25–7.42 (m, 6H, ArH).^13^C NMR (126 MHz, CDCl_3_) δ 23.4, 42.10, 45.1, 48.6 (br d, *J* = 10.3 Hz), 53.9, 118.6, 123.7, 125.3, 127.8, 127.9, 128.6, 128.7 (q, *J* = 308.4 Hz), 129.3, 130.1, 137.6, 151.3, 168.3, 169.2. Chiral HPLC > 99% ee (*t*_R_ = 17.760 min). Anal. Calcd for C_21_H_22_N_3_O_2_SF_3_: C, 57.66; H, 5.07; N, 9.61. Found: C, 57.93; H, 5.33; N, 9.72.

### Antiseizure Activity

#### Animals

For the *in vivo* investigations, adult male Albino Swiss mice weighing between 22 and 26 g were employed. They were housed in colony cages with conventional laboratory settings, including a 12 h natural light–dark cycle, a temperature range of 20–24 °C, an air humidity of 45–65%, and unrestricted access to food (chow pellets) and tap water. The mice were given 7 days to acclimate to life in the lab. All operations involving animals and their care were carried out in compliance with Polish and current EU regulations regarding animal experimentation. According to the European Communities Council Directive of September 22, 2010 (2010/63/EU), the research was conducted in compliance with experimental protocols approved by the Local Ethics Committee in Lublin (144/2018, 85/2019, 25/2021, 122/2022, 13/2021, 67/2021, 46/2021, and 29/2023). According to the 3Rs (replacement, reduction, and refinement) guideline, every attempt was made to reduce animal suffering and to utilize only the amount of animals required to generate trustworthy scientific data.

#### Antiseizure Activity and Acute Neurotoxicity

Four mice per group were randomly assigned to each experimental group in the initial screening experiments (each mouse was used just once). Four groups, each with eight animals, received injections of different doses of the investigated substances in order to determine the ED_50_ or TD_50_ values. The PIs for the compounds investigated and reference ASMs were calculated by dividing the TD_50_ value, as determined in the chimney test by the respective ED_50_ value, as determined in the MES, *sc*PTZ, or 6 Hz (32 mA or 44 mA) tests (PI = TD_50_/ED_50_). The PIs are a measure of the potential therapeutic window of the tested agent.

All substances were suspended in Tween 80 (1% aqueous solution) and administered *i.p*. as a single injection at a volume of 10 mL/kg. On each day of experimentation, fresh solutions were prepared. The detailed *in vivo* procedures are described elsewhere: the maximal electroshock seizure test (MES),^83^ the subcutaneous pentylenetetrazole seizure test (*sc*PTZ),^84^ the 6 Hz psychomotor seizure model (32 mA and 44 mA),^85^ and the chimney test.^86^ The reference ASMs were purchased from commercial suppliers: VPA (Sigma-Aldrich, St. Louis, MO, USA), LCS and LEV (UCB Pharma, Braine l’Alleud, Belgium).

### Timed *iv*PTZ Seizure Threshold Test and the Grip Strength Test in Mice

30 min prior to the testing, eutomers **(*R*)-31**, **(*R*)-32**, and **(*R*)-33** were given *i.p*. after being suspended in a 1% Tween 80 solution. To reduce the number of animals utilized, neuromuscular strength measurements were made right before the seizure threshold test. The grip-strength test and the timed *iv*PTZ test experimental protocols were covered in full elsewhere.^87^

### PTZ-Induced Kindling in Mice

The procedure was performed as described in detail in our previous studies.^88,89^ In short, **(*R*)-32** was suspended in 1% Tween 80, and VPA (as sodium salt) and PTZ (Sigma-Aldrich, St. Louis, MO, USA) were dissolved in saline. **(*R*)-32**, VPA, or vehicle were administered *i.p*. every 24 h. Seizures were induced three times a week by *i.p*. administration of PTZ at a subconvulsive dose of 40 mg/kg. 30 min following the administration of **(*R*)-32**, VPA, or vehicle, PTZ was injected. The seizure severity was scored using the modified Racine’s scale. Experimental grouping: (a) 1% Tween + saline (nonkindled control group not exposed to the FST), (b) 1% Tween + saline (nonkindled control group exposed to the FST), (c) 1% Tween + PTZ (PTZ-kindled control group); (d) VPA at 150 mg/kg + PTZ (positive control group); and (e)–(g) **(*R*)-32** at 20, 40, or 80 mg/kg + PTZ. The locomotor activity test, the elevated plus maze test, and the FST were performed 24 h after the last PTZ injection, according to the methods described in detail elsewhere. After completion of behavioral tests, animals were sacrificed. Hippocampi and cortices were dissected from the brains, frozen, and stored at −80 °C until assay. Following each PTZ injection, the mean seizure severity scores were determined for each experimental group. A mixed effects model for repeated measurements was used for analysis, along with Tukey’s post hoc test. The percentage of fully kindled mice was compared using Fisher’s exact probability test.

### Spontaneous Electrographic Bursting in an *In Vitro* Model of Pharmacoresistant Seizure-like Activity

Evaluation of spontaneous recurrent electrographic discharges (REDs) from the medial entorhinal cortex in acute brain slices obtained from Sprague–Dawley rats that had previously experienced kainate-induced status epilepticus (KA-rats) were performed as previously described (West *et al.*, 2018).^90^ Between 2 and 3 weeks after kainate treatment, acute horizontal brain slices containing the entorhinal cortex and hippocampus were made. Slices were transferred to the submersion recording chambers of an eight-channel Scientifica Slicemaster (Scientifica Inc., Uckfield, UK). Extracellular field excitatory postsynaptic potentials (fEPSPs) were then recorded from layer II of the medial entorhinal cortex from 8 brain slices simultaneously. Spontaneous REDs were recorded for a 20 min baseline period. Afterward, either investigational compounds or control artificial cerebral spinal fluid (ACSF) was applied by bath exchange for 20 min. The investigational compound was first dissolved in DMSO and then diluted to the final working concentration in ACSF (working concentration of DMSO was 0.01%). Finally, investigational compounds were washed from the recording chamber *via* control ACSF perfusion for 20 min to assess reversibility. Data were acquired using pClamp 10.4.2 and analyzed using the included clampfit 10.4 software. After applying a 5 Hz digital high-pass filter, a threshold search method was used to identify and quantify burst duration, frequency, and amplitude of REDs. These parameters were binned in 60 s increments, normalized to baseline, and plotted versus time. The effects of investigational compounds were statistically evaluated after a 20 min exposure using a paired 2-tailed Student’s *t*-test with significance set at *p* < 0.05.

### Determination of GABA and Glutamate Concentrations in Murine Brain Structures

GABA and glutamate concentrations were determined using liquid chromatography tandem mass spectrometry (LC-MS/MS) in the mouse prefrontal cortex and hippocampus. Toronto Research Chemicals Inc. was the source of the standards for both analytes (Toronto, ON, Canada). GABA and glutamate stock standard solutions were made in methanol and deionized water, respectively, and kept cold at 4 °C. The stock solutions were suitably diluted to create a range of solution combinations with the appropriate concentrations. Prior to analysis, a hand-held pestle and glass tube homogenizer (Potter–Elvehjem PTFE pestle and glass tube, Sigma-Aldrich, St. Louis, MO, USA) were used to homogenize mouse brains in distilled water at a ratio of 50 μL/mg. Following a 10 min centrifugation at 8000*g* for 10 min at 4 °C, homogenates were diluted 10 times with 0.1% formic acid in MeCN from the supernatant. Samples (10 μL) were deproteinized with 80 μL of 0.1% formic acid in MeCN by shaking for 10 min (IKA Vibrax VXR, IKA Werke GmbH & Co. KG, Staufen im Breisgau, Germany) and centrifuged for 5 min at the speed of 8000*g* (Eppendorf miniSpin centrifuge) after isotope-labeled GABA-d6 and glutamate-d5 (Toronto Research Chemicals Inc., Toronto, ON, Canada) were added as internal standards (10 μL at the concentration of 500 ng/mL). The autosampler vials were filled with the acquired supernatants. Chromatographic separation was performed using an Excion LC AC HPLC system (Sciex, USA) and an XBridgeTM HILIC analytical column (2.1 × 150 mm, 3.5 μm, Waters, Ireland) with the oven temperature set at 25 °C. A 2 μL sample volume was injected into the LC-MS/MS system, while the autosampler temperature was kept at 15 °C. The mobile phase was mixed at a ratio of 70:30 and ran at 0.3 mL/min, containing 0.1% formic acid in acetonitrile and 0.1% formic acid in water. A Sciex QTRAP 4500 triple quadrupole mass spectrometer (Sciex, USA) was used for mass spectrometric detection. Ion generation was accomplished using positive ion mode electrospray ionization (ESI). In the selected reaction monitoring mode (SRM), the tandem mass spectrometer was run at unit resolution to track the transition of the protonated molecular ions for GABA and glutamate, respectively, at *m*/*z* 104 to 87 and *m*/*z* 104 to 69 and *m*/*z* 148 to 84 and *m*/*z* 148 to 102 (the first pair served as a quantifier and the second as an identity verification qualifier). The monitored pairs were *m*/*z* 110 to 93 for the isotope tagged GABA-d6 and *m*/*z* 153 to 88 for glutamate-d5. Using a Harvard infusion pump, the standard solution was continuously infused at a rate of 7 μL/min to optimize the mass spectrometric conditions for GABA and glutamate. At 450 °C, the ion source temperature was kept constant. At 5000 V, the voltage for the ionspray was established. The collision gas (CAD) was set to medium and the curtain gas (CUR) to 40 psi. The software Applied Biosystems Analyst version 1.7 was used for both data processing and acquisition. Plotting the ratio of the investigated compound’s peak area to the corresponding internal standard against compound concentration resulted in the construction of the calibration curves, which were then produced by weighted (1/*x*·*x*) linear regression analysis. Owing to the stable isotope standards availability and the high endogenous quantities of glutamate and GABA, calibration curves were created using repeated dilutions of the calibrators in water. The method’s validated quantification ranges fell within the expected concentration ranges, which were 100 to 5000 μg/g of brain tissue with accuracy ranging from 90.89 to 108.43% for glutamate and GABA, respectively, and from 90.48 to 111.36% for both. During the normal analysis of the samples, no stability-related issues or substantial matrix effect were found. Tukey’s post hoc test was employed in conjunction with one-way ANOVA to examine changes in glutamate and GABA concentrations.

### BDNF and proNGF Expressions in the Hippocampus and Cortex of PTZ-Kindled Mice

The Western Blot method was used to assess the expression of proNGF and mBDNF in the mouse brain and hippocampal tissues. Using a bead homogenizer (Bead Ruptor Elite, Omni International, USA), the cortical and hippocampal regions of the mouse brain were weighted and homogenized at a ratio of 9 μL/mg in 2% SDS supplemented with a cocktail of protease (Thermo Scientific, Walthman, MA, USA) and phosphatase inhibitors (Sigma-Aldrich, St. Louis, MO, USA). Samples were then centrifuged at 10,000*g* for 10 min at 4 °C after being denatured for 10 min at 95 °C. Using the PierceTM BCA Protein Assay Kit (Thermo Scientific, Walthman, MA, USA), the total protein content in the obtained supernatants was ascertained. Following the determination of the appropriate protein concentration, the samples were heated for 10 min at 95 °C in a loading buffer containing 10% 2-mercaptoethanol at a ratio of 3:1. Equal amounts of protein (45 μg each lane) were placed onto Any kD precast polyacrylamide gels (Bio-Rad, Hercules, CA, USA; Criterion, TGX Stain-Free gel) and electrophoresed at 170 V for 60 min. Following their transfer to a polyvinylidene difluoride (PVDF) membrane (Bio-Rad, Hercules, CA, USA), the separated proteins were blocked using a 5% albumin solution in TBST. Primary antibodies, rabbit polyclonal anti-NGF (ab6199, Abcam, 1:1000), and rabbit monoclonal anti-BDNF (ab108319, Abcam, 1:1000) were incubated on the membranes for an overnight period at 4 °C. Subsequently, goat antirabbit IgG peroxidase-conjugated antibody (ab205718, Abcam, 1:5000) was applied to the membranes. The ECL method (Western Bright Quantum, Advansta Inc., San Jose, CA, USA) was used to detect the proteins. The G-Box Imaging System (Syngene, Frederick, MD, USA) was used to photograph the membranes’ chemiluminescence, and Gene Tools software (Syngene, Frederick, MD, USA) was used to assess the protein expression and express it as a percentage of the membrane lane’s total protein content. One-way ANOVA with Tukey’s post hoc test was utilized to examine changes in the relative expression of the proteins that were evaluated.

### Capsaicin-Induced Hypothermia Test in Mice

The changes in body temperature in the capsaicin-induced hypothermia model were evaluated as described previously. Eutomers **(*R*)-31**, **(*R*)-32**, and **(*R*)-33** were suspended in a 1% Tween 80 and administered *i.p*. 15 min before injection of capsaicin (5 mg/kg, *i.p*.). The corresponding vehicles (1% DMSO or 1% Tween 80) were given to the control animals. Using an electronic thermometer (ThermoWorks, Alpine, Utah, USA), changes in the mouse’s rectal temperature were recorded by inserting the rectal probe approximately 2 cm into the mouse’s rectum. After −15, 0, 15, 30, 60, 90, 120, and 180 min, the measurements were collected. Subsequently, the variations in rectal temperature between the baseline (few minutes for the groups receiving compounds and BCTC in conjunction with the vehicle, or 1 min for the groups receiving the vehicle, compounds, and BCTC in combination with the capsaicin) and the corresponding time point Δ*T* (°C) were computed and examined.

### Antinociceptive Activity

The experimental groups consisted of 10 adult male Albino Swiss mice (CD-1, 18–25 g). Each animal was tested only once. Immediately after the assay, the animals were sacrificed by cervical dislocation. Behavioral measurements were observed by trained observers. The *in vivo* antinociceptive assays were in accordance with Polish regulations and the European Union Directive of 22 September 2010 (2010/63/EU). All operations were approved by the Local Ethics Committee in Cracow, Poland (104/2015, 279/2019, and 614/2022) and conducted in accordance with the guidelines set forth by the International Council on Laboratory Animal Science (ICLAS). The tested materials were administered intraperitoneally half an hour before the test, after being suspended in a 1% aqueous solution of Tween-80. 30 min before the test, the animals in the control group (negative control) received an appropriate dose of the vehicle (Tween-80, 1% aqueous solution, *i.p*.). The experimental *in vivo* procedures were previously reported for the formalin test,^91^ and compounds **(*R*)-31** and **(*R*)-32** were tested in three doses, 25, 50, and 100 mg/kg. Before formalin application, different groups of animals were injected *i.p*. with vehicle (10 mL/kg, negative control). The *in vivo* procedure for the model of capsaicin-induced nociception was previously reported;^92^ the animals were pretreated with vehicle (10 mL/kg, negative control), and the dose–response of investigated compounds was evaluated at 25, 50, and 100 mg/kg. The *in vivo* procedure for the model of OXPT-induced peripheral neuropathy was previously reported;^93^ the mice with developed tactile allodynia were pretreated *i.p*. with test compounds **(*R*)-31** or **(*R*)-32** (50, 75, and 100 mg/kg) and vehicle. The *in vivo* procedure of streptozotocin-induced hyperglycemia was previously reported;^94^ the mice with developed mechanical allodynia were pretreated *i.p*. with test compound **(*R*)-31** or **(*R*)-32** (25, 50, and 100 mg/kg) and vehicle.

The means ± standard error of the mean (SD) are used to present data. The great majority of the data was analyzed using GraphPad Prism Software (v.5). One-way analysis of variance (ANOVA) and the post hoc Dunnett’s multiple comparison test or two-way ANOVA were used to calculate statistically significant differences between groups. The significance threshold was established at *p* < 0.05. The ED_50_ values were statistically determined with 95% confidence limits using the log-probit method.

### Pharmacokinetic Studies

#### Animals and Study Design

The study was performed on 8–10 week old male CD-1 mice with a mean weight of 28.5 g (range 25–32 g). They were housed in conditions of constant temperature with the 12:12 h light–dark cycle and free access to food and water. The investigated compound was suspended in 1% Tween solution in water and administered *i.p*. at the doses of 25 and 50 mg/kg. Moreover, the compound was dissolved in a mixture of DMSO/PEG400/water (1:4:5, *v*/*v*/*v*) and administered *p.o*. at a dose of 25 mg/kg. The mice were sacrificed by decapitation under isoflurane anesthesia at the following time points: 5, 15, 30, 60, 120, 240, and 480 min after dosing and blood and brains were harvested. After allowing the blood to coagulate for 20 min at room temperature, the serum was separated using a 5000*g* Eppendorf miniSpin centrifuge (Germany) for 10 min. Samples were kept at −80 °C until they were analyzed. The First Ethical Committee on Animal Experimentation in Kraków granted approval for all animal treatments (license no. 270/2019).

#### Analytical Method

Concentrations of compound **(*R*)-32** in mouse serum and brain tissue were measured using a liquid chromatography tandem mass spectrometry method (LC-MS/MS). Mouse brains were homogenized in distilled water at the ratio of 1:4 (w/v) using the ULTRA-TURRAX T10 basic tissue homogenizer (IKA, Germany). To 50 μL of brain homogenates or serum samples, 150 μL of 0.1% formic acid in acetonitrile with an addition of valsartan (used as an internal standard, IS) was added. Samples were shaken for 10 min (IKA Vibrax VXR, Germany) and then centrifuged for 5 min at the speed of 8000*g* (Eppendorf miniSpin centrifuge, Germany). Supernatants were transferred into the autosampler vials, and a sample volume of 1 μL was injected into the LC-MS/MS system.

Liquid chromatography was performed on an Exion LC AC HPLC system (Sciex, USA) with a Hypersil Gold C18 analytical column (3 × 50 mm, 5 μm, Thermo Scientific, USA). A gradient elution program was conducted for chromatographic separation with mobile phase A (0.1% formic acid in acetonitrile) and mobile phase B (0.1% formic acid in water). The initial mobile phase composition was 95% B and 5% A for the first 2 min with a linear gradient to 5% B in the next 2 min and then isocratic mode for 2 min with the following rapid change back to 95% B in 0.1 min. The remaining time of elution was set at 95% B. The whole HPLC operation lasted 10 min, and the flow rate was set to 0.4 mL/min. A QTRAP 4500 mass spectrometer (Sciex, USA) equipped with an ESI source, was used for detection. The mass spectrometric parameters were optimized to obtain maximum sensitivity at unit resolution. The ion source temperature was maintained at 450 °C, and the ion spray voltage was set to 5500 V. The CUR was set to 40 psi and the collision gas (CAD) to medium. The MRM experiments were conducted in the positive ion mode by monitoring the precursor to product ion transitions from *m*/*z* 422 to 247 (CE = 25 eV) and from *m*/*z* 422 to 106 (CE = 57 eV) for compound (***R***)**-32** and from *m*/*z* 436 to 207 (CE = 42 eV) for IS.

The working solutions for **(*R*)-32** were created in methanol, while the stock solution was made in DMSO at a concentration of 1 mg/mL. Samples for the calibration curve were made ready by vortexing 45 μL of matrix (serum or brain homogenate) for 10 s after adding 5 μL of standard working solution at the following concentrations: 0.01, 0.1, 0.25, 0.5, 1, 2.5, 5, 10, 20, 50, 100, and 200 μg/mL. Two distinct calibration curves were created for serum samples, one for greater concentrations and one for lower amounts. The precipitating agent was also diluted 10 times with calibrators and samples from the highest concentration range. Plotting the ratio of the investigated compound’s peak area to IS against **(*R*)-32** concentration resulted in the construction of the calibration curves, which were then produced by weighted (1/*xx*) linear regression analysis. The limits used for quantification were 0.001 to 5, 0.1 to 20 μg/mL for serum, and 0.005 to 25 μg/g for brain tissue. Samples with **(*R*)-32** concentrations higher than the quantitative upper limit were 10-fold diluted using the blank matrix, which is either serum or brain homogenate. The method’s precision and accuracy fell within the bounds established by the FDA’s standards for the verification of bioanalytical techniques. During the normal analysis of the samples, no substantial matrix effect was identified, and no stability issues were encountered. The Analyst program version 1.7 was utilized for both data collecting and analysis.

#### Pharmacokinetic Data Analysis

Noncompartmental analysis was used to estimate pharmacokinetic parameters of **(*R*)-32** in serum and brain tissue. The area under the mean concentration versus time curve extrapolated to infinity (AUC_0-inf_) was estimated using the linear trapezoidal rule by combining trapezoid calculation of AUC_0–*t*_ and the area extrapolated to infinity. The MRT was calculated from AUMC_0-inf_/AUC_0-inf_, where AUMC_0-inf_ was estimated by calculation of the total area under the first-moment curve. The terminal slope (λ_*z*_) was calculated by log–linear regression of the drug concentration versus time data in the terminal phase and the terminal half-life (*t*_0.5_) was calculated as 0.693/λ_*z*_. Serum clearance (CL/F) was estimated from the dose (D) administered *i.p*. or *p.o*. (in mg per kg of body weight) divided by AUC_0-inf_. The terminal volume of distribution (V_*z*_/F) was calculated from CL/F/λ_*z*_.

### *In Vitro* ADME-Tox Studies

All assays and protocols used for the evaluation of compounds ADME-Tox parameters were described previously.^35−38,95−98^ The precoated PAMPA Plate System Gentest was utilized to estimate passive permeability (Tewksbury, MA, USA). HLMs, obtained from Sigma-Aldrich (St. Louis, MO, USA), were used for the metabolic stability assay. The most likely sites of metabolism could be identified, thanks to the MetaSite 6.0.1 program from Molecular Discovery Ltd. (Hertfordshire, UK), which was used to support the microsome experiments. The impact on recombinant human cytochromes CYP3A4, CYP2D6, and CYP2C9 was examined using CYP3A4, CYP2D6, and CYP2C9 P450-Glo kits supplied by Promega (Madison, WI, USA) in order to anticipate possible DDIs. The hepatoma HepG2 (ATCC HB-8065) cell line obtained directly from ATCC (American Type Culture Collection, Manassas, VA, USA) was used for cell-based safety testing. The vitality of the cells was assessed using the CellTiter 96 AQueous Non-Radioactive Cell Proliferation Assay (Promega, Madison, WI, USA), following a 48 h incubation period with either the reference medication, doxorubicin (DOX), or serial dilutions of the investigated compounds. Using an EnSpire PerkinElmer microplate reader (Waltham, MA, USA), the luminescent signal and absorbances (measured at 490 nm) in DDIs and safety assays were determined. The PAMPA and metabolic stability tests employed LC/MS/MS analyses, which were acquired using the Waters ACQUITY TQD system (Waters, Milford, CT, USA). Caffeine, quinidine, doxorubicin, ketoconazole, sulfaphenazole, and verapamil were the reference medications acquired from Sigma-Aldrich (St. Louis, MO, USA).

### Binding/Functional Studies

Using previously described testing methodologies, binding/functional tests were conducted on a commercial basis at Eurofins Laboratories (Poitiers, France) and Eurofins Panlabs Discovery Services Taiwan, Ltd. (New Taipei City, Taiwan); see Table S9 for more information.

Patch-clamp studies were carried out in prefrontal cortex pyramidal neurons. The methodology of slice preparation, preparation of dispersed cortical neurons, and sodium currents recording technique were the same as in our previous study.^99^ Compounds (***R*)-31** and **(*R*)-32** were tested at a concentration of 10 μM and were applied to the whole bath.

### Scanning Electron Microscopy

The morphology of the particles was analyzed by a Hitachi S-4700 (Japan) scanning electron microscope (SEM). The powder samples were adhered to a holder with double-sided copper tape. Before analysis, their surface was coated with carbon using a 208 HR carbon sputter coater (Cressington Scientific Instruments, Watford, UK). The images were taken at a magnification of 50x, 200x and 500x.

### Thermogravimetric Analysis

A Mettler Toledo thermogravimetric analysis/SDTA 851^e^ apparatus calibrated with indium, zinc, and aluminum was used. Its accuracy was equal to 10^–6^ g. The samples were placed in an open aluminum crucible. The measurements were performed in Ar 5.0 (50 mL/min). The temperature ranged from 25 to 400 °C. The constant heating rate of 10 °C/min was applied.

### Differential Scanning Calorimetry

The measurements were carried out using a Mettler Toledo DSC 3+ differential scanning calorimeter (Switzerland) with the software STARe v.16.4. The samples (ca. 5 mg) were placed in an aluminum pan sealed with a pierced lid. They were heated from 10 to 150 °C (1st heating scan), then cooled down from 150 to 10 °C, and reheated again from 10 to 150 °C (2nd heating scan). The heating rate was 5 °C/min. The measurements were carried out in Ar (50 mL/min).

## Supporting Information Available

Synthetic procedure, physicochemical and spectral data for the starting amines, antiseizure screening data, binding/functional assay information, effect of repeated pretreatment on anxiety, depressive-like behavior, and spontaneous locomotor activity in mice subjected to the PTZ kindling, *in silico* metabolic biotransformations pathways, metabolism data (ion fragment analysis and structures of probable metabolites) and ^1^H NMR, ^13^C NMR spectra for target compounds and HRMS spectra for selected molecules, thermogravimetric curve, and DSC scans (PDF)

## Notes

Safety: No unexpected or unusually high safety hazards were encountered.

## Abbreviations Used

ADME-Tox: absorption, distribution, metabolism, excretion, toxicity
ASM: antiseizure medication
BDNF: brain-derived neurotrophic factor
CBD: cannabidiol
CDI: carbonyldiimidazole
CNS: central nervous system
DCC: dicyclohexylcarbodiimide
DCM: dichloromethane
DDIs: drug–drug interactions
DRE: drug-resistant epilepsy
GABA: gamma-aminobutyric acid
DSC: differential scanning calorimetry
HLMs: human liver microsomes
HRMS: high resolution mass spectroscopy
Six Hz: six-Hertz seizure test
LC-MS: liquid chromatography-mass spectrometry
LCS: lacosamide
LEV: levetiracetam
MeCN: acetonitrile
MES: maximal electroshock seizure test
MeOH: methanol
NGF: nerve growth factor
OXPT: oxaliplatin
QD: quinidine
PAMPA: parallel artificial membrane permeability assay
PI: protective index (TD_50_/ED_50_)
PT: pretreatment time
PTZ: pentylenetetrazole
*sc*PTZ: subcutaneous pentylenetetrazole seizure test
STZ: streptozotocin
SV2A: synaptic vesicle glycoprotein 2A
TFA: trifluoroacetic acid
TGA: thermogravimetric analysis
TRPV1: transient receptor potential cation channel vanilloid type 1
UPLC: ultraperformance liquid chromatography
VPA: valproic acid

## References

[ref1] Thijs R. D.; Surges R.; O’Brien T. J.; Sander J. W. Epilepsy in Adults. Lancet 2019, 393 (10172), 689–701. 10.1016/S0140-6736(18)32596-0. 30686584

[ref2] Perucca P.; Bahlo M.; Berkovic S. F. The Genetics of Epilepsy. Annu. Rev. Genomics Hum. Genet. 2020, 21 (1), 205–230. 10.1146/annurev-genom-120219-074937. 32339036

[ref3] Tang F.; Hartz A. M. S.; Bauer B. Drug-Resistant Epilepsy: Multiple Hypotheses, Few Answers. Front. Neurol. 2017, 8, 301 10.3389/fneur.2017.00301. 28729850 PMC5498483

[ref4] Hange N.; Poudel S.; Ozair S.; Paul T.; Nambakkam M.; Shrestha R.; Greye F.; Shah S.; Raj Adhikari Y.; Thapa S.; Patel P. Managing Chronic Neuropathic Pain: Recent Advances and New Challenges. Neurol. Res. Int. 2022, 2022, 1–14. 10.1155/2022/8336561. PMC9581623 36277331

[ref5] Colloca L.; Ludman T.; Bouhassira D.; Baron R.; Dickenson A. H.; Yarnitsky D.; Freeman R.; Truini A.; Attal N.; Finnerup N. B.; Eccleston C.; Kalso E.; Bennett D. L.; Dworkin R. H.; Raja S. N. Neuropathic Pain. Nat. Rev. Dis. Primers 2017, 3 (1), 17002 10.1038/nrdp.2017.2. 28205574 PMC5371025

[ref6] Kremer M.; Salvat E.; Muller A.; Yalcin I.; Barrot M. Antidepressants and Gabapentinoids in Neuropathic Pain: Mechanistic Insights. Neuroscience 2016, 338, 183–206. 10.1016/j.neuroscience.2016.06.057. 27401055

[ref7] Johannessen Landmark C. Antiepileptic Drugs in Non-Epilepsy Disorders. CNS Drugs 2008, 22 (1), 27–47. 10.2165/00023210-200822010-00003. 18072813

[ref8] Talevi A. Multi-Target Pharmacology: Possibilities and Limitations of the “Skeleton Key Approach” from a Medicinal Chemist Perspective. Front. Pharmacol 2015, 6, 205 10.3389/fphar.2015.00205. 26441661 PMC4585027

[ref9] Bansal Y.; Silakari O. Multifunctional Compounds: Smart Molecules for Multifactorial Diseases. Eur. J. Med. Chem. 2014, 76, 31–42. 10.1016/j.ejmech.2014.01.060. 24565571

[ref10] Löscher W. Single-Target Versus Multi-Target Drugs Versus Combinations of Drugs With Multiple Targets: Preclinical and Clinical Evidence for the Treatment or Prevention of Epilepsy. Front. Pharmacol 2021, 12, 730257 10.3389/fphar.2021.730257. 34776956 PMC8580162

[ref11] Youdim M. B. H.; Kupershmidt L.; Amit T.; Weinreb O. Promises of Novel Multi-Target Neuroprotective and Neurorestorative Drugs for Parkinson’s Disease. Parkinsonism Relat. Disord. 2014, 20, S132–S136. 10.1016/S1353-8020(13)70032-4. 24262165

[ref12] Simone Tranches Dias K.; Viegas C. Multi-Target Directed Drugs: A Modern Approach for Design of New Drugs for the Treatment of Alzheimer’s Disease. Curr. Neuropharmacol. 2014, 12 (3), 239–255. 10.2174/1570159X1203140511153200. 24851088 PMC4023454

[ref13] Millan M. J. On ‘Polypharmacy’ and Multi-Target Agents, Complementary Strategies for Improving the Treatment of Depression: A Comparative Appraisal. Int. J. Neuropsychopharmacol. 2014, 17 (07), 1009–1037. 10.1017/S1461145712001496. 23719026

[ref14] Hwang H.; Wecksler A. T.; Wagner K.; Hammock B. D. Rationally Designed Multitarget Agents Against Inflammation and Pain. Curr. Med. Chem. 2013, 20 (13), 1783–1799. 10.2174/0929867311320130013. 23410172 PMC4113248

[ref15] Kucuksayan E.; Ozben T. Hybrid Compounds as Multitarget Directed Anticancer Agents. Curr. Top. Med. Chem. 2017, 17 (8), 907–918. 10.2174/1568026616666160927155515. 27697050

[ref16] Petrelli A.; Valabrega G. Multitarget Drugs: The Present and the Future of Cancer Therapy. Expert Opin. Pharmacother. 2009, 10 (4), 589–600. 10.1517/14656560902781907. 19284362

[ref17] Wood M.; Daniels V.; Provins L.; Wolff C.; Kaminski R. M.; Gillard M. Pharmacological Profile of the Novel Antiepileptic Drug Candidate Padsevonil: Interactions with Synaptic Vesicle 2 Proteins and the GABAA Receptor. J. Pharmacol. Exp. Ther. 2020, 372 (1), 1–10. 10.1124/jpet.119.261149. 31619465

[ref18] Makhoba X. H.; Viegas C. Jr.; Mosa R. A.; Viegas F. P.; Pooe O. J. Potential Impact of the Multi-Target Drug Approach in the Treatment of Some Complex Diseases. DDDT 2020, 14, 3235–3249. 10.2147/DDDT.S257494. 32884235 PMC7440888

[ref19] Bolognesi M. L. Polypharmacology in a Single Drug: Multitarget Drugs. Curr. Med. Chem. 2013, 20 (13), 1639–1645. 10.2174/0929867311320130004. 23410164

[ref20] Zhou J.; Jiang X.; He S.; Jiang H.; Feng F.; Liu W.; Qu W.; Sun H. Rational Design of Multitarget-Directed Ligands: Strategies and Emerging Paradigms. J. Med. Chem. 2019, 62 (20), 8881–8914. 10.1021/acs.jmedchem.9b00017. 31082225

[ref21] Löscher W.; Klein P. The Pharmacology and Clinical Efficacy of Antiseizure Medications: From Bromide Salts to Cenobamate and Beyond. CNS Drugs 2021, 35 (9), 935–963. 10.1007/s40263-021-00827-8. 34145528 PMC8408078

[ref22] Socała K.; Jakubiec M.; Abram M.ł; Mlost J.; Starowicz K.; Kaminski R.ł M.; Ciepiela K.; Andres-Mach M.; Zagaja M.ła.; Metcalf C. S.; Zawadzki P.ła.; Wlaz P.; Kaminski K. TRPV1 channel in the pathophysiology of epilepsy and its potential as a molecular target for the development of new antiseizure drug candidates. Prog. Neurobiol. 2024, 240, 102634 10.1016/j.pneurobio.2024.102634. 38834133

[ref23] Lee K.; Jo Y. Y.; Chung G.; Jung J. H.; Kim Y. H.; Park C.-K. Functional Importance of Transient Receptor Potential (TRP) Channels in Neurological Disorders. Front. Cell Dev. Biol. 2021, 9, 611773 10.3389/fcell.2021.611773. 33748103 PMC7969799

[ref24] Nilius B.; Owsianik G. The Transient Receptor Potential Family of Ion Channels. Genome Biol. 2011, 12 (3), 218 10.1186/gb-2011-12-3-218. 21401968 PMC3129667

[ref25] Nishida M.; Hara Y.; Yoshida T.; Inoue R.; Mori Y. TRP Channels: Molecular Diversity and Physiological Function. Microcirculation 2006, 13 (7), 535–550. 10.1080/10739680600885111. 16990213

[ref26] Ho K. W.; Ward N. J.; Calkins D. J. TRPV1: A Stress Response Protein in the Central Nervous System. Am. J. Neurodegener. Dis. 2012, 1 (1), 1–14. 22737633 PMC3560445

[ref27] Nazıroglu M. TRPV1 Channel: A Potential Drug Target for Treating Epilepsy. Curr. Neuropharmacol. 2015, 13 (2), 239–247. 10.2174/1570159X13666150216222543. 26411767 PMC4598436

[ref28] Nazıroğlu M.; Taner A. N.; Balbay E.; Çiğ B. Inhibitions of Anandamide Transport and FAAH Synthesis Decrease Apoptosis and Oxidative Stress through Inhibition of TRPV1 Channel in an in Vitro Seizure Model. Mol. Cell. Biochem. 2019, 453 (1–2), 143–155. 10.1007/s11010-018-3439-0. 30159798

[ref29] Socała K.; Jakubiec M.; Abram M.; Mlost J.; Starowicz K.; Kamiński R. M.; Ciepiela K.; Andres-Mach M.; Zagaja M.; Metcalf C. S.; Zawadzki P.; Wlaź P.; Kamiński K. TRPV1 Channel in the Pathophysiology of Epilepsy and Its Potential as a Molecular Target for the Development of New Antiseizure Drug Candidates. Prog. Neurobiol. 2024, 240, 102634 10.1016/j.pneurobio.2024.102634. 38834133

[ref30] Devinsky O.; Cilio M. R.; Cross H.; Fernandez-Ruiz J.; French J.; Hill C.; Katz R.; Di Marzo V.; Jutras-Aswad D.; Notcutt W. G.; Martinez-Orgado J.; Robson P. J.; Rohrback B. G.; Thiele E.; Whalley B.; Friedman D. Cannabidiol: Pharmacology and Potential Therapeutic Role in Epilepsy and Other Neuropsychiatric Disorders. Epilepsia 2014, 55 (6), 791–802. 10.1111/epi.12631. 24854329 PMC4707667

[ref31] Patra P. H.; Barker-Haliski M.; White H. S.; Whalley B. J.; Glyn S.; Sandhu H.; Jones N.; Bazelot M.; Williams C. M.; McNeish A. J. Cannabidiol Reduces Seizures and Associated Behavioral Comorbidities in a Range of Animal Seizure and Epilepsy Models. Epilepsia 2019, 60 (2), 303–314. 10.1111/epi.14629. 30588604 PMC6378611

[ref32] Kamiński K.; Zagaja M.; Łuszczki J. J.; Rapacz A.; Andres-Mach M.; Latacz G.; Kieć-Kononowicz K. Design, Synthesis, and Anticonvulsant Activity of New Hybrid Compounds Derived from 2-(2,5-Dioxopyrrolidin-1-yl)Propanamides and 2-(2,5-Dioxopyrrolidin-1-yl)Butanamides. J. Med. Chem. 2015, 58 (13), 5274–5286. 10.1021/acs.jmedchem.5b00578. 26052884

[ref33] Abram M.; Zagaja M.; Mogilski S.; Andres-Mach M.; Latacz G.; Baś S.; Łuszczki J. J.; Kieć-Kononowicz K.; Kamiński K. Multifunctional Hybrid Compounds Derived from 2-(2,5-Dioxopyrrolidin-1-yl)-3-Methoxypropanamides with Anticonvulsant and Antinociceptive Properties. J. Med. Chem. 2017, 60 (20), 8565–8579. 10.1021/acs.jmedchem.7b01114. 28934547

[ref34] Abram M.; Rapacz A.; Latacz G.; Szulczyk B.; Kalinowska-Tłuścik J.; Otto-Ślusarczyk D.; Struga M.; Kamiński R. M.; Kamiński K. Asymmetric Synthesis and in Vivo/in Vitro Characterization of New Hybrid Anticonvulsants Derived from (2,5-Dioxopyrrolidin-1-yl)Phenylacetamides. Bioorg. Chem. 2021, 109, 104751 10.1016/j.bioorg.2021.104751. 33647745

[ref35] Abram M.; Jakubiec M.; Rapacz A.; Mogilski S.; Latacz G.; Kamiński R. M.; Kamiński K. The Search for New Anticonvulsants in a Group of (2,5-Dioxopyrrolidin-1-yl)(Phenyl)Acetamides with Hybrid Structure—Synthesis and In Vivo/In Vitro Studies. Int. J. Mol. Sci. 2020, 21 (22), 8780 10.3390/ijms21228780. 33233618 PMC7699745

[ref36] Abram M.; Rapacz A.; Mogilski S.; Latacz G.; Lubelska A.; Kamiński R. M.; Kamiński K. Multitargeted Compounds Derived from (2,5-Dioxopyrrolidin-1-yl)(Phenyl)-Acetamides as Candidates for Effective Anticonvulsant and Antinociceptive Agents. ACS Chem. Neurosci. 2020, 11 (13), 1996–2008. 10.1021/acschemneuro.0c00257. 32479058

[ref37] Kamiński K.; Mogilski S.; Abram M.; Rapacz A.; Latacz G.; Szulczyk B.; Walczak M.; Kuś K.; Matyjaszczyk K.; Kamiński R. M. KA-104, a New Multitargeted Anticonvulsant with Potent Antinociceptive Activity in Preclinical Models. Epilepsia 2020, 61 (10), 2119–2128. 10.1111/epi.16669. 32929733

[ref38] Jakubiec M.; Abram M.; Zagaja M.; Andres-Mach M.; Szewczyk A.; Latacz G.; Szulczyk B.; Socała K.; Nieoczym D.; Wlaź P.; Metcalf C. S.; Wilcox K.; Kamiński R. M.; Kamiński K. New Phenylglycinamide Derivatives with Hybrid Structure as Candidates for New Broad-Spectrum Anticonvulsants. Cells 2022, 11 (12), 1862 10.3390/cells11121862. 35740990 PMC9221546

[ref39] Brooks W. H.; Guida W. C.; Daniel K. G. The Significance of Chirality in Drug Design and Development. Curr. Top. Med. Chem. 2011, 11 (7), 760–770. 10.2174/156802611795165098. 21291399 PMC5765859

[ref40] Abram M.; Jakubiec M.; Kamiński K. Chirality as an Important Factor for the Development of New Antiepileptic Drugs. ChemMedChem 2019, 14 (20), 1744–1761. 10.1002/cmdc.201900367. 31476107

[ref41] Wolfe J. P.; Wagaw S.; Buchwald S. L. An Improved Catalyst System for Aromatic Carbon-Nitrogen Bond Formation: The Possible Involvement of Bis(Phosphine) Palladium Complexes as Key Intermediates. J. Am. Chem. Soc. 1996, 118 (30), 7215–7216. 10.1021/ja9608306.

[ref42] Galanopoulou A. S.; Kokaia M.; Loeb J. A.; Nehlig A.; Pitkänen A.; Rogawski M. A.; Staley K. J.; Whittemore V. H.; Edward Dudek F. Epilepsy Therapy Development: Technical and Methodologic Issues in Studies with Animal Models. Epilepsia 2013, 54 (s4), 13–23. 10.1111/epi.12295. PMC3747731 23909850

[ref43] Barker-Haliski M.; Steve White H. Validated Animal Models for Antiseizure Drug (ASD) Discovery: Advantages and Potential Pitfalls in ASD Screening. Neuropharmacol. 2020, 167, 107750 10.1016/j.neuropharm.2019.107750. PMC7470169 31469995

[ref44] Löscher W. Critical Review of Current Animal Models of Seizures and Epilepsy Used in the Discovery and Development of New Antiepileptic Drugs. Seizure 2011, 20 (5), 359–368. 10.1016/j.seizure.2011.01.003. 21292505

[ref45] Barton M. E.; Klein B. D.; Wolf H. H.; Steve White H. Pharmacological Characterization of the 6 Hz Psychomotor Seizure Model of Partial Epilepsy. Epilepsy Res. 2001, 47 (3), 217–227. 10.1016/S0920-1211(01)00302-3. 11738929

[ref46] Kehne J. H.; Klein B. D.; Raeissi S.; Sharma S. The National Institute of Neurological Disorders and Stroke (NINDS) Epilepsy Therapy Screening Program (ETSP). Neurochem. Res. 2017, 42 (7), 1894–1903. 10.1007/s11064-017-2275-z. 28462454 PMC5504134

[ref47] Neuropharmacology Methods in Epilepsy Research; Peterson S. L., Albertson T. E., Eds.; CRC Press Methods in the Life Sciences Cellular and Molecular Neuropharmacology; CRC Press: Boca Raton, 1988.

[ref48] Malik A. R.; Willnow T. E. Excitatory Amino Acid Transporters in Physiology and Disorders of the Central Nervous System. Int. J. Mol. Sci. 2019, 20 (22), 5671 10.3390/ijms20225671. 31726793 PMC6888459

[ref49] Hubbard J. A.; Szu J. I.; Yonan J. M.; Binder D. K. Regulation of Astrocyte Glutamate Transporter-1 (GLT1) and Aquaporin-4 (AQP4) Expression in a Model of Epilepsy. Exp. Neurol. 2016, 283, 85–96. 10.1016/j.expneurol.2016.05.003. 27155358 PMC4992641

[ref50] Szyndler J.; Piechal A.; Blecharz-Klin K.; Skórzewska A.; Maciejak P.; Walkowiak J.; Turzyñska D.; Bidziñski A.; Płaźnik K.; Widy-Tyszkiewicz E. Effect of Kindled Seizures on Rat Behavior in Water Morris Maze Test and Amino Acid Concentrations in Brain Structures. Pharmacol. Rep. 2006, 58, 75. 16531633

[ref51] Jankowsky J. L.; Patterson P. H. The Role of Cytokines and Growth Factors in Seizures and Their Sequelae. Prog. Neurobiol. 2001, 63 (2), 125–149. 10.1016/S0301-0082(00)00022-8. 11124444

[ref52] Javaid S.; Alqahtani F.; Ashraf W.; Anjum S. M. M.; Rasool M. F.; Ahmad T.; Alasmari F.; Alasmari A. F.; Alqarni S. A.; Imran I. Tiagabine Suppresses Pentylenetetrazole-Induced Seizures in Mice and Improves Behavioral and Cognitive Parameters by Modulating BDNF/TrkB Expression and Neuroinflammatory Markers. Biomed. Pharmacother. 2023, 160, 114406 10.1016/j.biopha.2023.114406. 36791567

[ref53] Garami A.; Pakai E.; McDonald H. A.; Reilly R. M.; Gomtsyan A.; Corrigan J. J.; Pinter E.; Zhu D. X. D.; Lehto S. G.; Gavva N. R.; Kym P. R.; Romanovsky A. A. TRPV1 Antagonists That Cause Hypothermia, Instead of Hyperthermia, in Rodents: Compounds’ Pharmacological Profiles, in Vivo Targets, Thermoeffectors Recruited and Implications for Drug Development. Acta Physiol. 2018, 223 (3), e13038 10.1111/apha.13038. PMC6032921 29352512

[ref54] Garami A.; Shimansky Y. P.; Rumbus Z.; Vizin R. C. L.; Farkas N.; Hegyi J.; Szakacs Z.; Solymar M.; Csenkey A.; Chiche D. A.; Kapil R.; Kyle D. J.; Van Horn W. D.; Hegyi P.; Romanovsky A. A. Hyperthermia Induced by Transient Receptor Potential Vanilloid-1 (TRPV1) Antagonists in Human Clinical Trials: Insights from Mathematical Modeling and Meta-Analysis. Pharmacol. Ther. 2020, 208, 107474 10.1016/j.pharmthera.2020.107474. 31926897

[ref55] Gomtsyan A.; McDonald H. A.; Schmidt R. G.; Daanen J. F.; Voight E. A.; Segreti J. A.; Puttfarcken P. S.; Reilly R. M.; Kort M. E.; Dart M. J.; Kym P. R. TRPV1 Ligands with Hyperthermic, Hypothermic and No Temperature Effects in Rats. Temperature 2015, 2 (2), 297–301. 10.1080/23328940.2015.1046013. PMC4843892 27227030

[ref56] Salinas-Abarca A. B.; Avila-Rojas S. H.; Barragán-Iglesias P.; Pineda-Farias J. B.; Granados-Soto V. Formalin Injection Produces Long-Lasting Hypersensitivity with Characteristics of Neuropathic Pain. Eur. J. Pharmacol. 2017, 797, 83–93. 10.1016/j.ejphar.2017.01.018. 28095324

[ref57] López-Cano M.; Fernández-Dueñas V.; Llebaria A.; Ciruela F. Formalin Murine Model of Pain. Bio-Protocol 2017, 7 (23), e2628 10.21769/BioProtoc.2628. 34595296 PMC8438377

[ref58] Frias B.; Merighi A. Capsaicin, Nociception and Pain. Molecules 2016, 21 (6), 797 10.3390/molecules21060797. 27322240 PMC6273518

[ref59] Fornasari D. Pharmacotherapy for Neuropathic Pain: A Review. Pain Ther. 2017, 6 (S1), 25–33. 10.1007/s40122-017-0091-4. 29178034 PMC5701897

[ref60] Hama A.; Natsume T.; Ogawa S.; Higo N.; Hayashi I.; Takamatsu H. Gaps in Understanding Mechanism and Lack of Treatments: Potential Use of a Nonhuman Primate Model of Oxaliplatin-Induced Neuropathic Pain. Pain Res. Manag. 2018, 2018, 1630709 10.1155/2018/1630709. 29854035 PMC5954874

[ref61] Fumagalli G.; Monza L.; Cavaletti G.; Rigolio R.; Meregalli C. Neuroinflammatory Process Involved in Different Preclinical Models of Chemotherapy-Induced Peripheral Neuropathy. Front. Immunol. 2021, 11, 626687 10.3389/fimmu.2020.626687. 33613570 PMC7890072

[ref62] Cowan A. Buprenorphine: New Pharmacological Aspects. Int. J. Clin. Pract. Suppl. 2003, (133), 3–8. 12665117

[ref63] Ito S.; Pham V.; Matsumura S.; Katano T.; Funatsu N. Diabetic neuropathy research from mouse models to targets for treatment. NRR 2019, 14 (11), 1870 10.4103/1673-5374.259603. 31290436 PMC6676867

[ref64] Sloan G.; Shillo P.; Selvarajah D.; Wu J.; Wilkinson I. D.; Tracey I.; Anand P.; Tesfaye S. A New Look at Painful Diabetic Neuropathy. Diabetes Res. Clin. Pract. 2018, 144, 177–191. 10.1016/j.diabres.2018.08.020. 30201394

[ref65] Sałat K.; Kołaczkowski M.; Furgała A.; Rojek A.; Śniecikowska J.; Varney M. A.; Newman-Tancredi A. Antinociceptive, Antiallodynic and Antihyperalgesic Effects of the 5-HT1A Receptor Selective Agonist, NLX-112 in Mouse Models of Pain. Neuropharmacol. 2017, 125, 181–188. 10.1016/j.neuropharm.2017.07.022. 28751195

[ref66] Jolivalt C. G.; Frizzi K. E.; Guernsey L.; Marquez A.; Ochoa J.; Rodriguez M.; Calcutt N. A. Peripheral Neuropathy in Mouse Models of Diabetes. Curr. Protoc. Mouse Biol. 2016, 6 (3), 223–255. 10.1002/cpmo.11. 27584552 PMC5023323

[ref67] Nicita F.; Spalice A.; Raucci U.; Iannetti P.; Parisi P. The Possible Use of the L-Type Calcium Channel Antagonist Verapamil in Drug-Resistant Epilepsy. Expert Rev. Neurother. 2016, 16 (1), 9–15. 10.1586/14737175.2016.1121097. 26567612

[ref68] Weiergräber M.; Stephani U.; Köhling R. Voltage-Gated Calcium Channels in the Etiopathogenesis and Treatment of Absence Epilepsy. Brain Res. Rev. 2010, 62 (2), 245–271. 10.1016/j.brainresrev.2009.12.005. 20026356

[ref69] Kaplan D. I.; Isom L. L.; Petrou S. Role of Sodium Channels in Epilepsy. Cold Spring. Harb. Perspect. Med. 2016, 6 (6), a022814 10.1101/cshperspect.a022814. 27143702 PMC4888813

[ref70] Roca-Lapirot O.; Radwani H.; Aby F.; Nagy F.; Landry M.; Fossat P. Calcium Signalling through L-Type Calcium Channels: Role in Pathophysiology of Spinal Nociceptive Transmission. Br. J. Pharmacol. 2018, 175 (12), 2362–2374. 10.1111/bph.13747. 28214378 PMC5980403

[ref71] Radwani H.; Lopez-Gonzalez M. J.; Cattaert D.; Roca-Lapirot O.; Dobremez E.; Bouali-Benazzouz R.; Eiríksdóttir E.; Langel U. ¨.; Favereaux A.; Errami M.; Landry M.; Fossat P. Cav1.2 and Cav1.3 L-Type Calcium Channels Independently Control Short- and Long-Term Sensitization to Pain. J. Physiol. 2016, 594 (22), 6607–6626. 10.1113/JP272725. 27231046 PMC5108908

[ref72] Kushnarev M.; Pirvulescu I. P.; Candido K. D.; Knezevic N. N. Neuropathic Pain: Preclinical and Early Clinical Progress with Voltage-Gated Sodium Channel Blockers. Expert Opin. Inv. Drugs 2020, 29 (3), 259–271. 10.1080/13543784.2020.1728254. 32070160

[ref73] Lai J.; Hunter J. C.; Porreca F. The Role of Voltage-Gated Sodium Channels in Neuropathic Pain. Curr. Opin. Neurobiol. 2003, 13 (3), 291–297. 10.1016/S0959-4388(03)00074-6. 12850213

[ref74] Sills G. J.; Rogawski M. A. Mechanisms of Action of Currently Used Antiseizure Drugs. Neuropharmacology 2020, 168, 107966 10.1016/j.neuropharm.2020.107966. 32120063

[ref75] Muller C.; Morales P.; Reggio P. H. Cannabinoid Ligands Targeting TRP Channels. Front. Mol. Neurosci. 2019, 11, 487 10.3389/fnmol.2018.00487. 30697147 PMC6340993

[ref76] Sałat K.; Filipek B. Antinociceptive Activity of Transient Receptor Potential Channel TRPV1, TRPA1, and TRPM8 Antagonists in Neurogenic and Neuropathic Pain Models in Mice. J. Zhejiang Univ. Sci. B 2015, 16 (3), 167–178. 10.1631/jzus.B1400189. 25743118 PMC4357366

[ref77] González-Muñiz R.; Bonache M. A.; Martín-Escura C.; Gómez-Monterrey I. Recent Progress in TRPM8Modulation: An Update. Int. J. Mol. Sci. 2019, 20 (11), 2618 10.3390/ijms20112618. 31141957 PMC6600640

[ref78] Kobayashi J.; Hirasawa H.; Fujimori Y.; Nakanishi O.; Kamada N.; Ikeda T.; Yamamoto A.; Kanbe H. Identification of N-Acyl-N-Indanyl-α-Phenylglycinamides as Selective TRPM8 Antagonists Designed to Mitigate the Risk of Adverse Effects. Bioorg. Med. Chem. 2021, 30, 115903 10.1016/j.bmc.2020.115903. 33333445

[ref79] Preti D.; Saponaro G.; Szallasi A. Transient Receptor Potential Ankyrin 1 (TRPA1) Antagonists. Pharmaceutical Patent Analyst 2015, 4 (2), 75–94. 10.4155/ppa.14.60. 25853468

[ref80] de Lera Ruiz M.; Kraus R. L. Voltage-Gated Sodium Channels: Structure, Function, Pharmacology, and Clinical Indications. J. Med. Chem. 2015, 58 (18), 7093–7118. 10.1021/jm501981g. 25927480

[ref81] Barbieri R.; Nizzari M.; Zanardi I.; Pusch M.; Gavazzo P. Voltage-Gated Sodium Channel Dysfunctions in Neurological Disorders. Life 2023, 13 (5), 1191 10.3390/life13051191. 37240836 PMC10223093

[ref82] Więckowska A.; Wichur T.; Godyń J.; Bucki A.; Marcinkowska M.; Siwek A.; Więckowski K.; Zaręba P.; Knez D.; Głuch-Lutwin M.; Kazek G.; Latacz G.; Mika K.; Kołaczkowski M.; Korabecny J.; Soukup O.; Benkova M.; Kieć-Kononowicz K.; Gobec S.; Malawska B. Novel Multitarget-Directed Ligands Aiming at Symptoms and Causes of Alzheimer’s Disease. ACS Chem. Neurosci. 2018, 9 (5), 1195–1214. 10.1021/acschemneuro.8b00024. 29384656

[ref83] Toman J. E. P.; Swinyard E. A.; Goodman L. S. Properties of Maximal Seizures, and Their Alteration by Anticonvulsant Drugs and Other Agents. J. Neurophysiol. 1946, 9 (3), 231–239. 10.1152/jn.1946.9.3.231. 21028165

[ref84] Ferreri G.; Chimirri A.; Russo E.; Gitto R.; Gareri P.; De Sarro A.; De Sarro G. Comparative Anticonvulsant Activity of N-Acetyl-1-Aryl-6,7-Dimethoxy-1,2,3,4-Tetrahydroisoquinoline Derivatives in Rodents. Pharmacol., Biochem. Behav. 2004, 77 (1), 85–94. 10.1016/j.pbb.2003.09.019. 14724045

[ref85] Florek-Luszczki M.; Wlaz A.; Kondrat-Wrobel M. W.; Tutka P.; Luszczki J. J. Effects of WIN 55,212–2 (a Non-Selective Cannabinoid CB1 and CB2 Receptor Agonist) on the Protective Action of Various Classical Antiepileptic Drugs in the Mouse 6 Hz Psychomotor Seizure Model. J. Neural. Transm. 2014, 121 (7), 707–715. 10.1007/s00702-014-1173-7. 24549572 PMC4065376

[ref86] Boissier J.-R.; Tardy J.; Diverres J.-C. Une Nouvelle Méthode Simple Pour Explorer l’action ≪tranquillisante≫: Le Test de La Cheminée. PHA 1960, 3 (1), 81–84. 10.1159/000134913.

[ref87] Socała K.; Nieoczym D.; Pieróg M.; Wyska E.; Szafarz M.; Doboszewska U.; Wlaź P. Effect of Tadalafil on Seizure Threshold and Activity of Antiepileptic Drugs in Three Acute Seizure Tests in Mice. Neurotox Res. 2018, 34 (3), 333–346. 10.1007/s12640-018-9876-4. 29427285 PMC6154210

[ref88] Socała K.; Mogilski S.; Pieróg M.; Nieoczym D.; Abram M.; Szulczyk B.; Lubelska A.; Latacz G.; Doboszewska U.; Wlaź P.; Kamiński K. KA-11, a Novel Pyrrolidine-2,5-Dione Derived Broad-Spectrum Anticonvulsant: Its Antiepileptogenic, Antinociceptive Properties and in Vitro Characterization. ACS Chem. Neurosci. 2019, 10 (1), 636–648. 10.1021/acschemneuro.8b00476. 30247871

[ref89] Kamiński K.; Socała K.; Zagaja M.; Andres-Mach M.; Abram M.; Jakubiec M.; Pieróg M.; Nieoczym D.; Rapacz A.; Gawel K.; Esguerra C. V.; Latacz G.; Lubelska A.; Szulczyk B.; Szewczyk A.; Łuszczki J. J.; Wlaź P. N-Benzyl-(2,5-Dioxopyrrolidin-1-yl)Propanamide (AS-1) with Hybrid Structure as a Candidate for a Broad-Spectrum Antiepileptic Drug. Neurotherapeutics 2020, 17 (1), 309–328. 10.1007/s13311-019-00773-w. 31486023 PMC7007424

[ref90] West P. J.; Saunders G. W.; Billingsley P.; Smith M. D.; White H. S.; Metcalf C. S.; Wilcox K. S. Recurrent Epileptiform Discharges in the Medial Entorhinal Cortex of Kainate-Treated Rats Are Differentially Sensitive to Antiseizure Drugs. Epilepsia 2018, 59 (11), 2035–2048. 10.1111/epi.14563. 30328622 PMC6215509

[ref91] Beirith A.; Santos A. R. S.; Rodrigues A. L. S.; Creczynski-Pasa T. B.; Calixto J. B. Spinal and Supraspinal Antinociceptive Action of Dipyrone in Formalin, Capsaicin and Glutamate Tests. Study of the Mechanism of Action. Eur. J. Pharmacol. 1998, 345 (3), 233–245. 10.1016/S0014-2999(98)00026-0. 9592021

[ref92] Mogilski S.; Kubacka M.; Redzicka A.; Kazek G.; Dudek M.; Malinka W.; Filipek B. Antinociceptive, Anti-Inflammatory and Smooth Muscle Relaxant Activities of the Pyrrolo[3,4-d]Pyridazinone Derivatives: Possible Mechanisms of Action. Pharmacol., Biochem. Behav. 2015, 133, 99–110. 10.1016/j.pbb.2015.03.019. 25847619

[ref93] Sałat K.; Cios A.; Wyska E.; Sałat R.; Mogilski S.; Filipek B.; Więckowski K.; Malawska B. Antiallodynic and Antihyperalgesic Activity of 3-[4-(3-Trifluoromethyl-Phenyl)-Piperazin-1-yl]-Dihydrofuran-2-One Compared to Pregabalin in Chemotherapy-Induced Neuropathic Pain in Mice. Pharmacol., Biochem. Behav. 2014, 122, 173–181. 10.1016/j.pbb.2014.03.025. 24726707

[ref94] Furman B. L. Streptozotocin-Induced Diabetic Models in Mice and Rats. Curr. Protoc. 2021, 1 (4), e78 10.1002/cpz1.78. 33905609

[ref95] Abram M.; Jakubiec M.; Rapacz A.; Mogilski S.; Latacz G.; Szulczyk B.; Szafarz M.; Socała K.; Nieoczym D.; Wyska E.; Wlaź P.; Kamiński R. M.; Kamiński K. Identification of New Compounds with Anticonvulsant and Antinociceptive Properties in a Group of 3-Substituted (2,5-Dioxo-Pyrrolidin-1-yl)(Phenyl)-Acetamides. Int. J. Mol. Sci. 2021, 22 (23), 13092 10.3390/ijms222313092. 34884898 PMC8658016

[ref96] Latacz G.; Lubelska A.; Jastrzębska-Więsek M.; Partyka A.; Marć M. A.; Satała G.; Wilczyńska D.; Kotańska M.; Więcek M.; Kamińska K.; Wesołowska A.; Kieć-Kononowicz K.; Handzlik J. The 1,3,5-Triazine Derivatives as Innovative Chemical Family of 5-HT6 Serotonin Receptor Agents with Therapeutic Perspectives for Cognitive Impairment. Int. J. Mol. Sci. 2019, 20 (14), 3420 10.3390/ijms20143420. 31336820 PMC6678253

[ref97] Ali W.; Więcek M.; Łażewska D.; Kurczab R.; Jastrzębska-Więsek M.; Satała G.; Kucwaj-Brysz K.; Lubelska A.; Głuch-Lutwin M.; Mordyl B.; Siwek A.; Nasim M. J.; Partyka A.; Sudoł S.; Latacz G.; Wesołowska A.; Kieć-Kononowicz K.; Handzlik J. Synthesis and Computer-Aided SAR Studies for Derivatives of Phenoxyalkyl-1,3,5-Triazine as the New Potent Ligands for Serotonin Receptors 5-HT6. Eur. J. Med. Chem. 2019, 178, 740–751. 10.1016/j.ejmech.2019.06.022. 31229876

[ref98] Lubelska A.; Latacz G.; Jastrzębska-Więsek M.; Kotańska M.; Kurczab R.; Partyka A.; Marć M. A.; Wilczyńska D.; Doroz-Płonka A.; Łażewska D.; Wesołowska A.; Kieć-Kononowicz K.; Handzlik J. Are the Hydantoin-1,3,5-Triazine 5-HT6R Ligands a Hope to a Find New Procognitive and Anti-Obesity Drug? Considerations Based on Primary In Vivo Assays and ADME-Tox Profile In Vitro. Molecules 2019, 24 (24), 4472 10.3390/molecules24244472. 31817628 PMC6943527

[ref99] Szulczyk B.; Spyrka A. Menthol Exerts TRPM8-Independent Antiepileptic Effects in Prefrontal Cortex Pyramidal Neurons. Brain Res. 2022, 1783, 147847 10.1016/j.brainres.2022.147847. 35227652

