# Supplementary material for: Discovery and Profiling of New Multimodal Phenylglycinamide Derivatives as Potent Antiseizure and Antinociceptive Drug Candidates
Source: ACS Chem Neurosci. 2024 Aug 21;15(17):3228–56. doi: 10.1021/acschemneuro.4c00438 (PMC11378297; doi:10.1021/acschemneuro.4c00438)
Supplement: Supplementary file 1 — cn4c00438_si_001.pdf [file cn4c00438_si_001.pdf]

Supplementary material for

Discovery and profiling of new multimodal phenylglycinamide derivatives as potent antiseizure and antinociceptive drug candidates

Marcin Jakubiec [†], Michał Abram [†], Mirosław Zagaja [‡], Katarzyna Socała [⁑], Vanja Panic [#], Gniewomir Latacz [▲], Szczepan Mogilski [⊥], Małgorzata Szafarz [◊], Joanna Szala-Rycaj [‡], Jerry Saunders [#], Peter J. West [#], Dorota Nieoczym [⁑], Katarzyna Przejczowska-Pomierny [◊], Bartłomiej Szulczyk [♦], Anna Krupa [■], Elżbieta Wyska [◊], Piotr Wlaź [⁑], Cameron S. Metcalf [#], Karen Wilcox [#], Marta Andres-Mach [‡], Rafał M. Kamiński [†], and Krzysztof Kamiński [†,*]

[†] Department of Medicinal Chemistry, Faculty of Pharmacy, Jagiellonian University Medical College, Medyczna 9, 30-688 Cracow, Poland
[‡] Department of Experimental Pharmacology, Institute of Rural Health, Jaczewskiego 2, 20-950 Lublin, Poland
[⁑] Department of Animal Physiology and Pharmacology, Institute of Biological Sciences, Faculty of Biology and Biotechnology, Maria Curie-Skłodowska University, Akademicka 19, 20-033 Lublin, Poland
[#] Department of Pharmacology and Toxicology, University of Utah, Salt Lake City, UT 84112, USA
[▲] Department of Technology and Biotechnology of Drugs, Faculty of Pharmacy, Jagiellonian University Medical College, Medyczna 9, 30-688 Cracow, Poland
[⊥] Department Pharmacodynamics, Faculty of Pharmacy, Jagiellonian University Medical College, Medyczna 9, 30-688 Cracow, Poland
[◊] Department of Pharmacokinetics and Physical Pharmacy, Faculty of Pharmacy, Jagiellonian University Medical College, Medyczna 9, 30-688 Cracow, Poland
[♦] Chair and Department of Pharmacotherapy and Pharmaceutical Care, Centre for Preclinical Research and Technology, Medical University of Warsaw, Banacha 1B, 02-097 Warsaw, Poland
[■] Department of Pharmaceutical Technology and Biopharmaceutics, Jagiellonian University Medical College, Medyczna 9, 30-688 Cracow, Poland

*__Corresponding author__:

Krzysztof Kamiński, Jagiellonian University Medical College, Faculty of Pharmacy, Department of Medicinal Chemistry, Medyczna 9, 30-688 Krakow, Poland.

E-mail: k.kaminski@uj.edu.pl

Table of contents

The procedure for synthesis, physicochemical and spectra data for **A1-A8.** 3

Scheme S1. Synthesis of Boc-protected intermediates **(A1-A4)** and 1-phenylpyrrolidin-3-amine derivatives **A5–A8.** 4

Table S1. Antiseizure activity screening data for compounds **3–12**, **21–24**, **(*R*)-31–(*R*)-33**, and **(*S*)-31–(*S*)-33** in mice *i.p.* 5

Table S2. In vitro TRPV1 channel antagonist activity for compounds **3–12** (series A), **21–24** (series B) in concentration of 100 µM. 6

Table S3. *In vitro* binding and functional assays for CBD. 6

Table S4. Effect of the **(*R*)-32** on subunit $Nav_{1.1}$. 7

Table S5. Effect of the **(*R*)-32** on subunit $Nav_{1.2.}$ 8

Table S6. Effect of the **(*R*)-32** on subunit $Nav_{1.3}$. 8

Table S7. Effect of the **(*R*)-32** on subunit $Nav_{1.6}$. 9

Table S8. Effect of the **(*R*)-32** on subunit $Nav_{1.7}$. 9

Table S9. Radioligand binding and functional assays. 10

Figure S1. Thermogravimetric curve (10 °C/min) of **(*R*)-32.** 11

Figure S2. Heating (5 °C/min) DSC scans of **(*R*)-32.** 12

Figure S3. Effects of **(*R*)-31, (*R*)-32,** and **(*R*)-33** on neuromuscular strength in mice. 13

Figure S4. Effects of repeated treatment with **(*R*)-32** on anxiety, depressive-like behavior, and spontaneous locomotor activity in mice subjected to the PTZ kindling 13

Figure S5. Acute effects of BCTC**, (*R*)-31, (*R*)-32, and (*R*)-33** on rectal temperature in the capsaicin-induced test in mice. 14

Figure S6. Mean serum and brain concentrations (± SD) for **(*R*)-32** after *i.p*. administration of this compound at a dose of 25 mg/kg and 50 mg/kg. 15

Figure S7. Mean serum and brain concentrations (± SD) for **(*R*)-32** after *p.o.* administration of this compound at a dose of 25 mg/kg. 15

Figure S8. UPLC spectra after 120 min incubation of compound **(*R*)-31** with human liver microsomes in TRIS buffer pH=7.4 at 37ºC 16

Figure S9. MS ion fragment analyses and the most probable structure of **(*R*)-31** metabolite M1 16

Figure S10. MS ion fragment analyses and the most probable structure of **(*R*)-31** metabolite M2 17

Figure S11. MS ion fragment analyses and the most probable structure of **(*R*)-31** metabolite M3 17

Figure S12. UPLC spectra after 120 min incubation of compound **(*R*)-32** with human liver microsomes in TRIS buffer pH=7.4 at 37ºC 17

Figure S13. MS ion fragment analyses and the most probable structure of **(*R*)-32** metabolite M1 18

Figure S14. MS ion fragment analyses and the most probable structure of **(*R*)-32** metabolite M2 18

Figure S15. UPLC spectra after 120 min incubation of compound **(*R*)-33** with human liver microsomes in TRIS buffer pH=7.4 at 37ºC. 19

Figure S16. MS ion fragment analyses and the most probable structure of **(*R*)-33** metabolite M1 19

Figure S17. MS ion fragment analyses and the most probable structure of **(*R*)-33** metabolite M2 20

Figure S18. MS ion fragment analyses and the most probable structure of (***R*)-33** metabolite M3 20

References: 21

HMRS traces for selected final compounds 22

^{1}H NMR and ^{13}C NMR spectra for the final compounds 28

The procedure for synthesis, physicochemical and spectra data for A1-A8.

General method for the preparation of intermediates A1–A4. The starting (non-commercial) Boc-derivatives of 1-phenylpyrrolidin-3-amine were obtained by the *N*-arylation reaction. Appropriate aryl bromide (10 mmol, 1 eq), Pd_2dba_3 (0.37 g, 0.4 mmol, 0.04 eq), BINAP (0.37 g, 0.6 mmol, 0.06 eq), caesium carbonate (3.26 g, 10 mmol, 1 eq), and pyrrolidin-3-amine (3.74 g, 20 mmol, 2 eq) were suspended under an inert gas (nitrogen) atmosphere in 50 mL of dry toluene. The reaction mixture was refluxed for 12 h, subsequently cooled, and filtered through Celite 545 Merck (Darmstadt, Germany). The organic layer was washed with water and next with brine, dried over anhydrous Na_2SO_4, and finally concentrated in vacuo. The Boc protected intermediates **A1**–**A4** were purified by column chromatography using the following developing system S_1.

***Tert*-butyl (1-phenylpyrrolidin-3-yl)carbamate (A1)** Yellow oil, yield 66% (1.73 g); TLC: R_f = 0.76 (S_1); UPLC (purity 96.6%): t_R = 7.38 min. LC-MS (ESI): *m/z* calcd for $C_{15}H_{22}N_2O_2$ $(M+H)^+$ 263.17, found 263.2.

***Tert*-butyl (1-(3-(trifluoromethyl)phenyl)pyrrolidin-3-yl)carbamate (A2)** Yellow oil, yield 62% (2.05 g); TLC: R_f = 0.81 (S_1); UPLC (purity 95.3%): t_R = 8.20 min. LC-MS (ESI): *m/z* calcd for $C_{16}H_{21}F_3N_2O_2$ $(M+H)^+$ 331.16, found 331.3.

***Tert*-butyl (1-(3-(trifluoromethoxy)phenyl)pyrrolidin-3-yl)carbamate (A3)** Yellow oil, yield 68% (2.35 g); TLC: R_f = 0.83 (S_1); UPLC (purity 97.8%): t_R = 8.39 min. LC-MS (ESI): *m/z* calcd for $C_{16}H_{21}F_3N_2O_3$ $(M+H)^+$ 347.15, found 347.3.

***Tert*-butyl (1-(3-((trifluoromethyl)thio)phenyl)pyrrolidin-3-yl)carbamate (A4)** Yellow oil, yield 63% (2.28 g); TLC: R_f = 0.84 (S_1); UPLC (purity 95.1%): t_R = 8.78 min. LC-MS (ESI): *m/z* calcd for $C_{16}H_{21}F_3N_2O_2S$ $(M+H)^+$ 363.13, found 363.2.

General method for the preparation of starting amines A5–A8. The DCM (5 mL) solution of **A1–A4** (5 mmol, 1 eq) was treated with TFA (1.71 g, 15 mmol, 3 eq) and stirred at room temperature for 3 h. Afterwards, the organic solvents were evaporated to dryness. The resulting oil residue was dissolved in water (20 mL), and then 25% ammonium hydroxide was carefully added to pH = 8. The aqueous layer was extracted with DCM (3 × 20 mL), dried over Na_2SO_4, and concentrated to give the **A5–A8** as yellow or bronze oils. Starting amines **A5–A8** were advanced as substrates for the next reactions without additional purification.

1-phenylpyrrolidin-3-amine (A5) Yellow oil, yield 97% (0.81 g); TLC: R_f = 0.40 (S_2); UPLC (purity 98.3%): t_R = 2.73 min. LC-MS (ESI): *m/z* calcd for $C_{10}H_{14}N_2$ $(M+H)^+$ 163.12, found 163.3. ^1H NMR (500 MHz, $CDCl_3$) δ 1.46–1.93 (m, 3 H, pyrrolidin-3-amine), 2.14–2.33 (m, 1 H, pyrrolidin-3-amine), 3.01 (dd, *J*=9.3, 4.7 Hz, 1 H, pyrrolidin-3-amine), 3.22–3.80 (m, 4 H, pyrrolidin-3-amine), 6.43–6.60 (m, 2 H, ArH), 6.64–6.74 (m, 1 H, ArH), 7.07–7.37 (m, 2 H, ArH).

1-(3-(trifluoromethyl)phenyl)pyrrolidin-3-amine (A6) Yellow oil, yield 98% (1.11 g); TLC: R_f = 0.44 (S_2); UPLC (purity 96.8%): t_R = 4.06 min. LC-MS (ESI): *m/z* calcd for $C_{11}H_{13}F_3N_2$ $(M+H)^+$ 231.11, found 231.2. ^1H NMR (500 MHz, $CDCl_3$) δ 1.63–2.05 (m, 3 H, pyrrolidin-3-amine), 2.13–2.38 (m, 1 H, pyrrolidin-3-amine), 3.04 (dd, *J*=9.5, 4.6 Hz, 1 H, pyrrolidin-3-amine), 3.25–3.40 (m, 1 H, pyrrolidin-3-amine), 3.43–3.59 (m, 2 H, pyrrolidin-3-amine), 3.67–3.81 (m, 1 H, pyrrolidin-3-amine), 6.51–6.80 (m, 2 H, ArH), 6.84–6.95 (m, 1 H, ArH), 7.16–7.38 (m, 1 H, ArH).

1-(3-(trifluoromethoxy)phenyl)pyrrolidin-3-amine (A7) Bronze oil, yield 96% (1.18 g); TLC: R_f = 0.46 (S_2); UPLC (purity 97.7%): t_R = 4.27 min. LC-MS (ESI): *m/z* calcd for $C_{11}H_{13}F_3N_2O$ $(M+H)^+$ 247.10, found 247.1. ^1H NMR (500 MHz, $CDCl_3$) δ 1.70–2.39 (m, 4 H, pyrrolidin-3-amine), 2.88–3.12 (m, 1 H, pyrrolidin-3-amine), 3.19–3.81 (m, 4 H, pyrrolidin-3-amine), 6.20–6.64 (m, 3 H, ArH), 6.93–7.22 (m, 1 H, ArH).

1-(3-((trifluoromethyl)thio)phenyl)pyrrolidin-3-amine (A8) Bronze oil, yield 97% (1.18 g); TLC: R_f = 0.47 (S_2); UPLC (purity 96.2%): t_R = 4.63 min. LC-MS (ESI): *m/z* calcd for $C_{11}H_{13}F_3N_2S$ $(M+H)^+$ 263.08, found 263.1. ^1H NMR (500 MHz, $CDCl_3$) δ 1.49–2.03 (m, 3 H, pyrrolidin-3-amine), 2.07–2.42 (m, 1 H, pyrrolidin-3-amine), 2.93–3.80 (m, 5 H, pyrrolidin-3-amine), 6.61 (dd, *J*=8.3, 2.3 Hz, 1 H, ArH), 6.76 (s, 1 H, ArH), 6.91 (d, *J*=7.5 Hz, 1 H, ArH), 7.14–7.33 (m, 1 H, ArH).

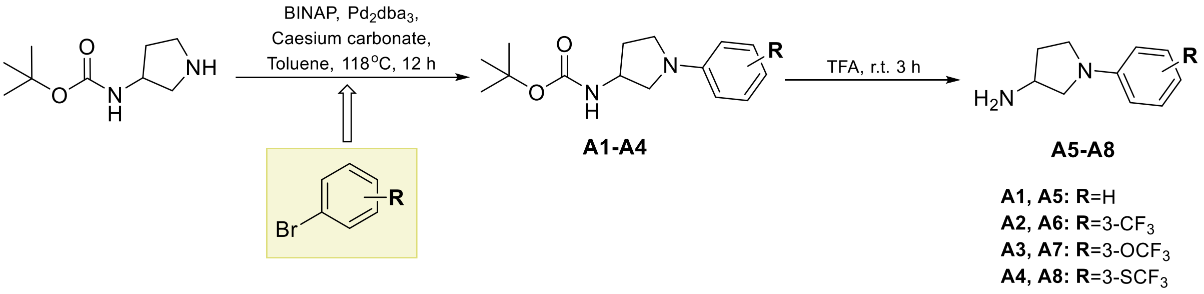

Scheme S1. Synthesis of Boc-protected intermediates (**A1**-**A4**) and 1-phenylpyrrolidin-3-amine derivatives **A5**–**A8**.

Table S1. Antiseizure activity screening data for compounds **3**–**12**, **21**–**24**, **(*R*)-31–(*R*)-33**, and **(*S*)-31**–**(*S*)-33** in mice *i.p.*

Cmpd	MES[a]	6 Hz (32 mA)[b]	*sc*PTZ[c]
**3**	0/4	2/4	-
**4**	0/4	0/4	-
**5**	0/4	2/4	-
**6**	0/4	1/4	-
**7**	0/4	0/4	-
**8**	0/4	0/4	-
**9**	0/4	0/4	-
**10**	0/4	2/4	-
**11**	0/4	2/4	-
**12**	0/4	2/4	-
**21**	0/4	0/4	
**22**	0/4	2/4	-
**23**	0/4	2/4	-
**24**	0/4	1/4	-
**(*R*)-31**	**4/4**	**4/4**	1/4
**(*S*)-31**	1/4	2/4	-
**(*R*)-32**	**4/4**	**4/4**	0/4
**(*S*)-32**	1/4	3/4	-
**(*R*)-33**	**4/4**	**4/4**	0/4
**(*S*)-33**	1/4	3/4	-

Data indicate number of mice protected/number of mice tested. **The data for the most potent compounds (4/4) have been bolded for better visualization.** Dose of 100 mg/kg was administered *i.p.* The animals were examined at 0.5 h. A dash indicates not tested. [a] MES – maximal electroshock seizure test; [b] 6 Hz – psychomotor seizure test (32 mA); [c] *sc*PTZ – subcutaneous pentylenetetrazole seizure test.

Table S2. In vitro TRPV1 channel antagonist activity for compounds **3**–**12** (series A), **21**–**24** (series B) in concentration of 100 μM.

TRPV1 (VR1) (*h*) (antagonist effect)*			
Cmpd	% Inhibition of control agonist response[a]	Cmpd	% Inhibition of control agonist response[a]
**3**	11.4	**10**	-3.5
**4**	14.4	**11**	-4.2
**5**	3.8	**12**	-1.2
**6**	6.8	**21**	4.2
**7**	-2.6	**22**	-8.4
**8**	-6.1	**23**	-2.7
**9**	6.2	**24**	1.3

*Source: human recombinant CHO cells. [a] **Results showing activity higher than 50% are considered to represent significant effects of the te st compounds**; results showing an inhibition between 25% and 50% are indicative of weak effect; results showing an inhibition lower than 25% are not considered significant and mostly attributable to variability of the sign al around the control level. Assays were performed commercially in Eurofins Laboratories (Poitiers, France).

Table S3. *In vitro* binding and functional assays for CBD.

**Binding studies**	Source	% Inhibition of control specific binding (concentration [μM])[a]
Na^+ channel (site 2)	Rat cerebral cortex	94.9 (100)
$Cav_{1.2}$ (dihydropyridine site, antagonist radioligand)	Rat cerebral cortex	58.0 (100)
**Functional studies**	Source	% Inhibition of control specific binding (concentration [μM])[a]
TRPV1 (VR1) (*h*) (antagonist effect)	Human recombinant CHO cells	47.8 (100)
CB1 (*h*) (agonist effect)	Human recombinant CHO cells	102.9 (100)

Human sodium ion channels cell based automated patch clamp assay

Human sodium ion channels cell based automated patch clamp assays: sodium channels exist in, a) a resting or closed state at -120 mV, b) a transient open state that inactivates to c) inactivated state at -10 mV. Sodium current inhibition within 1-2 ms of channel opening at 0 mV is open channel inhibition (pulse 1). In order to completely inactivate the sodium channels and facilitate inactivated state-dependent binding of the drug, the channels were kept at open state (-10 mV) longer (pulsed for 500 ms) and then stepped back to -120 mV for 10 ms to recover from inactivation into resting or closed state (but the channels that had drug bound to them will not recover from inactivation and will not open) before stepping to -10 mV for 50 ms (pulse 2) to measure sodium channels that are available to open. The higher inhibition seen at pulse 2 is due to inactivated state-dependent inhibition. Pulse 1 and 2 are used to investigate drug binding to the open state and inactivated state of the Na channels, respectively. Each concentration of compound is applied for 5 minutes. The results obtained for **(*R*)-32** are summerized in **Tables S4–S8**.

Table S4. Effect of the **(*R*)-32** on subunit Nav$_{1.1}$.

**Voltage-Gated Sodium channel *h*Nav$_{1.1}$**					
Compound	Concentration (µM)	Measurement	% Inhibition		
			n1	n2	Mean
Vehicle	0.3% DMSO	Pulse 1	0.39	-1.57	-0.59
	0.3% DMSO	Pulse 1	-0.36	0.52	0.08
	0.3% DMSO	Pulse 1	6.58	2.87	4.73
**(*R*)-32**	1	Pulse 1	2.91	0.31	1.61
	10	Pulse 1	-0.28	2.68	1.20
	100	Pulse 1	24.03	38.00	31.02
Tetracaine (positive control)	3	Pulse 1	4.33	2.23	3.28
	30	Pulse 1	20.53	11.91	16.22
	300	Pulse 1	90.95	88.36	89.66
Vehicle	0.3% DMSO	Pulse 2	-0.54	-3.48	-2.01
	0.3% DMSO	Pulse 2	1.47	-2.06	-0.30
	0.3% DMSO	Pulse 2	5.95	0.14	3.04
**(*R*)-32**	1	Pulse 2	1.74	2.11	1.92
	10	Pulse 2	45.28	38.56	41.92
	**100**	**Pulse 2**	**97.88**	**97.38**	**97.63**
Tetracaine (positive control)	3	Pulse 2	58.30	51.45	54.88
	30	Pulse 2	96.61	94.78	95.70
	300	Pulse 2	101.71	100.33	101.02

Data interpretation: Results showing inhibition greater than 50% are considered to represent significant effects of test compound and have been bolded for **(*R*)-32** for better visualization. The studies were carried out in Eurofins Discovery (St. Charles, MO, USA).

Table S5. Effect of the **(*R*)-32** on subunit $Nav_{1.2.}$

**Voltage-Gated Sodium channel *h*$Nav_{1.2}$**					
Compound	Concentration (μM)	Measurement	% Inhibition		
			n1	n2	Mean
Vehicle	0.3% DMSO	Pulse 1	1.37	2.69	2.03
	0.3% DMSO	Pulse 1	-0.82	5.93	2.55
	0.3% DMSO	Pulse 1	5.51	7.33	6.42
**(*R*)-32**	1	Pulse 1	1.86	-0.29	0.79
	10	Pulse 1	2.03	5.18	3.60
	100	Pulse 1	35.70	31.27	33.49
Tetracaine (positive control)	3	Pulse 1	5.99	-11.27	-2.64
	30	Pulse 1	19.01	8.92	13.96
	300	Pulse 1	64.90	75.12	70.01
Vehicle	0.3% DMSO	Pulse 2	-1.58	0.85	-0.37
	0.3% DMSO	Pulse 2	-4.67	3.01	-0.83
	0.3% DMSO	Pulse 2	0.13	3.70	1.92
**(*R*)-32**	1	Pulse 2	1.90	1.33	1.62
	10	Pulse 2	45.49	38.23	41.86
	**100**	**Pulse 2**	**100.47**	**100.13**	**100.30**
Tetracaine (positive control)	3	Pulse 2	55.23	54.66	54.94
	30	Pulse 2	98.84	100.35	99.59
	300	Pulse 2	99.88	101.47	100.67

Data interpretation: Results showing inhibition greater than 50% are considered to represent significant effects of test compound and have been bolded for **(*R*)-32** for better visualization. The studies were carried out in Eurofins Discovery (St. Charles, MO, USA).

Table S6. Effect of the **(*R*)-32** on subunit $Nav_{1.3}$.

**Voltage-Gated Sodium channel *h*$Nav_{1.3}$**					
Compound	Concentration (μM)	Measurement	% Inhibition		
			n1	n2	Mean
Vehicle	0.3% DMSO	Pulse 1	6.09	-2.52	5.74
	0.3% DMSO	Pulse 1	6.88	-3.27	4.41
	0.3% DMSO	Pulse 1	4.32	-5.60	-3.55
**(*R*)-32**	1	Pulse 1	-7.33	0.56	-3.38
	10	Pulse 1	-8.46	2.11	-3.18
	100	Pulse 1	5.68	7.82	6.75
Tetracaine (positive control)	3	Pulse 1	3.41	-1.92	0.75
	30	Pulse 1	10.26	9.44	9.85
	300	Pulse 1	81.81	87.85	84.83
Vehicle	0.3% DMSO	Pulse 2	4.71	-4.30	0.2
	0.3% DMSO	Pulse 2	4.97	-5.80	-0.42
	0.3% DMSO	Pulse 2	5.71	-4.15	-0.78
**(*R*)-32**	1	Pulse 2	-5.59	0.03	-2.78
	10	Pulse 2	42.11	36.99	39.55
	**100**	**Pulse 2**	**99.58**	**98.58**	**99.08**
Tetracaine (positive control)	3	Pulse 2	56.07	56.13	56.10
	30	Pulse 2	98.87	101.13	100.00
	300	Pulse 2	100.65	101.53	101.09

Data interpretation: Results showing inhibition greater than 50% are considered to represent significant effects of test compound and have been bolded for **(*R*)-32** for better visualization. The studies were carried out in Eurofins Discovery (St. Charles, MO, USA).

Table S7. Effect of the ***(R)*-32** on subunit $Nav_{1.6}$.

Voltage-Gated Sodium channel ***h*Nav$_{1.6}$**					
Compound	Concentration (µM)	Measurement	% Inhibition		
			n1	n2	Mean
Vehicle	0.3% DMSO	Pulse 1	4.62	-2.79	0.91
	0.3% DMSO	Pulse 1	14.21	-4.60	4.80
	0.3% DMSO	Pulse 1	-1.73	-2.11	-1.92
**(*R*)-32**	1	Pulse 1	-8.40	-1.89	-5.14
	10	Pulse 1	-14.40	-7.25	-10.83
	100	Pulse 1	-1.89	1.68	-0.11
Tetracaine (positive control)	3	Pulse 1	7.73	1.85	4.79
	30	Pulse 1	34.74	34.70	34.72
	300	Pulse 1	68.16	81.78	74.97
Vehicle	0.3% DMSO	Pulse 2	6.15	-0.72	2.71
	0.3% DMSO	Pulse 2	11.50	-13.99	-1.25
	0.3% DMSO	Pulse 2	-5.52	-10.09	-7.81
**(*R*)-32**	1	Pulse 2	-10.11	0.93	-4.59
	10	Pulse 2	52.98	26.87	39.92
	**100**	**Pulse 2**	**120.83**	**95.15**	**107.99**
Tetracaine (positive control)	3	Pulse 2	80.83	91.92	86.37
	30	Pulse 2	95.67	102.76	99.21
	300	Pulse 2	91.34	104.45	97.90

Data interpretation: Results showing inhibition greater than 50% are considered to represent significant effects of test compound and have been bolded for **(*R*)-32** for better visualization. The studies were carried out in Eurofins Discovery (St. Charles, MO, USA).

Table S8. Effect of the ***(R)*-32** on subunit $Nav_{1.7}$.

Voltage-Gated Sodium channel ***h*Nav$_{1.7}$**					
Compound	Concentration (µM)	Measurement	% Inhibition		
			n1	n2	Mean
Vehicle	0.3% DMSO	Pulse 1	0.32	2.71	1.51
	0.3% DMSO	Pulse 1	2.20	1.00	1.60
	0.3% DMSO	Pulse 1	6.82	3.40	5.11
**(*R*)-32**	1	Pulse 1	-4.13	-7.35	-5.74
	10	Pulse 1	-0.03	12.33	6.15
	100	Pulse 1	19.16	26.54	22.85
Tetracaine (positive control)	3	Pulse 1	11.28	6.36	8.82
	30	Pulse 1	27.51	27.76	27.64
	300	Pulse 1	94.64	95.60	95.12
Vehicle	0.3% DMSO	Pulse 2	2.17	5.01	3.59
	0.3% DMSO	Pulse 2	3.91	6.53	5.22
	0.3% DMSO	Pulse 2	8.82	10.36	9.59
**(*R*)-32**	1	Pulse 2	-3.09	-3.07	-3.08
	10	Pulse 2	35.29	40.36	37.82
	**100**	**Pulse 2**	**98.72**	**98.01**	**98.37**
Tetracaine (positive control)	3	Pulse 2	45.94	49.13	47.53
	30	Pulse 2	98.03	99.25	98.64
	300	Pulse 2	101.15	101.81	101.48

Data interpretation: Results showing inhibition greater than 50% are considered to represent significant effects of test compound and have been bolded for **(*R*)-32** for better visualization. The studies were carried out in Eurofins Discovery (St. Charles, MO, USA).

Table S9. Radioligand binding and functional assays.

**Binding studies**	
Na^+ channel (site 2)	[1]
**Functional studies**	
$Cav_{1.2}$ (L-type) *(h)* calcium ion channel cell-based antagonist calcium flux assay	[2,3]
TRPV1 (VR1) *(h)* (antagonist effect)	[4]
TRPA1 (*h*) transient potential ion channel cell based antagonist calcium flux assay	[5]
TRPM8 (*h*) (antagonist effect)	[6]
$Cav_{1.2}$ (L-type) (dihydropyridine site, antagonist radioligand)	[7]
CB1 (*h*) (agonist effect)	[8]

Assays were performed commercially in Eurofins Laboratories (Poitiers, France) or Eurofins Panlabs Discovery Services Taiwan, Ltd. (New Taipei City, Taiwan).

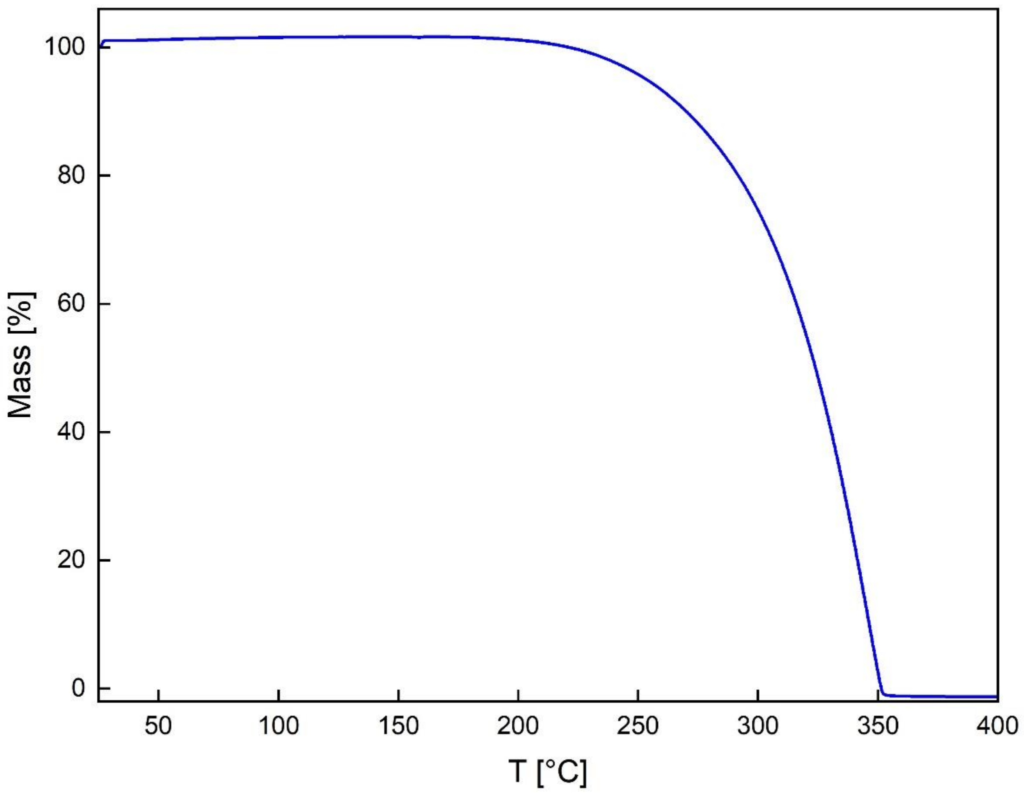

Figure S1. Thermogravimetric curve (10 °C/min) of ***(R)*-32.**

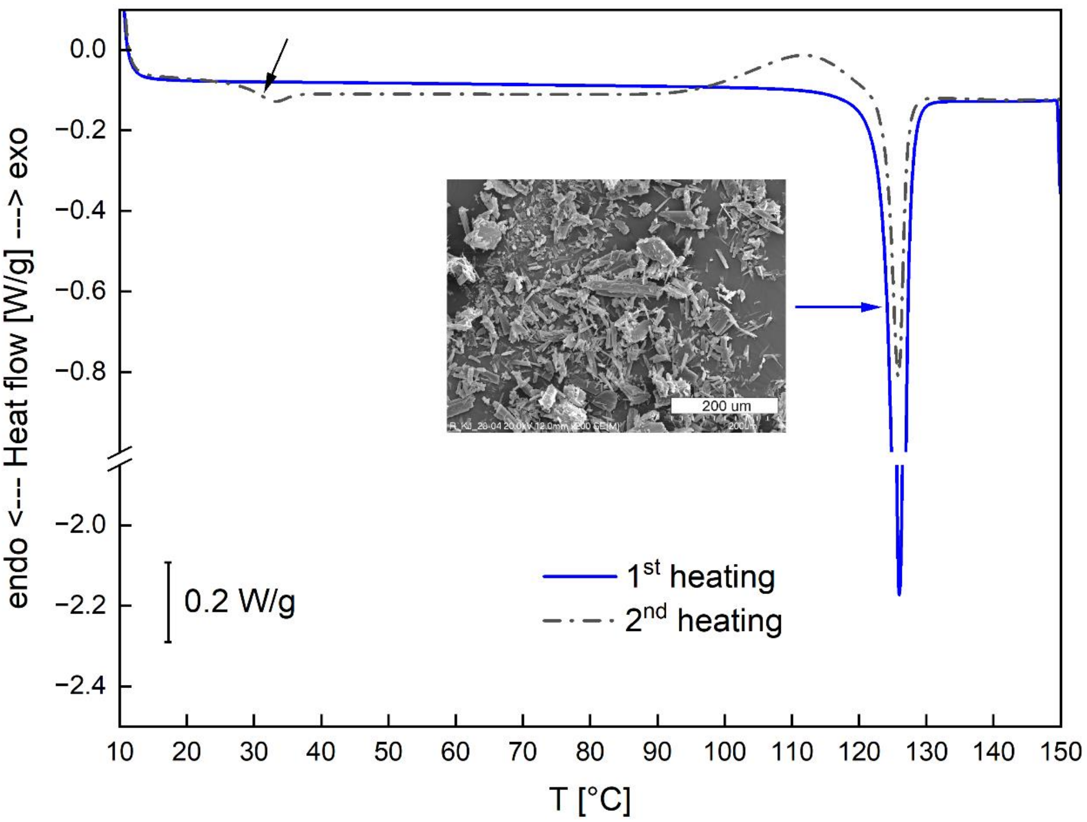

Figure S2. Heating (5 °C/min) DSC scans of ***(R)*-32**. First heating scan in blue (solid line). Second heating scan in black (dash-dot line). Inset shows ***(R)*-32** particles recorded using a scanning electron microscope (SEM, magnification 200 x). White scale bar corresponds to 200 µm. Arrows indicate position of melting endotherm (in blue) and glass transition (in black).

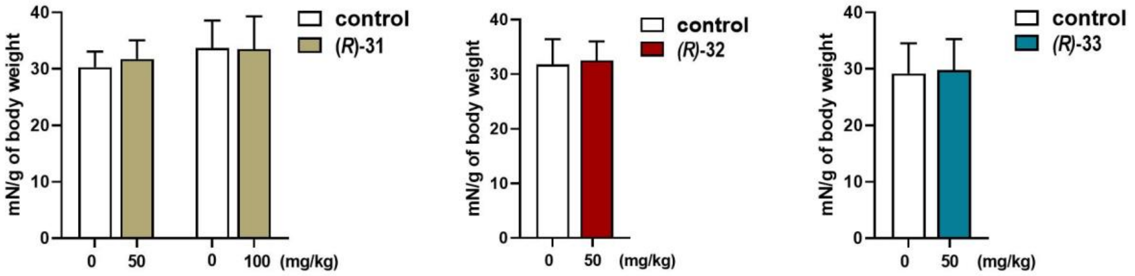

Figure S3. Effects of **(*R*)-31**, **(*R*)-32**, and **(*R*)-33** on neuromuscular strength in mice. The compounds were administered *i.p.*, 30 min before the test. Control animals received vehicle. Data are presented as mean ± SD grip strengths in millinewtons per gram of mouse body weight (mN/g); n = 10 animals. The statistical significance was evaluated using the Student's t test (GraphPad Prism 8).

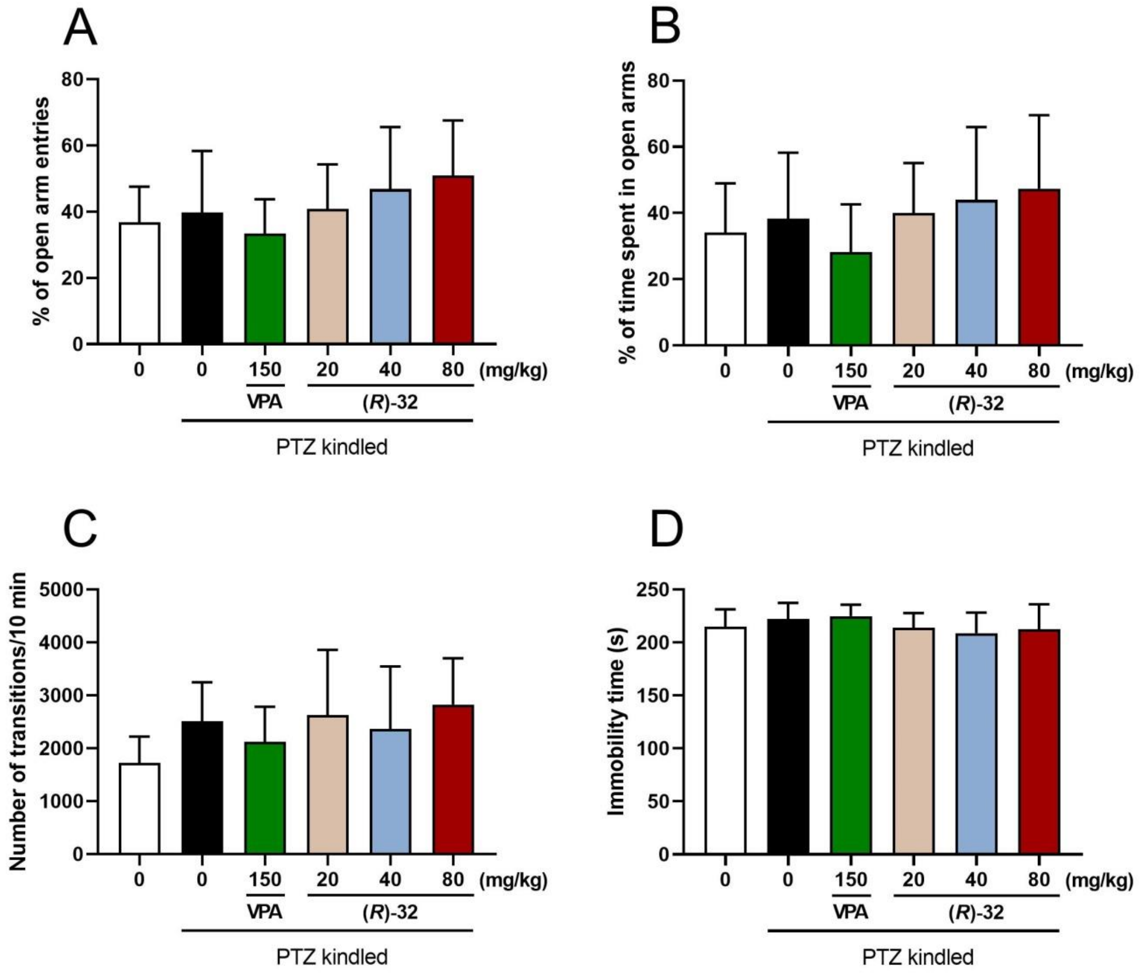

Figure S4. Effects of repeated treatment with **(*R*)-32** on anxiety, depressive-like behavior, and spontaneous locomotor activity in mice subjected to the PTZ kindling**:** (A) effect of (*R*)-32 on the percentage of the open arms entries in the elevated plus maze test; (B) effect of (*R*)-32 on the percentage of the time spent in the open arms in the elevated plus maze test; (C) effect of (*R*)-32 on the total immobility duration in the forced swim test, and (D) effect of (*R*)-32 on locomotor activity. PTZ at a subconvulsive dose of 40 mg/kg was given *i.p.* three times a week, 30 min after administration of (*R*)-32, VPA, or vehicle. Data are presented as means + SD (n=10–15). The statistical significance was evaluated by one-way ANOVA (GraphPad Prism 8).

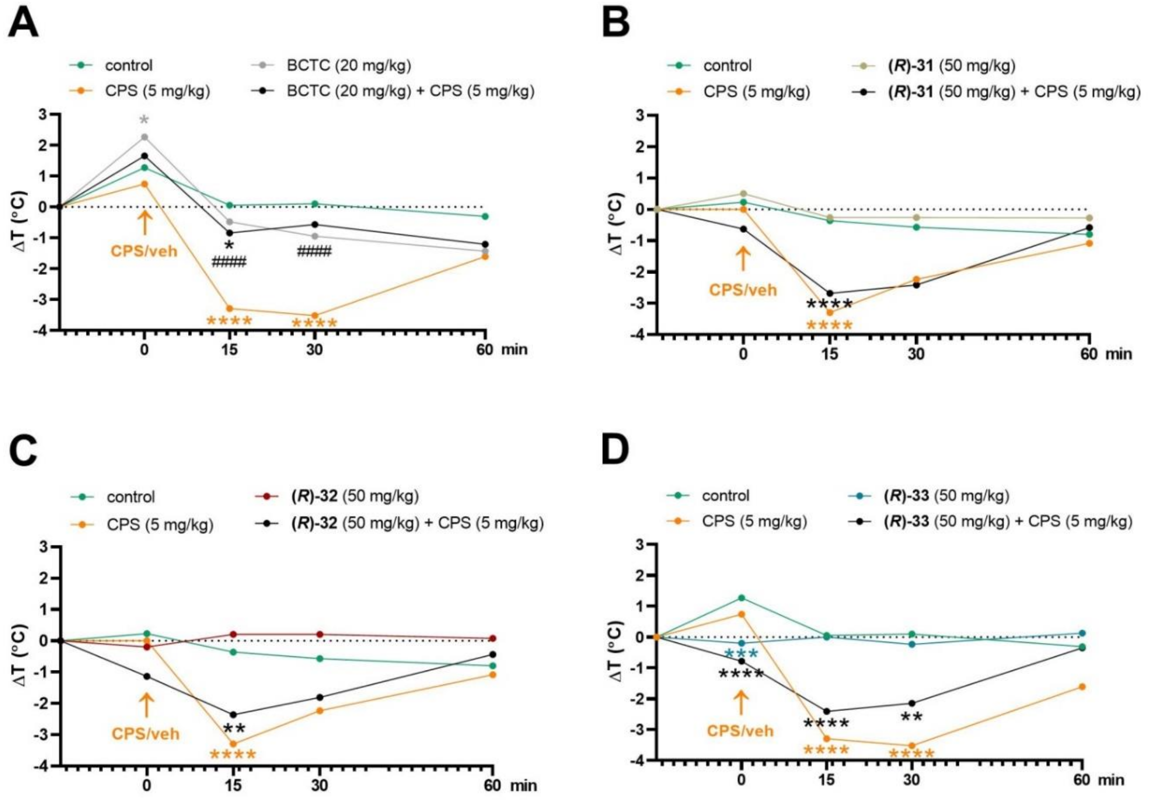

Figure S5. Acute effects of BCTC (**A**), **(*R*)-31** (**B**), **(*R*)-32** (**C**), and **(*R*)-33** (**D**) on rectal temperature in the capsaicin-induced test in mice. Capsaicin (CPS) or vehicle (1% DMSO) were injected *i.p.* at 0 min, as indicated by the arrows. BCTC, **(*R*)-31**, **(*R*)-32**, and **(*R*)-33** were suspended in 1% Tween 80 and administered *i.p.* 15 min before CPS or vehicle injection. The temperature was measured at -15, 0, 15, 30, 60, 90, 120, and 180 min. Since body temperature returned to control values 60 min after CPS injection, subsequent measurements are not shown. Data are presented as the mean differences (ΔT) in rectal temperature from 0 to -15 min (time point 0 min), from 15 to 0 min (time point 15 min), from 30 to 0 min (time point 30 min), and from 60 to 0 min (time point 60 min); n = 6–8 animals/group. Statistical significance was evaluated using one-way ANOVA with Tukey's *post hoc* test: *$p < 0.05$,**$p < 0.01$, ****$p < 0.0001$ vs. the control group; ####$p < 0.0001$ vs. the CPS-treated group.

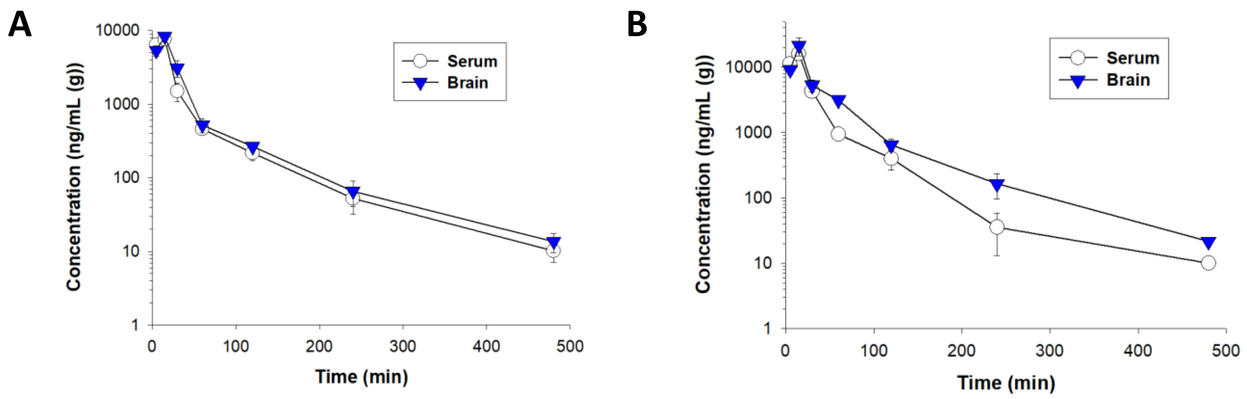

Figure S6. Mean serum and brain concentrations (± SD) for **(*R*)-32** after *i.p*. administration of this compound at a dose of 25 mg/kg (**A**) and 50 mg/kg (**B**).

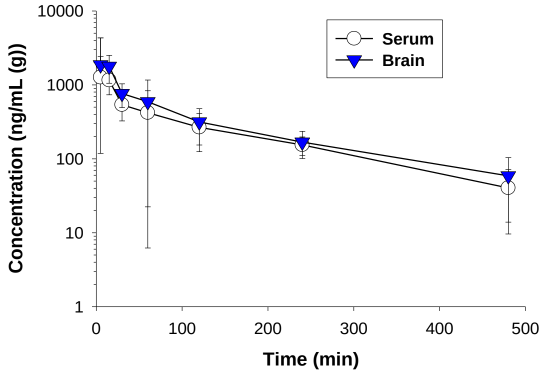

Figure S7. Mean serum and brain concentrations (± SD) for **(*R*)-32** after *p.o.* administration of this compound at a dose of 25 mg/kg.

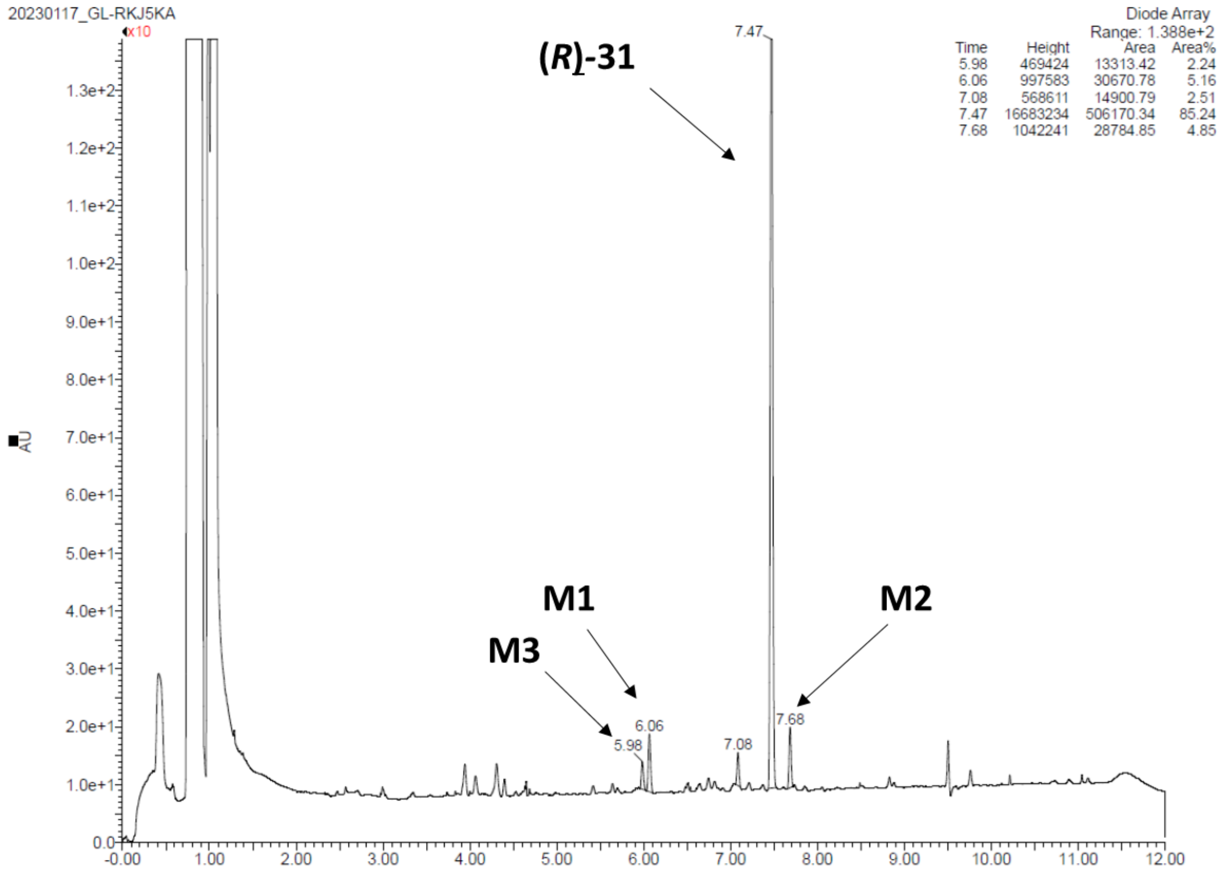

Figure S8. UPLC spectra after 120 min incubation of compound **(*R*)-31** with human liver microsomes in TRIS buffer pH=7.4 at 37ºC.

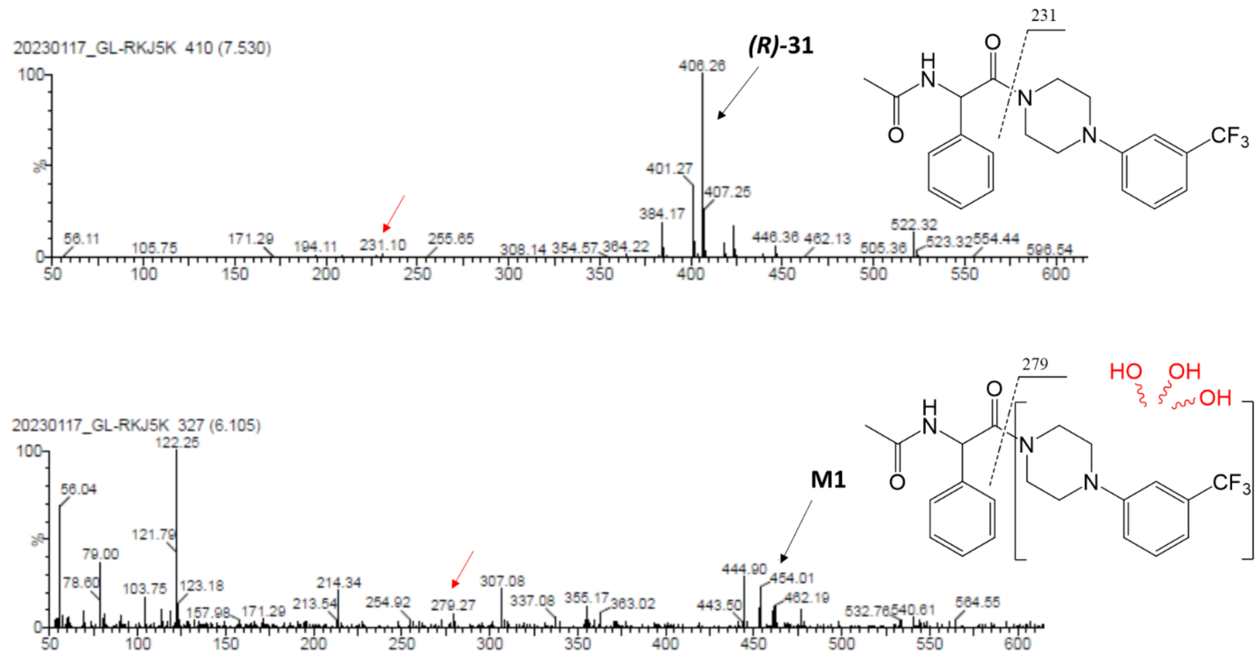

Figure S9. MS ion fragment analyses and the most probable structure of **(*R*)-31** metabolite M1.

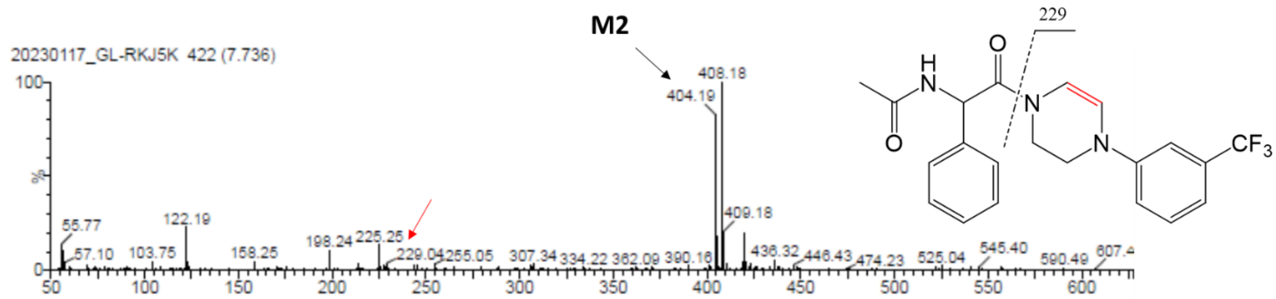

Figure S10. MS ion fragment analyses and the most probable structure of **(*R*)-31** metabolite M2.

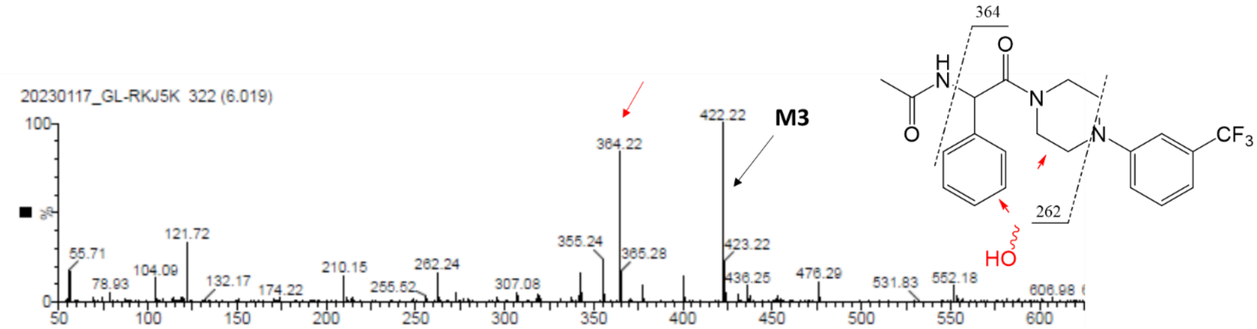

Figure S11. MS ion fragment analyses and the most probable structure of **(*R*)-31** metabolite M3.

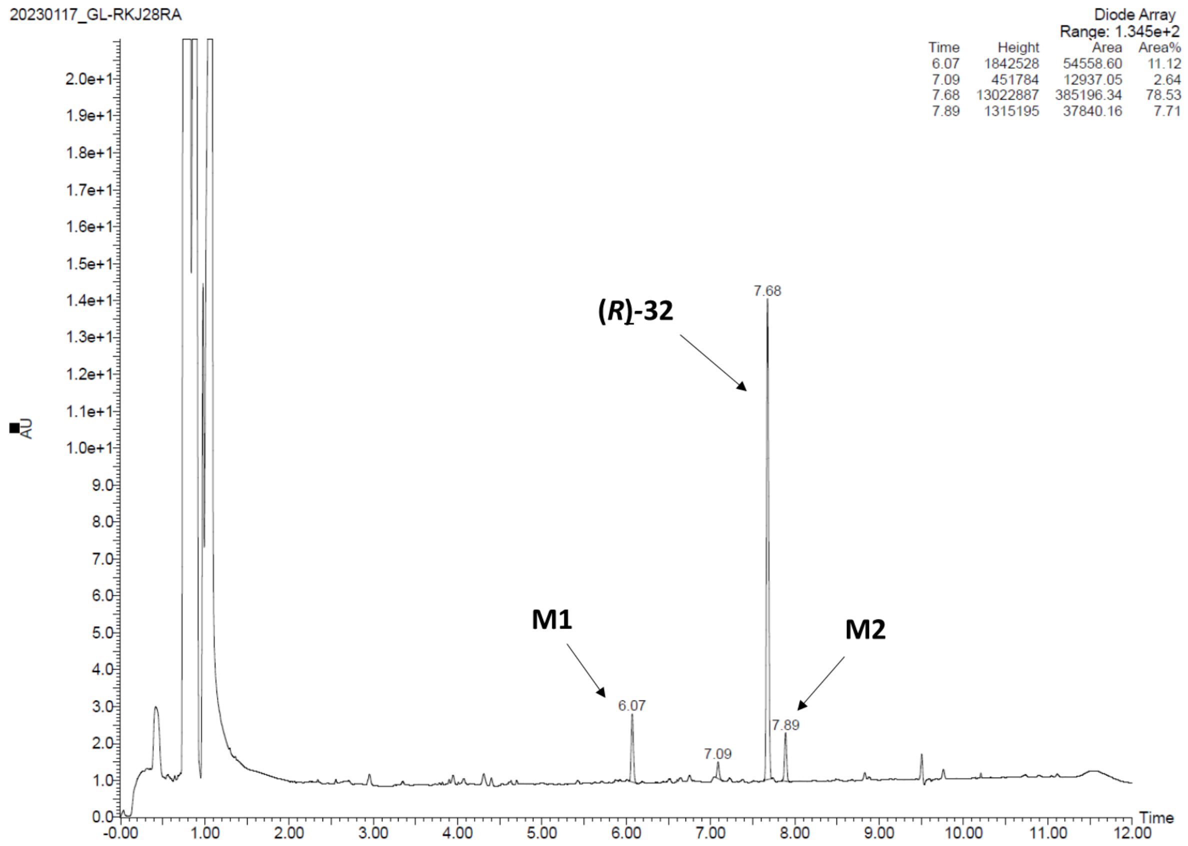

Figure S12. UPLC spectra after 120 min incubation of compound **(*R*)-32** with human liver microsomes in TRIS buffer pH=7.4 at 37ºC.

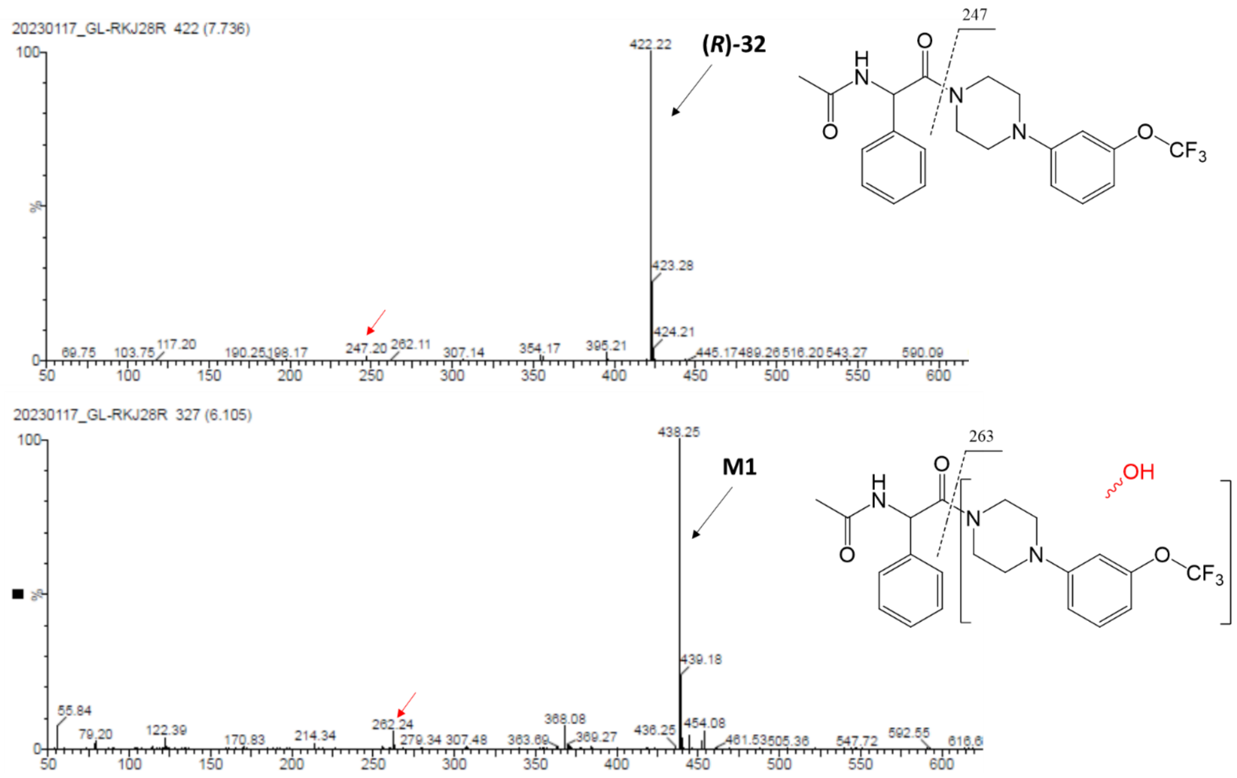

Figure S13. MS ion fragment analyses and the most probable structure of ***(R)*-32** metabolite M1.

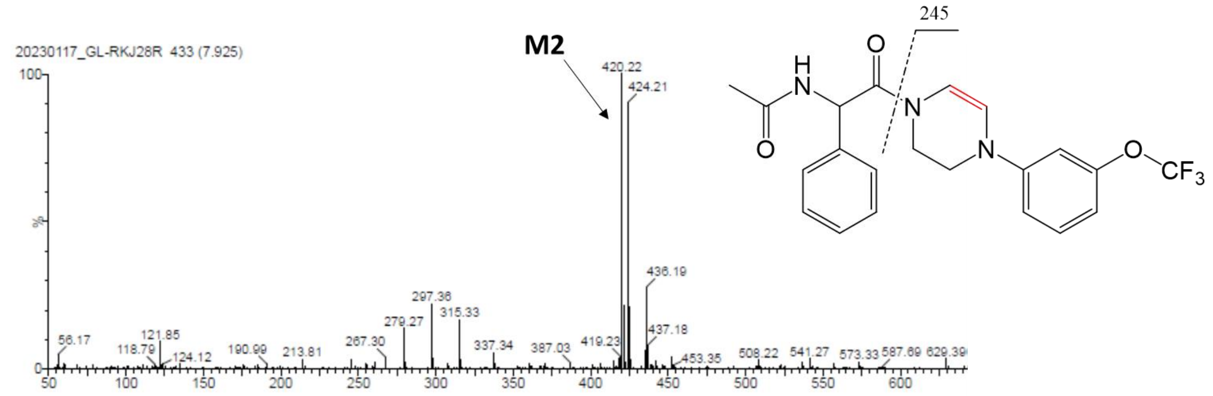

Figure S14. MS ion fragment analyses and the most probable structure of ***(R)*-32** metabolite M2.

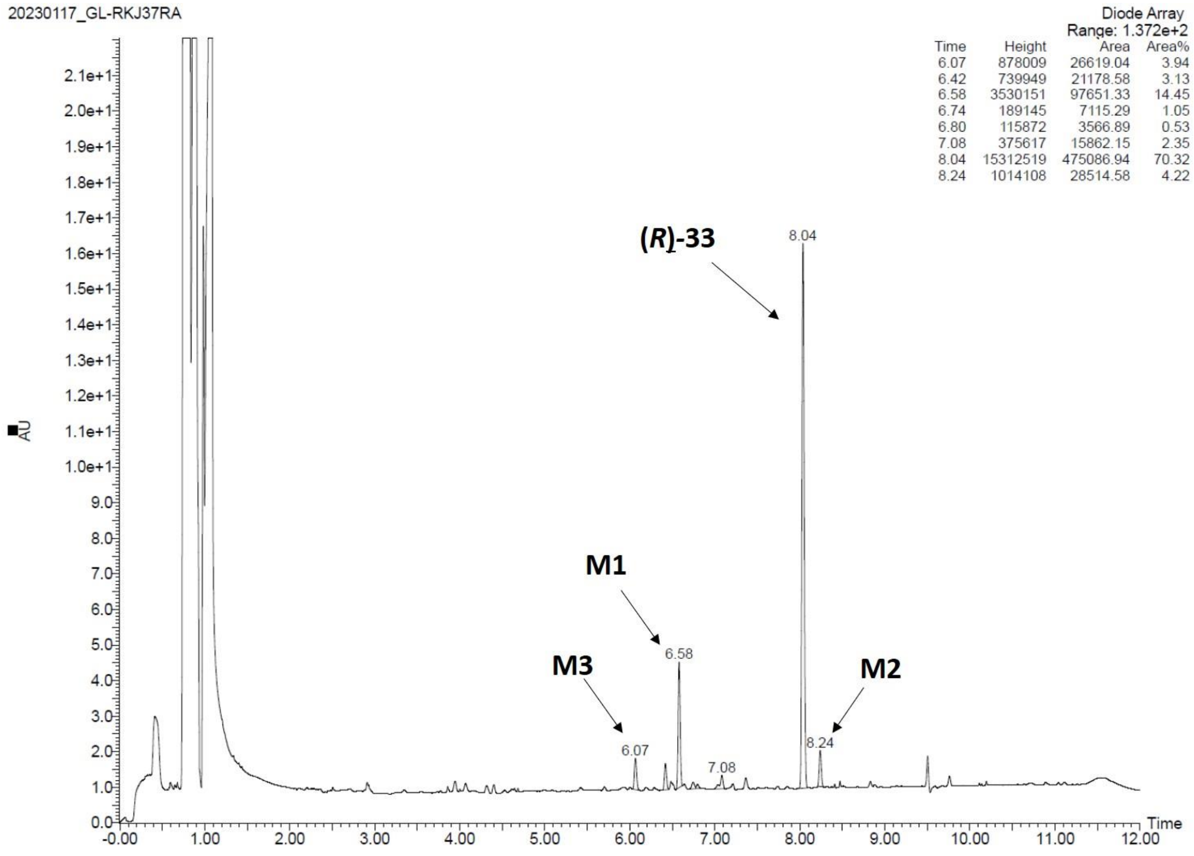

Figure S15. UPLC spectra after 120 min incubation of compound ***(R)*-33** with human liver microsomes in TRIS buffer pH=7.4 at 37ºC.

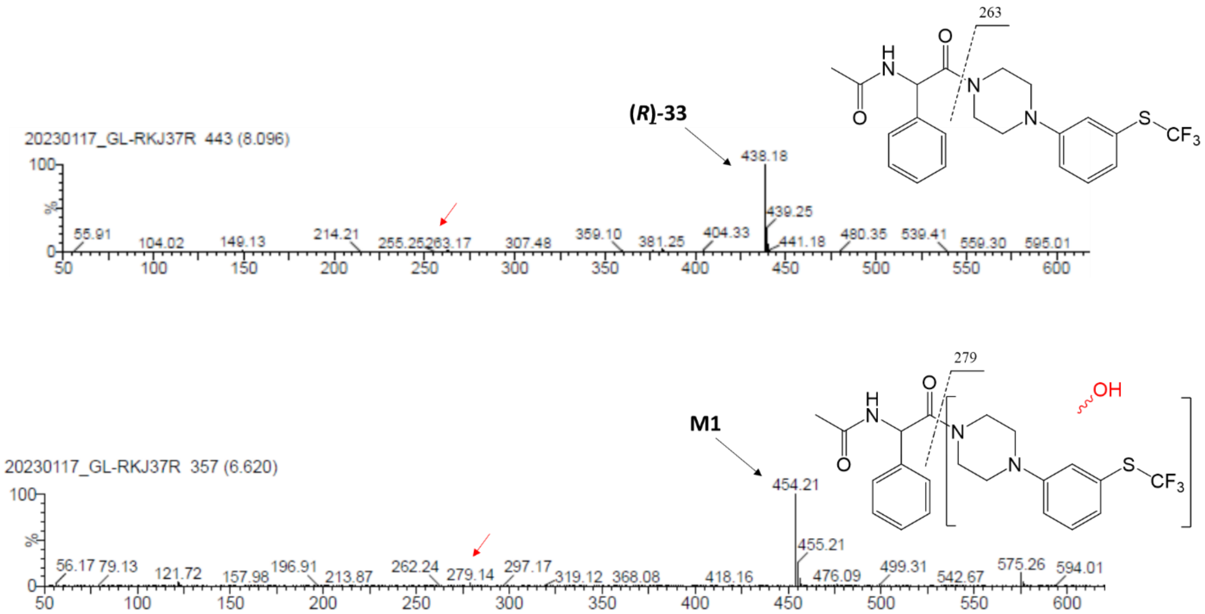

Figure S16. MS ion fragment analyses and the most probable structure of ***(R)*-33** metabolite M1.

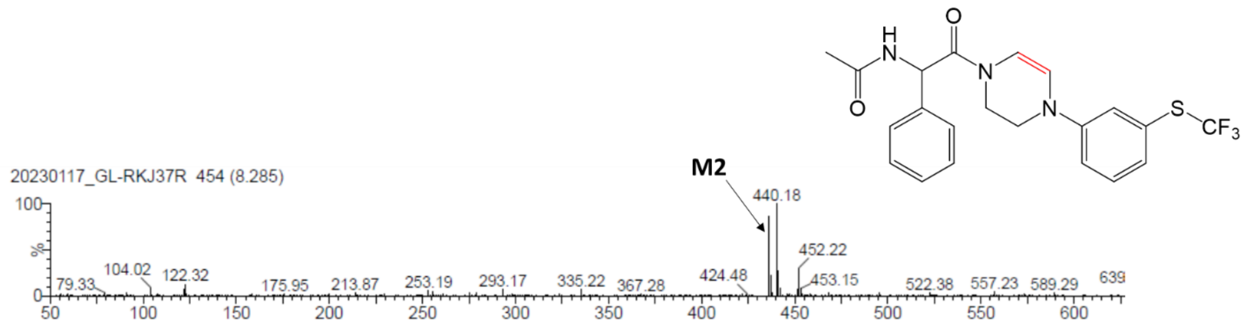

Figure S17. MS ion fragment analyses and the most probable structure of **(*R*)-33** metabolite M2.

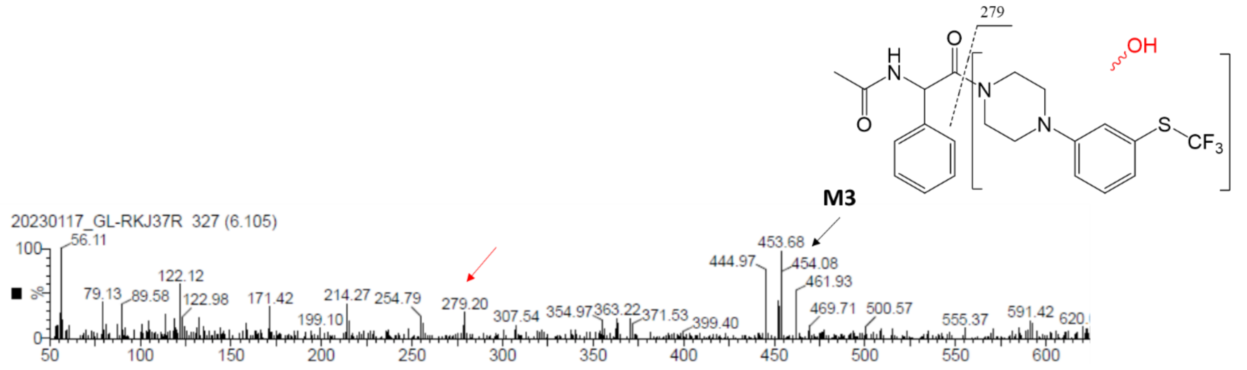

Figure S18. MS ion fragment analyses and the most probable structure of **(*R*)-33** metabolite M3.

References:

1. Brown, G.B. 3H-Batrachotoxinin-A Benzoate Binding to Voltage-Sensitive Sodium Channels: Inhibition by the Channel Blockers Tetrodotoxin and Saxitoxin. *J. Neurosci.* **1986**, *6*, 2064–2070, doi:10.1523/JNEUROSCI.06-07-02064.1986.
2. Sirenko, O.; Crittenden, C.; Callamaras, N.; Hesley, J.; Chen, Y.-W.; Funes, C.; Rusyn, I.; Anson, B.; Cromwell, E.F. Multiparameter In Vitro Assessment of Compound Effects on Cardiomyocyte Physiology Using IPSC Cells. *J. Biomol. Screen.* **2013**, *18*, 39–53, doi:10.1177/1087057112457590.
3. Xia, M.; Imredy, J.P.; Koblan, K.S.; Bennett, P.; Connolly, T.M. State-Dependent Inhibition of L-Type Calcium Channels: Cell-Based Assay in High-Throughput Format. *Analytical Biochemistry* **2004**, *327*, 74–81, doi:10.1016/j.ab.2004.01.003.
4. Phelps, P.T.; Anthes, J.C.; Correll, C.C. Cloning and Functional Characterization of Dog Transient Receptor Potential Vanilloid Receptor-1 (TRPV1). *Eur. J. Pharmacol.* **2005**, *513*, 57–66, doi:10.1016/j.ejphar.2005.02.045.
5. Https://Www.Eurofinsdiscovery.Com/Catalogmanagement/ViewItem/TRPA1-Human-Transient-Potential-Ion-Channel-Cell-Based-Antagonist-Calcium-Flux-Assay-Cerep/5372.
6. Behrendt, H.-J.; Germann, T.; Gillen, C.; Hatt, H.; Jostock, R. Characterization of the Mouse Cold-Menthol Receptor TRPM8 and Vanilloid Receptor Type-1 VR1 Using a Fluorometric Imaging Plate Reader (FLIPR) Assay. *Br. J. Pharmacol.* **2004**, *141*, 737–745, doi:10.1038/sj.bjp.0705652.
7. Gould, R.J.; Murphy, K.M.; Snyder, S.H. [3H]Nitrendipine-Labeled Calcium Channels Discriminate Inorganic Calcium Agonists and Antagonists. *Proc. Natl. Acad. Sci. U.S.A.* **1982**, *79*, 3656–3660, doi:10.1073/pnas.79.11.3656.
8. Felder, C.; Joyce, K.; Briley, E. Comparison of the Pharmacology and Signal Transduction of the Human Cannabinoid CB1 and CB2 Receptor. *Mol. Pharmacol.***1995**, *48*, 443–450.

HMRS traces for selected final compounds

(*R*)-*N*-(2-oxo-1-phenyl-2-(4-(3-(trifluoromethyl)phenyl)piperazin-1-yl)ethyl)acetamide ((*R*)-31)

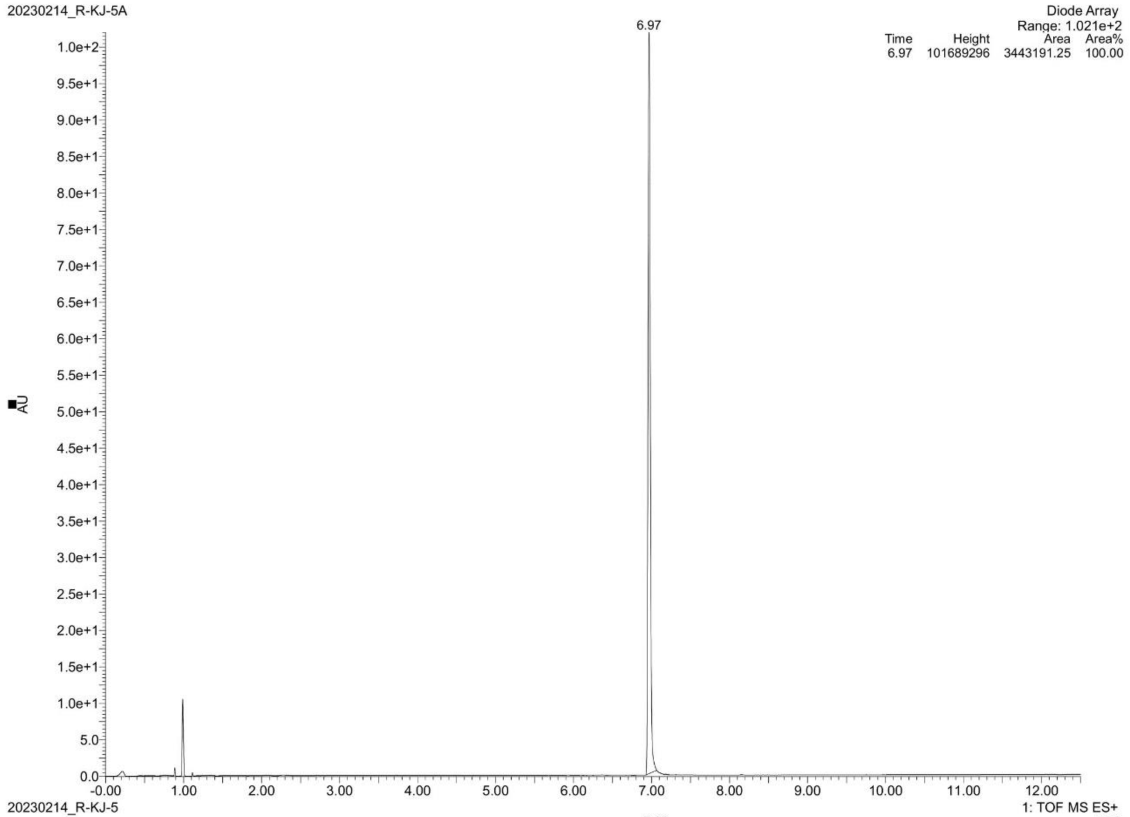

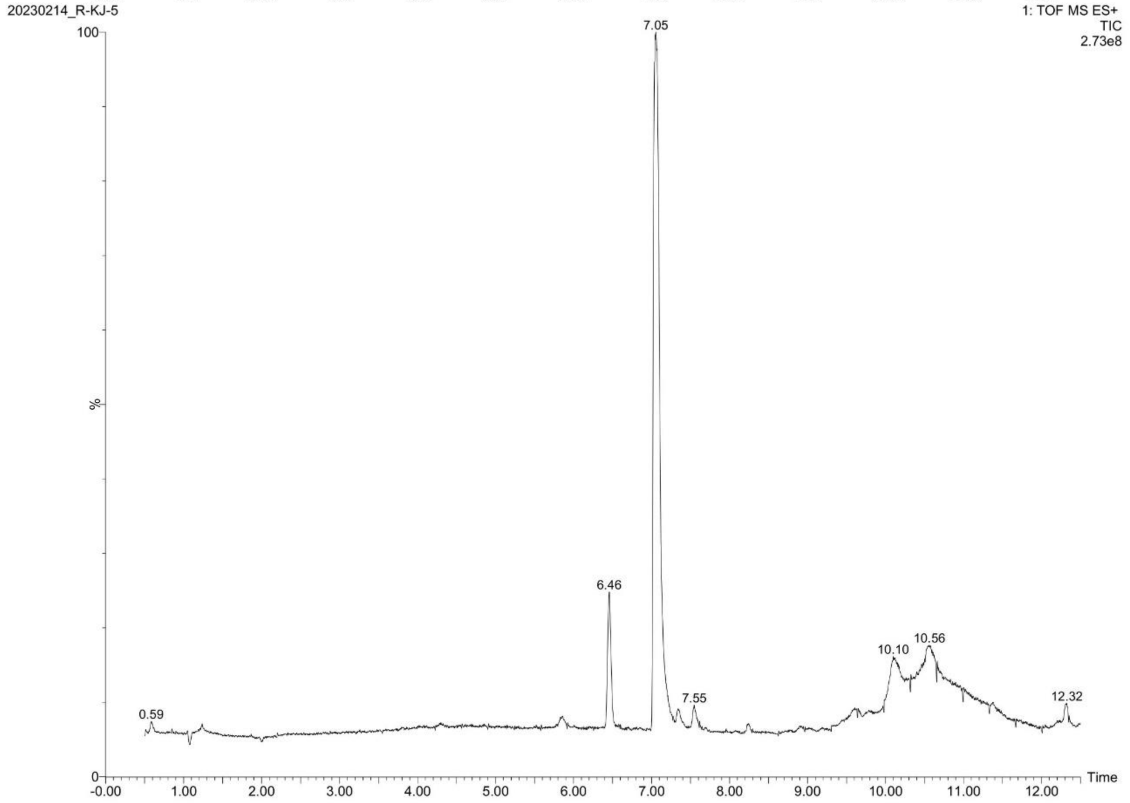

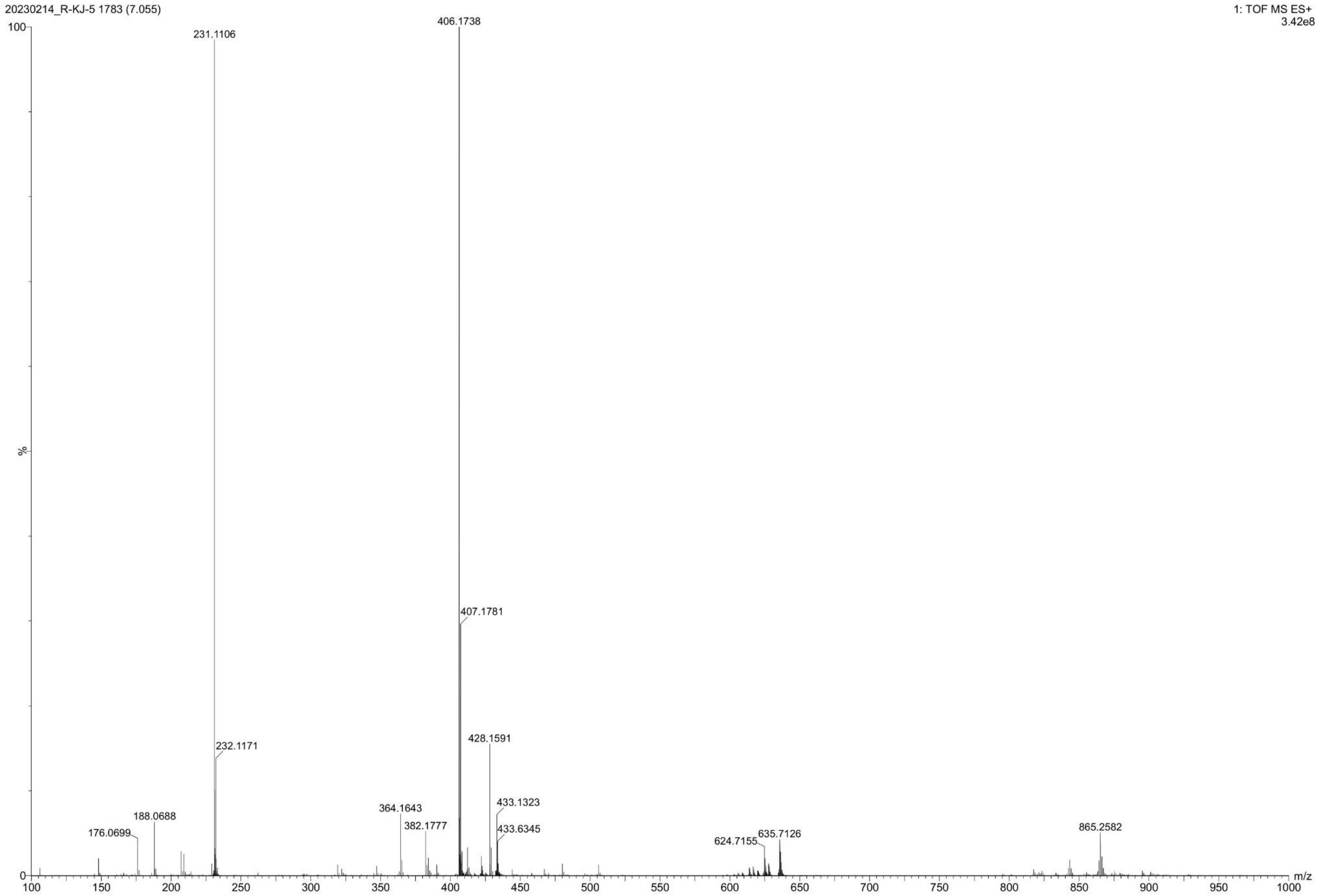
20230214_R-KJ-5 1783 (7.055)
1: TOF MS ES+
3.42e8
100
%
0
406.1738
231.1106
407.1781
428.1591
232.1171
433.1323
433.6345
364.1643
382.1777
188.0688
176.0699
624.7155
635.7126
865.2582
m/z
100
150
200
250
300
350
400
450
500
550
600
650
700
750
800
850
900
950
1000

(*R*)-*N*-(2-oxo-1-phenyl-2-(4-(3-(trifluoromethoxy)phenyl)piperazin-1-yl)ethyl)acetamide ((*R*)-32)

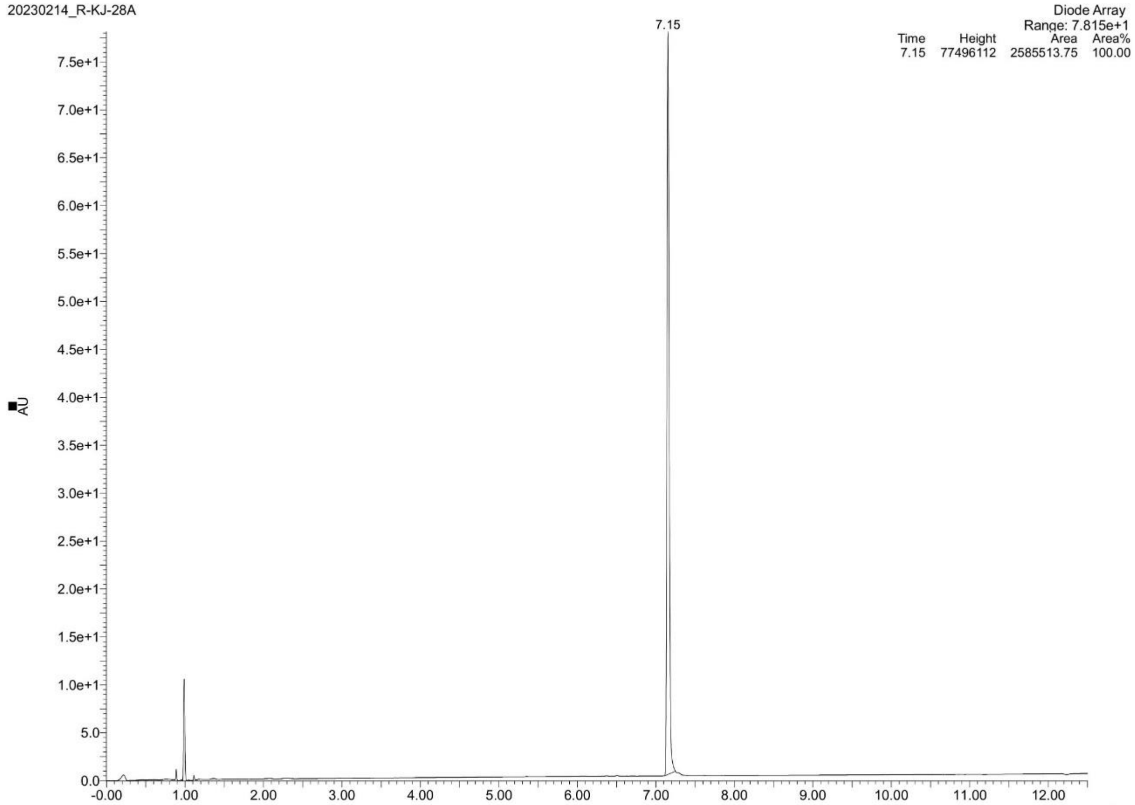

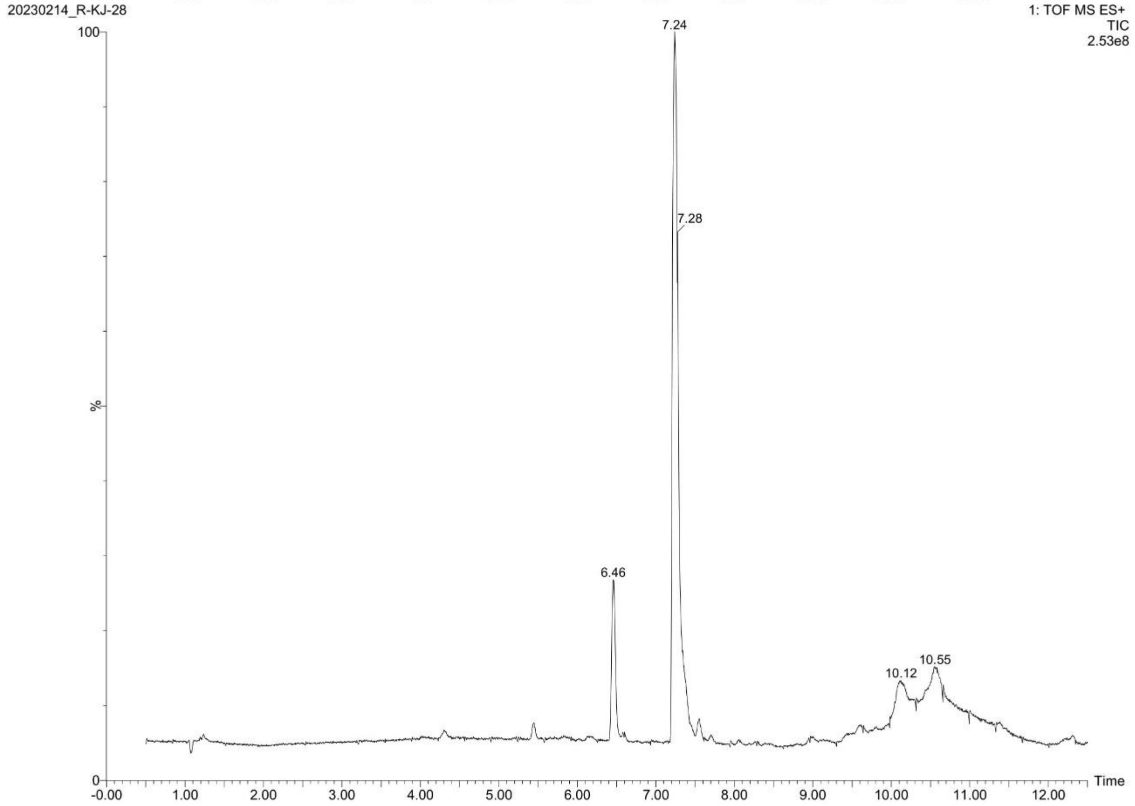

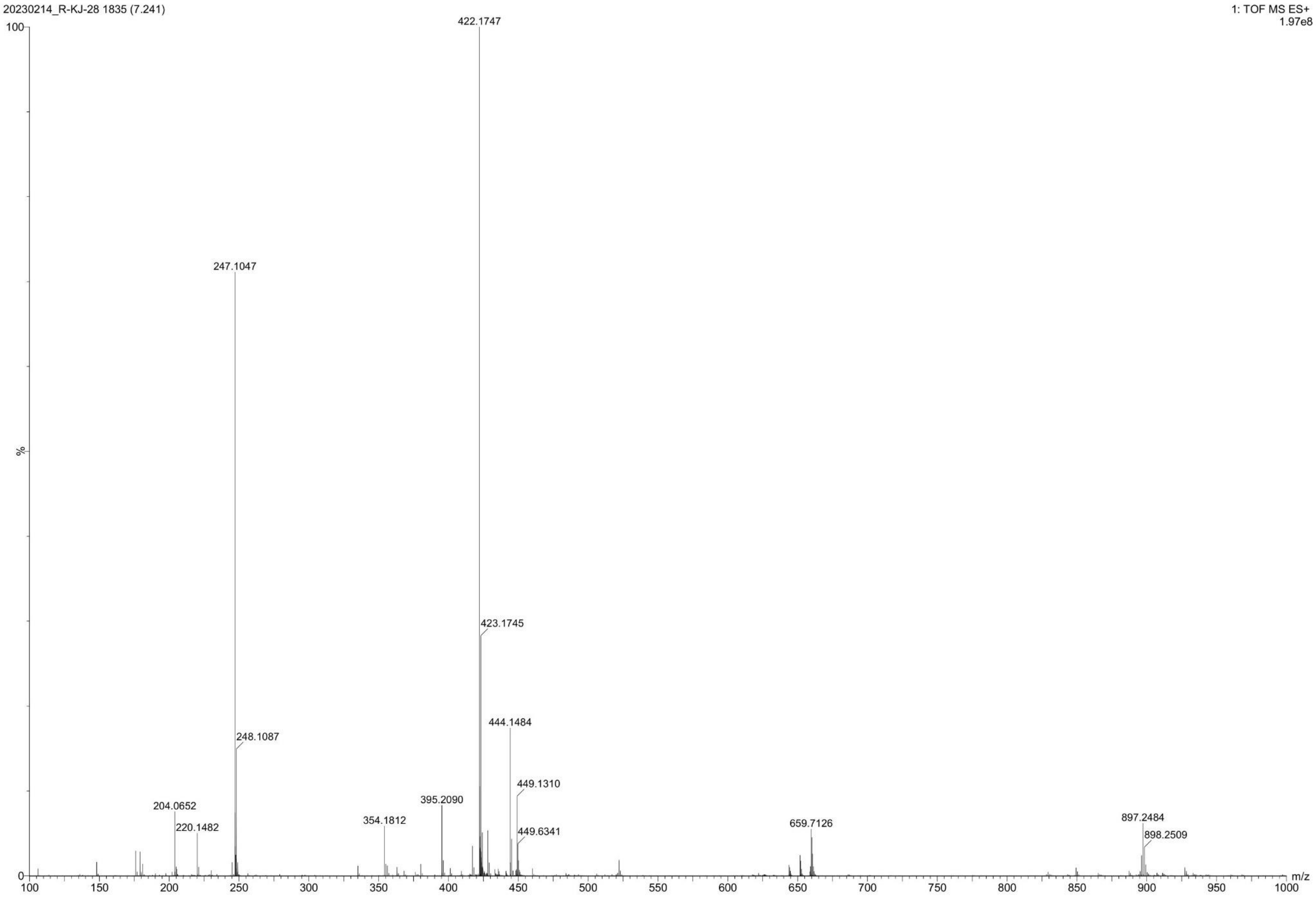

20230214_R-KJ-28 1835 (7.241)
1: TOF MS ES+
1.97e8
422.1747
247.1047
423.1745
444.1484
248.1087
449.1310
204.0652
395.2090
220.1482
354.1812
449.6341
659.7126
897.2484
898.2509
%
m/z

(*R*)-*N*-(2-oxo-1-phenyl-2-(4-(3-((trifluoromethyl)thio)phenyl)piperazin-1-yl)ethyl)acetamide ((*R*)-33).

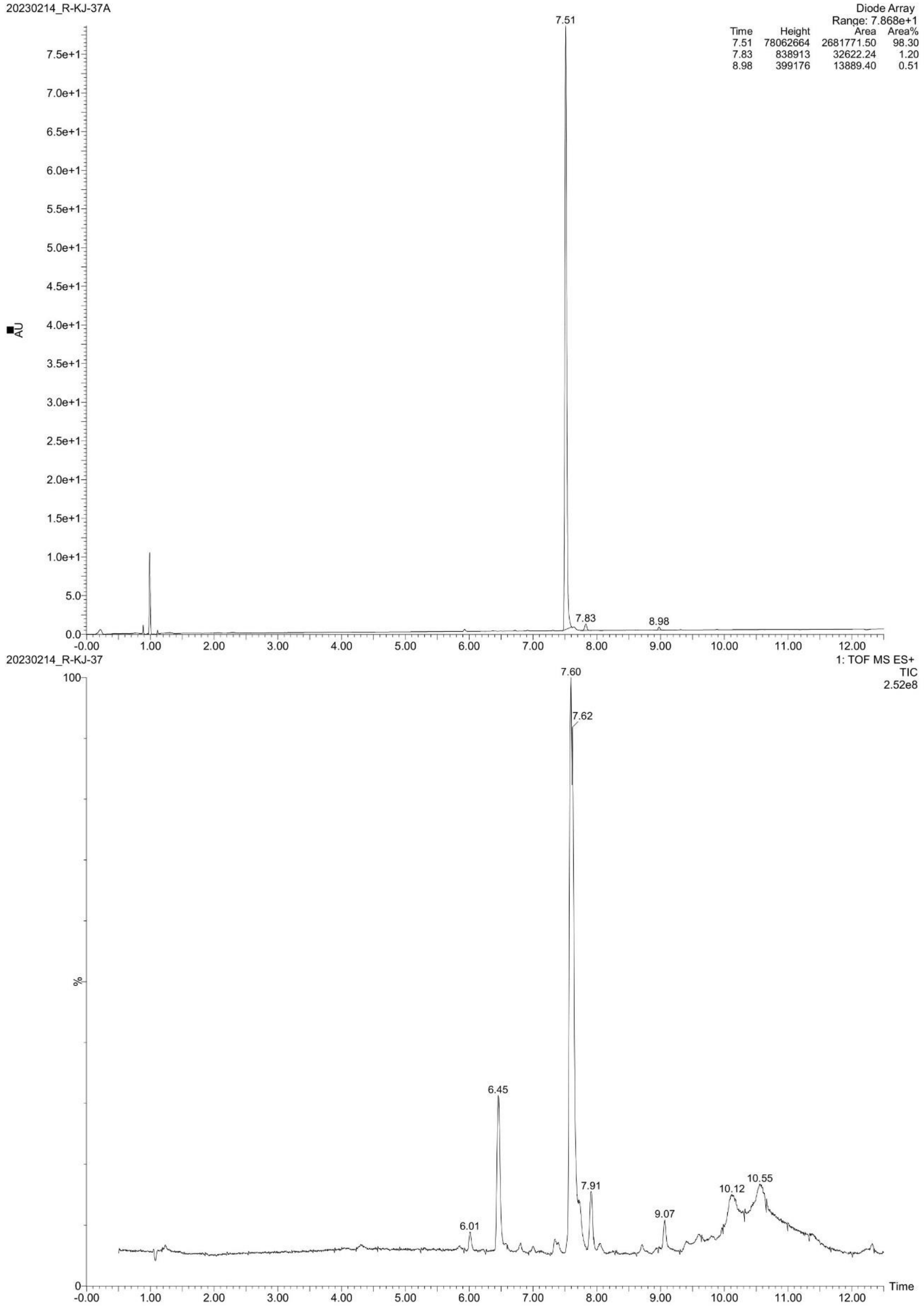

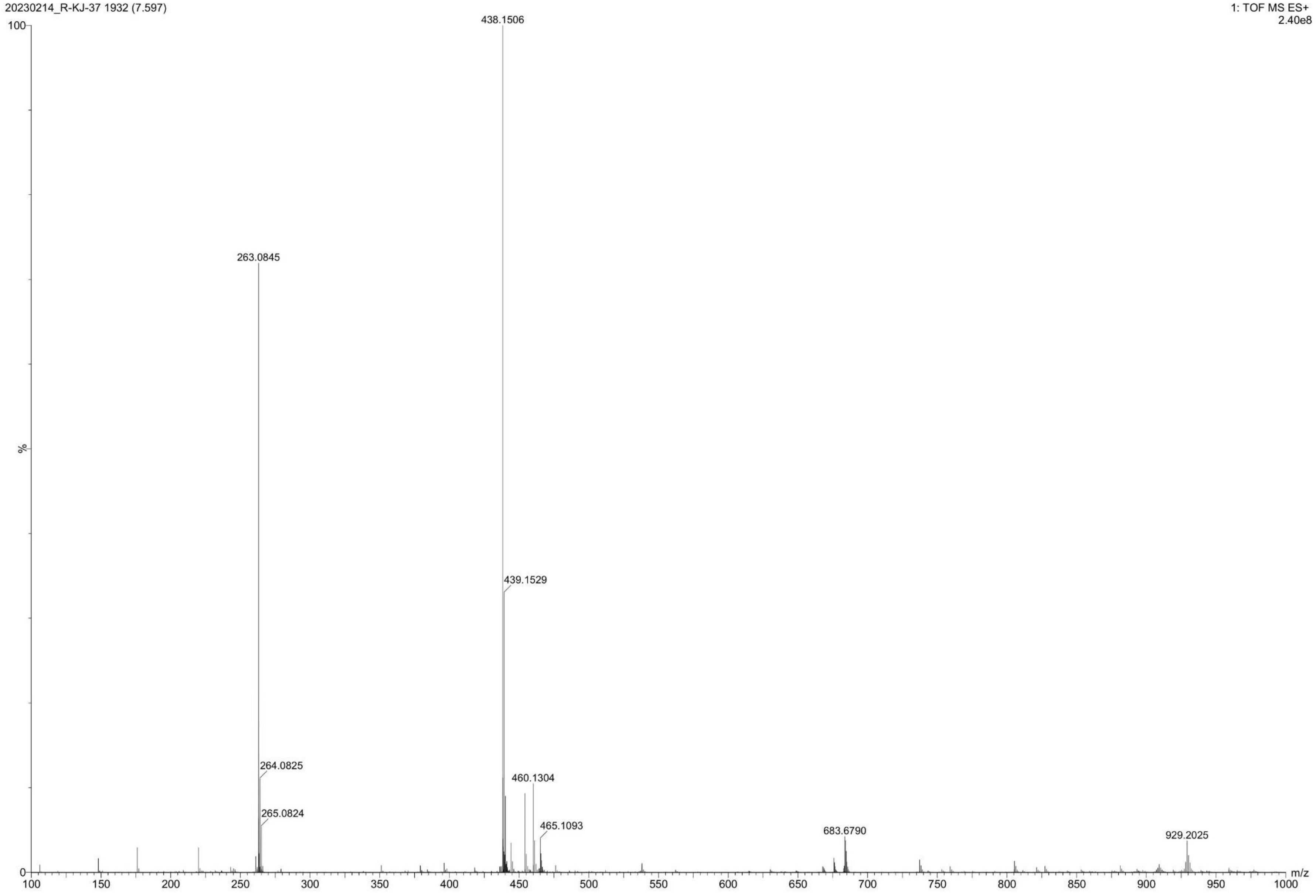
20230214_R-KJ-37 1932 (7.597)
1: TOF MS ES+
2.40e8
438.1506
263.0845
439.1529
264.0825
460.1304
265.0824
465.1093
683.6790
929.2025
100
%
0
100
150
200
250
300
350
400
450
500
550
600
650
700
750
800
850
900
950
1000
m/z

^{1}H NMR and ^{13}C NMR spectra for the final compounds

N-(2-oxo-1-phenyl-2-(4-(3-(trifluoromethyl)phenyl)piperazin-1-yl)ethyl)benzamide (3) – ^{1}H NMR

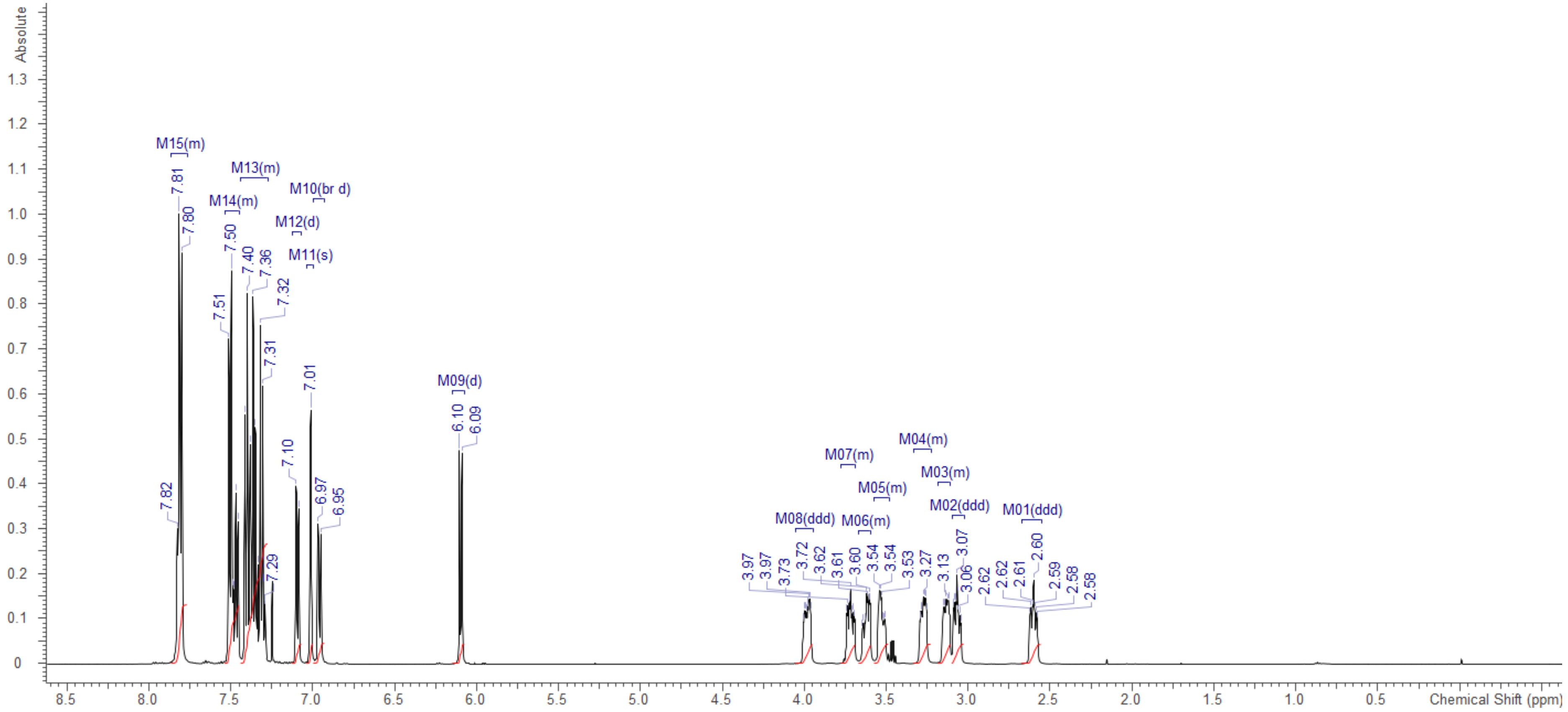

***N*-(2-oxo-1-phenyl-2-(4-(3-(trifluoromethyl)phenyl)piperazin-1-yl)ethyl)benzamide (3) – ^{13}CNMR**

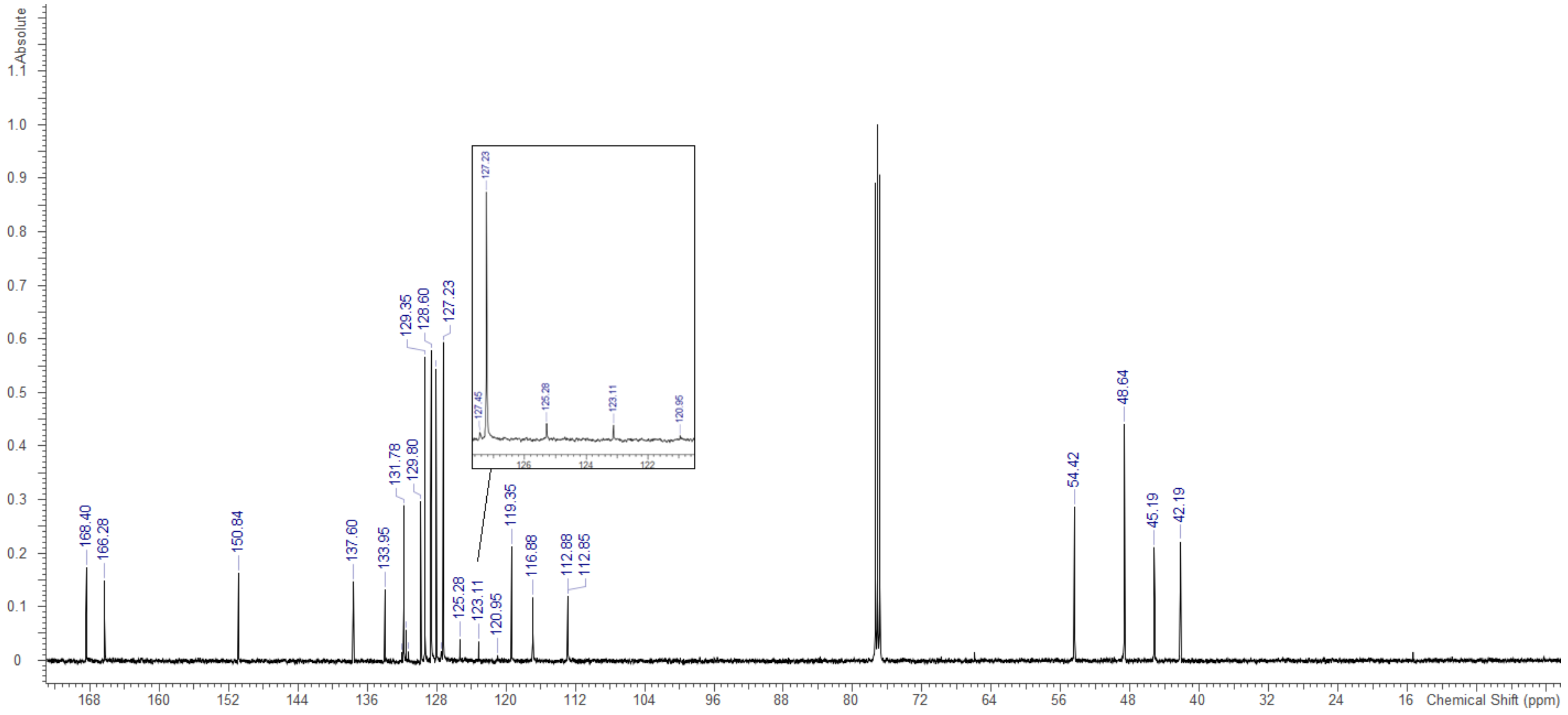

2-chloro-*N*-(2-oxo-1-phenyl-2-(4-(3-(trifluoromethyl)phenyl)piperazin-1-yl)ethyl) benzamide (4) – ^{1}H NMR

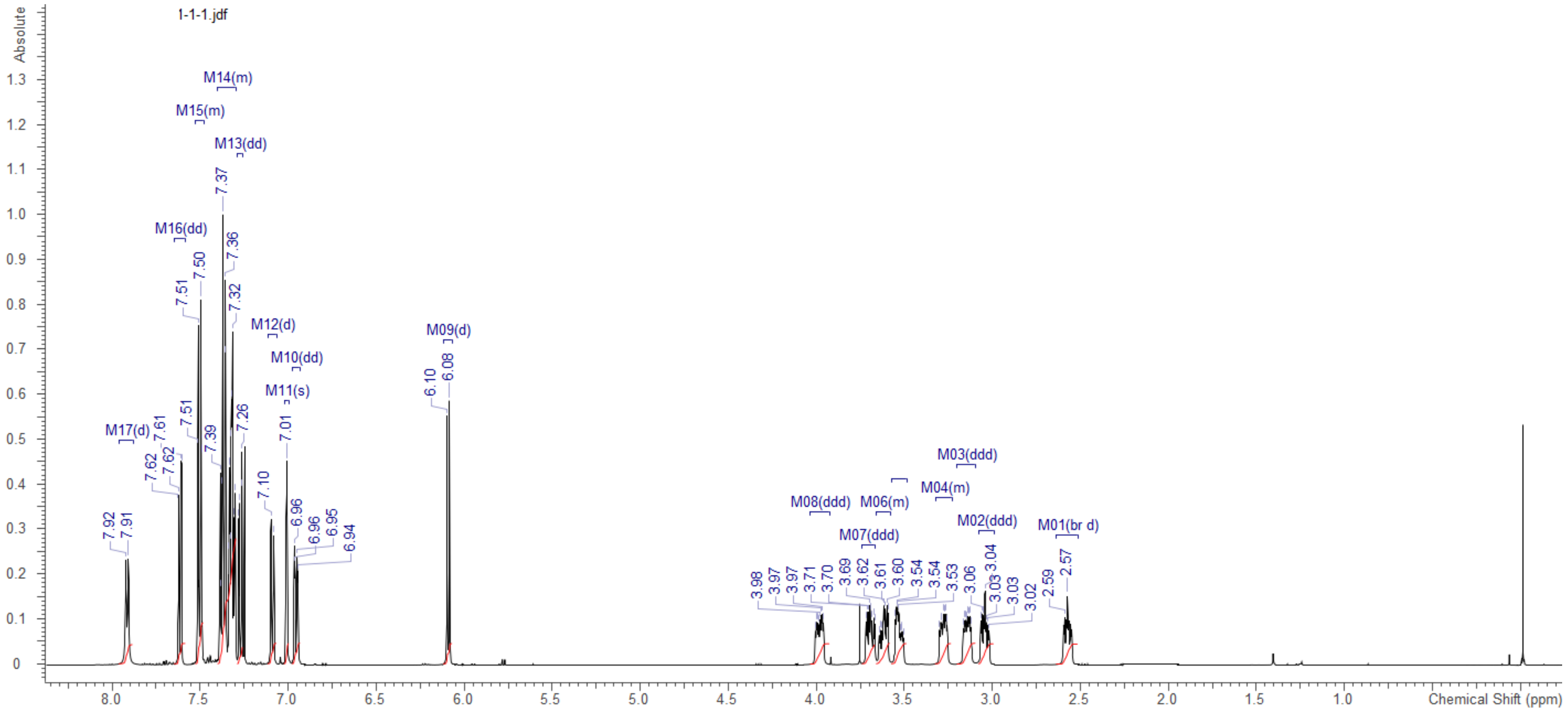

2-chloro-*N*-(2-oxo-1-phenyl-2-(4-(3-(trifluoromethyl)phenyl)piperazin-1-yl)ethyl) benzamide (4) – ^{13}C NMR

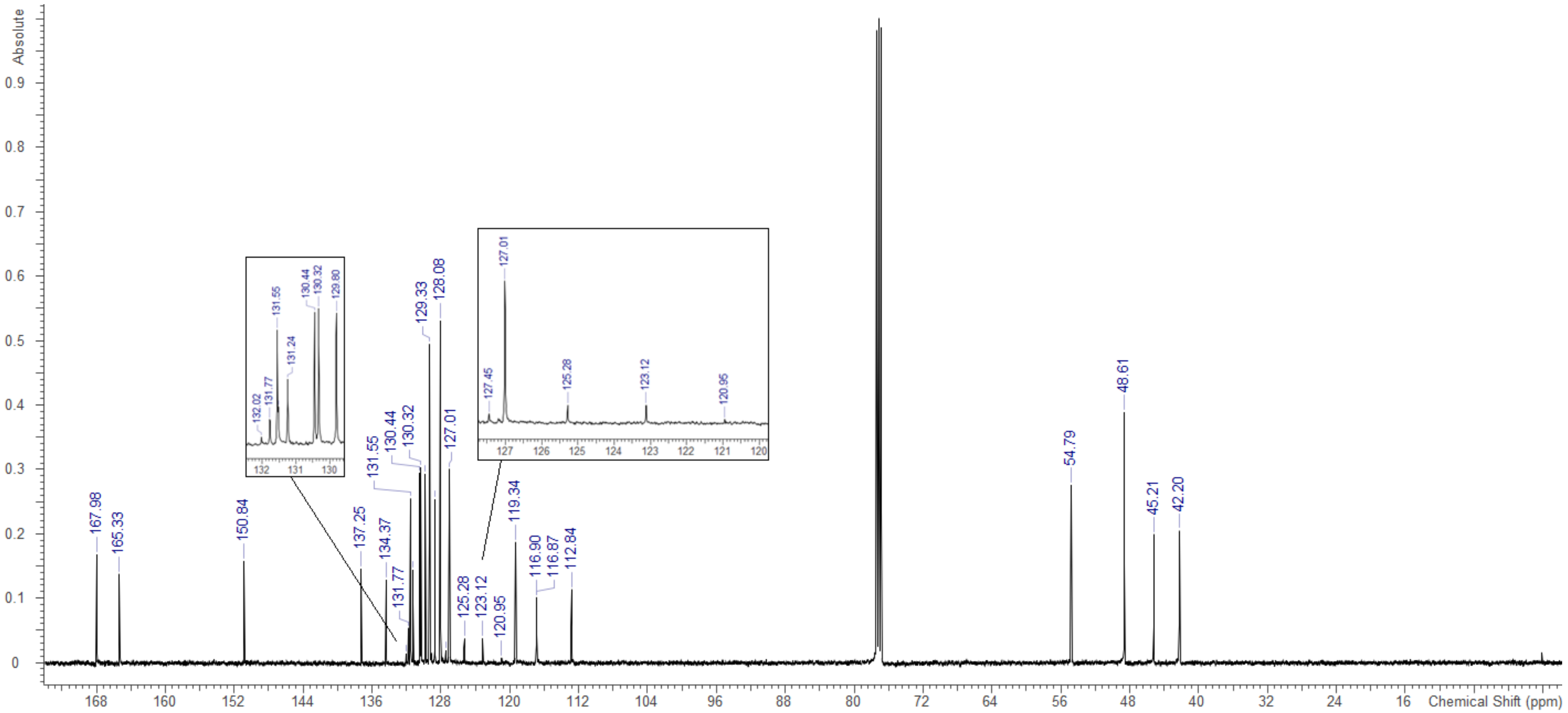

3-chloro-*N*-(2-oxo-1-phenyl-2-(4-(3-(trifluoromethyl)phenyl)piperazin-1-yl)ethyl) benzamide (5) – ^{1}H NMR

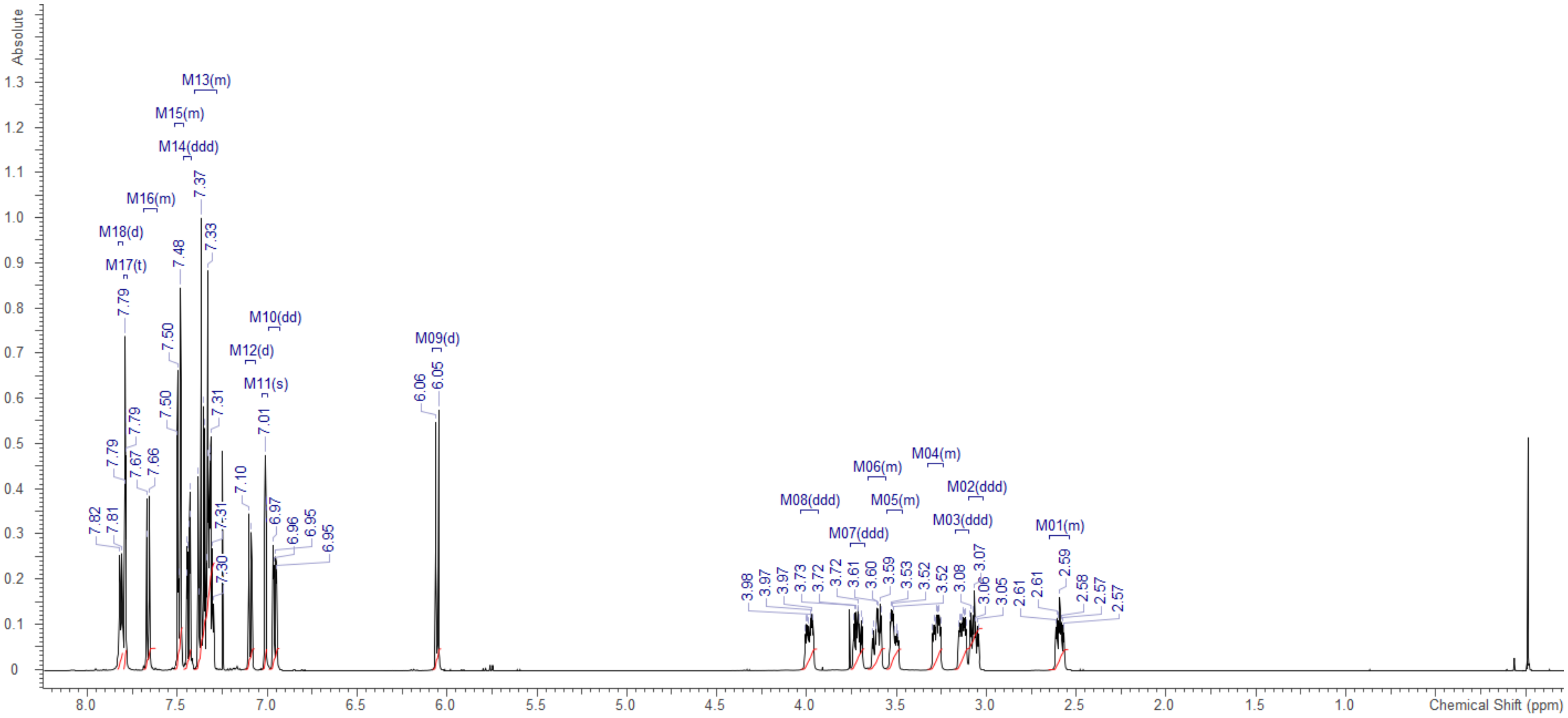

3-chloro-*N*-(2-oxo-1-phenyl-2-(4-(3-(trifluoromethyl)phenyl)piperazin-1-yl)ethyl) benzamide (5) – ^{13}C NMR

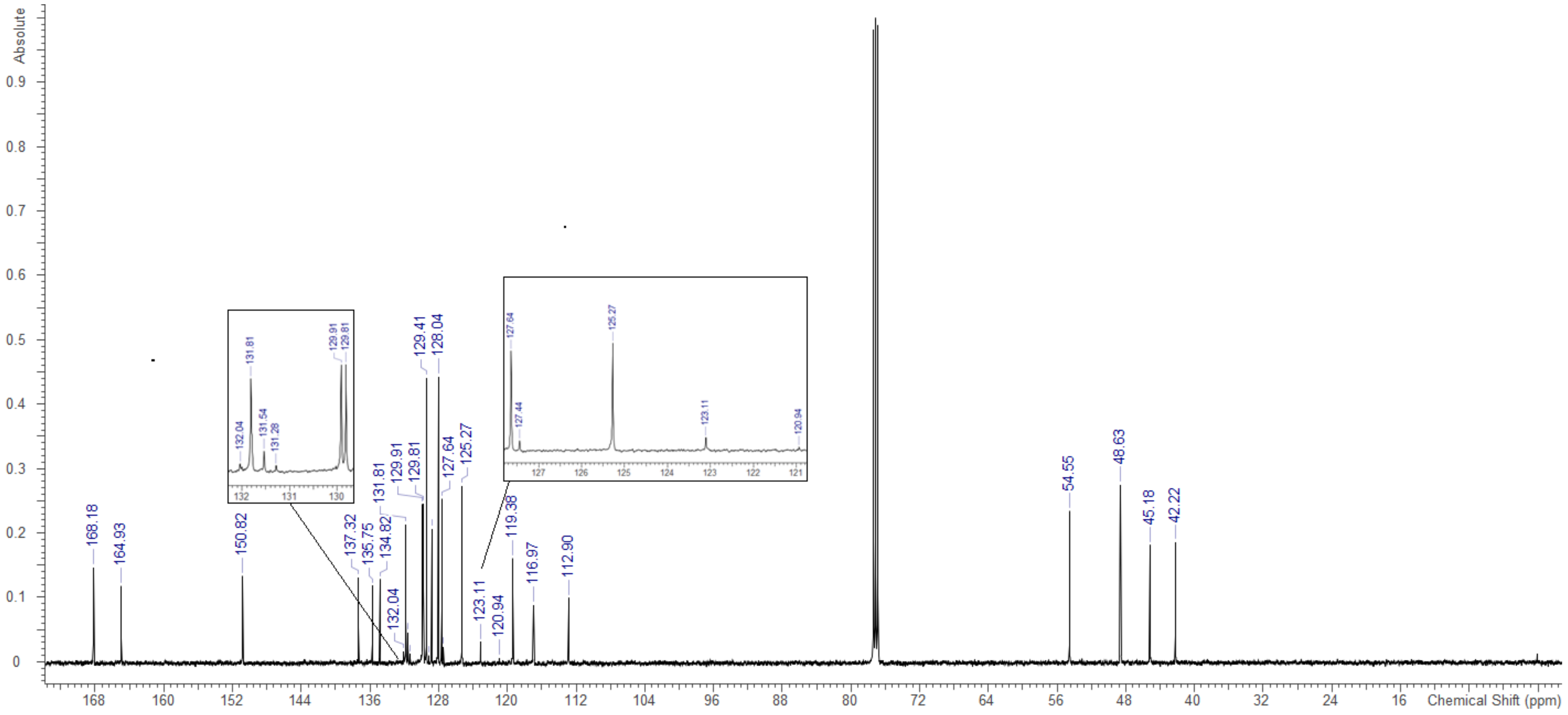

4-chloro-*N*-(2-oxo-1-phenyl-2-(4-(3-(trifluoromethyl)phenyl)piperazin-1-yl)ethyl) benzamide (6) – ^{1}H NMR

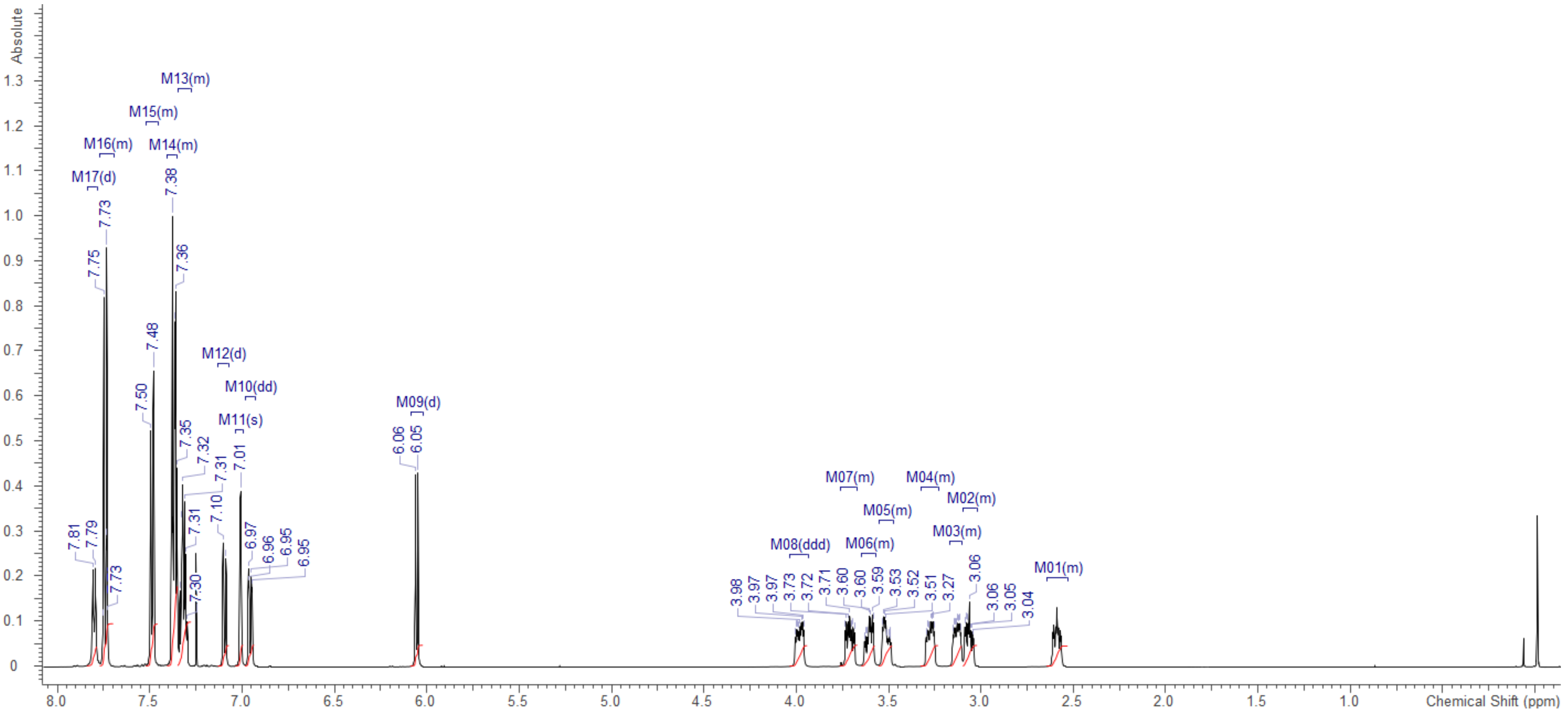

4-chloro-*N*-(2-oxo-1-phenyl-2-(4-(3-(trifluoromethyl)phenyl)piperazin-1-yl)ethyl) benzamide (6) – ^{13}C NMR

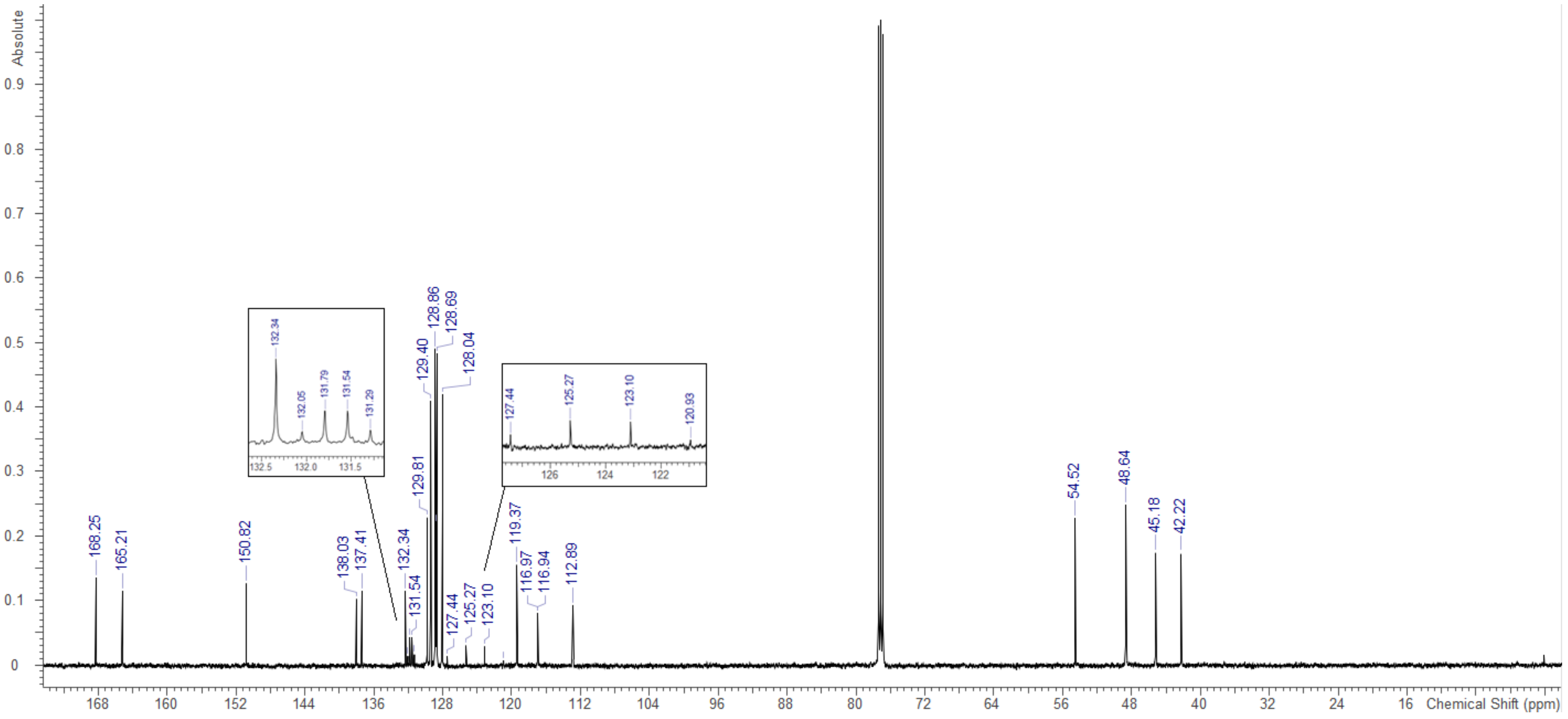

***N*-(2-oxo-1-phenyl-2-(4-(3-(trifluoromethyl)phenyl)piperazin-1-yl)ethyl)-2 (trifluoromethyl)benzamide (7) – ^{1}H NMR**

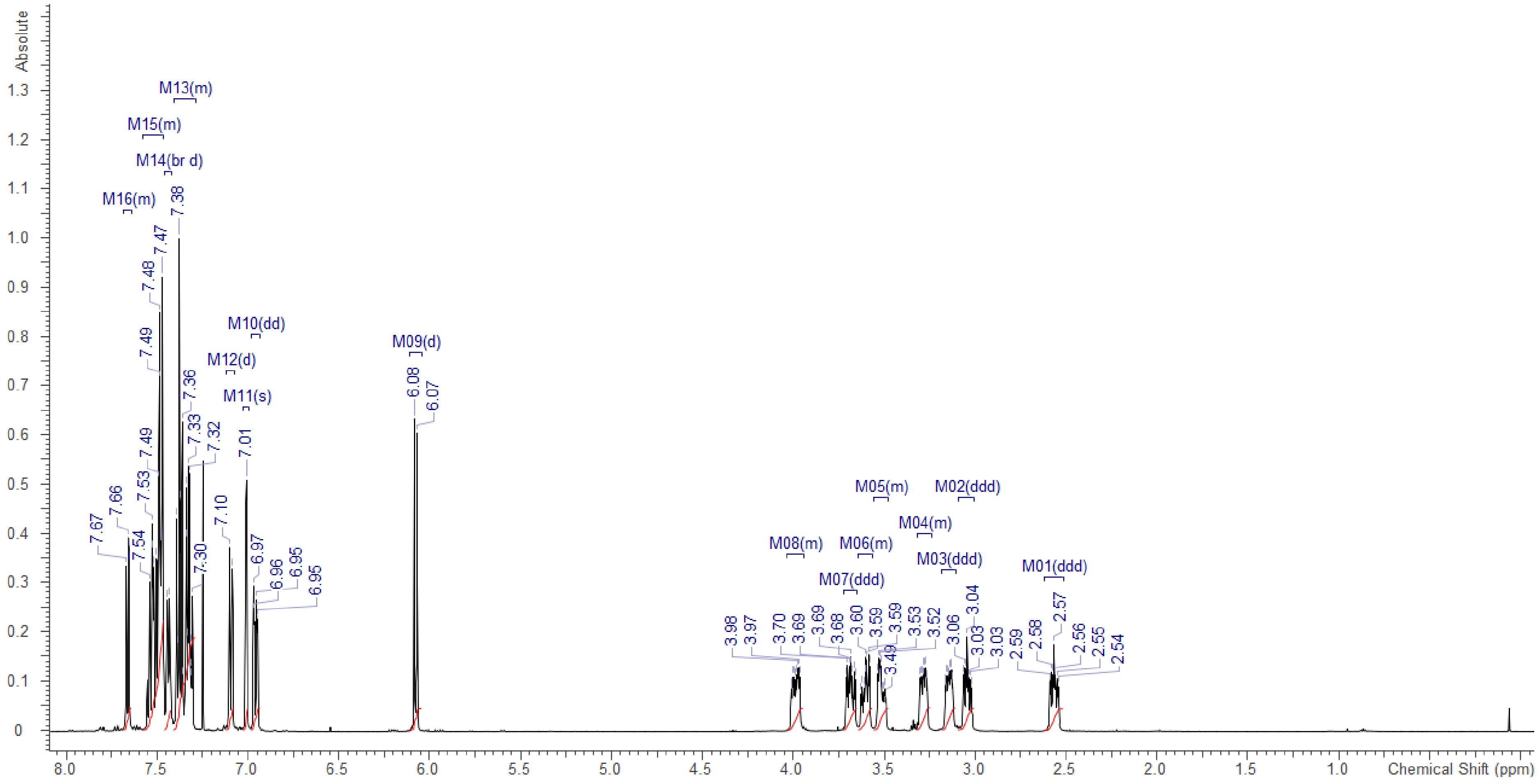

***N*-(2-oxo-1-phenyl-2-(4-(3-(trifluoromethyl)phenyl)piperazin-1-yl)ethyl)-2 (trifluoromethyl)benzamide (7) – ^{13}C NMR**

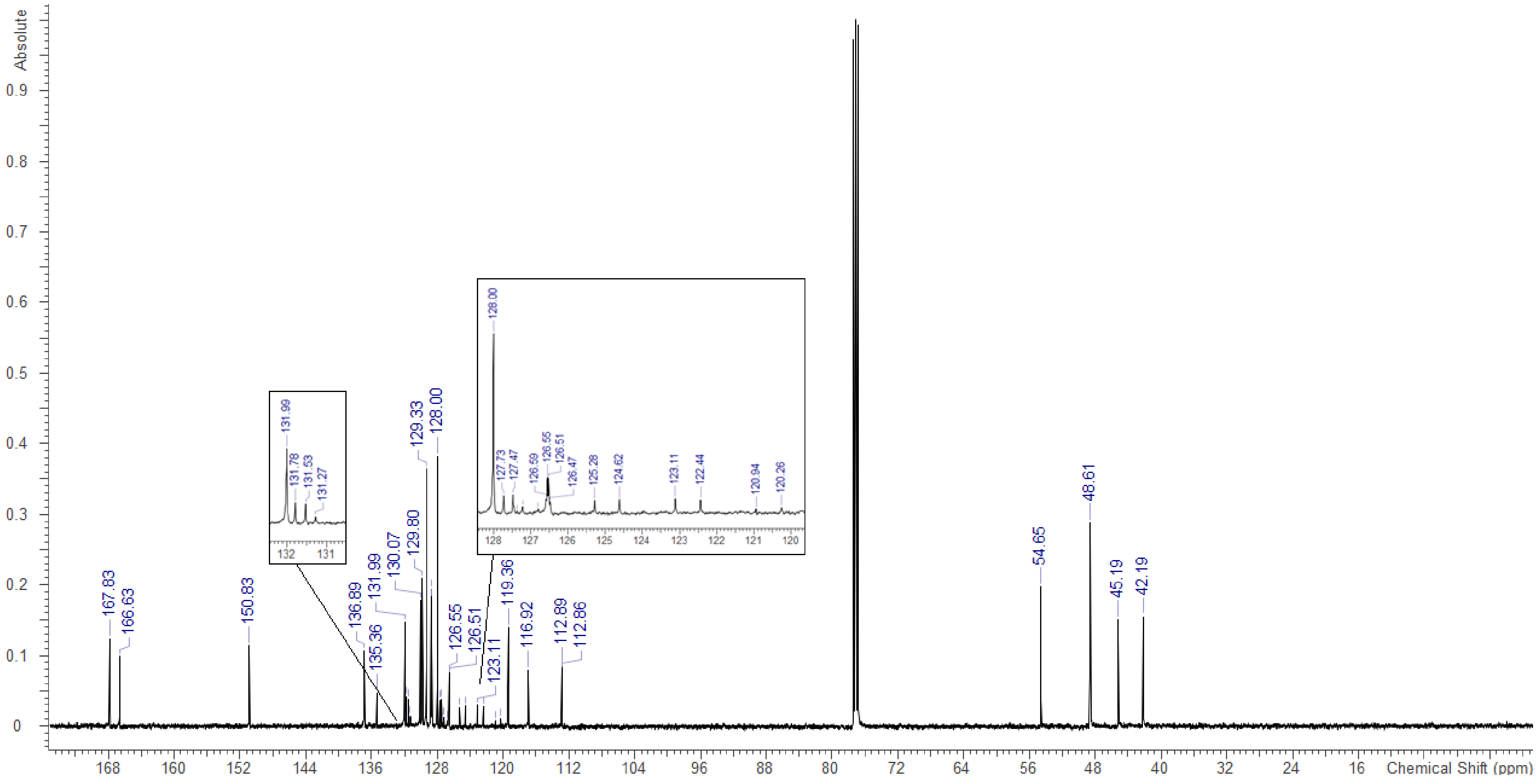

***N*-(2-oxo-1-phenyl-2-(4-(3-(trifluoromethyl)phenyl)piperazin-1-yl)ethyl)-3-(trifluoromethyl)benzamide (8) – ^{1}H NMR**

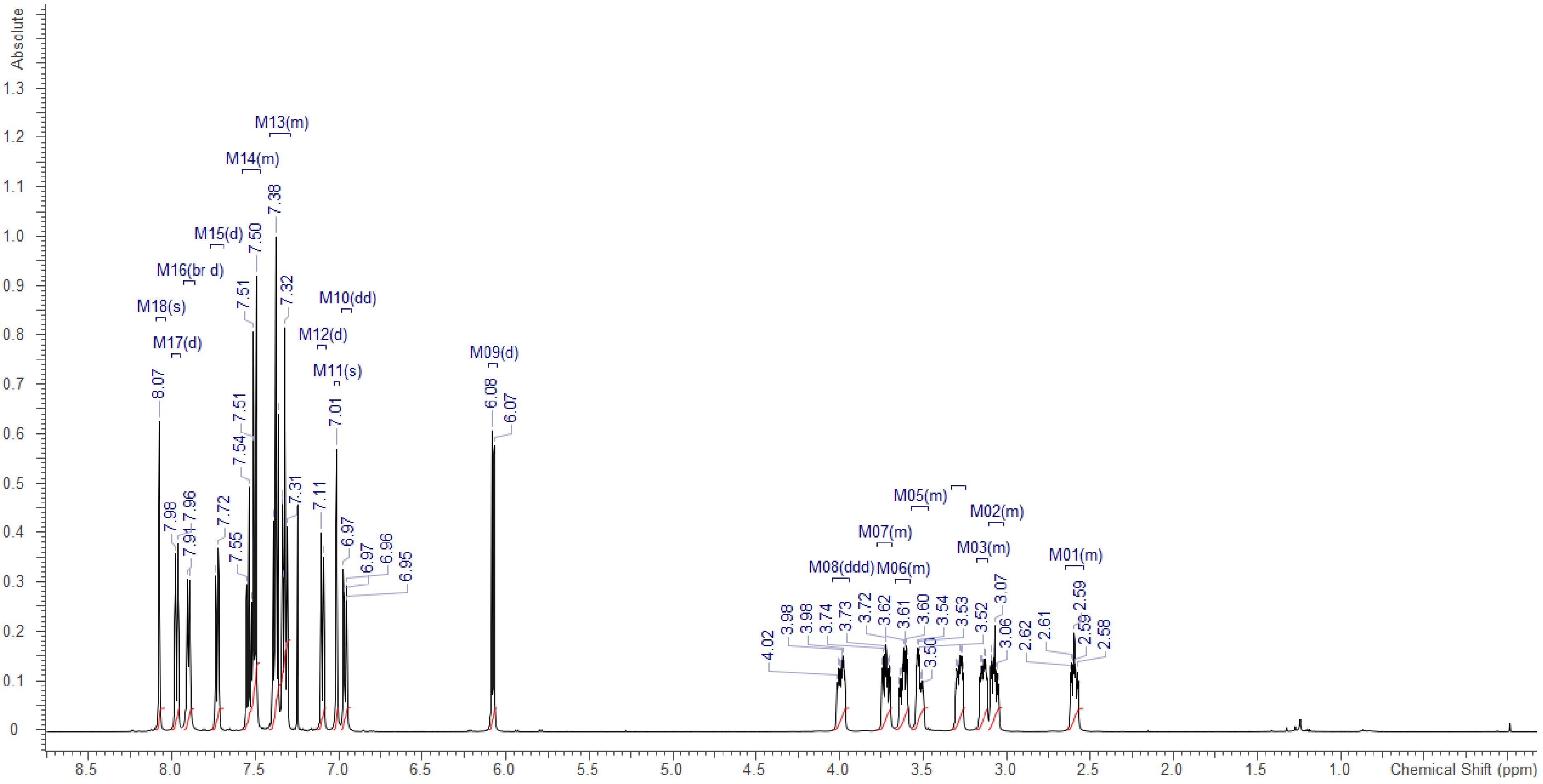

***N*-(2-oxo-1-phenyl-2-(4-(3-(trifluoromethyl)phenyl)piperazin-1-yl)ethyl)-3-(trifluoromethyl)benzamide (8) – ^{13}C NMR**

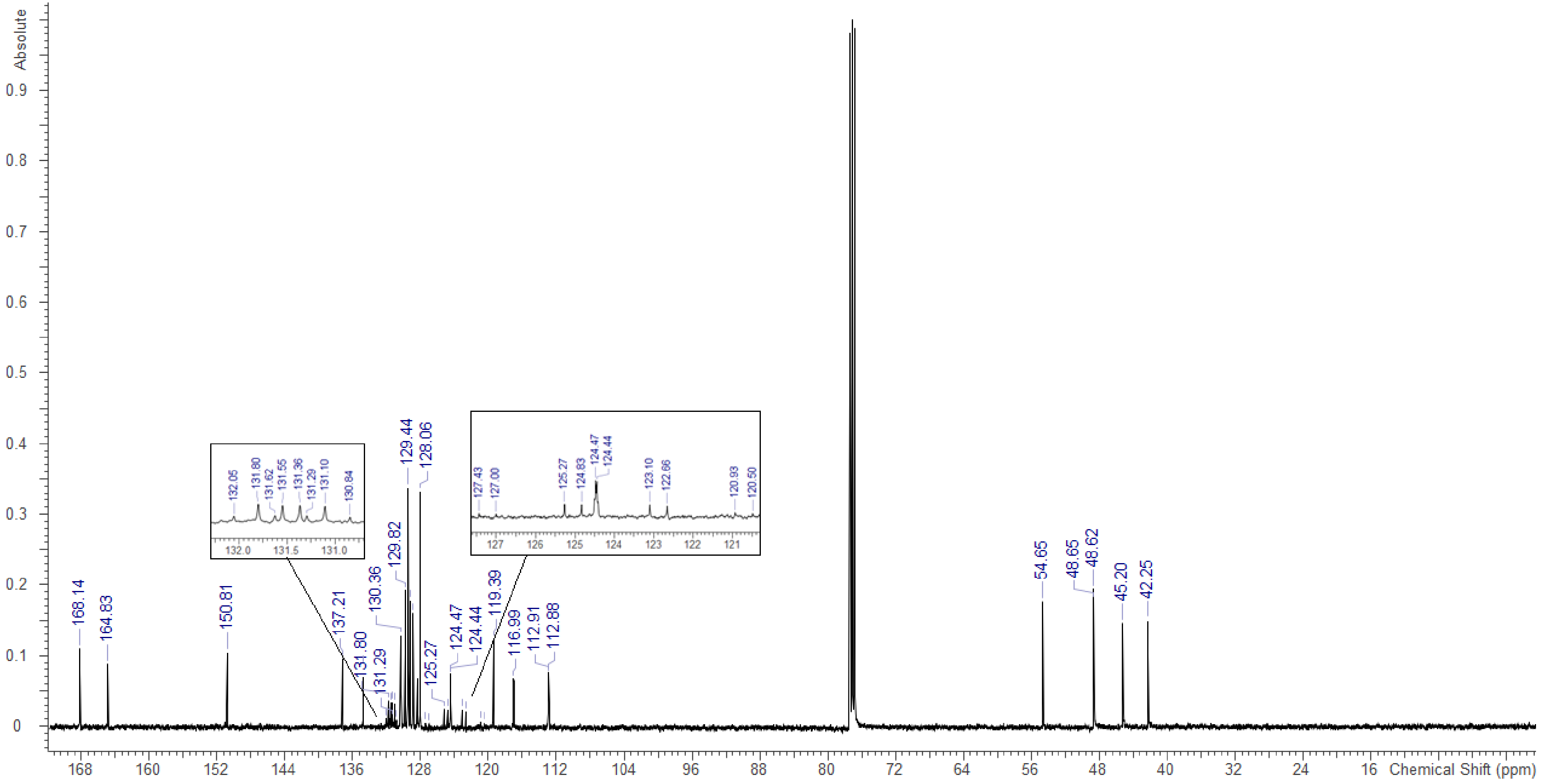

***N*-(2-oxo-1-phenyl-2-(4-(3-(trifluoromethyl)phenyl)piperazin-1-yl)ethyl)-4-(trifluoromethyl)benzamide (9) – ^{1}H NMR**

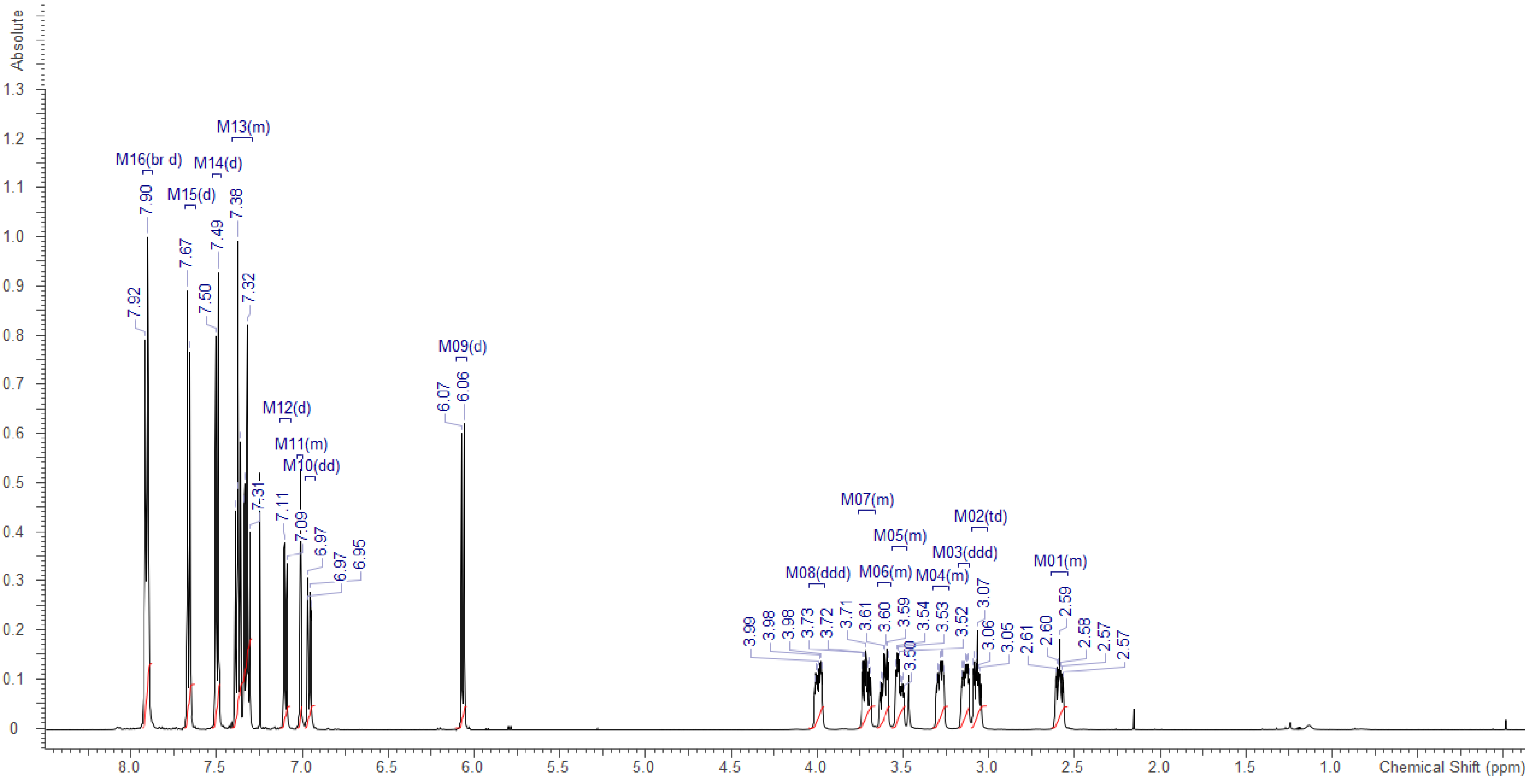

N-(2-oxo-1-phenyl-2-(4-(3-(trifluoromethyl)phenyl)piperazin-1-yl)ethyl)-4-(trifluoromethyl)benzamide (9) – ^{13}C NMR

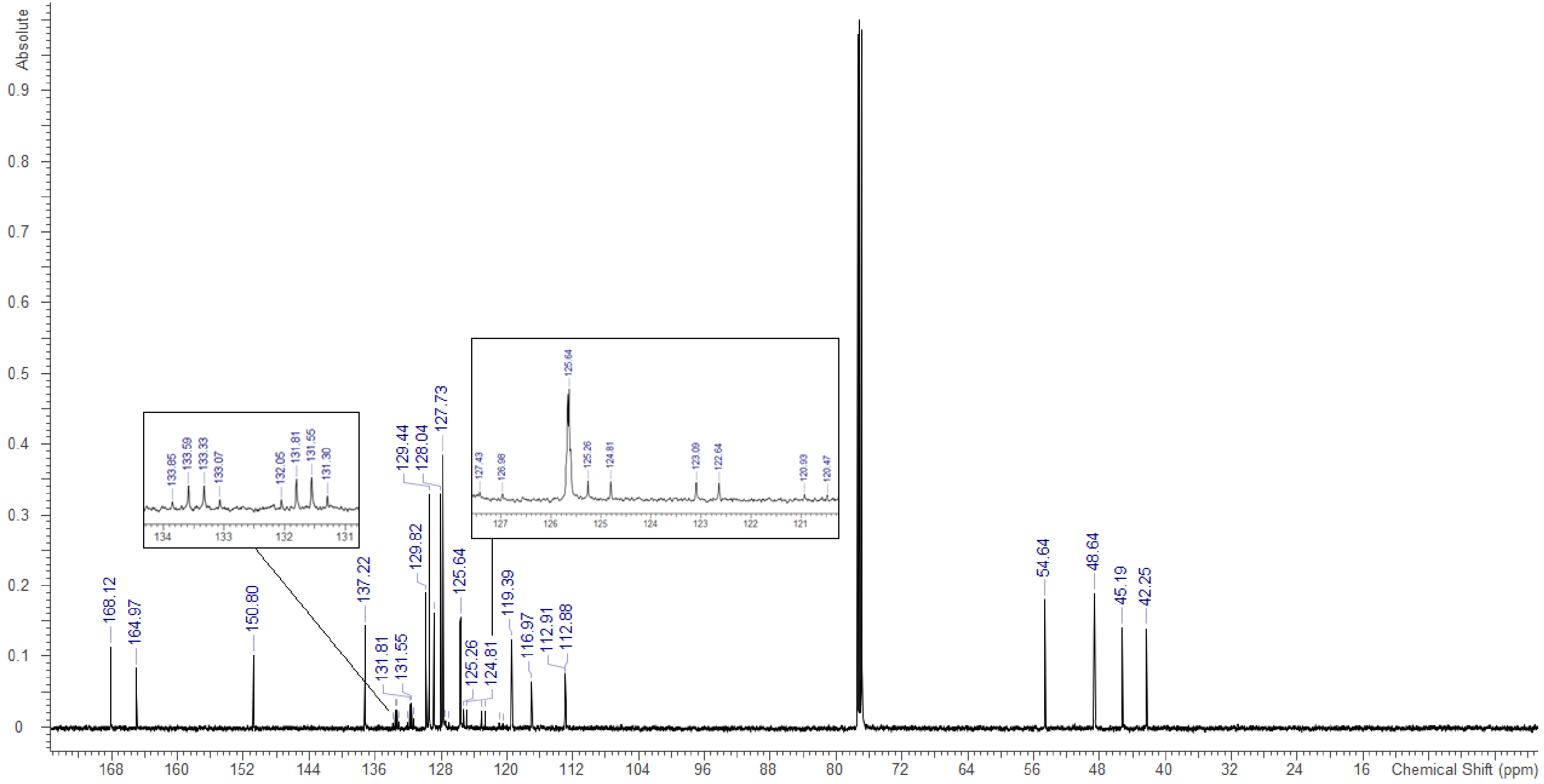

N-(2-oxo-1-phenyl-2-(4-(3-(trifluoromethyl)phenyl)piperazin-1-yl)ethyl)-3-(trifluoromethyl)picolinamide (10) – ^{1}H NMR

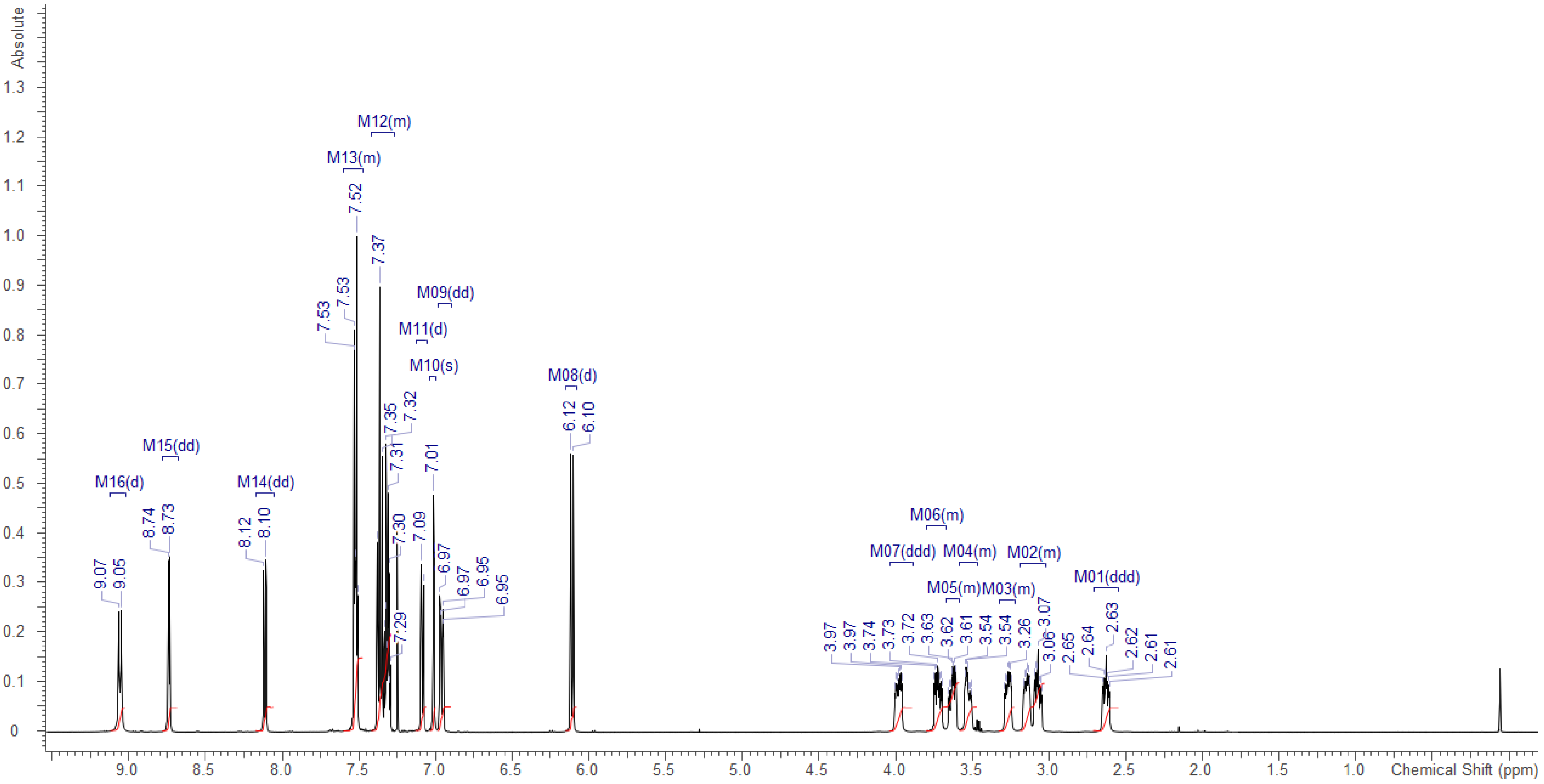

***N*-(2-oxo-1-phenyl-2-(4-(3-(trifluoromethyl)phenyl)piperazin-1-yl)ethyl)-3-(trifluoromethyl)picolinamide (10) – ^{13}C NMR**

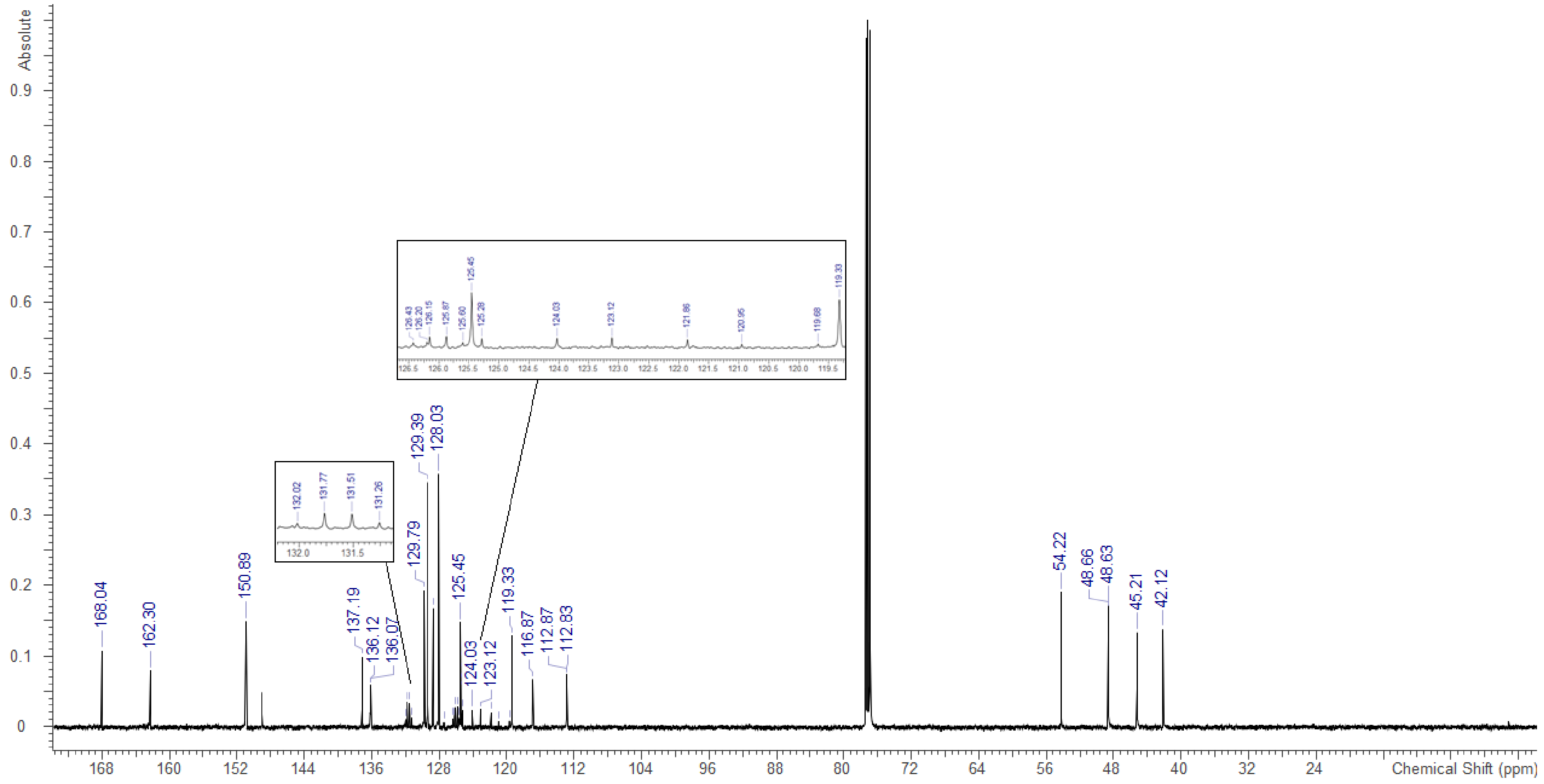

N-(2-oxo-1-phenyl-2-(4-(3-(trifluoromethyl)phenyl)piperazin-1-yl)ethyl)-4-(trifluoromethyl)picolinamide (11) – ^{1}H NMR

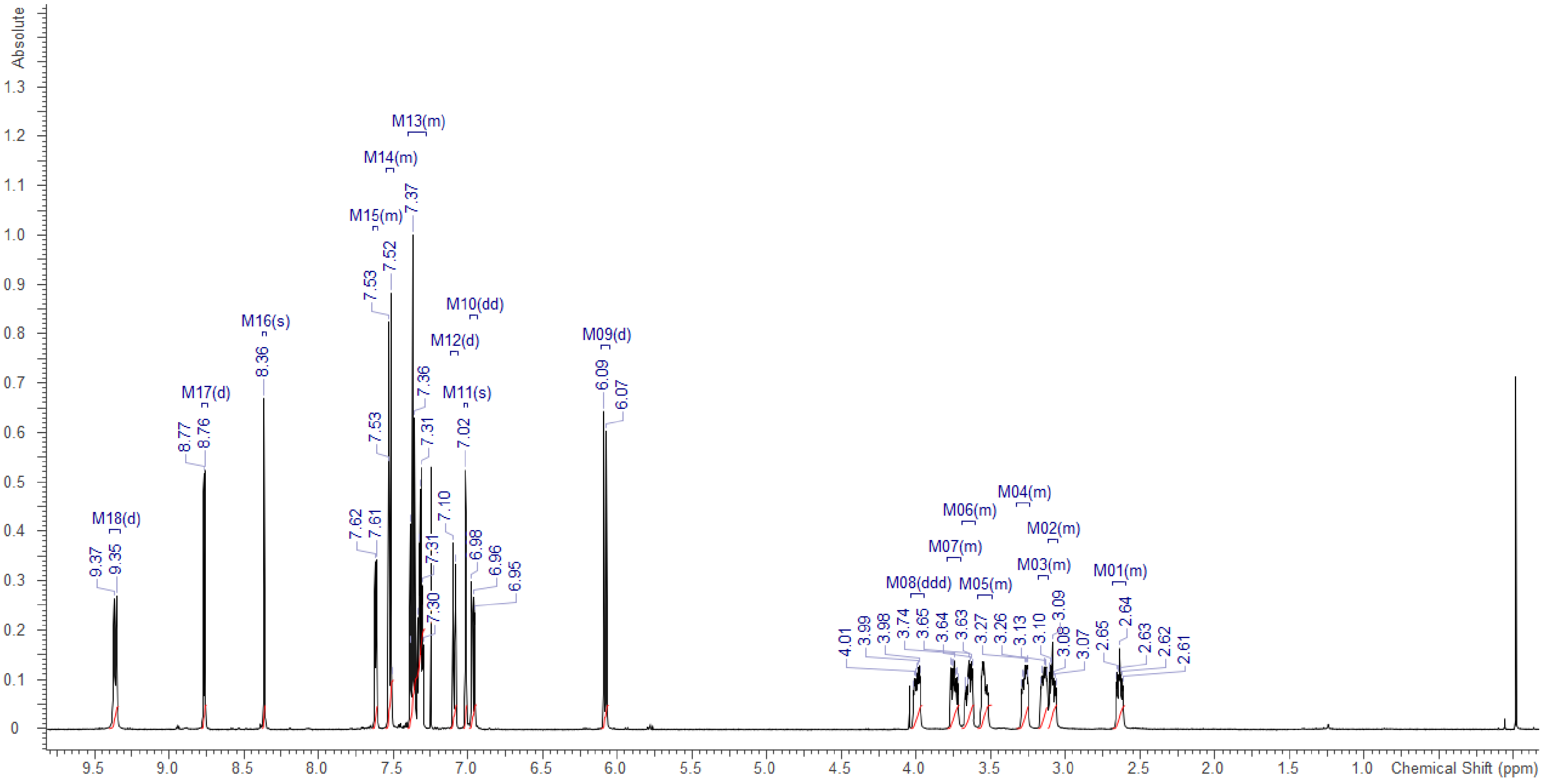

***N*-(2-oxo-1-phenyl-2-(4-(3-(trifluoromethyl)phenyl)piperazin-1-yl)ethyl)-4-(trifluoromethyl)picolinamide (11) – ^{13}C NMR**

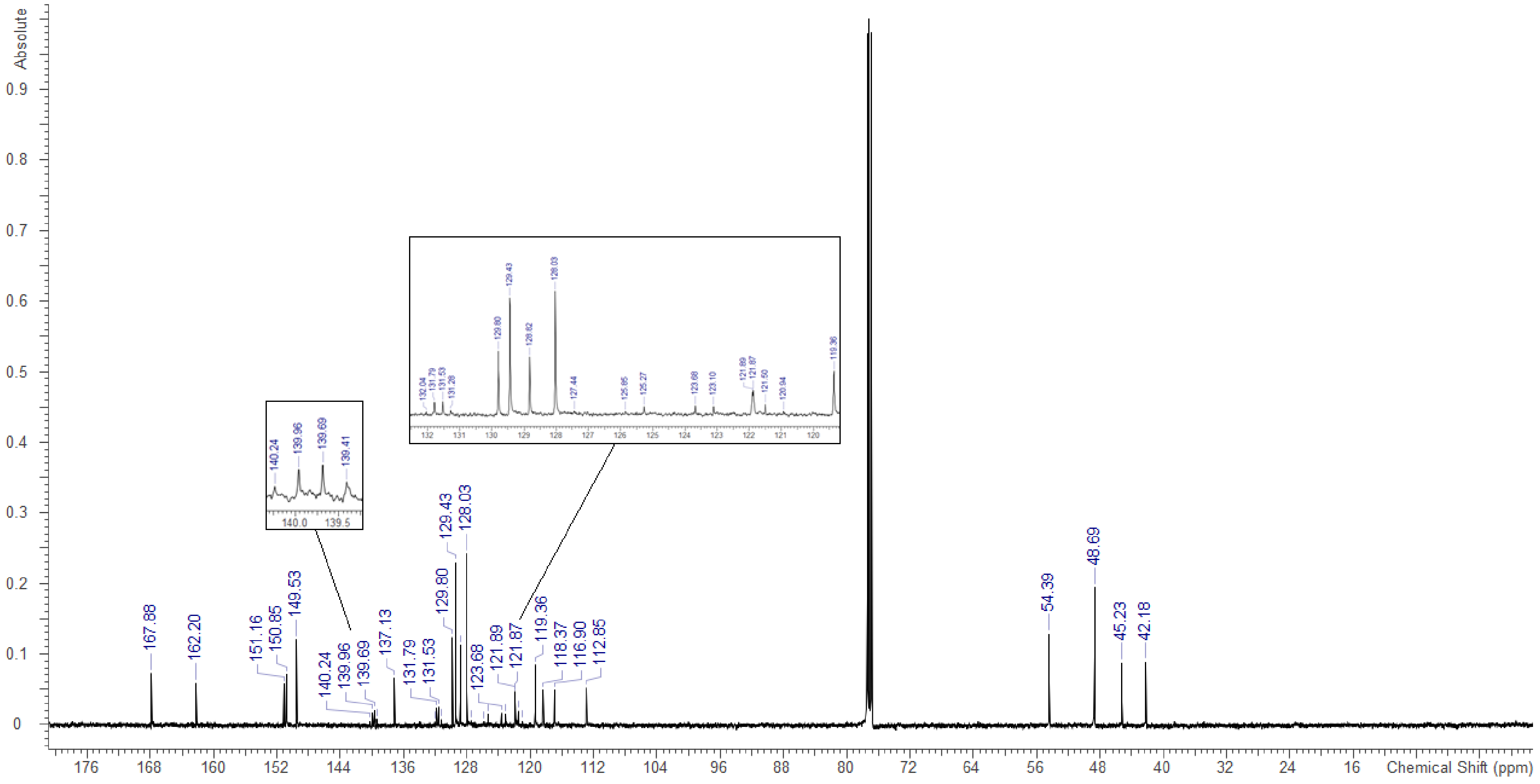

***N*-(2-oxo-1-phenyl-2-(4-(3-(trifluoromethyl)phenyl)piperazin-1-yl)ethyl)-5-(trifluoromethyl)picolinamide (12) – ^{1}H NMR**

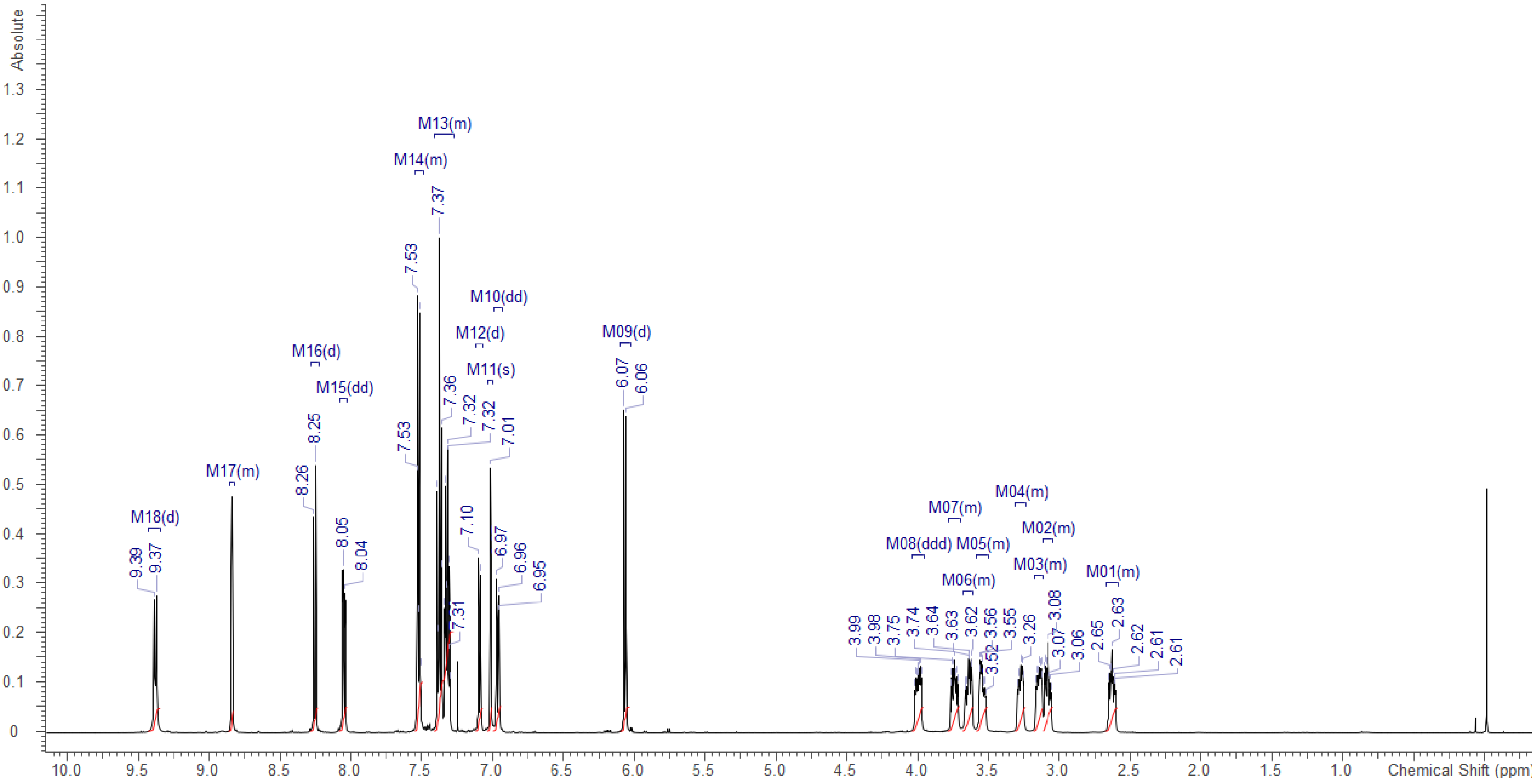

***N*-(2-oxo-1-phenyl-2-(4-(3-(trifluoromethyl)phenyl)piperazin-1-yl)ethyl)-5-(trifluoromethyl)picolinamide (12) – ^{13}C NMR**

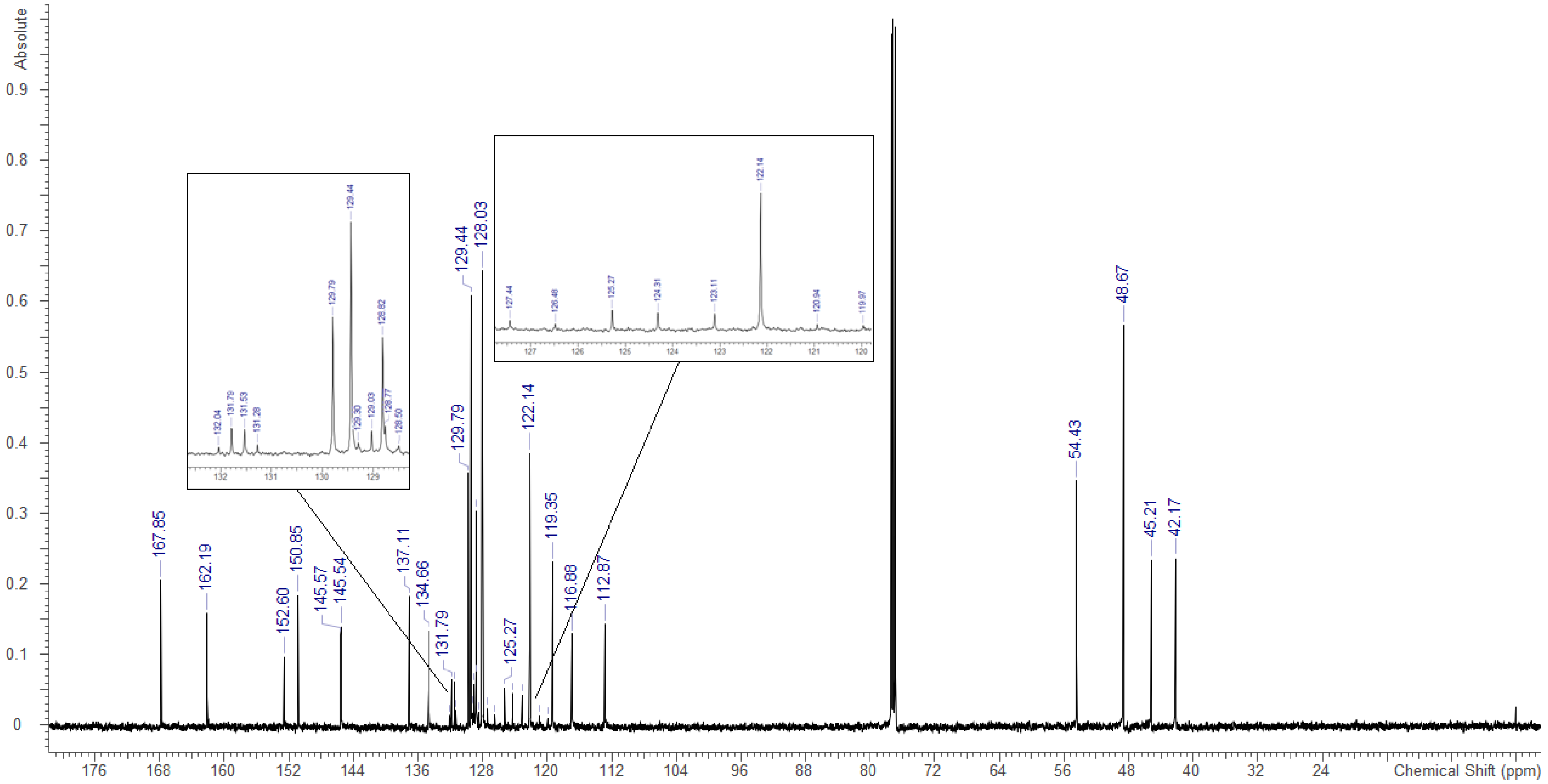

2-Acetamido-2-phenyl-*N*-(1-phenylpyrrolidin-3-yl)acetamide (21) – ^{1}H NMR

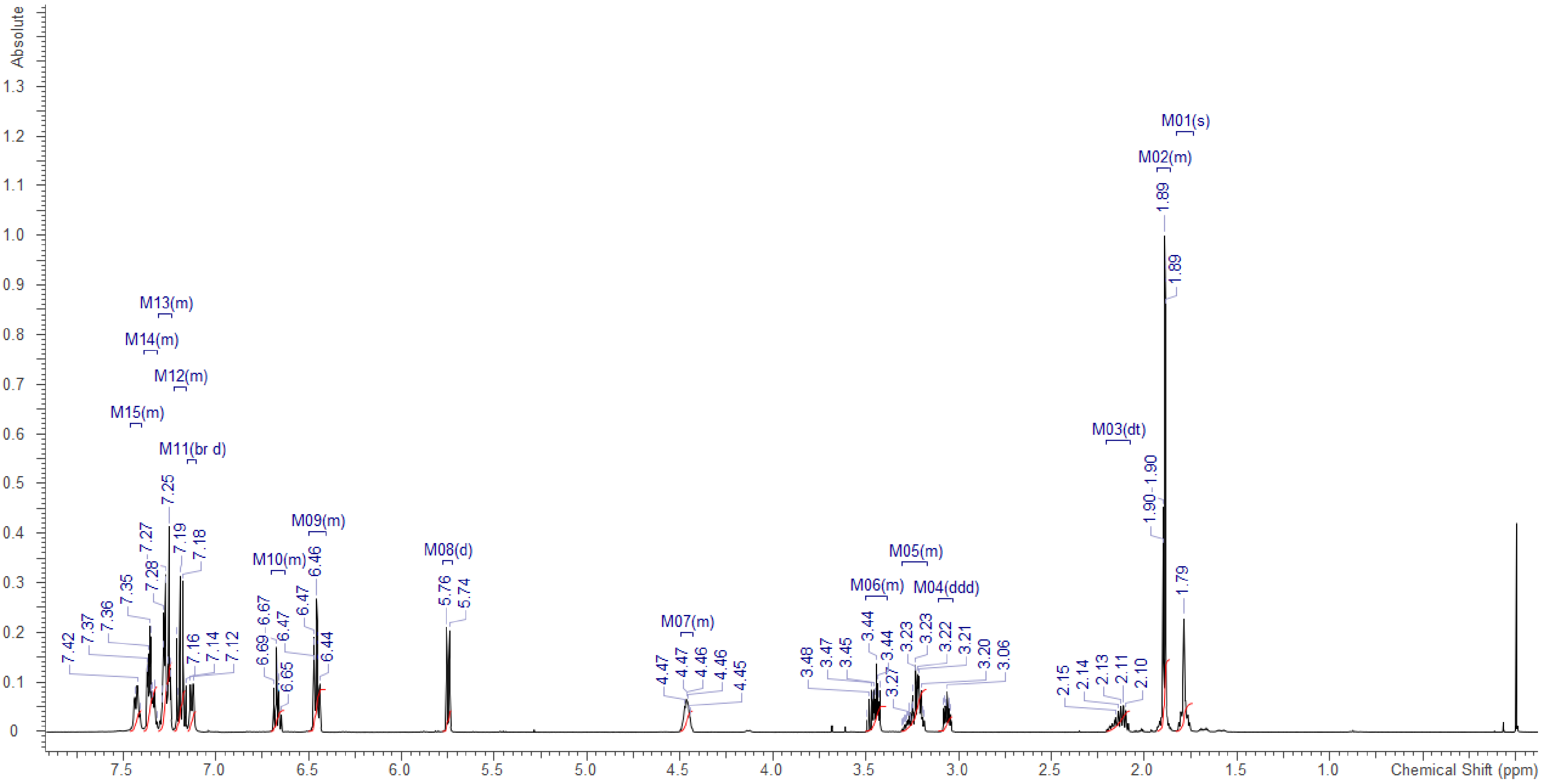

2-Acetamido-2-phenyl-*N*-(1-phenylpyrrolidin-3-yl)acetamide (21) – ^{13}C NMR

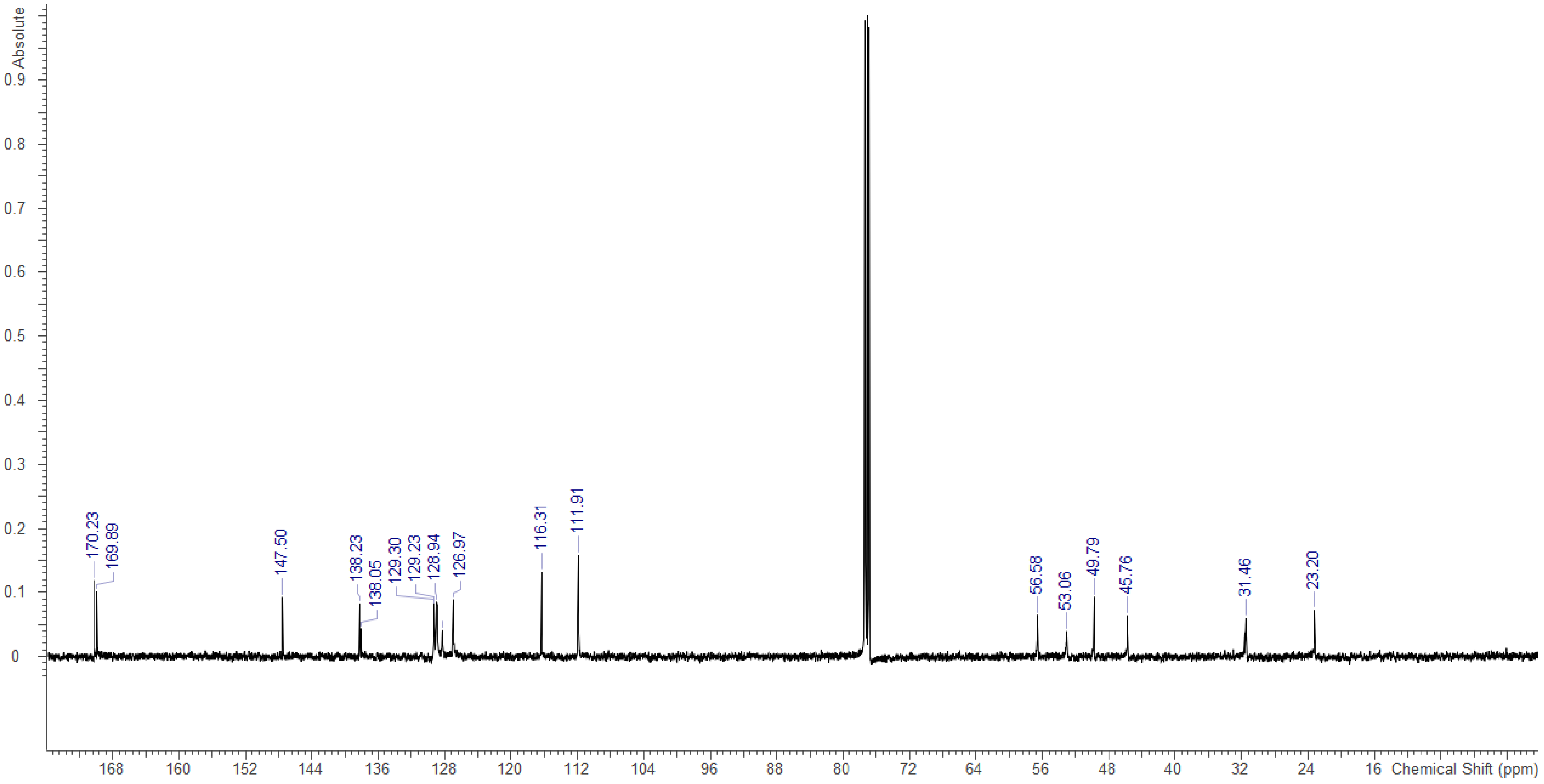

2-Acetamido-2-phenyl-*N*-(1-(3-(trifluoromethyl)phenyl)pyrrolidin-3-yl)acetamide (22) – ^{1}H NMR

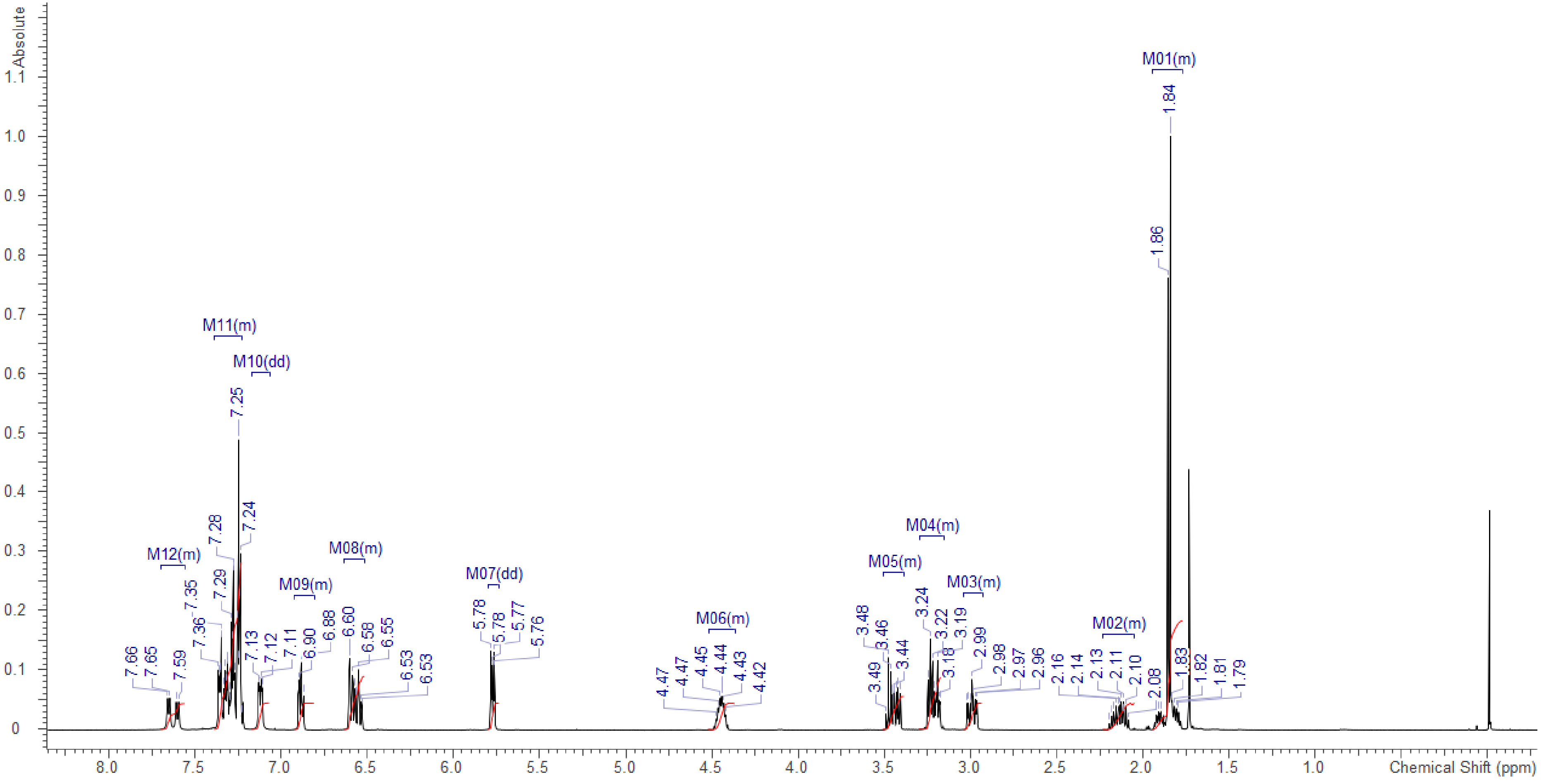

2-Acetamido-2-phenyl-*N*-(1-(3-(trifluoromethyl)phenyl)pyrrolidin-3-yl)acetamide (22) – ^{13}C NMR

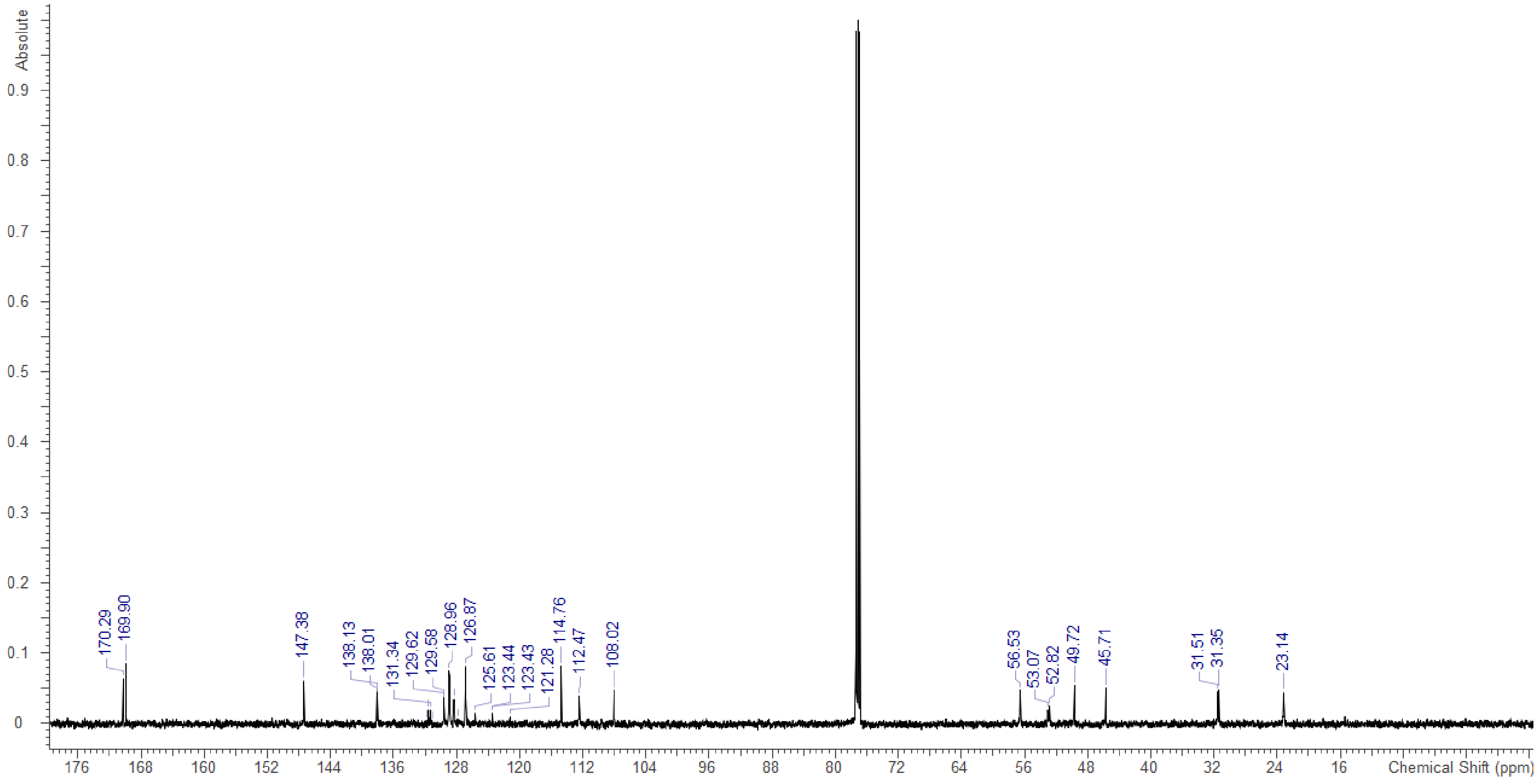

2-Acetamido-2-phenyl-*N*-(1-(3-(trifluoromethoxy)phenyl)pyrrolidin-3-yl)acetamide (23) – ^{1}H NMR

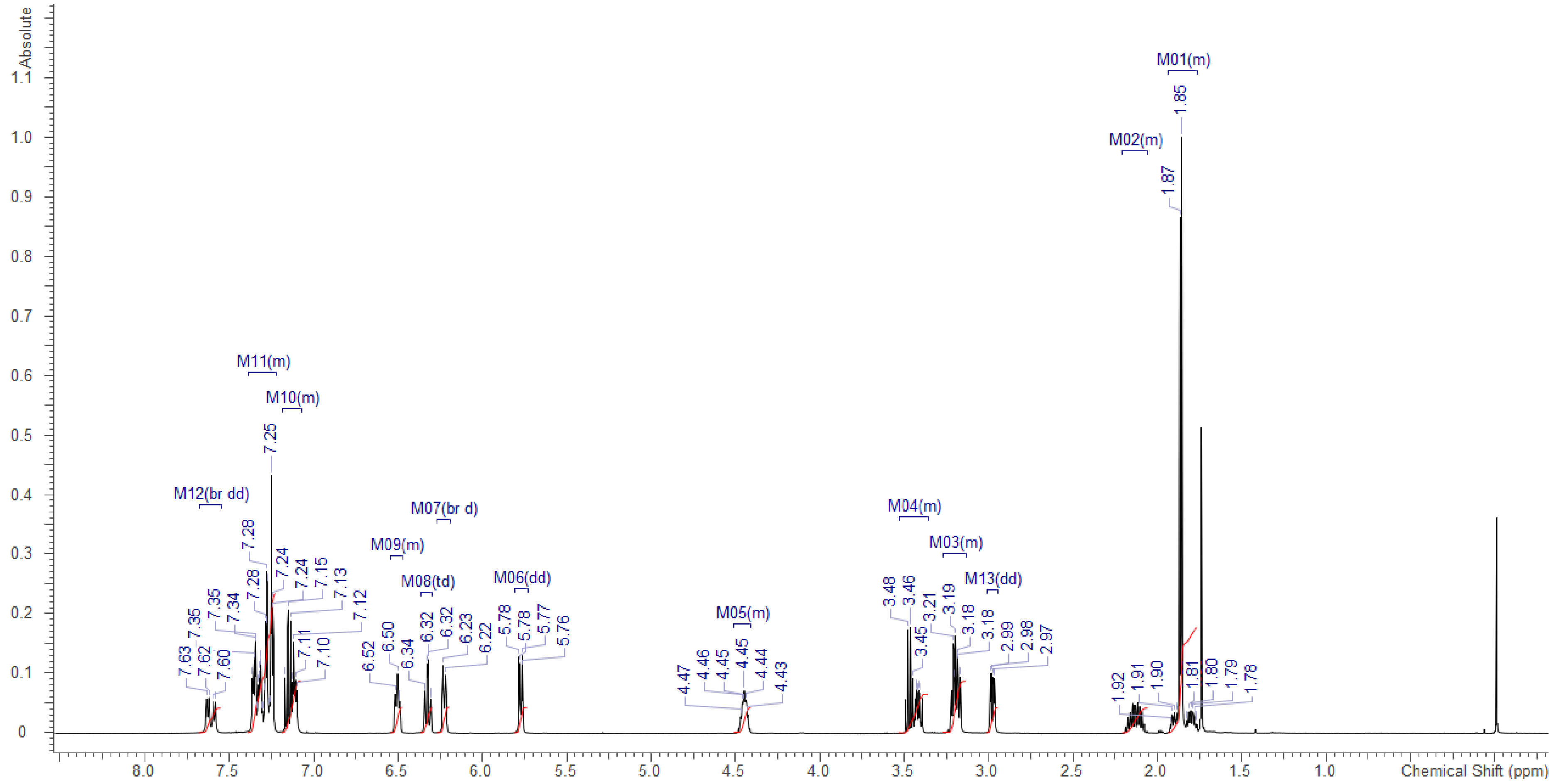

2-Acetamido-2-phenyl-*N*-(1-(3-(trifluoromethoxy)phenyl)pyrrolidin-3-yl)acetamide (23) – ^{13}C NMR

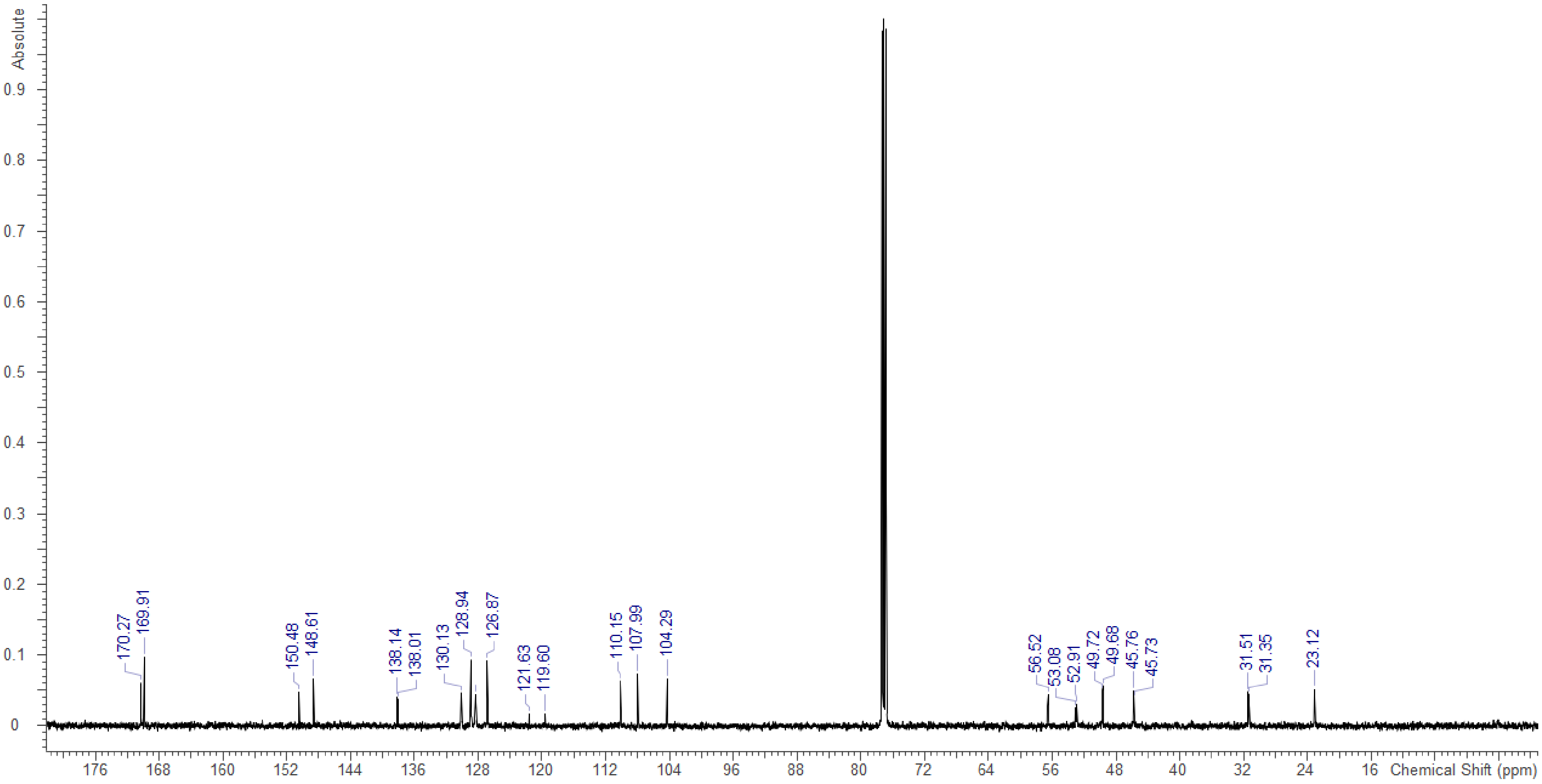

2-Acetamido-2-phenyl-N-(1-(3-((trifluoromethyl)thio)phenyl)pyrrolidin-3-yl)acetamide (24) – ^{1}H NMR

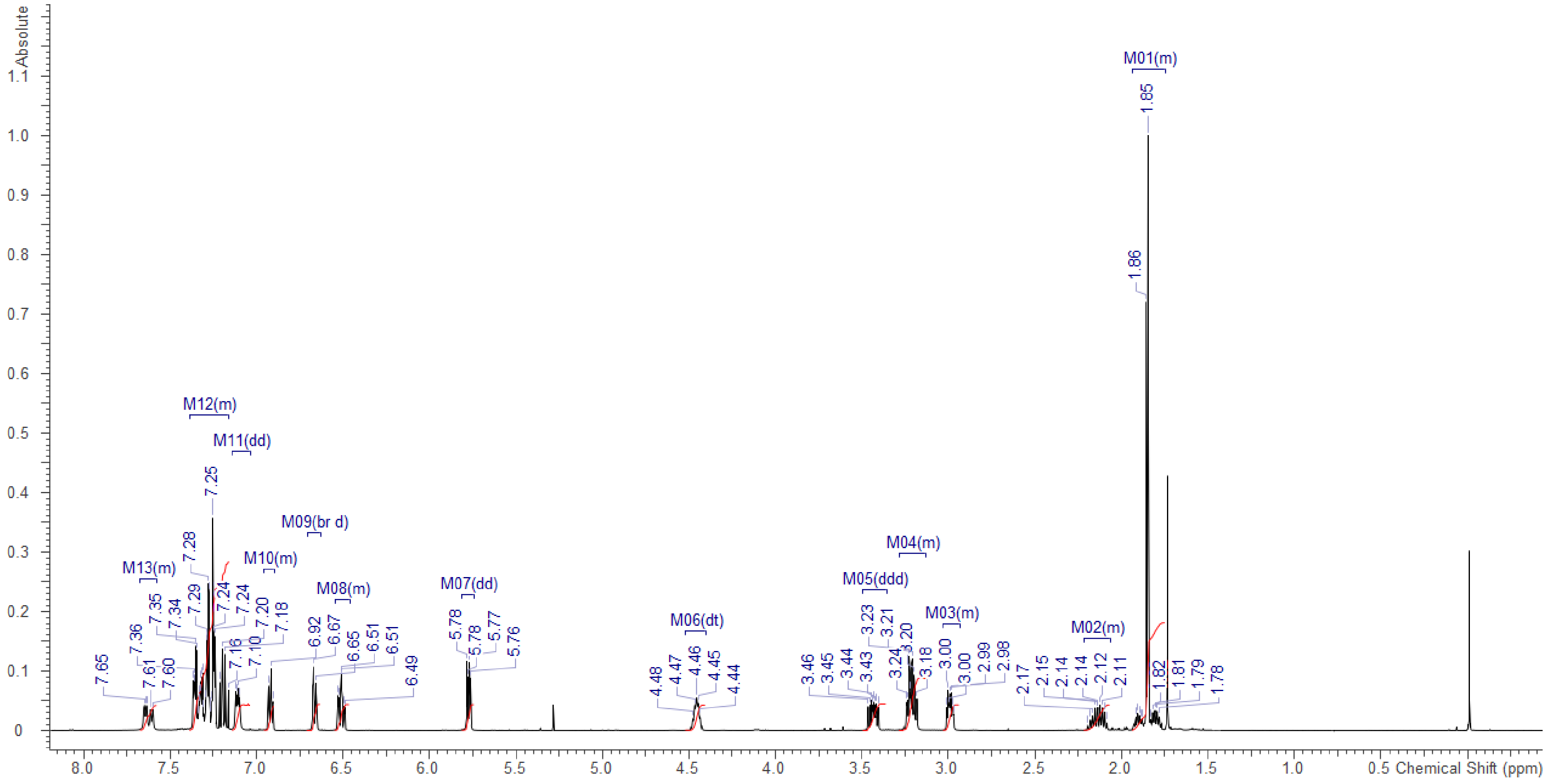

2-Acetamido-2-phenyl-N-(1-(3-((trifluoromethyl)thio)phenyl)pyrrolidin-3-yl)acetamide (24) – ^{13}C NMR

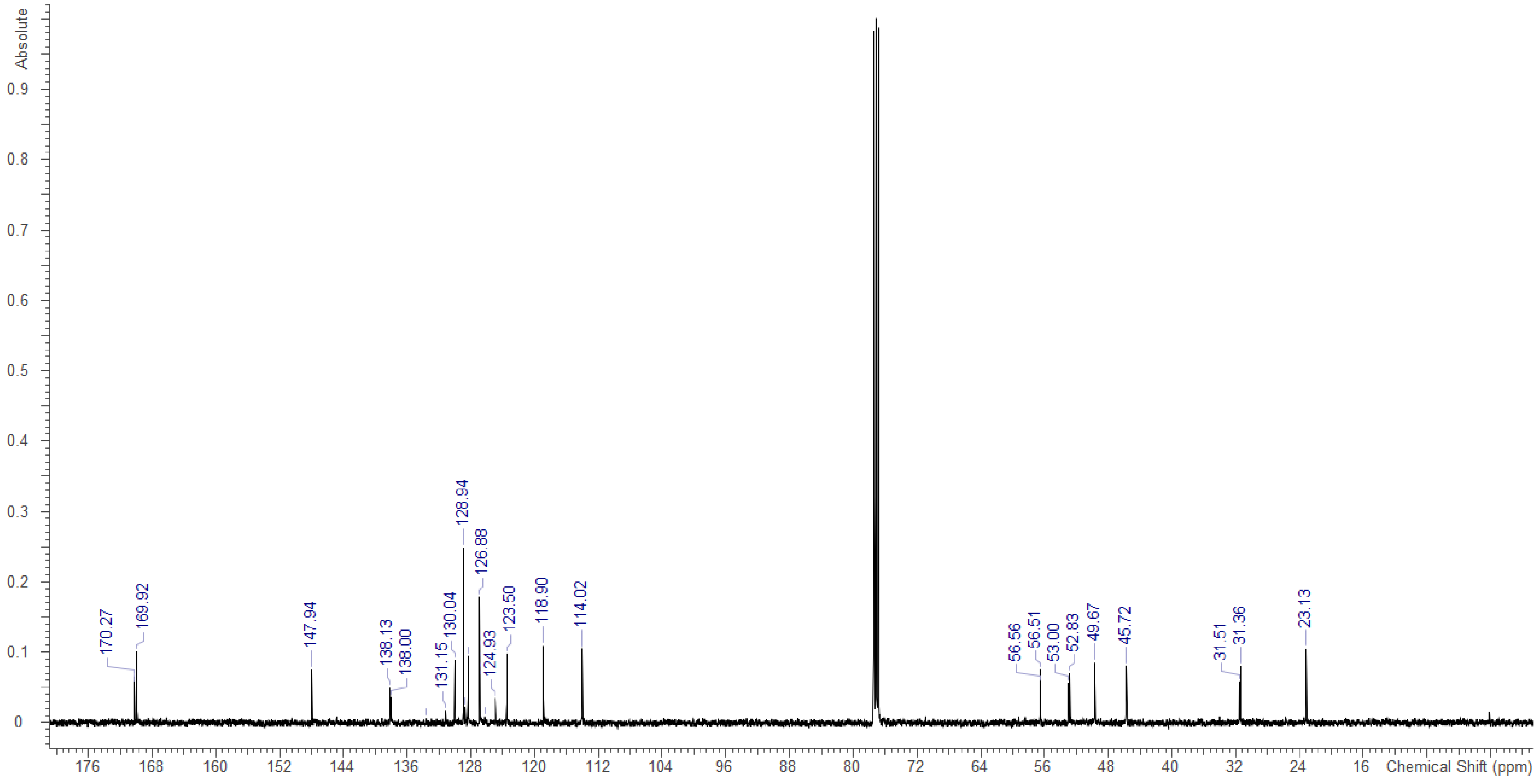

(*R*)-*N*-(2-oxo-1-phenyl-2-(4-(3-(trifluoromethyl)phenyl)piperazin-1-yl)ethyl)acetamide ((*R*)-31) – ^{1}H NMR

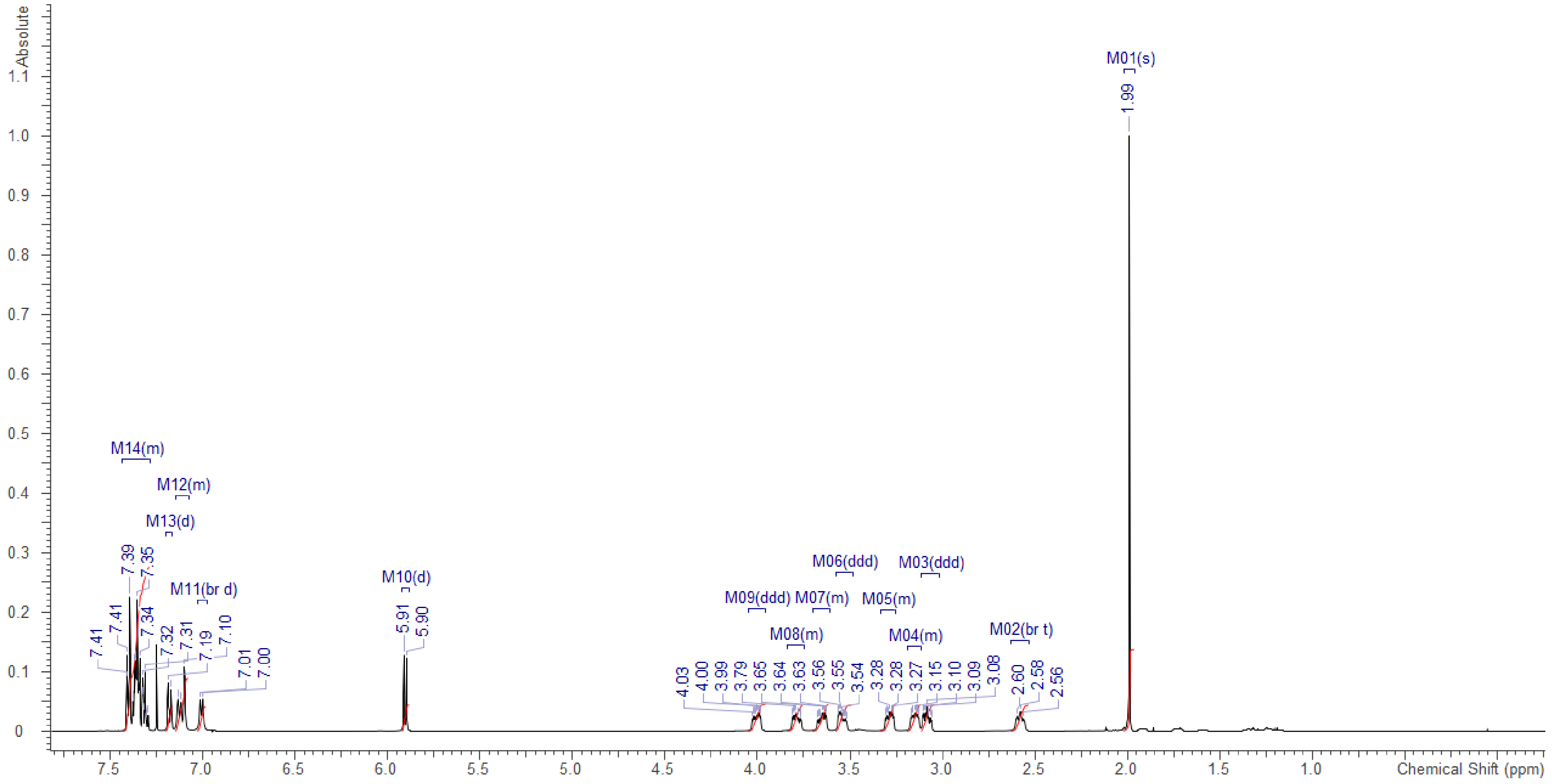

(*R*)-*N*-(2-oxo-1-phenyl-2-(4-(3-(trifluoromethyl)phenyl)piperazin-1-yl)ethyl)acetamide ((*R*)-31) – ^{13}C NMR

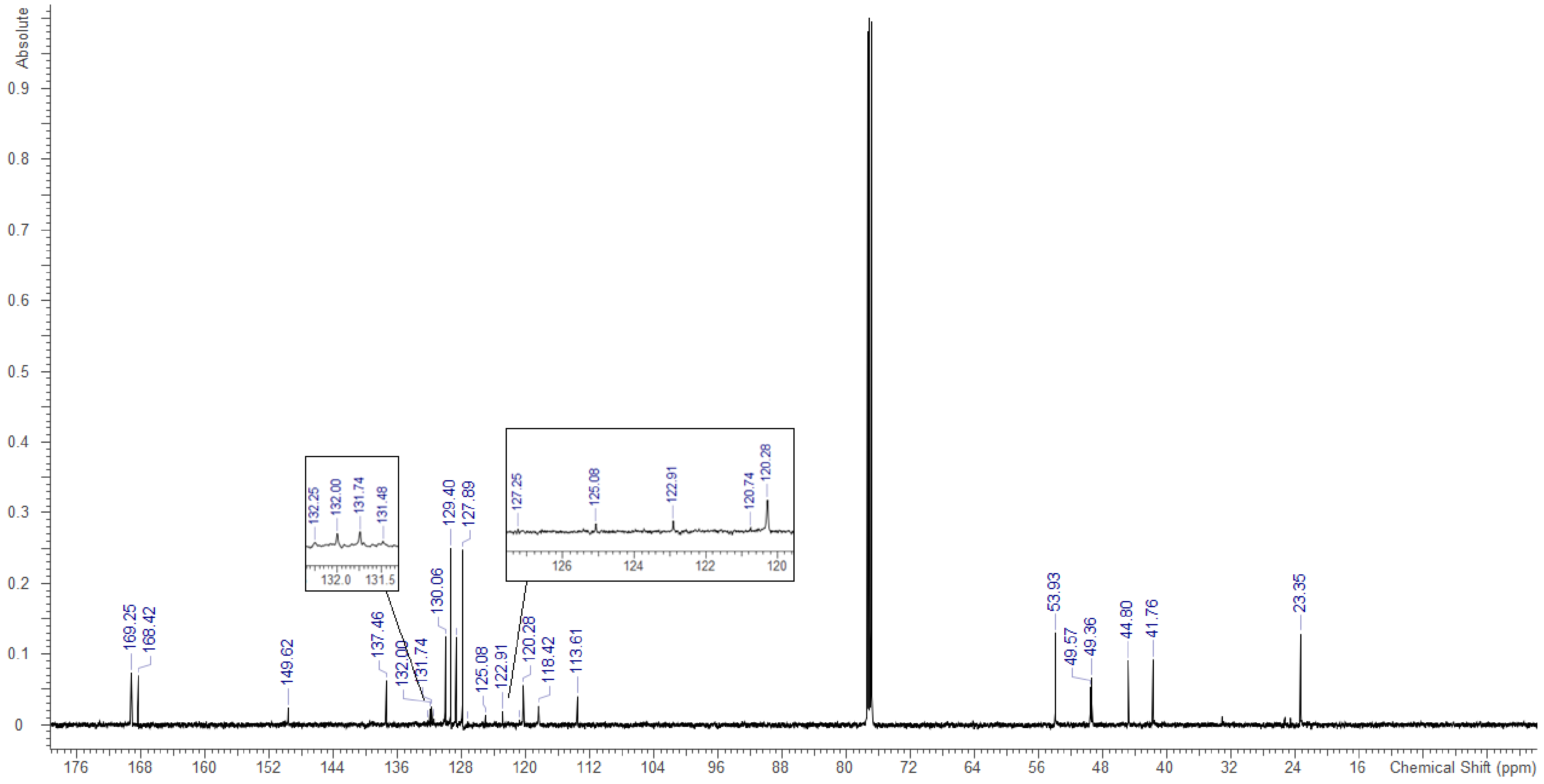

(*S*)-*N*-(2-oxo-1-phenyl-2-(4-(3-(trifluoromethyl)phenyl)piperazin-1-yl)ethyl)acetamide ((*S*)-31) – ^{1}H NMR

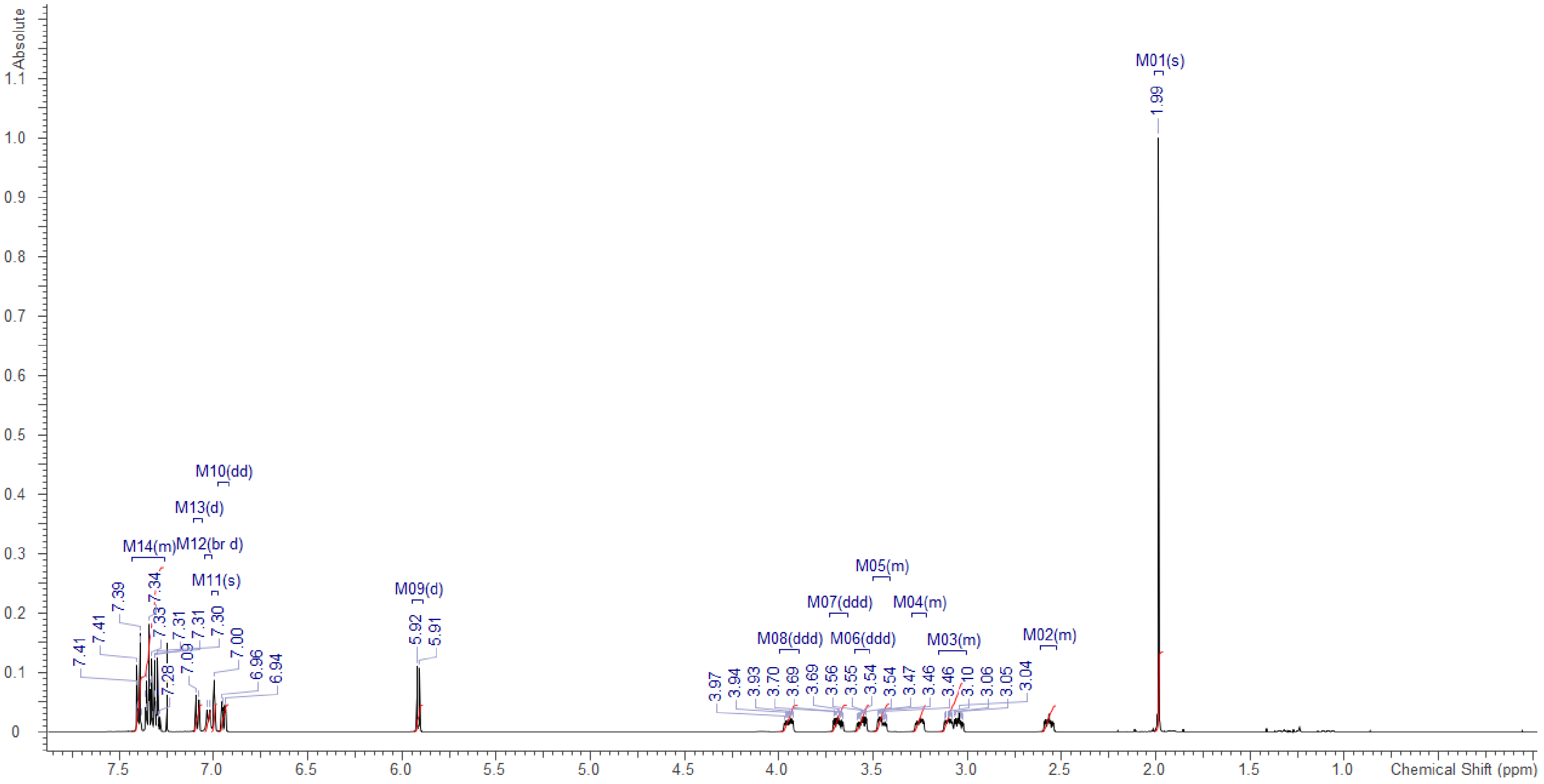

(*S*)-*N*-(2-oxo-1-phenyl-2-(4-(3-(trifluoromethyl)phenyl)piperazin-1-yl)ethyl)acetamide ((*S*)-31) – ^{13}C NMR

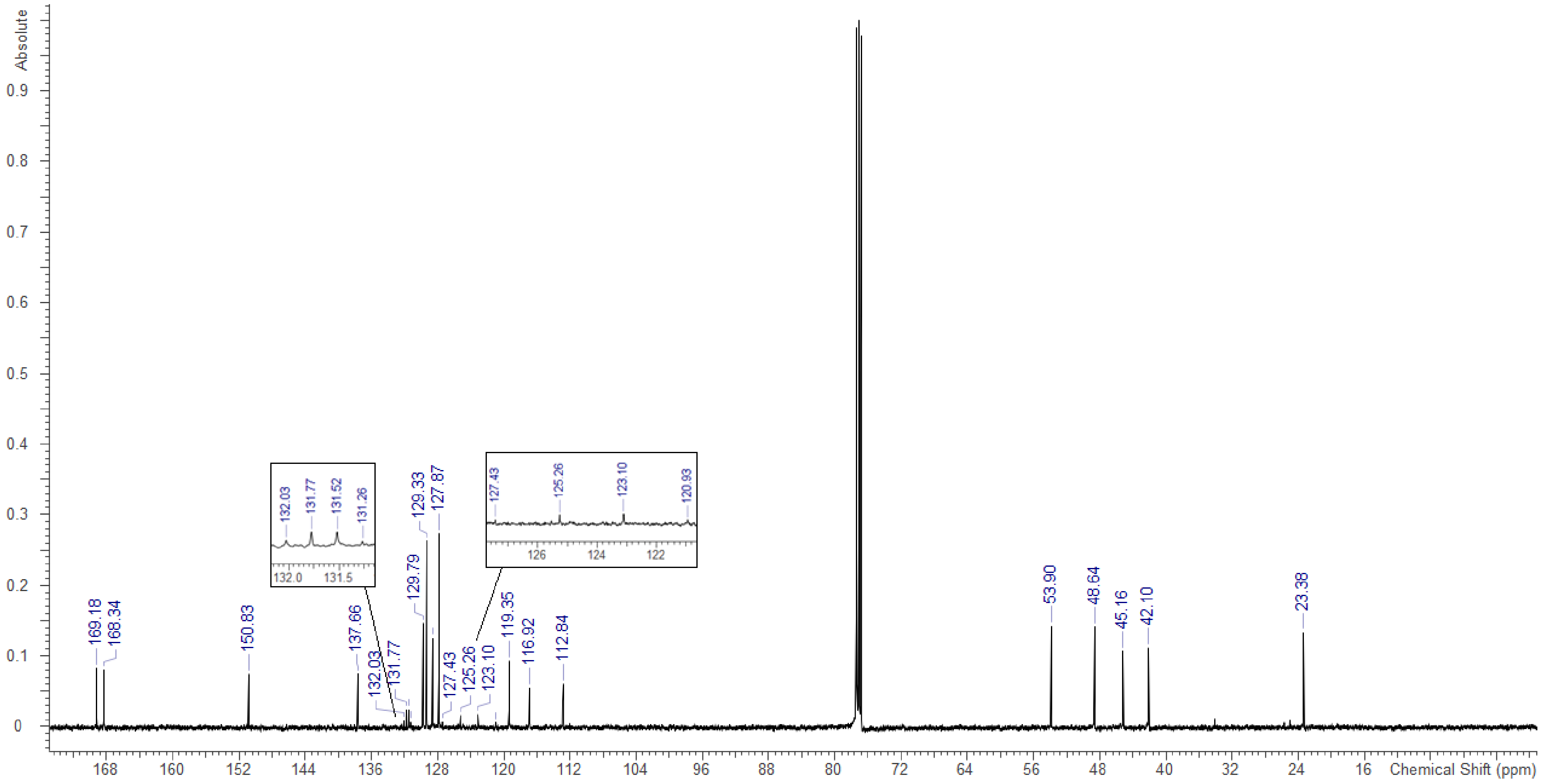

(*R*)-*N*-(2-oxo-1-phenyl-2-(4-(3-(trifluoromethoxy)phenyl)piperazin-1-yl)ethyl)acetamide ((*R*)-32) – ^{1}H NMR

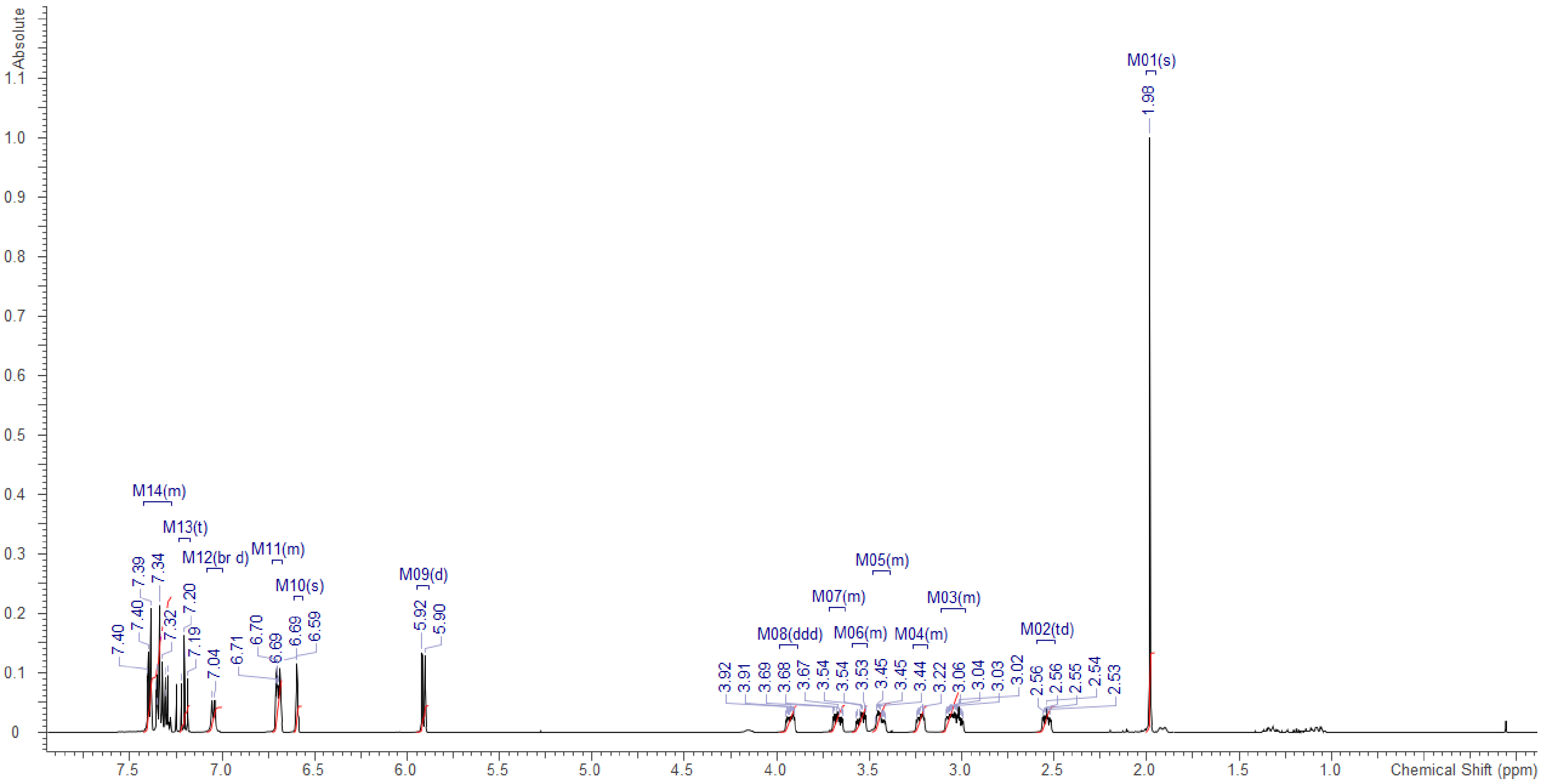

(*R*)-*N*-(2-oxo-1-phenyl-2-(4-(3-(trifluoromethoxy)phenyl)piperazin-1-yl)ethyl)acetamide ((*R*)-32) – ^{13}C NMR

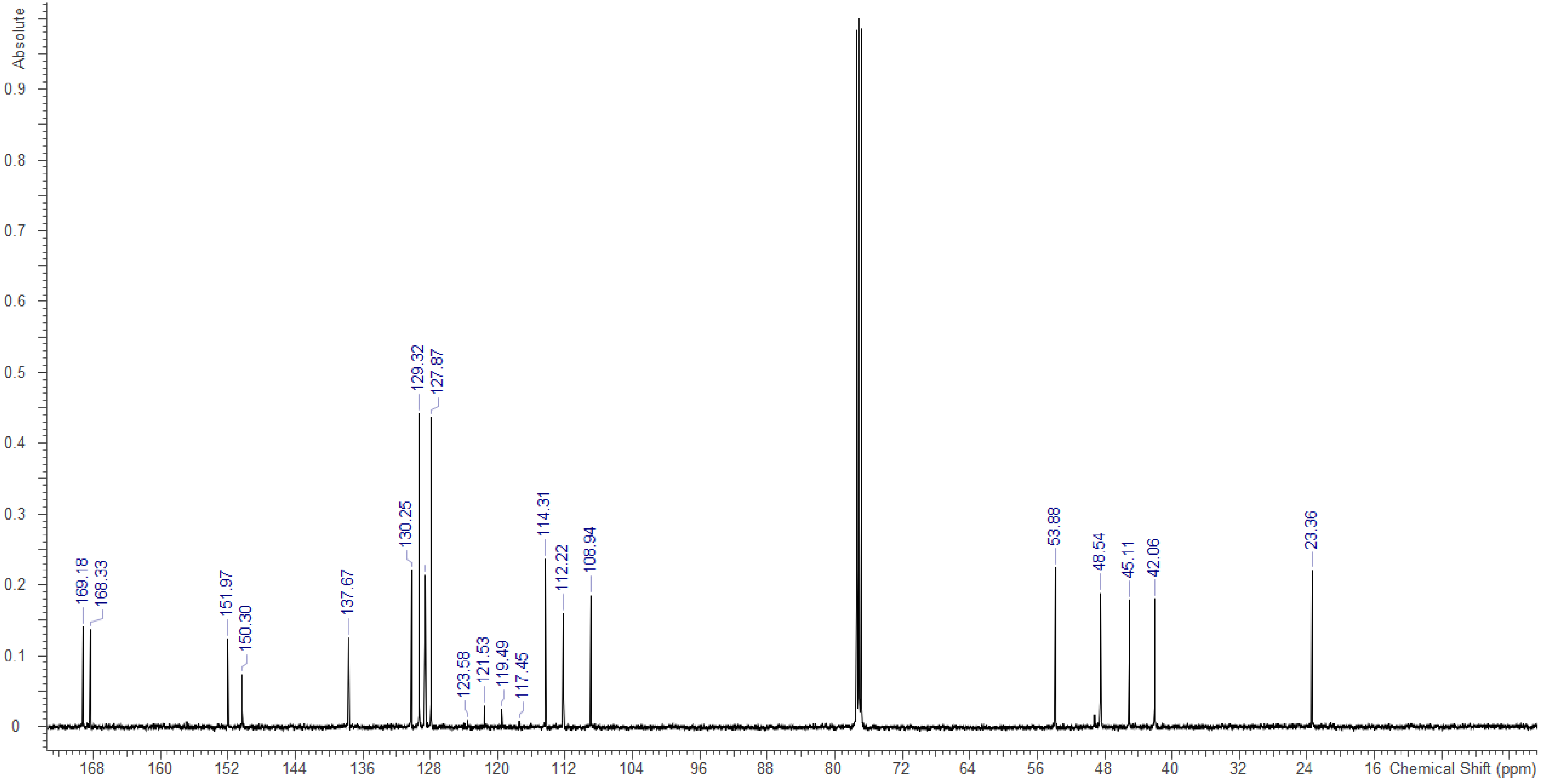

(*S*)-*N*-(2-oxo-1-phenyl-2-(4-(3-(trifluoromethoxy)phenyl)piperazin-1-yl)ethyl)acetamide ((*S*)-32) – ^{1}H NMR

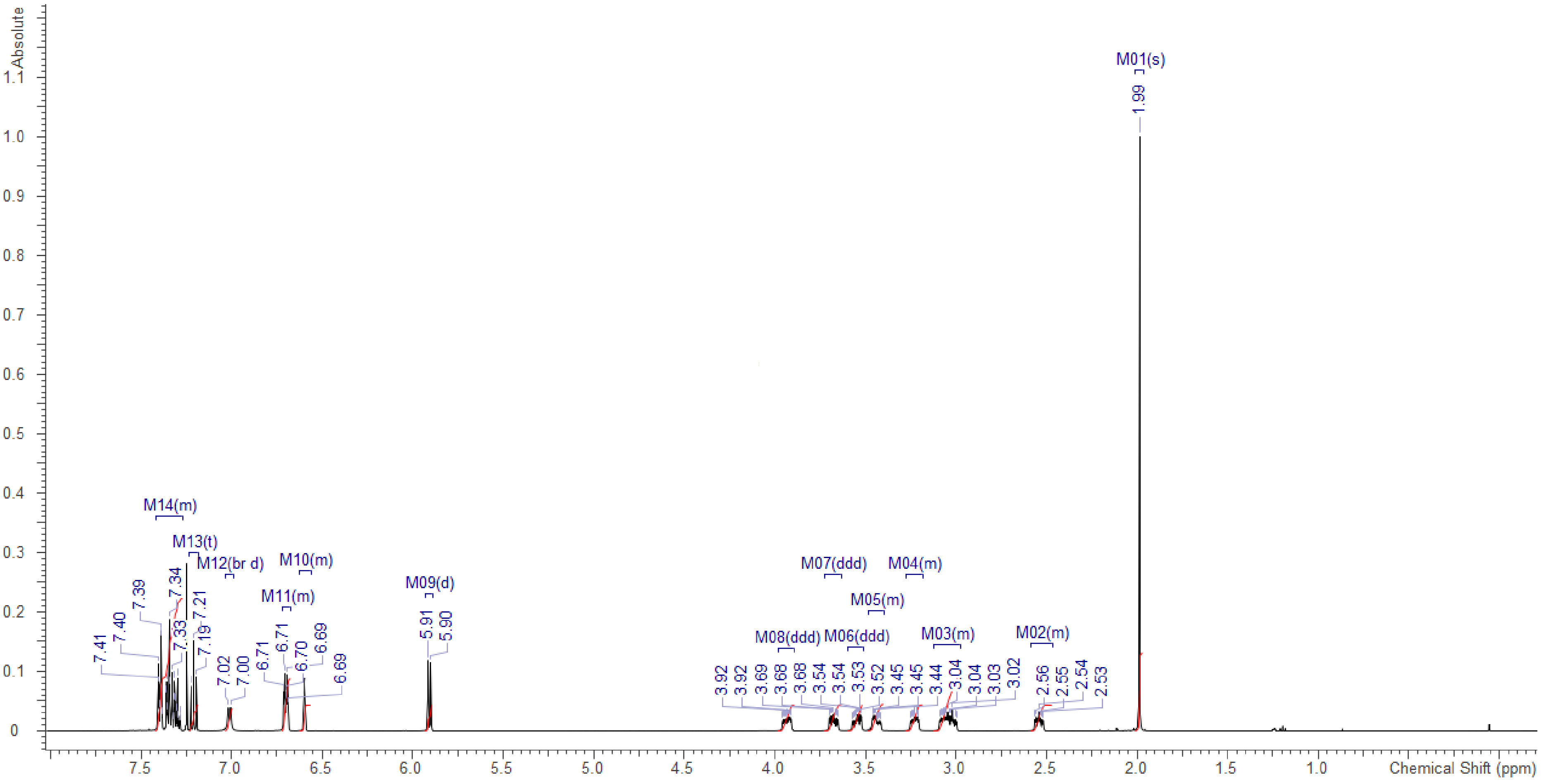

(*S*)-*N*-(2-oxo-1-phenyl-2-(4-(3-(trifluoromethoxy)phenyl)piperazin-1-yl)ethyl)acetamide ((*S*)-32) – ^{13}C NMR

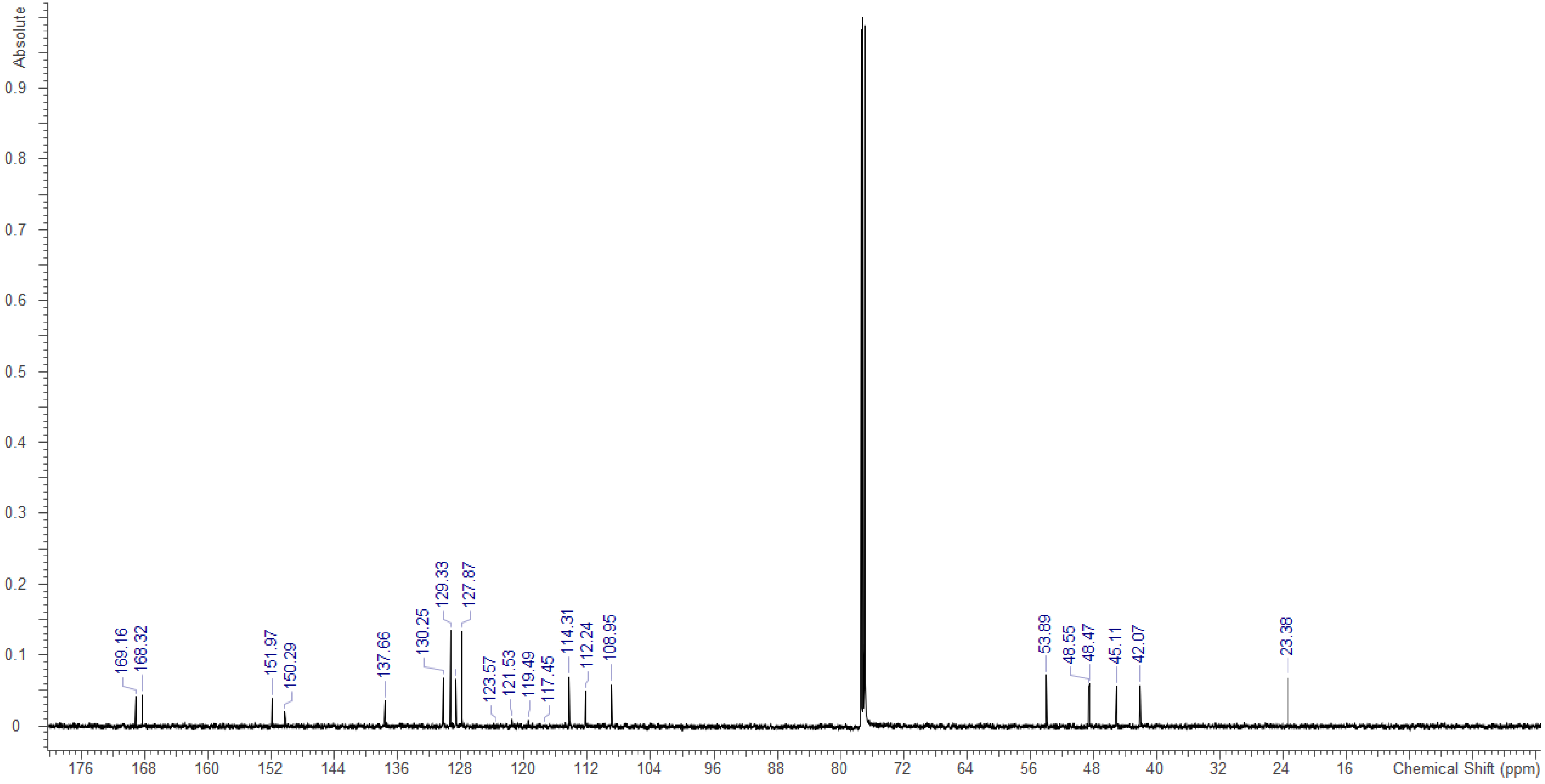

(*R*)-*N*-(2-oxo-1-phenyl-2-(4-(3-((trifluoromethyl)thio)phenyl)piperazin-1-yl)ethyl)acetamide ((*R*)-33) – ^{1}H NMR

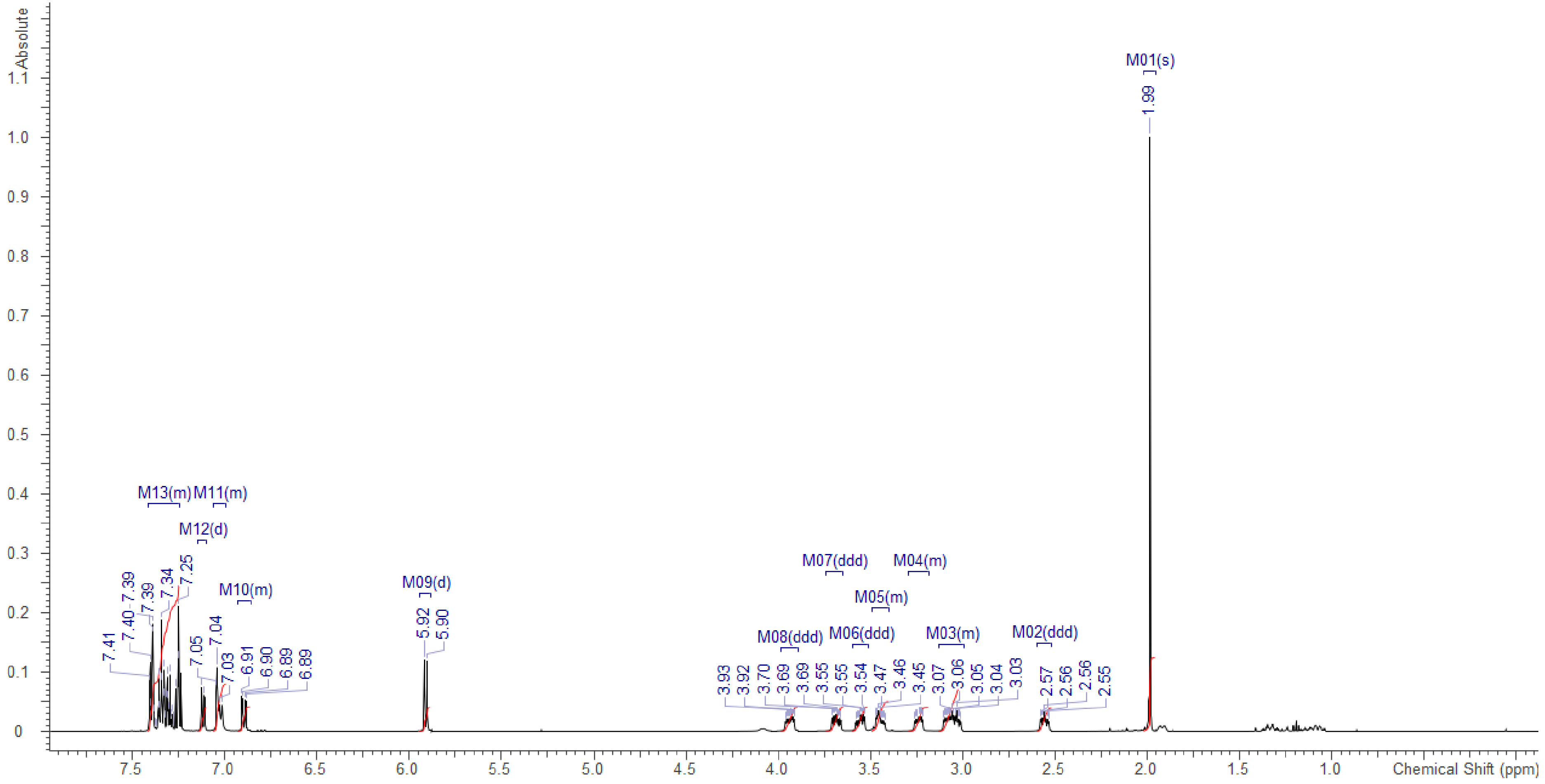

(*R*)-*N*-(2-oxo-1-phenyl-2-(4-(3-((trifluoromethyl)thio)phenyl)piperazin-1-yl)ethyl)acetamide ((*R*)-33) – ^{13}C NMR

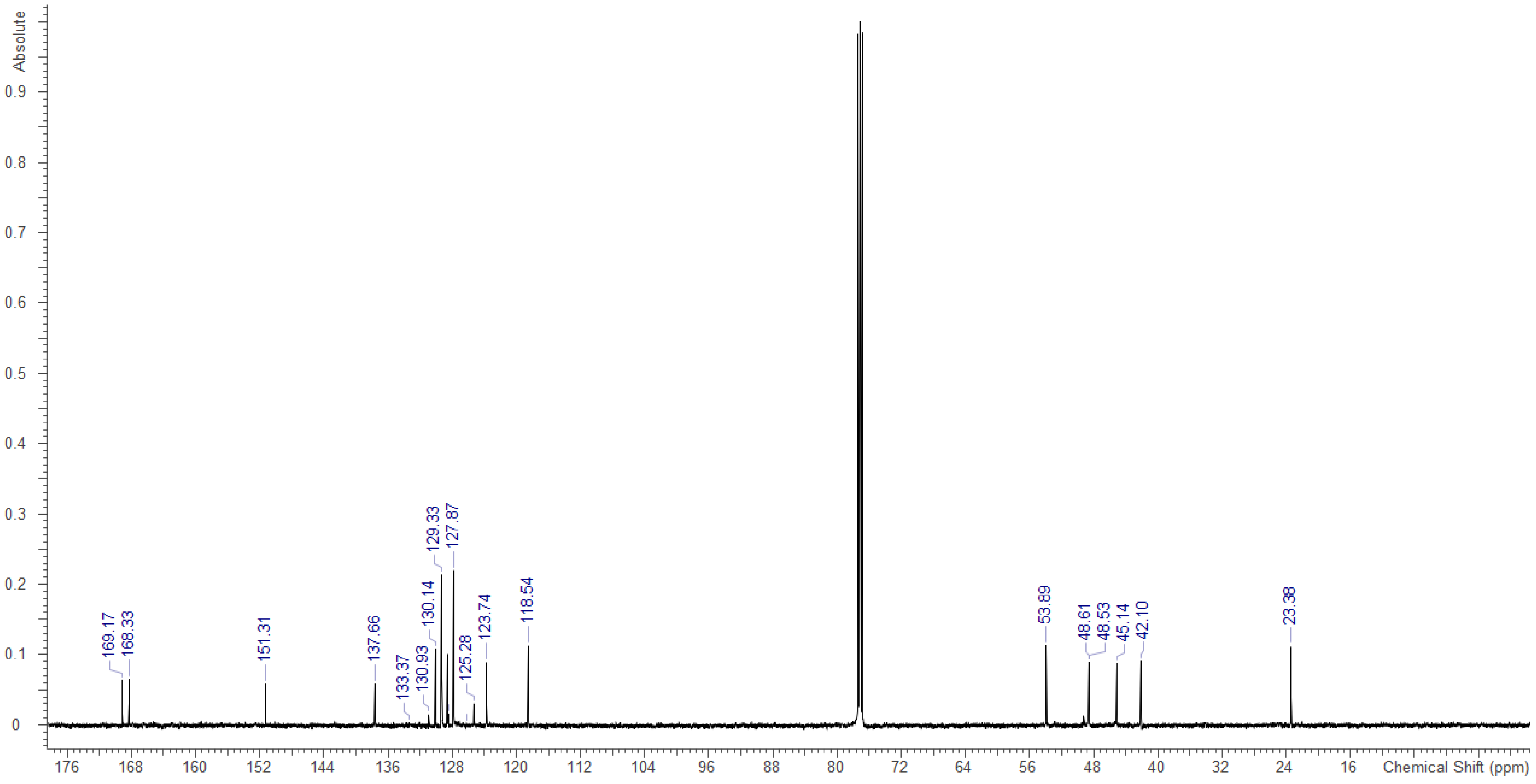

(*S*)-*N*-(2-oxo-1-phenyl-2-(4-(3-((trifluoromethyl)thio)phenyl)piperazin-1-yl)ethyl)acetamide ((*S*)-33) – ^{1}H NMR

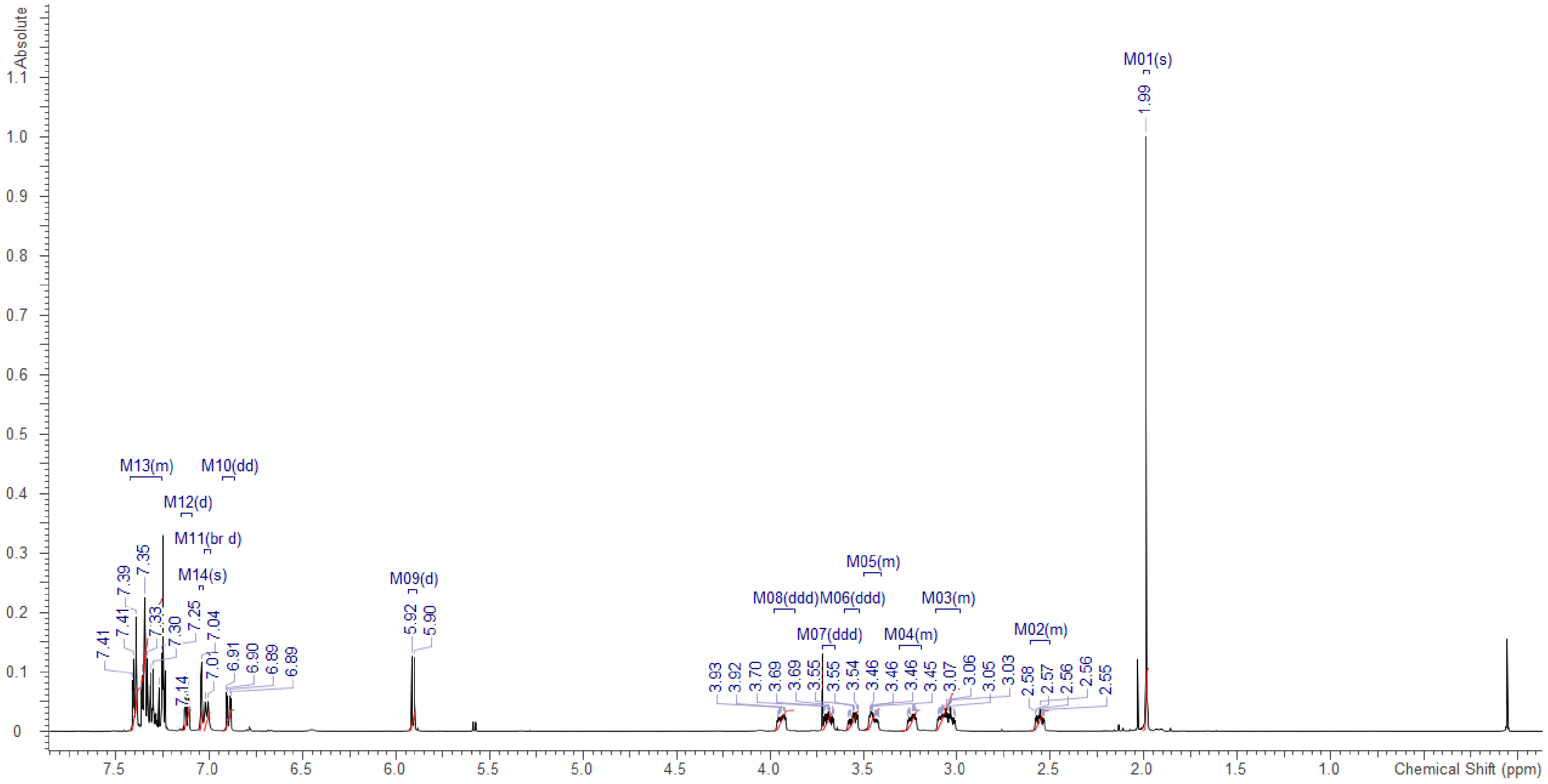

(*S*)-*N*-(2-oxo-1-phenyl-2-(4-(3-((trifluoromethyl)thio)phenyl)piperazin-1-yl)ethyl)acetamide ((*S*)-33) – ^{13}C NMR

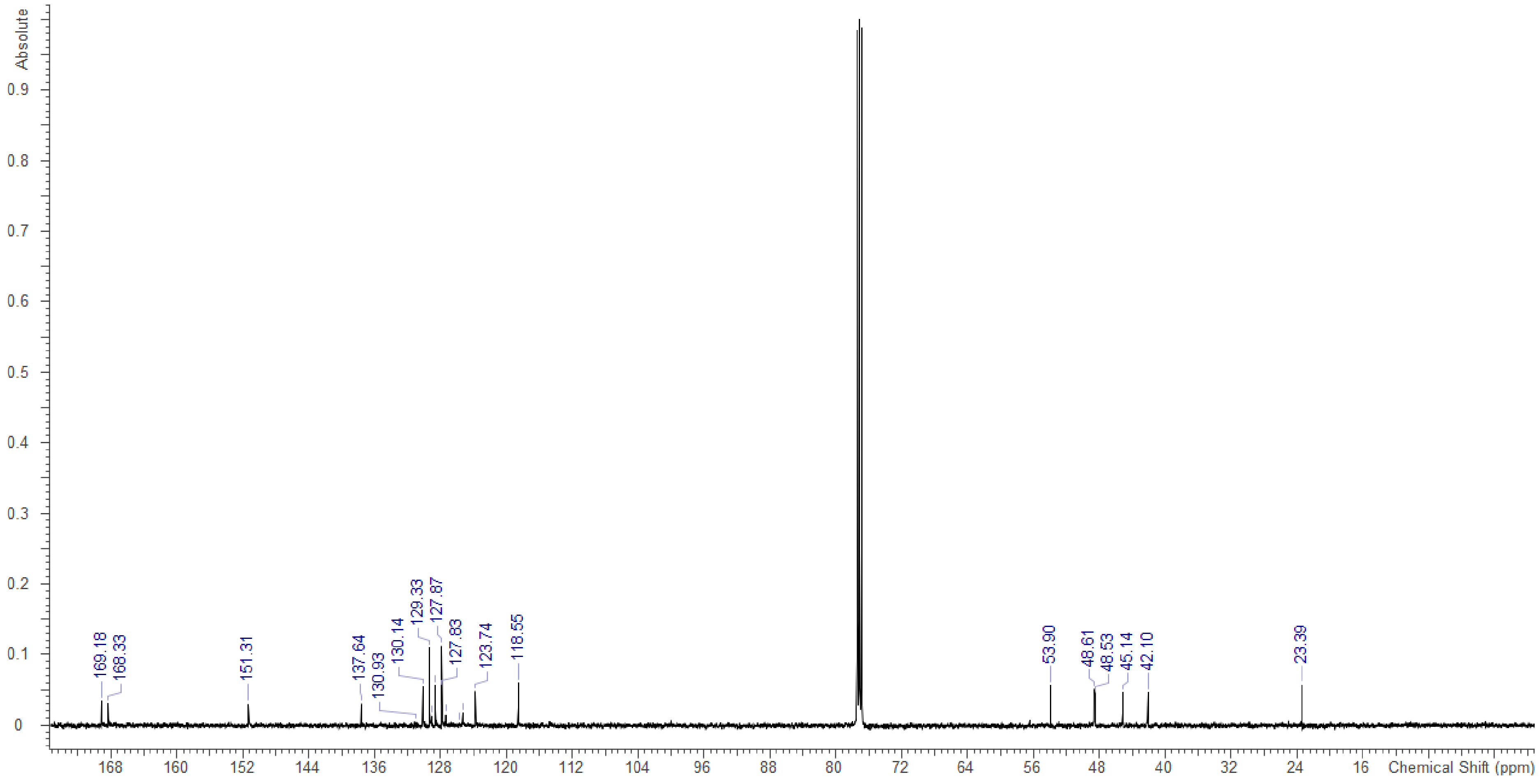

Chiral HPLC traces for target final compounds

(*R*,*S*)-*N*-(2-oxo-1-phenyl-2-(4-(3-(trifluoromethyl)phenyl)piperazin-1-yl)ethyl)acetamide ((*R*,*S*)-31, KJ-5)

SHIMADZU LabSolutions **Analysis Report**

<Sample Information>

Sample Name : R,S-KJ-5
Sample ID :
Data Filename : R,S-KJ-5.lcd
Method Filename : chiralne_KA_.lcm
Batch Filename :
Vial # : 1-45
Sample Type : Unknown
Injection Volume : 10 uL
Date Acquired : 11/23/2022 11:00:34 AM
Acquired by : System
Date Processed : 11/23/2022 11:41:07 AM
Processed by : System

<Chromatogram>

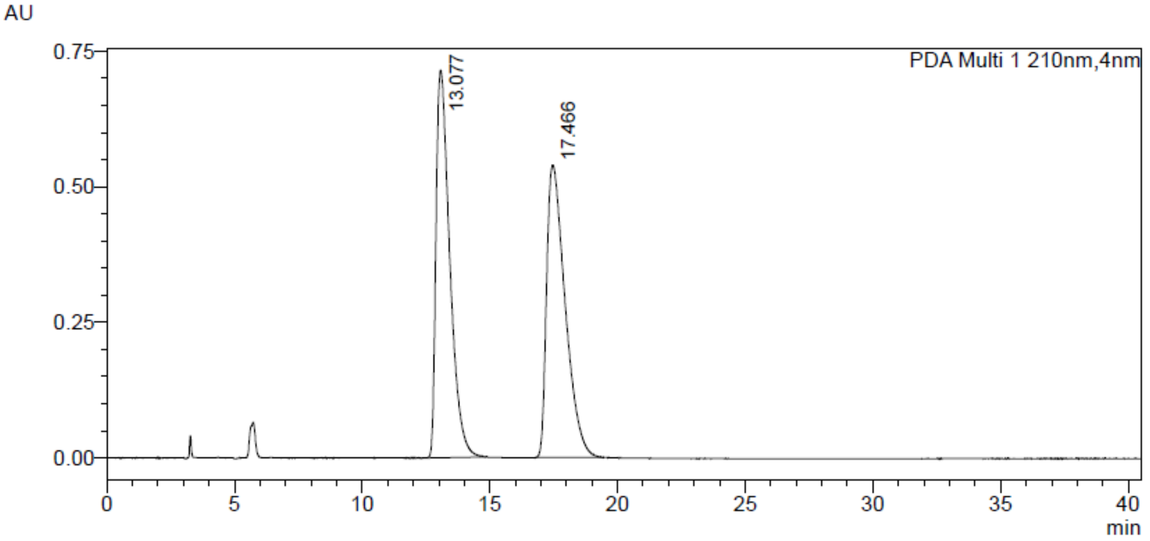

<Peak Table>

PDA Ch1 210nm

Peak#	Ret. Time	Area%
1	13.077	49.619
2	17.466	50.381
Total		100.000

(*R*)-*N*-(2-oxo-1-phenyl-2-(4-(3-(trifluoromethyl)phenyl)piperazin-1-yl)ethyl)acetamide ((*R*)-31)

SHIMADZU LabSolutions

Analysis Report

<Sample Information>

Sample Name	: R-KJ-5		
Sample ID	:		
Data Filename	: R-KJ-5.lcd		
Method Filename	: chiralne_KA_.lcm		
Batch Filename	:		
Vial #	: 1-46	Sample Type	: Unknown
Injection Volume	: 10 uL		
Date Acquired	: 11/23/2022 11:43:06 AM	Acquired by	: System
Date Processed	: 11/23/2022 12:35:37 PM	Processed by	: System

<Chromatogram>

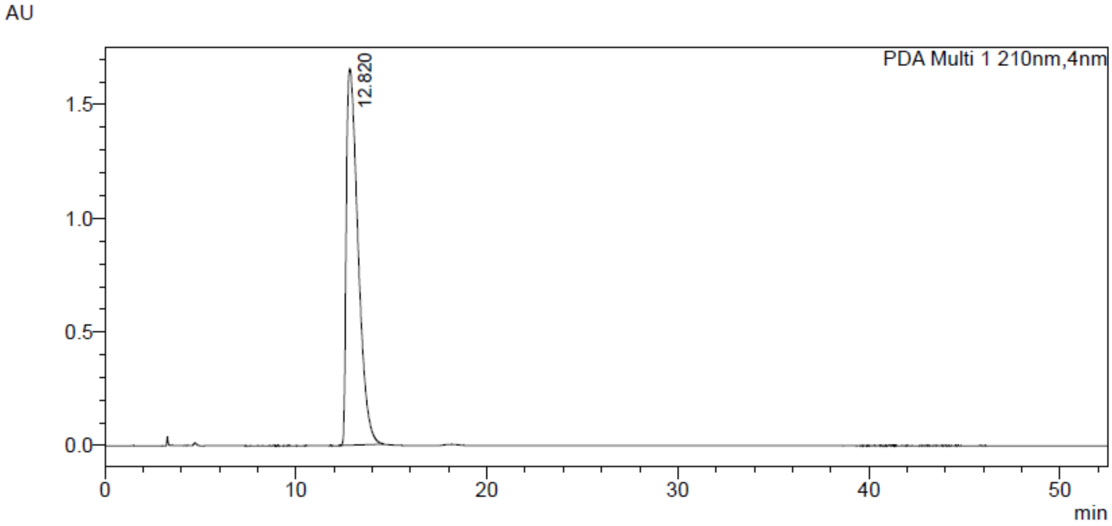

<Peak Table>

PDA Ch1 210nm

Peak#	Ret. Time	Area%
1	12.820	100.000
Total		100.000

(*S*)-*N*-(2-oxo-1-phenyl-2-(4-(3-(trifluoromethyl)phenyl)piperazin-1-yl)ethyl)acetamide ((*S*)-31)

SHIMADZU LabSolutions

Analysis Report

<Sample Information>

Sample Name	: S-KJ-5		
Sample ID	:		
Data Filename	: S-KJ-5.lcd		
Method Filename	: chiralne_KA_.lcm		
Batch Filename	:		
Vial #	: 1-47	Sample Type	: Unknown
Injection Volume	: 10 uL		
Date Acquired	: 11/23/2022 12:43:10 PM	Acquired by	: System
Date Processed	: 11/23/2022 1:23:21 PM	Processed by	: System

<Chromatogram>

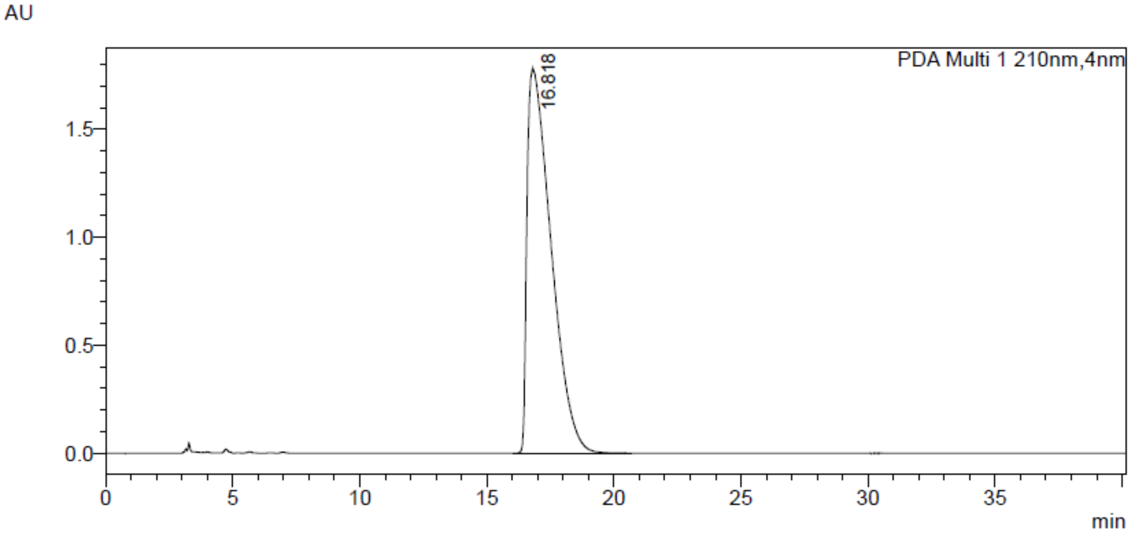

<Peak Table>

PDA Ch1 210nm

Peak#	Ret. Time	Area%
1	16.818	100.000
Total		100.000

(*R,S*)-*N*-(2-oxo-1-phenyl-2-(4-(3-(trifluoromethoxy)phenyl)piperazin-1-yl)ethyl)acetamide ((*R,S*)-32, KJ-28)

SHIMADZU LabSolutions

Analysis Report

<Sample Information>

Sample Name	: R,S-KJ-28		
Sample ID	:		
Data Filename	: R,S-KJ-28.lcd		
Method Filename	: chiralne_KA_.lcm		
Batch Filename	:		
Vial #	: 1-43	Sample Type	: Unknown
Injection Volume	: 10 uL		
Date Acquired	: 11/23/2022 9:23:14 AM	Acquired by	: System
Date Processed	: 11/23/2022 10:05:53 AM	Processed by	: System

<Chromatogram>

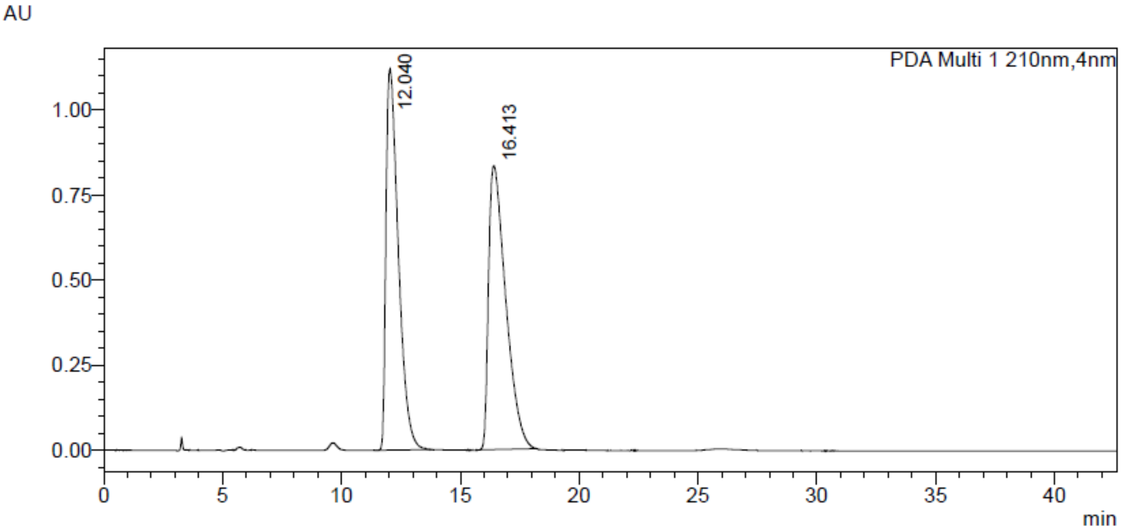

<Peak Table>

PDA Ch1 210nm

Peak#	Ret. Time	Area%
1	12.040	49.182
2	16.413	50.818
Total		100.000

(*R*)-*N*-(2-oxo-1-phenyl-2-(4-(3-(trifluoromethoxy)phenyl)piperazin-1-yl)ethyl)acetamide ((*R*)-32)

SHIMADZU LabSolutions **Analysis Report**

<Sample Information>

Sample Name	: R-KJ-28		
Sample ID	:		
Data Filename	: R-KJ-28		
Method Filename	: chiralne_KA_.lcm		
Batch Filename	:		
Vial #	: 1-44	Sample Type	: Unknown
Injection Volume	: 10 uL		
Date Acquired	: 11/23/2022 10:07:34 AM	Acquired by	: System
Date Processed	: 11/23/2022 10:49:20 AM	Processed by	: System

<Chromatogram>

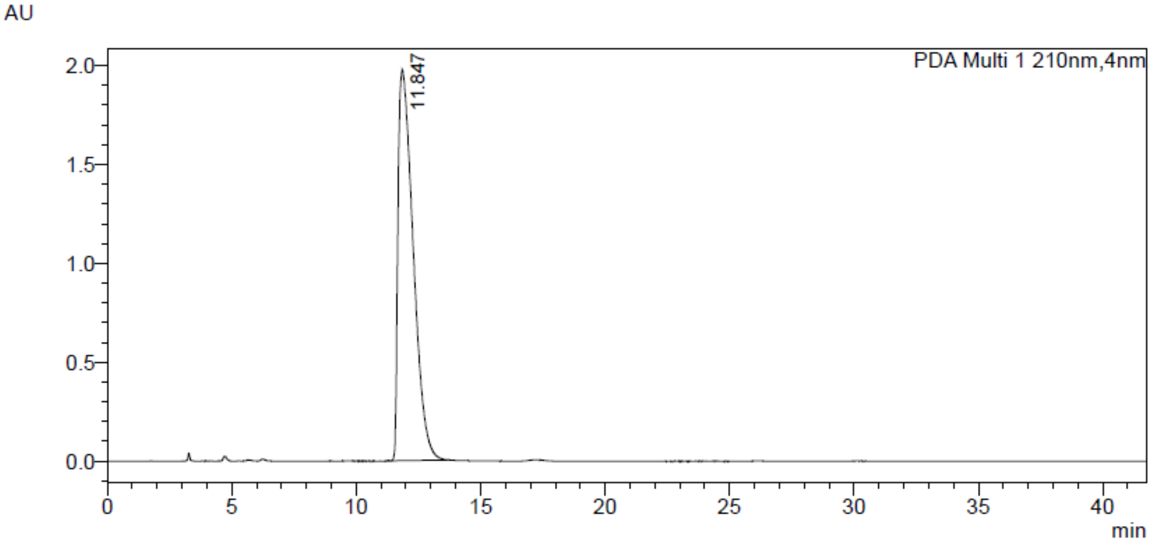

<Peak Table>

PDA Ch1 210nm

Peak#	Ret. Time	Area%
1	11.847	100.000
Total		100.000

(*S*)-*N*-(2-oxo-1-phenyl-2-(4-(3-(trifluoromethoxy)phenyl)piperazin-1-yl)ethyl)acetamide ((*S*)-32)

Analysis Report

<Sample Information>

Sample Name	: S-KJ-28		
Sample ID	:		
Data Filename	: S-KJ-28.lcd		
Method Filename	: chiralne_KA_.lcm		
Batch Filename	:		
Vial #	: 1-14	Sample Type	: Unknown
Injection Volume	: 10 uL		
Date Acquired	: 12/6/2022 10:48:42 AM	Acquired by	: System
Date Processed	: 12/6/2022 11:33:04 AM	Processed by	: System

<Chromatogram>

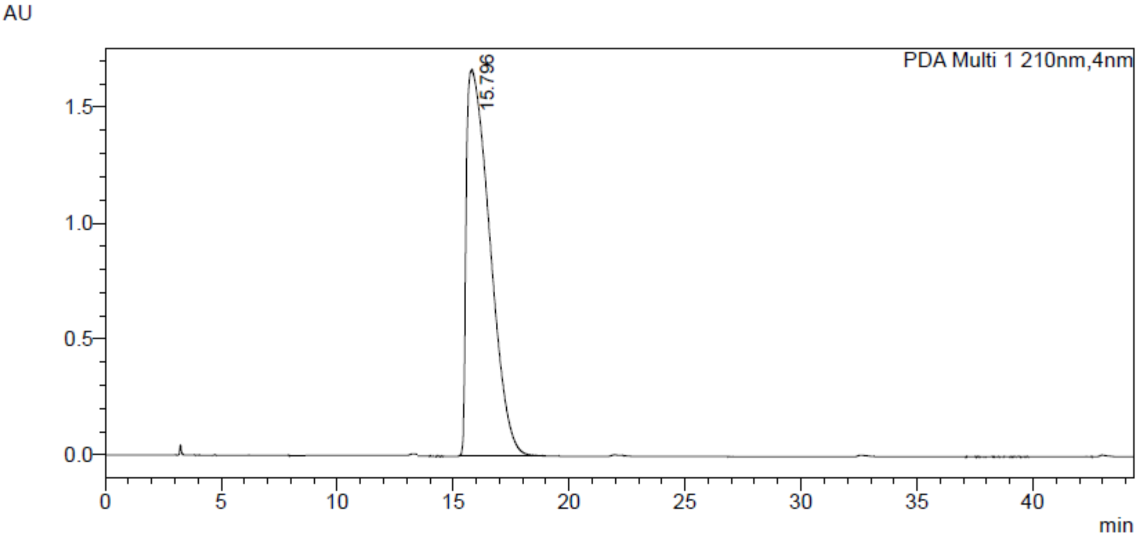

<Peak Table>

PDA Ch1 210nm

Peak#	Ret. Time	Area%
1	15.796	100.000
Total		100.000

(*R*,*S*)-*N*-(2-oxo-1-phenyl-2-(4-(3-((trifluoromethyl)thio)phenyl)piperazin-1-yl)ethyl)acetamide ((*R*,*S*)-33, KJ-37)

SHIMADZU LabSolutions **Analysis Report**

<Sample Information>

Sample Name	: R,S-KJ-37		
Sample ID	:		
Data Filename	: R,S-KJ-37.lcd		
Method Filename	: chiralne_KA_.lcm		
Batch Filename	:		
Vial #	: 1-40	Sample Type	: Unknown
Injection Volume	: 10 uL		
Date Acquired	: 11/21/2022 12:00:03 PM	Acquired by	: System
Date Processed	: 11/21/2022 12:40:05 PM	Processed by	: System

<Chromatogram>

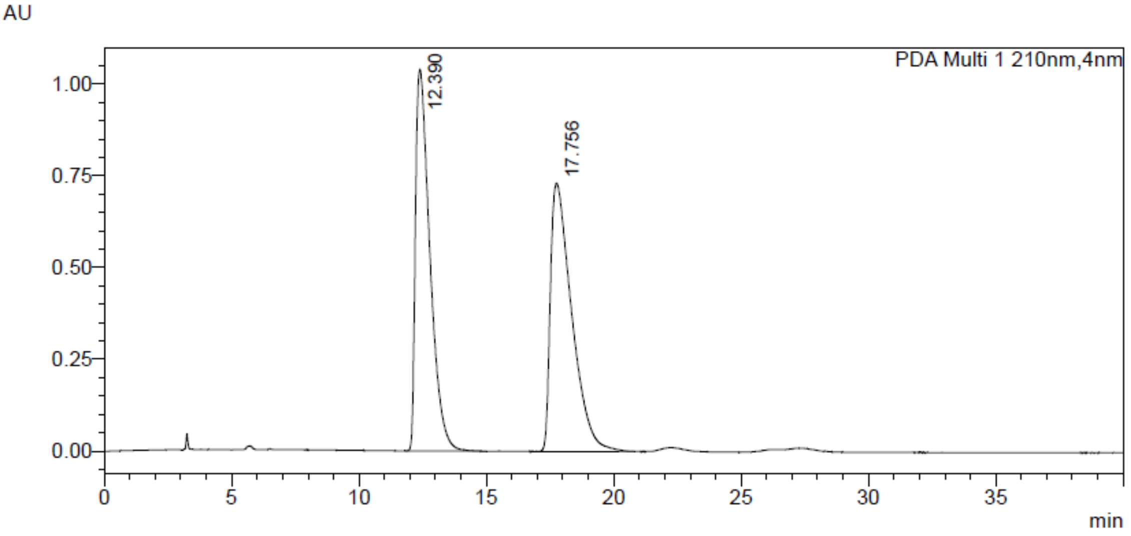

<Peak Table>

PDA Ch1 210nm

Peak#	Ret. Time	Area%
1	12.390	49.114
2	17.756	50.886
Total		100.000

(*R*)-*N*-(2-oxo-1-phenyl-2-(4-(3-((trifluoromethyl)thio)phenyl)piperazin-1-yl)ethyl)acetamide ((*R*)-33)

SHIMADZU LabSolutions

Analysis Report

<Sample Information>

Sample Name : R-KJ-37
Sample ID :
Data Filename : R-KJ-37.lcd
Method Filename : chiralne_KA_.lcm
Batch Filename :
Vial # : 1-41 Sample Type : Unknown
Injection Volume : 10 uL
Date Acquired : 11/21/2022 2:01:07 PM Acquired by : System
Date Processed : 11/21/2022 2:43:42 PM Processed by : System

<Chromatogram>

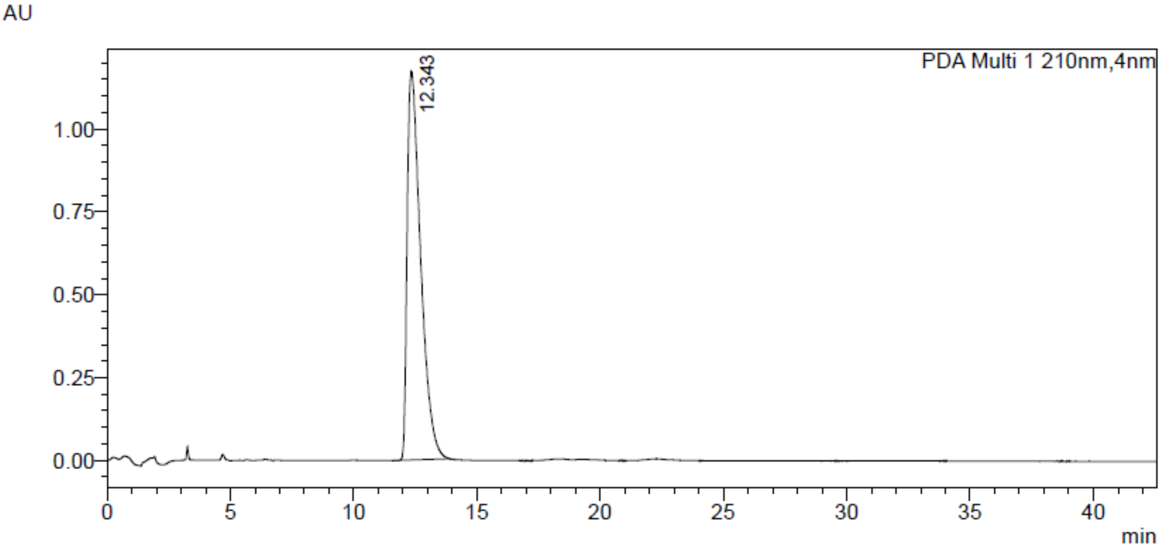

<Peak Table>

PDA Ch1 210nm

Peak#	Ret. Time	Area%
1	12.343	100.000
Total		100.000

(*S*)-*N*-(2-oxo-1-phenyl-2-(4-(3-((trifluoromethyl)thio)phenyl)piperazin-1-yl)ethyl)acetamide ((*S*)-33)

Analysis Report

<Sample Information>

Sample Name	: S-KJ-37		
Sample ID	:		
Data Filename	: S-KJ-37.lcd		
Method Filename	: chiralne_KA_.lcm		
Batch Filename	:		
Vial #	: 1-42	Sample Type	: Unknown
Injection Volume	: 10 uL		
Date Acquired	: 11/21/2022 2:45:18 PM	Acquired by	: System
Date Processed	: 11/21/2022 3:26:57 PM	Processed by	: System

<Chromatogram>

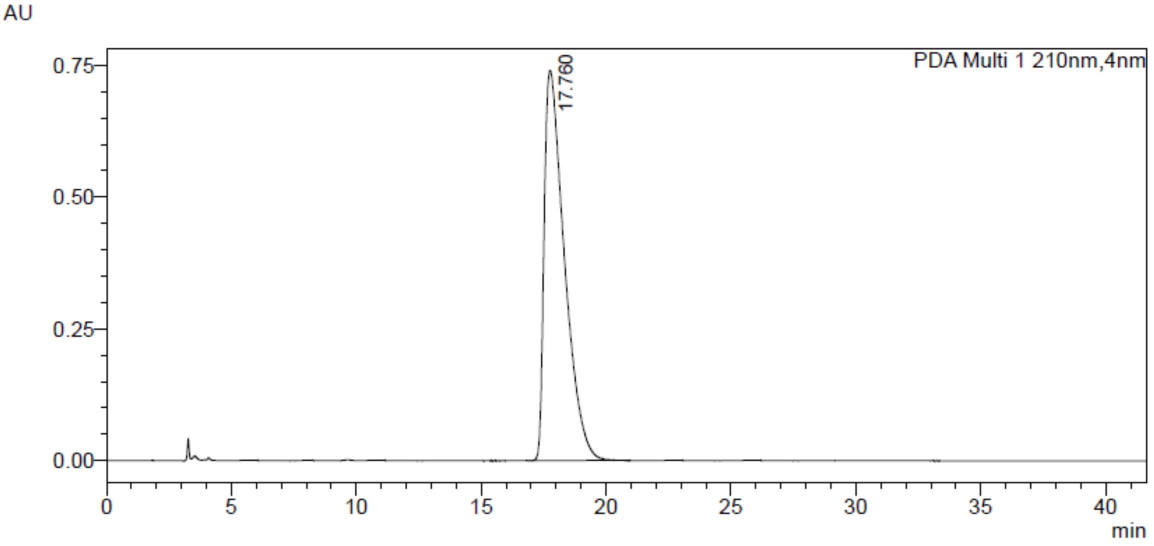

<Peak Table>

PDA Ch1 210nm

Peak#	Ret. Time	Area%
1	17.760	100.000
Total		100.000
